# Aerobic synthesis of *N*-sulfonylamidines mediated by N-heterocyclic carbene copper(I) catalysts

**DOI:** 10.3762/bjoc.16.43

**Published:** 2020-03-24

**Authors:** Faïma Lazreg, Marie Vasseur, Alexandra M Z Slawin, Catherine S J Cazin

**Affiliations:** 1 School of Chemistry, University of St Andrews, St Andrews, KY16 9ST, UK; 2 Centre for Sustainable Chemistry and Department of Chemistry, Ghent University, Krijgslaan 281-S3, 9000 Ghent, Belgium

**Keywords:** catalysis, copper, copper catalysis, N-heterocyclic carbene, solvent-free, sulfonylamidines

## Abstract

A new catalytic strategy for the one-pot synthesis of *N*-sulfonylamidines is described. The cationic copper(I) complexes were found to be highly active and efficient under mild conditions in air and in the absence of solvent. A copper acetylide is proposed as key intermediate in this transformation.

## Introduction

**Scheme 1:**
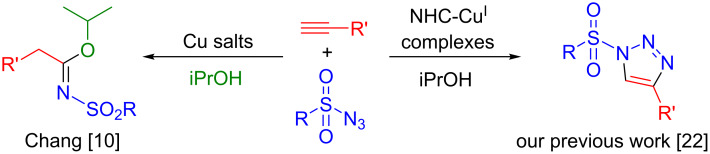
Formation of sulfonyltriazoles and sulfonamidines.

Amide derivatives represent a ubiquitous molecular construct in chemistry [1–3]. This structural motif favours rearrangements that lead to high reactivity and associated bioactivity [4–5]. Indeed, the presence of an *N*-atom in the amidine structure leads to opportunities as ligands and organocatalysts [6–8]. *N*-Sulfonylamidines and *N*-sulfonylimidates are members of a specific class of these amidines. One initial methodology developed for the formation of sulfonylamidines was based on the cleavage of the bond between the N-4 and C-benzene in thiadiazine ring-type molecules [9]. To date, only few examples of copper-based catalysts have been reported to enable access to such compounds [10–13]. Chang and co-workers were pioneers in this area [10–12]. A three-component reaction between alkyne, sulfonyl azide and amine/alcohol was described as a synthetic route to generate sulfonyltriazole intermediates. However, the presence of additives and high catalyst loading (CuI 10 mol %) were required for the synthesis of *N*-sulfonylimidates (Scheme 1, left).

Over the last two decades, NHCs (NHC = N-heterocyclic carbene) have become ligands of choice to permit the stabilisation and formation of highly reactive transition metal species [14]. Thus, significant advances have been achieved using this supporting ligand family [15–19]. Recently, our group contributed to this area, reporting on the synthesis of the first heteroleptic bis-NHC and mixed NHC/phosphine copper(I) complexes [20–22]. Interestingly, these new copper-based complexes have shown excellent activity in the [3 + 2] cycloaddition reaction of azides/sulfonyl azides and alkynes (Scheme 1, right) [22]. Based on these earlier results, the reactivity of these catalysts was investigated in the context of achieving formation of the challenging sulfonamide derivatives.

Herein, we report the high efficiency of cationic copper(I) complexes for the formation of *N*-sulfonylamidines via a three-component reaction performed in air, using solvent-free conditions and in the absence of any additive.

## Results and Discussion

**Figure 1:**
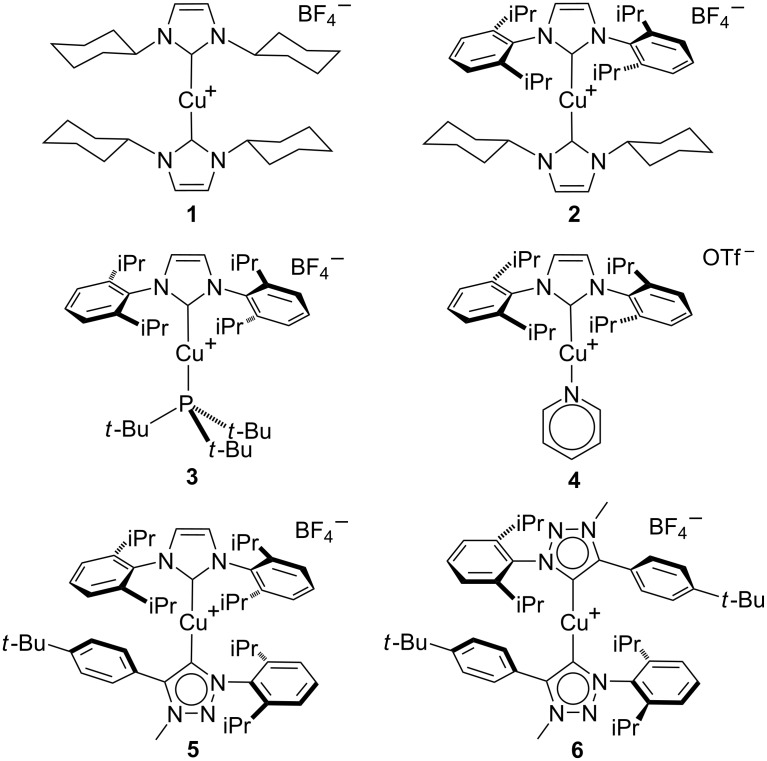
Catalytic systems used in this study.

[Cu(ICy)_2_]BF_4_ (**1**), [Cu(IPr)(ICy)]BF_4_ (**2**) and [Cu(IPr)(P*t-*Bu_3_)]BF_4_ (**3**) were initially selected as optimum candidates [22]. This class of catalysts was expanded through the synthesis of the pyridine derivative **4**, of the heteroleptic normal/mesoionic carbene complex **5**, and of the homoleptic mesoionic triazole derivative **6** (Figure 1). This special class of ligands presents unique electronic and steric properties and lead to unusual reactivity [23–28].

**Scheme 2:**
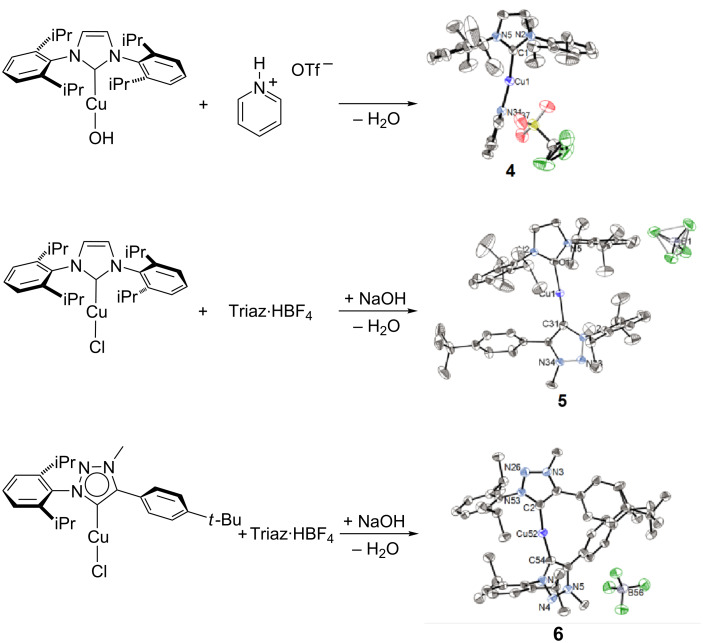
Synthetic access to complexes **4**–**6** [30].

[Cu(IPr)(Pyr)]OTf (**4**) was obtained by the reaction of the isolated hydroxide derivative [Cu(IPr)(OH)] [29] with pyridinium trifluoromethanesulfonate, while the biscarbene complexes **5** and **6** were obtained from the corresponding [Cu(NHC)Cl] through the in situ formation of the corresponding hydroxide complex [Cu(NHC)(OH)] [20] which deprotonates the triazolium salt (Scheme 2).

The reactivity of a series of cationic copper(I) complexes (**1**–**6**) was evaluated at 0.5 mol % loading using tosyl azide, phenylacetylene and diisopropylamine as benchmark substrates [31–32]. Various solvents were evaluated at room temperature under aerobic conditions (see Supporting Information File 1 for details). Tetrahydrofuran (THF), 1,2-DCE, 1,4-dioxane and acetonitrile proved to be the most suitable solvents for this transformation (Table 1). Interestingly, similar results were obtained for complexes **1**–**5**, while **6** displayed superior activity. Indeed, 71% conversion to the desired product was observed using the homoleptic cationic MIC (mesoionic carbene) complex **6** (Table 1, entry 6). For complexes **1**–**5**, the conversion proved modest and ranged between 42 and 49% (Table 1, entries 1–5).

**Table 1:** Catalyst and solvent optimisation.^a,b^

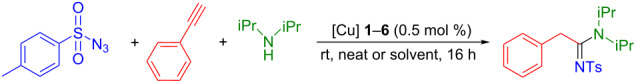			

Entry	Complex	Solvent	Conv.^c^ (%)

1	**1**	THF	49
2	**2**	THF	47
3	**3**	THF	42
4	**4**	THF	42
5	**5**	THF	46
6	**6**	THF	71
7	**6**	neat	65
8	**6**	1,2-DCE	63
9	**6**	water	41
10	**6**	1,4-dioxane	58
11	**1**	neat	30
12	**2**	neat	58
13	**5**	neat	55

^a^Reaction conditions: phenylacetylene (0.5 mmol), tosyl azide (0.6 mmol), diisopropylamine (0.6 mmol), [Cu] (0.5 mol %), solvent (1 mL), 16 hours. ^b^See Supporting Information File 1 for full optimisation. ^c^Conversion was determined by GC analysis based on phenylacetylene using mesitylene (42 µL) as internal standard.

**Scheme 3:**
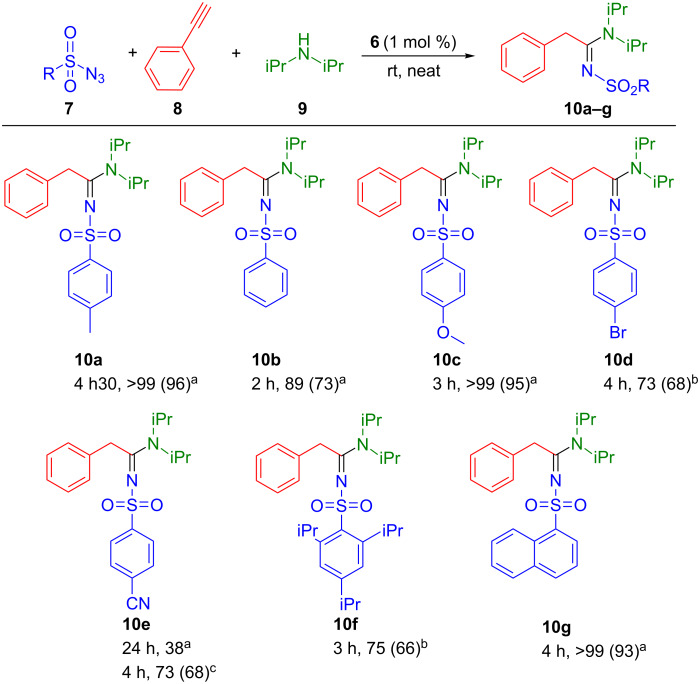
Variation of sulfonylazides. Reaction conditions: phenylacetylene (0.5 mmol), sulfonyl azide (0.6 mmol), diisopropylamine (0.6 mmol), **6** (1 mol %). Conversion determined by ^1^H NMR based on alkyne using mesitylene (42 µL) as internal standard. Isolated yield in parentheses. ^a^Solvent-free conditions, rt. ^b^Solvent-free conditions, 40 °C. ^c^THF (1 mL), rt.

Subsequently, solvent-free conditions were investigated (Table 1, entries 7 and 11–13). Interestingly, the absence of solvent proved to be highly effective, except for [Cu(ICy)_2_]BF_4_ **1** (Table 1 entry 11). An encouraging 65% conversion was obtained using [Cu(Triaz)_2_]BF_4_ **6** (Table 1, entry 7), while complexes **2** and **5** showed comparable results (Table 1, entries 12 and 13). Complex **6** was also shown to be active in water and in 1,4-dioxane. Based on these results, a reaction scope was conducted under solvent-free conditions, in air, using 1 mol % of [Cu(Triaz)_2_]BF_4_ **6** (Scheme 3).

Functionalised azides were reacted with phenylacetylene and diisopropylamine resulting in good to high yields (Scheme 3, **10a**–**g**). The presence of an activating/deactivating group in *para*-position of the aryl ring was evaluated in order to assess the substrate tolerance. Electron-donating groups, such as methyl, methoxy or naphthyl enhanced considerably the reactivity leading to quantitative conversion with respectively 96% (**10a**), 95% (**10c**) and 93% (**10g**) isolated yields, in reaction times of 3 to 4.5 hours. Regarding the 2,4,6-triisopropylsulfonyl azide, a slight decrease in the reactivity was observed (66%, **10f**), presumably due to the steric hindrance of the substrate. Electron-withdrawing groups such as bromo (**10d**) or cyano (**10e**) appeared to disfavour the reaction resulting in lower yields. Indeed for the cyanosulfonyl azide, under solvent-free conditions, only 38% of the desired product was obtained after 24 hours. This lower yield could also be due to the starting material being a solid which leads to poorer mixing and mass transport issues. Interestingly, when conducted in THF, an increase to 68% isolated yield was observed after 4 hours, supporting our inhomogeneity/transport hypothesis. In the case of the bromosulfonyl azide, a 73% conversion was obtained after 4 hours at 40 °C.

**Scheme 4:**
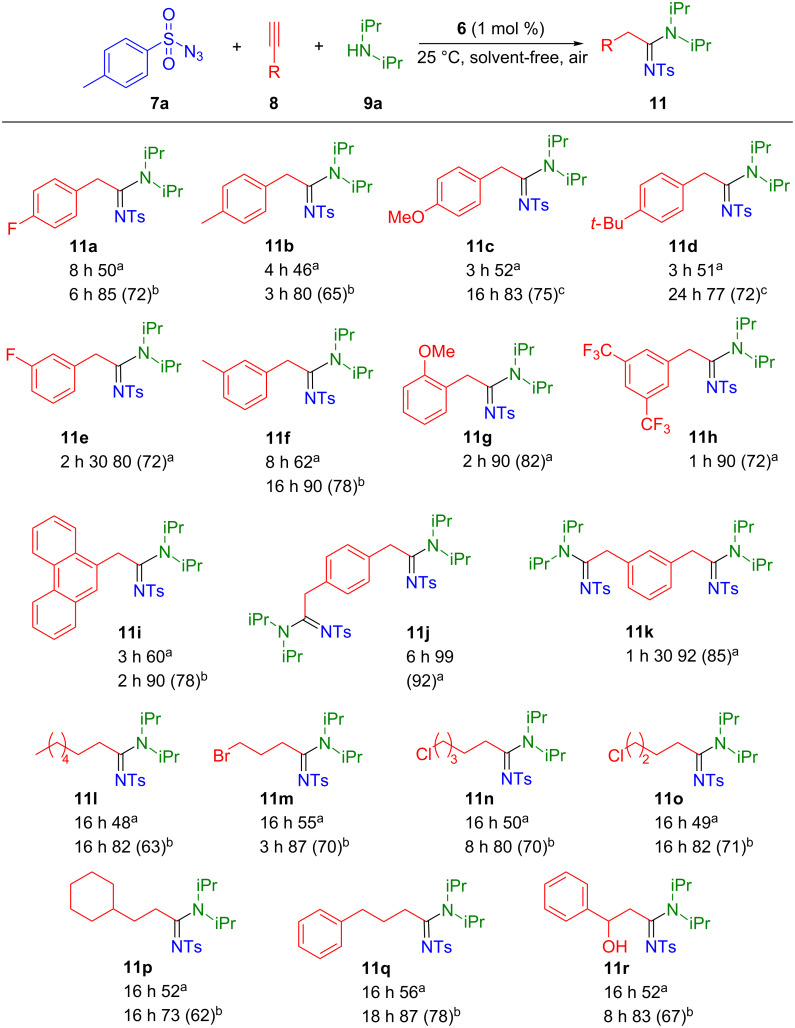
Variation of alkynes. Reaction conditions: alkyne (0.5 mmol), tosyl azide (0.6 mmol), diisopropylamine (0.6 mmol), **6** (1 mol %). Conversion determined by ^1^H NMR based on alkyne using mesitylene (42 µL) as internal standard. Isolated yield in parenthesis. ^a^Solvent-free conditions, rt. ^b^Solvent-free conditions, 40 °C. ^c^THF (1 mL), rt.

Various terminal aryl/alkyl-substituted alkynes were investigated in the presence of tosyl azide and diisopropylamine (Scheme 4). Under standard conditions, good to excellent yields were obtained. The presence of functional groups in *para*-position of the aryl ring leads to a decrease of the conversion to approximately 50% (**11a**, **11b**, **11c** and **11d**). The reactivity was considerably enhanced by increasing the temperature to 40 °C and/or the use of THF as reaction solvent. Interestingly, *ortho-* and *meta*-substitution of the aryl rings are well tolerated (**11e**–**i**). In the case of 9-ethynylphenanthrene, which is a solid substrate, solvent-free conditions lead to 78% isolated yield at 40 °C. Diynes were also investigated and excellent isolated yields were achieved (92% and 85% for **11j** and **11k**, respectively). Regarding the alkyl-alkynes, longer reactions times as well as higher temperature were required to reach high conversion. Interestingly, in the case of **11r**, the desired product was obtained in 67% isolated yield.

**Scheme 5:**
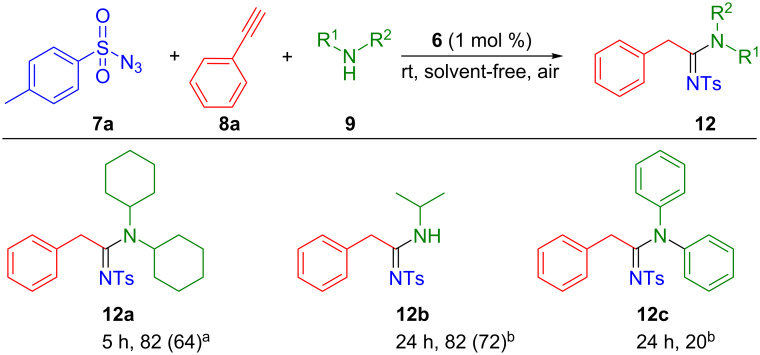
Variation of the amine substrate. Reaction conditions: phenylacetylene (0.5 mmol), tosyl azide (0.6 mmol), amine (0.6 mmol), **6** (1 mol %). Conversion determined by ^1^H NMR based on alkyne using mesitylene (42 µL) as internal standard. Isolated yield in parenthesis. ^a^Solvent-free conditions, 40 °C. ^b^THF (1 mL), rt.

The effect of the amines was also investigated. Amongst the amines evaluated, dicyclohexylamine (for **12a**) and isopropylamine (for **12b**) lead to good isolated yields (64% and 72%, Scheme 5). In contrast, with diphenylamine, only 20% of the desired product was observed (**12c**).

**Scheme 6:**
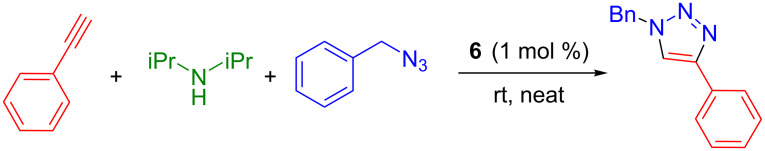
Reactivity of “non-sulfonyl” azide [33]. Reaction conditions: phenylacetylene (0.5 mmol), benzyl azide (0.6 mmol), diisopropylamine (0.6 mmol), 24 h.

Interestingly, with benzyl azide, a substrate not containing a sulfonyl moiety, the product obtained is the 1,2,3-triazole derivative [33], resulting from a [3 + 2] cycloaddition of azide and alkyne (Scheme 6).

**Scheme 7:**
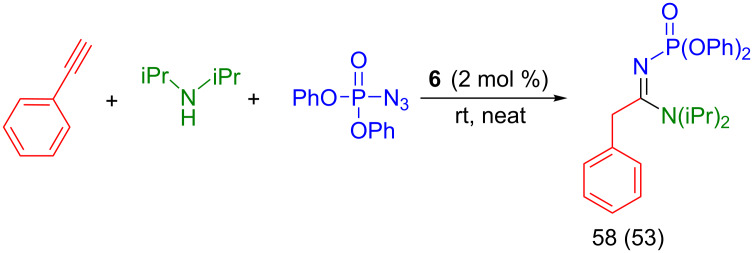
Reactivity of diphenylphosphoryl azide. Reaction conditions: phenylacetylene (0.5 mmol), diphenylphosphoryl azide (0.6 mmol), diisopropylamine (0.6 mmol), [Cu] (2 mol %), 24 h. Conversion determined by GC based on phenylacetylene using mesitylene (42 µL) as internal standard. Isolated yield in parentheses.

The catalytic system was also shown applicable to phosphoryl azides; and reaction of phenylacetylene with diisopropylamine and diphenylphosphoryl azide leads to the formation of the corresponding phosphorylamidine product [34] in good yield (Scheme 7), using 2 mol % of catalyst under mild conditions (solvent-free, room temperature).

**Scheme 8:**
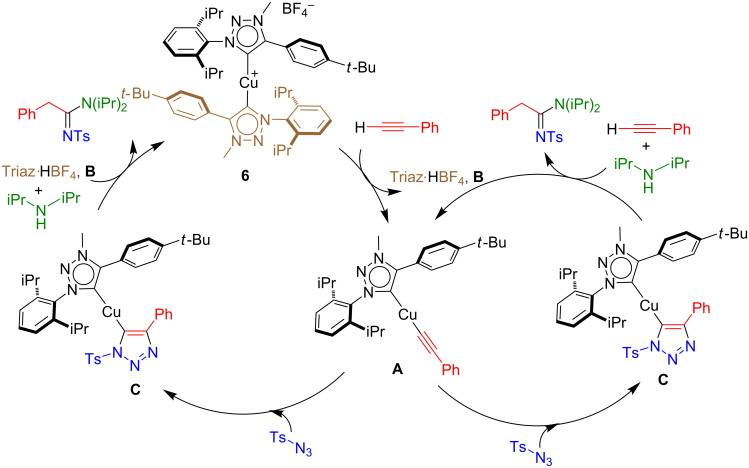
Proposed mechanism for the formation of sulfonamidine.

A proposed reaction mechanism occurring via formation of a copper-acetylide species is proposed and illustrated in Scheme 8. The bis-NHC copper(I) complex **6** reacts with the alkyne leading to the formation of an acetylide derivative **A** (left hand side, Scheme 8), with concomitant loss of a NHC ligand through the formation of the corresponding triazolium salt **B**. The intermediate **A** can then react with the azide substrate to form a triazolyl–copper complex **C**. The latter can liberate the amidine product and regenerate either catalyst **6** (triazolium salt **B** is source of proton) or directly the acetylide complex **A** (phenylacetylene is source of proton).

**Scheme 9:**
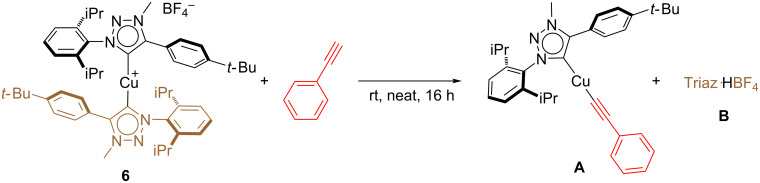
Stoichiometric reaction between **6** and **8**.

**Scheme 10:**
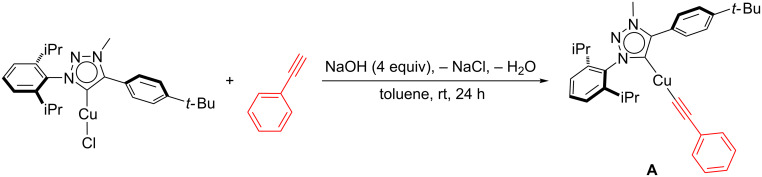
Synthesis of copper-acetylide intermediate **A** via [Cu(Cl)(Triaz)].

In order to support this mechanism, a number of stoichiometric reactions were conducted (see Supporting Information File 1). In a first instance, **6** was reacted with phenylacetylene at room temperature. This led to the rapid formation of the copper acetylide complex **A** with concomitant loss of the triazolium salt Triaz^.^HBF_4_ (**B**, Scheme 9). To further confirm the formation of **A**, [Cu(Triaz)Cl] was reacted with phenylacetylene and sodium hydroxide (4 equiv), in toluene for 24 hours under an inert atmosphere. The independent synthesis of **A** was successfully achieved in this manner (Scheme 10). The latter was then reacted with tosyl azide. An immediate colour change resulted and based on ^1^H NMR data, two new species were formed. They were identified as the sulfonyltriazole and an unstable organometallic compound, presumably the triazolylcopper(I) complex **C**. These results corroborated the proposed hypothesis regarding the formation of a triazole intermediate during the catalytic cycle.

**Scheme 11:**
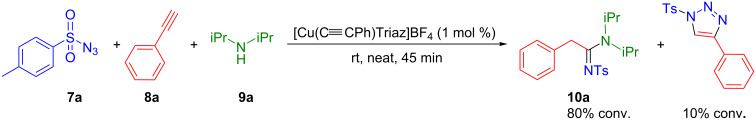
Catalytic reaction involving copper-acetylide complex **A**.

To support the relevance of the Cu-acetylide species in catalysis, the benchmark reaction was conducted at 1 mol % catalyst of the isolated acetylide complex (Scheme 11). After 45 minutes, 80% conversion into the sulfonamidine product was observed. Of note, the presence of sulfonyltriazole was also observed (10%).

## Conclusion

Cationic bis-carbene copper(I) complexes were shown to promote the formation of *N-*sulfonamidines in a Click reaction [35–36]. The new developed mesoionic NHC copper(I) complexes were found highly efficient under solvent-free and aerobic conditions. Stoichiometric reactions support the release of one NHC and the formation of a copper(I) acetylide as key elements in the catalytic cycle.

## Experimental

***N,N’*-Bis{2,6-(diisopropyl)phenyl}imidazol-2-ylidene(pyridine)copper(I) triflate**, **[Cu(IPr)(Pyr)]OTf (4).** In a glovebox, a vial was charged with [Cu(OH)(IPr)] (200 mg, 0.41 mmol), pyridinium trifluoromethanesulfonate (94.0 mg, 1 equiv, 0.41 mmol) and THF (2 mL). The reaction mixture was stirred at room temperature for 15 hours. The solution was concentrated and diethyl ether (10 mL) was added. The precipitate was collected by filtration and washed with diethyl ether (3 × 5 mL). The desired compound was obtained as a colourless solid (201 mg, 92%). ^1^H NMR (400 MHz, CD_2_Cl_2_, 298 K) δ (ppm) 0.82 (d, ^3^*J*_HH_ = 6.9 Hz, 12H, CHC*H*_3_ (IPr)), 1.01 (d, ^3^*J*_HH_ = 6.9 Hz, 12H, CHC*H*_3_ (IPr)), 1.44 (s, 9H, C(C*H*_3_)_3_), 2.36 (septet, ^3^*J*_HH_ = 6.9 Hz, 4H, C*H*CH_3_ (IPr)), 3.98 (s, 3H, C*H*_3_), 7.06 (s, 2H, *H*^4^ and *H*^5^), 7.19 (d, ^3^*J*_HH_ = 7.8 Hz, 4H, C_Ar_*H* (IPr)), 7.50 (t, *^3^J*_HH_ = 7.8 Hz, 2H, C_Ar_*H* (IPr)); ^13^C{^1^H} NMR (100 MHz, CD_2_Cl_2_, 298 K, TMS) δ (ppm) 23.5 (s, CH*C*H_3_ (Triaz)), 24.8 (s, CH*C*H_3_ (Triaz)), 28.8 (s, 2 *C*HCH_3_ (Triaz)), 124.4 (s, *C*_Ar_H), 124.5 (s, *C*_Ar_H), 131.0 (s, *C*_Ar_H), 134.2 (s, *C*_Ar_H), 140.8 (s, *C*^IV^), 145.8 (s, *C*H (IPr)), 148.9 (s, *C*^IV^), 177.5 (s, *C*_carbene_); ^19^F{^1^H} NMR (282 MHz, CDCl_3_, 298 K) δ (ppm) −78.6 (s); anal. calcd for C_33_H_41_CuF_3_N_3_O_3_S: C, 58.26; H, 6.07; N, 6.18; found: C, 58.39; H, 6.16; N, 6.08.

**1-{2,6-(Diisopropyl)phenyl}-3-methyl-4-(4-*tert*-butylphenyl)-1,2,3-triazol-5-ylidene-(*N,N’*-bis{2,6-(diisopropyl)phenyl}imidazol-2-ylidene)copper(I) tetrafluoroborate, [Cu(IPr)(Triaz)]BF_4 _(5).** In a glovebox, a microwave vial was charged with [Cu(Cl)(IPr)] (200.0 mg, 0.41 mmol), NaOH (66.0 mg, 4 equiv, 1.64 mmol), Triaz^.^HBF_4_ (190.0 mg, 1 equiv, 0.41 mmol) and acetonitrile (2 mL). The reaction mixture was stirred during 2 h at 80 °C in a microwave. The solution was concentrated and diethyl ether (10 mL) was added. The precipitate was collected by filtration and washed with diethyl ether (3 × 5 mL). The desired compound was obtained as a colourless solid (346 mg, 92%). ^1^H NMR (400 MHz, CD_2_Cl_2_, 298 K) δ (ppm) 0.77 (d, ^3^*J*_HH_ = 6.9 Hz, 6H, CHC*H*_3_ (Triaz)), 0.82 (d, ^3^*J*_HH_ = 6.9 Hz, 12H, CHC*H*_3_ (IPr)), 0.96 (d, ^3^*J*_HH_ = 6.9 Hz, 6H, CHC*H*_3_ (Triaz)), 1.01 (d, ^3^*J*_HH_ = 6.9 Hz, 12H, CHC*H*_3_ (IPr)), 1.44 (s, 9H, C(C*H*_3_)_3_) 1.99 (septet, ^3^*J*_HH_ = 6.9 Hz, 2H, C*H*CH_3_ (Triaz)), 2.36 (septet, ^3^*J*_HH_ = 6.9 Hz, 4H, C*H*CH_3_ (IPr)), 3.98 (s, 3H, C*H*_3_), 6.98 (d, ^3^*J*_HH_ = 8.3 Hz, 2H, C_Ar_*H* (Triaz)), 7.06 (s, 2H, *H*^4^ and *H*^5^), 7.10 (d, ^3^*J*_HH_ = 7.9 Hz, 2H, C_Ar_*H* (Triaz)), 7.19 (d, ^3^*J*_HH_ = 7.8 Hz, 4H, C_Ar_*H* (IPr)), 7.36 (d, ^3^*J*_HH_ = 8.4 Hz, 2H, C_Ar_*H* (Triaz)), 7.43 (t, *^3^J*_HH_ = 7.9 Hz, 1H, C_Ar_*H* (Triaz)), 7.50 (t, *^3^J*_HH_ = 7.8 Hz, 2H, C_Ar_*H* (IPr)); ^13^C{^1^H} NMR (75 MHz, CD_2_Cl_2_, 298 K) δ (ppm) 23.4 (s, CH*C*H_3_ (Triaz)), 24.8 (s, CH*C*H_3_ (IPr)), 24.4 (s, CH*C*H_3_ (IPr)), 24.7 (s, CH*C*H_3_ (Triaz)), 28.4 (s, *C*HCH_3_ (Triaz)), 28.6 (s, *C*HCH_3_ (IPr)), 31.3 (s, *C*CH_3_), 35.0 (s, *C*^IV^), 37.6 (s, *C*H_3_), 123.4 (s, *C*^IV^), 123.9 (s, *C*_Ar_H), 124.2 (s, *C*^4^ and *C*^5^), 124.4 (s, *C*_Ar_H), 126.6 (s, *C*_Ar_H), 129.4 (s, *C*_Ar_H), 130.5 (s, *C*_Ar_H), 130.9 (s, *C*_Ar_H), 134.3 (s, C^IV^), 144.8 (s, *C*^IV^), 145.2 (s, *C*^IV^), 152.0 (s, *C*^IV^), 152.8 (s, *C*^IV^), 179.4 (s, *C*_carbene_); ^19^F{^1^H} NMR (282 MHz, CDCl_3_, 298 K) δ (ppm) −155.0 (s, BF_4_), −155.1 (s, BF_4_); anal. calcd for C_52_H_69_BCuF_4_N_5_: C, 68.30; H, 7.61; N, 7.66; found: C, 68.15; H, 7.72; N, 7.68.

**Bis{1-{2,6-(diisopropyl)phenyl}-3-methyl-4-(4-*tert*-butylphenyl)-1,2,3-triazol-5-ylidene}copper(I) tetrafluoroborate, [Cu(Triaz)_2_]BF_4 _(6)_._** In a glovebox, a vial was charged with [Cu(Cl)(Triaz)] (150.0 mg, 0.32 mmol), NaOH (50 mg, 4 equiv, 1.28 mmol), Triaz.HBF_4_ (148 mg, 1 equiv, 0.32 mmol) and acetonitrile (2 mL). The reaction mixture was stirred during 2 h at 80 °C in a microwave. The solution was concentrated (0.5 mL) and diethyl ether (10 mL) was added. The precipitate was collected by filtration and washed with diethyl ether (3 × 5 mL). The desired compound was obtained as a colourless solid (281 mg, 97%). ^1^H NMR (400 MHz, CDCl_3_, 298 K, TMS) δ (ppm) 0.79 (d, ^3^*J*_HH_ = 6.8 Hz, 12H, CHC*H*_3_ (IPr)), 1.08 (d, ^3^*J*_HH_ = 6.8 Hz, 12H, CHC*H*_3_ (IPr)), 1.38 (s, 18H, C(C*H*_3_)_3_), 2.10 (septet, ^3^*J*_HH_ = 6.8 Hz, 4H, C*H*CH_3_ (IPr)), 4.20 (s, 6H, C*H*_3_), 7.19 (d, ^3^*J*_HH_ = 7.8 Hz, 4H, C_Ar_*H* (IPr)), 7.34 (m, 8H, C_Ar_*H*), 7.49 (t, *^3^J*_HH_ = 7.7 Hz, 2H, C_Ar_*H* (IPr)); ^13^C{^1^H} NMR (75 MHz, CDCl_3_, 298 K, TMS) δ (ppm) 23.8 (s, CH*C*H_3_ (Triaz)), 24.2 (s, CH*C*H_3_ (Triaz)), 28.4 (s, *C*HCH_3_ (Triaz)), 31.3 (s, C*C*H_3_), 35.0 (s, *C*^IV^), 37.8 (s, *C*H_3_), 123.6 (s, *C*^IV^), 124 (s, *C*_Ar_H), 126.2 (s, *C*_Ar_H), 129.0 (s, *C*_Ar_H), 131.1 (s, *C*_Ar_H), 134.3 (s, *C*^IV^), 145.0 (s, *C*^IV^), 149.2 (s, *C*^IV^), 153.4 (s, *C*^IV^); ^19^F{^1^H} NMR (282 MHz, CDCl_3_, 298 K) δ (ppm) −154.9 (s, BF_4_), −155.0 (s, BF_4_); anal. calcd for C_50_H_66_BCuF_4_N_6_: C, 66.62; H, 7.38; N, 9.32; found: C, 66.55; H, 7.46; N, 9.47.

**General catalytic procedure.** A vial was charged with [Cu(Triaz)_2_]BF_4_ (4.5 mg, 1 mol %), the alkyne (0.5 mmol), the azide (0.6 mmol) and the amine (0.6 mmol). The reaction was stirred neat for the appropriate amount of time. Dichloromethane (2 mL) and a saturated aqueous solution of ammonium chloride (3 mL) were added and the reaction mixture stirred during 30 minutes. The aqueous layer was extracted with dichloromethane (3 × 10 mL). The combined organic layers were dried over MgSO_4_, filtered and the solvent was removed under vacuum. The crude product was purified by flash column chromatography or by recrystallization. The reported yields are the average of two reactions.

## Supporting Information

File 1 Experimental and characterisation data.

File 2 Crystal data for **4**.

File 3 Crystal data for **5**.

File 4 Crystal data for **6**.
