# Supplementary material for: Aerobic synthesis of N-sulfonylamidines mediated by N-heterocyclic carbene copper(I) catalysts
Source: Beilstein J Org Chem. 2020 Mar 24;16:482–91. doi: 10.3762/bjoc.16.43 (PMC7113546; doi:10.3762/bjoc.16.43)
Supplement: File 1 — Experimental and characterisation data. [file Beilstein_J_Org_Chem-16-482-s001.pdf]

BEILSTEIN JOURNAL OF ORGANIC CHEMISTRY

Supporting Information

for

Aerobic synthesis of *N*-sulfonylamidines mediated by N-heterocyclic carbene copper(I) catalysts

Faïma Lazreg, Marie Vasseur, Alexandra M. Z. Slawin and Catherine S. J. Cazin

Beilstein J. Org. Chem. **2020,** *16,* 482–491. doi:10.3762/bjoc.16.43

Experimental and characterisation data

License and Terms: This is a supporting information file under the terms of the Creative Commons Attribution License (http://creativecommons.org/licenses/by/4.0). Please note that the reuse, redistribution and reproduction in particular requires that the authors and source are credited.
The license is subject to the *Beilstein Journal of Organic Chemistry* terms and conditions: (https://www.beilstein-journals.org/bjoc)

Contents

1. General Information S3

2. Sulfonyl azide substrates S3

3. Catalysis S5

3.1. General catalytic procedure S5

3.2. Optimisation S5

3.3. Sulfonamide compounds S7

4. Calculation of % V_{Bur} S27

4.1. [Cu(Pyr)(IPr)]OTf (**4**) S27

4.2. [Cu(IPr)(Triaz)]BF_4 (**5**) S28

4.3 [Cu(Triaz)$_2$]BF_4 (**6**) S31

5. Mechanistic studies S34

5.1. Stoichiometric reaction 1: [Cu(Triaz)$_2$]BF_4 and phenylacetylene S34

5.2. Synthesis of [Cu(Triaz)(C≡CPh)] S35

5.3. Stoichiometric reaction 2: tosyl azide and diisopropylamine S37

5.4. Stoichiometric reaction 3: [Cu(Triaz)$_2$]BF_4, tosyl azide and diisopropylamine S38

5.5. Stoichiometric reaction 4: Phenylacetylene and diisopropylamine S39

5.6. Stoichiometric reaction 5: [Cu(Triaz)$_2$]BF_4, phenylacetylene and diisopropylamine S40

5.7. Stoichiometric reaction 6: [Cu(Triaz)(C≡CPh)] and tosyl azide S41

5.8. Stoichiometric reaction 7: [Cu(Triaz)(C≡CPh)], tosyl azide and diisopropylamine S42

5.9. Stoichiometric reaction 8: [Cu(Triaz)(C≡CPh)], tosyl azide, diisopropylamine and $TriazHBF_4$ S43

5.10. Catalytic transformation using [Cu(Triaz)(C≡CPh)] S44

5.11. Stoichiometric benchmark reaction with $TriazHBF_4$ salt S45

5.12. Synthesis of the sulfonyl triazole, 1-(4-methylphenylsulfonyl)-4-phenyl-1*H*-1,2,3-triazole S46

5.13. Stoichiometric reaction 9: $[Cu(Triaz)_2]BF_4$ and 1-(4-methylphenylsulfonyl)-4-phenyl-1*H*-1,2,3-triazole S47

5.14. Stoichiometric reaction 10: 1-(4-methylphenylsulfonyl)-4-phenyl-1*H*-1,2,3-triazole and diisopropylamine S48

5.15. Stoichiometric reaction 11: $[Cu(Triaz)_2]BF_4$, 1-(4-methylphenylsulfonyl)-4-phenyl-1*H*-1,2,3-triazole and diisopropylamine S49

6. NMR Spectra S50

6.1. NMR Spectra of complexes S50

6.2. NMR Spectra of catalysis products S56

7. References S87

1. General Information

Solvents and reagents were used as received without further purification. Azides were synthesised according to reported procedures.[1] 4-Cyanosulfonyl chloride was synthesised following the described procedure.[2] Complexes were synthesised following the reported procedure except for the synthesis of [Cu(IPr)(Pyr)]OTf, [Cu(IPr)(Triaz)]BF_4 and [Cu$(Triaz)_2$]BF_4 complexes.[3] The complex syntheses were carried out in a CEM Discover microwave. ^{1}H, ^{13}C-{^{1}H} and ^{31}P-{^{1}H} Nuclear Magnetic Resonance (NMR) spectra were recorded 298K on a Bruker AVANCE 400 spectrometer using the residual solvent peak ($CDCl_3$: δ_H = 7.26 ppm, δ_C = 77.16 ppm) or TMS as reference. Elemental analyses were performed by the London Metropolitan University Service 166-220 Holloway Road, London, N7 8DB. Mass spectroscopy was performed by the EPSRC National Mass Spectrometry Service Centre at Swansea University, Grove Building, Singleton Park, Swansea, SA2 8PP, Wales.

2. Sulfonyl azide substrates[2]

Tosyl azide

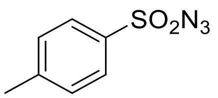

^{1}H NMR (300 MHz, $CDCl_3$, 298K, TMS): δ (ppm) = 2.48 (s, 3H, CH_3), 7.41 (d, 2H, $^3J_{HH}$ = 8.12 Hz, $C_{Ar}H$), 7.84 (d, 2H, $^3J_{HH}$ = 8.12 Hz, $C_{Ar}H$).

4-Methoxybenzenesulfonyl azide

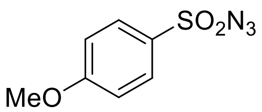

^{1}H NMR (300 MHz, $CDCl_3$, 298K, TMS): δ (ppm) = 7.06 (d, 2H, $^3J_{HH}$ = 9.14 Hz, $C_{Ar}H$), 7.90 (d, 2H, $^3J_{HH}$ = 9.14 Hz, $C_{Ar}H$).

Benzenesulfonyl azide

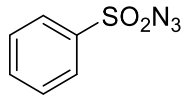

^{1}H NMR (400 MHz, CDCl$_3$, 298K, TMS): δ (ppm) = 7.63 (t, 2H, $^3J_{HH}$ = 7.7 Hz, C$_{Ar}$*H*), 7.74 (t, 1H, $^3J_{HH}$ = 7.7 Hz, C$_{Ar}$*H*), 7.97 (t, 2H, $^3J_{HH}$ = 8.4 Hz, C$_{Ar}$*H*).

^{1}H NMR (400 MHz, CDCl$_3$, 298K, TMS): δ (ppm) = 3.27 (s, 3H, C*H*$_3$).

Naphthalene-1-sulfonyl azide

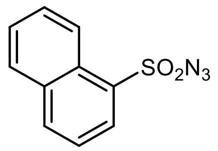

^{1}H NMR (400 MHz, CDCl$_3$, 298K, TMS): δ (ppm) = 7.55-7.64 (m, 1H, C$_{Ar}$*H*), 7.65-7.70 (m, 1H, C$_{Ar}$*H*), 7.72-7.79 (m, 1H, C$_{Ar}$*H*), 7.99 (dd, 1H, $^3J_{HH}$ = 8.3, 3.1 Hz, C$_{Ar}$*H*), 8.20 (dd, 2H, $^3J_{HH}$ = 8.3, 2.9 Hz, C$_{Ar}$*H*), 8.36 (dd, 1H, $^3J_{HH}$ = 7.3, 3.1 Hz, C$_{Ar}$*H*), 7.50 (dd, 1H, $^3J_{HH}$ = 8.6, 2.9 Hz, C$_{Ar}$*H*).

2,4,6-Triisopropylbenzenesulfonyl azide

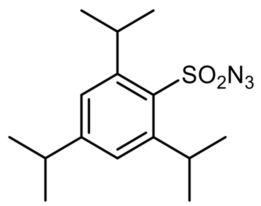

^{1}H NMR (400 MHz, CDCl$_3$, 298K, TMS): δ (ppm) = 1.27 (d, 6H, $^3J_{HH}$ = 6.8 Hz, CH(C*H*$_3$)$_2$) overlapped with 1.29 (d, 12H, $^3J_{HH}$ = 7.0 Hz CH(C*H*$_3$)$_2$), 2.93 (septet, 1H, $^3J_{HH}$ = 6.8 Hz, C*H*(CH$_3$)$_2$) 4.05 (septet, 2H, $^3J_{HH}$ = 7.0 Hz, C*H*(CH$_3$)$_2$), 3.82-4.07 (m, 2H, C$_{Ar}$*H*).

4-Bromobenzenesulfonyl azide

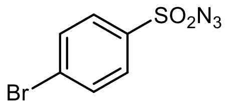

^{1}H NMR (400 MHz, $CDCl_3$, 298K, TMS): δ (ppm) = 7.74-7.80 (m, 2H, $C_{Ar}H$), 7.81-7.86 (m, 2H, $C_{Ar}H$).

3. Catalysis

3.1. General catalytic procedure

A vial was charged with $[Cu(Triaz)_2]BF_4$ (4.5 mg, 1 mol %), the alkyne (0.5 mmol), the azide (0.6 mmol), and the amine (0.6 mmol). The reaction was stirred neat for the appropriate amount of time. Dichloromethane (2 mL) and a saturated aqueous solution of ammonium chloride (3 mL) were added and the reaction mixture stirred during 30 minutes. The aqueous layer was extracted with dichloromethane (3 × 10 mL). The combined organic layers were dried over $MgSO_4$, filtered and the solvent was removed under vacuum. The crude product was purified by flash column chromatography or by recrystallization. The reported yields are the average of two reactions.

3.2. Optimisation

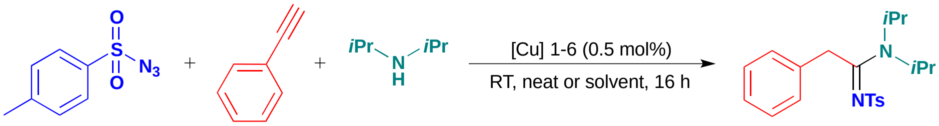

**Entry**	Catalyst	Solvent	Conversion (%)$^{a, b}$
**1**	$[Cu(ICy)_2]BF_4$ **1**	1,2-dichloromethane	24
**2**	$[Cu(ICy)_2]BF_4$ **1**	Water	30
**3**	$[Cu(ICy)_2]BF_4$ **1**	1,4-dioxane	47
**4**	$[Cu(ICy)_2]BF_4$ **1**	Ethanol	28

**5**	$[Cu(ICy)_2]BF_4$ **1**	Me-THF	37
**6**	$[Cu(ICy)_2]BF_4$ **1**	Acetonitrile	52
**7**	$[Cu(IPr)(ICy)]BF_4$ **2**	1,2-dichloromethane	25
**8**	$[Cu(IPr)(ICy)]BF_4$ **2**	Water	43
**9**	$[Cu(IPr)(ICy)]BF_4$ **2**	1,4-dioxane	45
**10**	$[Cu(IPr)(ICy)]BF_4$ **2**	Ethanol	25
**11**	$[Cu(IPr)(ICy)]BF_4$ **2**	Me-THF	35
**12**	$[Cu(IPr)(ICy)]BF_4$ **2**	Acetonitrile	32
**13**	$[Cu(IPr)(Triaz)]BF_4$ **5**	1,2-dichloromethane	17
**14**	$[Cu(IPr)(Triaz)]BF_4$ **5**	Water	43
**15**	$[Cu(IPr)(Triaz)]BF_4$ **5**	1,4-dioxane	44
**16**	$[Cu(IPr)(Triaz)]BF_4$ **5**	Acetonitrile	22
**17**	$[Cu(IPr)(Triaz)]BF_4$ **5**	Me-THF	32
**18**	$[Cu(IPr)(Triaz)]BF_4$ **5**	Ethanol	20
**19**	$[Cu(Triaz)_2]BF_4$ **6**	Ethanol	36
**20**	$[Cu(Triaz)_2]BF_4$ **6**	Me-THF	48
**21**	$[Cu(Triaz)_2]BF_4$ **6**	Acetonitrile	58

[a] Reaction conditions: phenylacetylene (0.5 mmol), tosyl azide (0.6 mmol), diisopropylamine (0.6 mmol), [Cu] (0.5 mol%), solvent (1 mL), 16 hours. [b] Conversion was determined by GC analysis based on phenylacetylene using mesitylene (42 μL) as internal standard.

3.3. Sulfonamide compounds

***N,N*-Diisopropyl-2-phenyl-*N'*-tosylacetimidamide[4] (10a)**

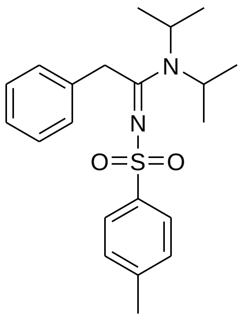

The general procedure yielded, after column chromatography (pentane/dichloromethane: 7/3), the title compound as a colorless solid (178.9 mg, 96%).

^{1}H NMR (400 MHz, CDCl$_3$, 298K, TMS): δ (ppm) = 0.87 (d, 6H, $^3J_{HH}$ = 6.6 Hz, CH(C*H*$_3$)$_2$), 1.39 (d, 6H, $^3J_{HH}$ = 6.6 Hz, CH(C*H*$_3$)$_2$), 2.39 (s, 3H, Ph-C*H*$_3$), 3.44 (m, 1H, C*H*(CH$_3$)$_2$), 4.00 (septet, 1H, $^3J_{HH}$ = 6.6 Hz, C*H*(CH$_3$)$_2$), 4.41 (s, 2H, Ph-C*H*$_2$), 7.19-7.30 (m, 7H, C$_{Ar}$*H*), 7.83 (d, 2H, $^3J_{HH}$ = 8.3 Hz, C$_{Ar}$*H*).

^{13}C-{^{1}H} NMR (100 MHz, CDCl$_3$, 298K, TMS): δ (ppm) = 19.8 (s, CH(*C*H$_3$)$_2$), 21.4 (s, Ph-*C*H$_3$), 38.8 (s, Ph-*C*H$_2$), 48.0 (s, *C*H(CH$_3$)$_2$), 50.4 (s, *C*H(CH$_3$)$_2$), 126.2 (s, *C*$_{Ar}$H), 126.7 (s, *C*$_{Ar}$H), 128.0 (s, *C*$_{Ar}$H), 128.8 (s, *C*$_{Ar}$H), 129.0 (s, *C*$_{Ar}$H), 134.9 (s, *C*^{IV}), 141.5 (s, *C*^{IV}), 141.6 (s, *C*^{IV}), 163.4 (s, *C*^{IV} *C*=N).

***N,N*-Diisopropyl-2-phenyl-*N'*-(phenylsulfonyl)acetimidamide[5] (10b)**

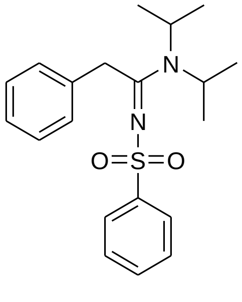

The general procedure yielded, after column chromatography (pentane/ethyl acetate: 7/3), the title compound as a colorless solid (130.7 mg, 73%).

^{1}H NMR (400 MHz, CDCl$_3$, 298K, TMS): δ (ppm) = 0.88 (d, 6H, $^3J_{HH}$ = 6.6 Hz, CH(C*H*$_3$)$_2$), 1.38 (d, 6H, $^3J_{HH}$ = 6.8 Hz, CH(C*H*$_3$)$_2$), 3.45 (m, 1H, C*H*(CH$_3$)$_2$), 4.01 (septet, 1H, $^3J_{HH}$ = 6.6 Hz, C*H*(CH$_3$)$_2$), 4.42 (s, 2H, Ph-C*H*$_2$), 7.19-7.23 (m, 3H, C$_{Ar}$*H*), 7.27-7.31 (m, 2H, C$_{Ar}$*H*), 7.42-7.49 (m, 3H, C$_{Ar}$*H*), 7.95 (m, 2H, C$_{Ar}$*H*).

^{13}C-{^{1}H} NMR (100 MHz, CDCl$_3$, 298K, TMS): δ (ppm) = 19.8 (s, CH(*C*H$_3$)$_2$), 38.9 (s, Ph-*C*H$_2$), 48.1 (s, *C*H(CH$_3$)$_2$), 50.5 (s, *C*H(CH$_3$)$_2$), 126.2 (s, *C*$_{Ar}$H), 126.8 (s, *C*$_{Ar}$H), 128.0 (s, *C*$_{Ar}$H), 128.4 (s, *C*$_{Ar}$H), 128.9 (s, *C*$_{Ar}$H), 131.2 (s, *C*$_{Ar}$H), 134.9 (s, *C*^{IV}), 144.3 (s, *C*^{IV}), 163.5 (s, *C*^{IV} *C*=N).

***N,N*-Diisopropyl-*N'*-((4-methoxyphenyl)sulfonyl)-2-phenylacetimidamide (10c)**

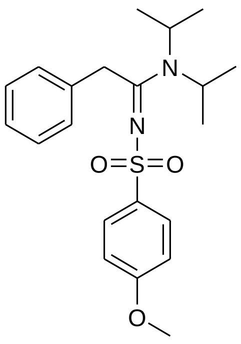

The general procedure yielded, after recrystallization (pentane/ethyl acetate: 8/2), the title compound as a colorless solid (184.4 mg, 95%).

^{1}H NMR (400 MHz, CDCl$_3$, 298K, TMS): δ (ppm) = 0.87 (d, 6H, $^3J_{HH}$ = 6.7 Hz, CH(C*H*$_3$)$_2$), 1.40 (d, 6H, $^3J_{HH}$ = 6.7 Hz, CH(C*H*$_3$)$_2$), 3.44 (m, 1H, C*H*(CH$_3$)$_2$), 3.84 (s, 3H, OC*H*$_3$), 4.00 (septet, 1H, $^3J_{HH}$ = 6.7 Hz, C*H*(CH$_3$)$_2$), 4.40 (s, 2H, Ph-C*H*$_2$), 6.91 (d, 2H, $^3J_{HH}$ = 8.9 Hz, C$_{Ar}$*H*), 7.18-7.22 (m, 4H, C$_{Ar}$*H*), 7.28-7.30 (m, 1H, C$_{Ar}$*H*), 7.87 (d, 2H, $^3J_{HH}$ = 8.9 Hz, C$_{Ar}$*H*).

^{13}C-{^{1}H} NMR (100 MHz, CDCl$_3$, 298K, TMS): δ (ppm) = 19.8 (s, CH(*C*H$_3$)$_2$), 38.6 (s, O*C*H$_3$), 48.0 (s, Ph-*C*H$_2$), 50.4 (s, *C*H(CH$_3$)$_2$), 55.5 (s, *C*H(CH$_3$)$_2$), 113.5 (s, *C*$_{Ar}$H), 126.7 (s, *C*$_{Ar}$H), 128.0 (s, *C*$_{Ar}$H), 128.2 (s, *C*$_{Ar}$H), 128.8 (s, *C*$_{Ar}$H), 135.0 (s, *C*^{IV}), 136.6 (s, *C*^{IV}), 161.7 (s, *C*^{IV}), 163.3 (s, *C*^{IV} *C*=N).

HRMS (EI) *m/z* for $C_{21}H_{28}N_2O_3S$: theoretical value: 389.1892, observed data: 389.1890.

***N'*-((4-Bromophenyl)sulfonyl)-*N,N*-diisopropyl-2-phenylacetimidamide (10d)**

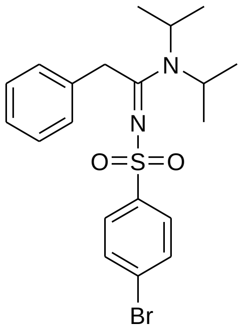

The general procedure yielded, after column chromatography (pentane/dichloromethane: 7/3), to the title compound as a colorless solid (148.3 mg, 68%).

^{1}H NMR (400 MHz, $CDCl_3$, 298K, TMS): δ (ppm) = 0.89 (d, 6H, $^3J_{HH}$ = 6.6 Hz, CH(C*H*$_3$)$_2$), 1.38 (d, 6H, $^3J_{HH}$ = 6.8 Hz, CH(C*H*$_3$)$_2$), 3.47 (m, 1H, C*H*(CH$_3$)$_2$), 4.01 (septet, 1H, $^3J_{HH}$ = 6.8 Hz C*H*(CH$_3$)$_2$), 4.40 (s, 2H, Ph-C*H$_2$*), 7.17 (d, 2H, $^3J_{HH}$ = 7.5 Hz, C_{Ar}*H*), 7.24 (d, 1H, $^3J_{HH}$ = 7.3 Hz, C_{Ar}*H*), 7.28-7.31 (m, 2H, C_{Ar}*H*), 7.56 (d, 2H, $^3J_{HH}$ = 8.6 Hz, C_{Ar}*H*), 7.80 (d, 2H, $^3J_{HH}$ = 8.6 Hz, C_{Ar}*H*).

^{13}C-{^{1}H} NMR (100 MHz, $CDCl_3$, 298K, TMS): δ (ppm) = 19.8 (s, CH(*C*H$_3$)$_2$), 38.9 (s, *C*H$_2$), 48.2 (s, *C*H(CH$_3$)$_2$), 50.6 (s, *C*H(CH$_3$)$_2$), 125.9 (s, C^{IV}), 126.9 (s, *C*$_{Ar}$H), 127.9 (s, *C*$_{Ar}$H), 128.9 (s, *C*$_{Ar}$H), 131.7 (s, *C*$_{Ar}$H), 134.6 (s, C^{IV}), 143.3 (s, C^{IV}), 163.6 (s, C^{IV} *C*=N).

HRMS (EI) *m/z* for $C_{20}H_{25}BrN_2O_2S$: theoretical value: 439.0874, observed data: 439.0864.

***N'*-((4-Cyanophenyl)sulfonyl)-*N,N*-diisopropyl-2-phenylacetimidamide (10e)**

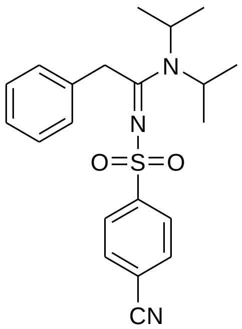

The general procedure yielded, after recrystallization (pentane/dichloromethane: 9/1), to the title compound as a colorless solid (130 mg, 68%).

^{1}H NMR (400 MHz, CDCl$_3$, 298K, TMS): δ (ppm) = 0.92 (d, 6H, $^{3}J_{HH}$ = 6.6 Hz, CH(C*H*$_3$)$_2$), 1.36 (d, 6H, $^{3}J_{HH}$ = 6.8 Hz, CH(C*H*$_3$)$_2$), 3.50 (m, 1H, C*H*(CH$_3$)$_2$), 4.02 (septet, 1H, $^{3}J_{HH}$ = 6.6 Hz, C*H*(CH$_3$)$_2$), 4.41 (s, 2H, Ph-C*H*$_2$), 7.17 (d, 2H, $^{3}J_{HH}$ = 7.5 Hz, C$_{Ar}$*H*), 7.22-7.32 (m, 3H, C$_{Ar}$*H*), 7.73 (d, 2H, $^{3}J_{HH}$ = 8.5 Hz, C$_{Ar}$*H*), 8.03 (d, 2H, $^{3}J_{HH}$ = 8.5 Hz, C$_{Ar}$*H*).

^{13}C-{^{1}H} NMR (100 MHz, CDCl$_3$, 298K, TMS): δ (ppm) = 19.8 (s, CH(*C*H$_3$)$_2$), 39.0 (s, *C*H$_2$), 48.3 (s, *C*H(CH$_3$)$_2$), 50.9 (s, *C*H(CH$_3$)$_2$), 114.9 (s, C^{IV}), 117.9 (s, *C*^{IV}), 126.9 (s, *C*$_{Ar}$H), 127.1 (s, *C*$_{Ar}$H), 127.8 (s, *C*$_{Ar}$H), 129.0 (s, *C*$_{Ar}$H), 132.4 (s, *C*$_{Ar}$H), 134.3 (s, C^{IV}), 148.1 (s, C^{IV}), 163.9 (s, C^{IV} *C*=N).

HRMS (EI) *m/z* for $C_{21}H_{25}N_3O_2S$: theoretical value: 384.1740, observed data: 384.1739.

N,N-Diisopropyl-2-phenyl-*N'*-((2,4,6-triisopropylphenyl)sulfonyl)acetimidamide (10f)

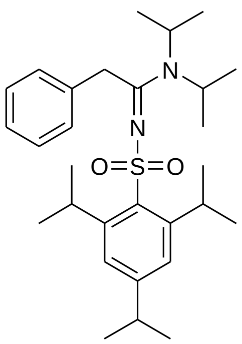

The general procedure yielded, after column chromatography (pentane/dichloromethane: 6/4), the title compound as a colorless solid (159.5 mg, 66%).

^{1}H NMR (400 MHz, CDCl$_3$, 298K, TMS): δ (ppm) = 0.88 (d, 6H, $^{3}J_{HH}$ = 6.6 Hz, CH(C*H*$_3$)$_2$), 1.23-1.26 (m, 18H, CH(C*H*$_3$)$_2$), 1.42 (d, 6H, $^{3}J_{HH}$ = 6.8 Hz, CH(C*H*$_3$)$_2$), 2.87 (septet, 1H, $^{3}J_{HH}$ = 6.8 Hz, C*H*(CH$_3$)$_2$), 3.45 (m, 1H, C*H*(CH$_3$)$_2$), 3.99 (septet, 1H, $^{3}J_{HH}$ = 6.6 Hz, C*H*(CH$_3$)$_2$), 4.30-4.40 (m, 4H, C*H*(CH$_3$)$_2$) overlapped with 4.37 (s, Ph-CH$_2$), 7.08 (s, 2H, C$_{Ar}$*H*), 7.16-7.23 (m, 3H, C$_{Ar}$*H*), 7.27-7.31 (m, 2H, C$_{Ar}$*H*).

^{13}C-{^{1}H} NMR (100 MHz, CDCl$_3$, 298K, TMS): δ (ppm) = 20.0 (s, CH(*C*H$_3$)$_2$), 20.2 (s, CH(*C*H$_3$)$_2$), 23.9 (s, CH(*C*H$_3$)$_2$), 25.1 (s, CH(*C*H$_3$)$_2$), 29.9 (s, *C*H(CH$_3$)$_2$), 34.2 (s, CH(*C*H$_3$)$_2$), 39.2 (s, *C*H$_2$), 48.1 (s, *C*H(CH$_3$)$_2$), 50.5 (s, *C*H(CH$_3$)$_2$), 123.2 (s, *C*$_{Ar}$H), 126.9 (s, *C*$_{Ar}$H), 128.2 (s,

C_{Ar}H), 128.9 (s, C_{Ar}H), 135.4 (s, C^{IV}), 138.0 (s, C^{IV}), 149.1 (s, C^{IV}), 151.1 (s, C^{IV}), 163.5 (s, C^{IV} C=N).

HRMS (EI) *m/z* for $C_{29}H_{44}N_2O_2S$, theoretical value: 485.3196, observed data: 485.3183.

N,N-Diisopropyl-*N'*-(naphthalen-1-ylsulfonyl)-2-phenylacetimidamide (10g)

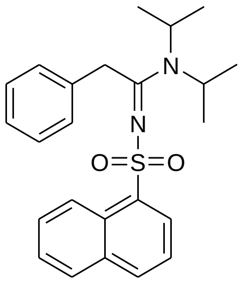

The general procedure yielded, after recrystallization (pentane/dichloromethane: 8/2), the title compound as colorless solid (189.8 mg, 93%).

^{1}H NMR (400 MHz, $CDCl_3$, 298K, TMS): δ (ppm) = 0.86 (d, 6H, $^3J_{HH}$ = 6.8 Hz, CH(CH_3)$_2$), 1.34 (d, 6H, $^3J_{HH}$ = 6.8 Hz, CH(CH_3)$_2$), 3.43 (m, 1H, CH(CH$_3$)$_2$, 3.95 (septet, 1H, $^3J_{HH}$ = 6.8 Hz, CH(CH$_3$)$_2$), 4.41 (s, 2H, Ph-CH_2), 6.98-7.00 (m, 2H, $C_{Ar}H$), 7.13-7.15 (m, 3H, $C_{Ar}H$), 7.37 (t, 1H, $^3J_{HH}$ = 7.8 Hz, $C_{Ar}H$), 7.55 (t, 1H, $^3J_{HH}$ = 7.6 Hz, $C_{Ar}H$), 7.63 (t, 1H, $^3J_{HH}$ = 7.8 Hz, $C_{Ar}H$), 7.88 (t, 2H, $^3J_{HH}$ = 8.6 Hz, $C_{Ar}H$), 8.24 (d, 1H, $^3J_{HH}$ = 7.3 Hz, $C_{Ar}H$), 8.94 (d, 1H, $^3J_{HH}$ = 8.6 Hz, $C_{Ar}H$).

^{13}C-{^{1}H} NMR (100 MHz, $CDCl_3$, 298K, TMS): δ (ppm) = 19.8 (s, CH(CH$_3$)$_2$), 20.0 (s, CH(CH$_3$)$_2$), 38.4 (s, Ph-CH$_2$), 48.1 (s, CH(CH$_3$)$_2$), 50.6 (s, CH(CH$_3$)$_2$), 124.1 (s, C_{Ar}H), 126.3 (s, C_{Ar}H), 126.7 (t, C_{Ar}H), 127.1 (s, C_{Ar}H), 127.6 (s, C_{Ar}H), 128.3 (s, C^{IV}), 128.7 (s, C_{Ar}H), 132.6 (s, C_{Ar}H), 134.1 (s, C^{IV}), 134.6 (s, C^{IV}), 139.3 (s, C^{IV}), 164.1 (s, C^{IV} C=N).

HRMS (EI) *m/z* for $C_{24}H_{28}N_2O_2S$: theoretical value: 409.1944, observed data: 409.1939.

2-(4-Fluorophenyl)-*N,N*-diisopropyl-*N'*-tosylacetimidamide[7] (11a)

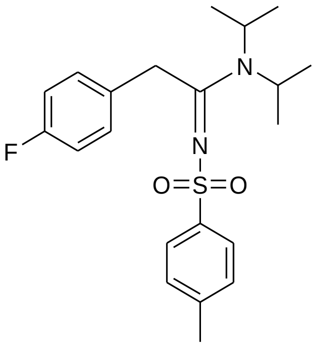

The general procedure yielded, after column chromatography (pentane/dichloromethane: 7/3), the title compound as a colorless solid (280.9 mg, 72%).

^{1}H NMR (400 MHz, $CDCl_3$, 298K, TMS): δ (ppm) = 0.90 (d, 6H, $^3J_{HH}$ = 6.6 Hz, $CH(CH_3)_2$), 1.38 (d, 6H, $^3J_{HH}$ = 6.8 Hz, $CH(CH_3)_2$), 2.40 (s, 3H, Ar CH_3), 3.45 (b. septet, 1H, $CH(CH_3)_2$), 3.97 (septet, 1H, $^3J_{HH}$ = 6.6 Hz, $CH(CH_3)_2$), 4.37 (s, 2H, Ph-CH_2), 6.99 (t, 2H, $^3J_{HH}$ = 8.2 Hz, $C_{Ar}H$), 7.18-7.25 (m, 4H, $C_{Ar}H$), 7.82 (d, 2H, $^3J_{HH}$ = 8.2 Hz, $C_{Ar}H$).

^{13}C-{^{1}H} NMR (100 MHz, $CDCl_3$, 298K, TMS): δ (ppm) = 19.8 (s, $CH(CH_3)_2$), 19.9 (s, $CH(CH_3)_2$), 21.4 (s, Ar-CH_3), 21.4 (s, Ar-CH_3), 38.0 (s, Ph-CH_2), 48.1 (s, $CH(CH_3)_2$), 50.4 (s, $CH(CH_3)_2$), 115.8 (s, C_{Ar}H), 115.6 (s, C_{Ar}H), 126.2 (s, C_{Ar}H), 129.1 (s, C_{Ar}H), 129.6 (d, C_{Ar}H), 141.4 (s, C^{IV}), 141.7 (s, C^{IV}), 160.5 (s, C^{IV}), 162.9 (s, C^{IV}), 163.1(s, C^{IV}, *C*=N). **^{19}F NMR (282 MHz, $CDCl_3$)** δ (ppm) -115.8

***N,N*-Diisopropyl-2-(*p*-tolyl)-*N'*-tosylacetimidamide[4] (11b)**

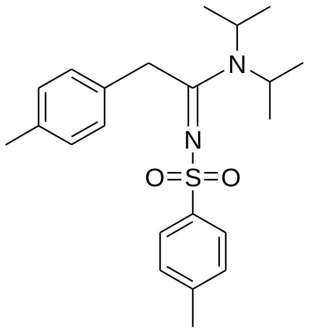

The general procedure yielded, after recrystallization (diethyl ether/dichloromethane: 8/2), the title compound as a colorless solid (251.0 mg, 65%).

^{1}H NMR (400 MHz, $CDCl_3$, 298K, TMS): δ (ppm) = 0.88 (d, 6H, $^{3}J_{HH}$ = 6.6 Hz, CH(CH_3)$_2$), 1.39 (d, 6H, $^{3}J_{HH}$ = 6.8 Hz, CH(CH_3)$_2$), 2.30 (s, 3H, Ph-CH_3), 2.39 (s, 3H, Ph-CH_3), 3.44 (m, 1H, C*H*(CH$_3$)$_2$), 4.01 (septet, 1H, $^{3}J_{HH}$ = 6.6 Hz, C*H*(CH$_3$)$_2$), 4.35 (s, 2H, Ph-CH_2), 7.07 (br. s, 4H, $C_{Ar}H$), 7.22 (d, 2H, $^{3}J_{HH}$ = 7.8 Hz, $C_{Ar}H$), 7.82 (d, 2H, $^{3}J_{HH}$ = 7.8 Hz, $C_{Ar}H$).

^{13}C-{^{1}H} NMR (100 MHz, $CDCl_3$, 298K, TMS): δ (ppm) = 19.8 (s, CH(*C*H$_3$)$_2$), 19.9 (s, CH(*C*H$_3$)$_2$), 21.0 (s, Ar-*C*H$_3$), 21.4 (s, Ar-*C*H$_3$), 38.4 (s, Ph-*C*H$_2$), 48.0 (s, *C*H(CH$_3$)$_2$), 50.3 (s, *C*H(CH$_3$)$_2$), 126.2 (s, C_{Ar}H), 127.8 (s, C_{Ar}H), 129.0 (s, C_{Ar}H), 129.5 (s, C_{Ar}H), 131.8 (s, C^{IV}), 136.3 (s, C^{IV}), 141.5 (s, C^{IV}), 141.6 (s, C^{IV}), 163.7(s, C^{IV} *C*=N).

***N,N*-Diisopropyl-2-(4-methoxyphenyl)-*N'*-tosylacetimidamide[7] (11c)**

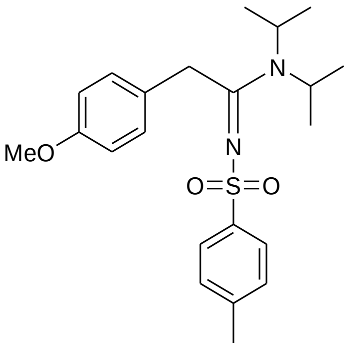

The general procedure yielded, after column chromatography (pentane/dichloromethane: 6/4), the title compound as a colorless solid (301.6 mg, 75%).

^{1}H NMR (400 MHz, $CDCl_3$, 298K, TMS): δ (ppm) = 0.89 (d, 6H, $^{3}J_{HH}$ = 6.6 Hz, CH(CH_3)$_2$), 1.38 (d, 6H, $^{3}J_{HH}$ = 6.8 Hz, CH(CH_3)$_2$), 2.39 (s, 3H, Ar-CH_3), 3.44 (m, 1H, C*H*(CH$_3$)$_2$), 3.78 (s, 3H, OCH_3), 4.00 (septet, 1H, $^{3}J_{HH}$ = 6.6 Hz, C*H*(CH$_3$)$_2$), 4.32 (s, 2H, Ph-CH_2), 6.82 (d, 2H, $^{3}J_{HH}$ = 8.7 Hz, $C_{Ar}H$), 7.12 (d, 2H, $^{3}J_{HH}$ = 8.6 Hz, $C_{Ar}H$), 7.23 (d, 2H, $^{3}J_{HH}$ = 8.6 Hz, $C_{Ar}H$), 7.83 (d, 2H, $^{3}J_{HH}$ = 8.7 Hz, $C_{Ar}H$).

^{13}C-{^{1}H} NMR (100 MHz, $CDCl_3$, 298K, TMS): δ (ppm) = 19.8 (s, CH(*C*H$_3$)$_2$), 19.9 (s, CH(*C*H$_3$)$_2$), 21.4 (s, Ar-*C*H$_3$), 38.0 (s, O*C*H$_3$), 48.0 (s, Ph-*C*H$_2$), 50.3 (s, *C*H(CH$_3$)$_2$), 55.3 (s, *C*H(CH$_3$)$_2$), 114.2 (s, C_{Ar}H), 126.2 (s, C_{Ar}H), 126.9 (s, C^{IV}), 129.0 (s, C_{Ar}H), 129.1 (s, C_{Ar}H), 141.5 (s, C^{IV}), 141.6 (s, C^{IV}), 158.4 (s, C^{IV}), 163.8 (s, C^{IV} *C*=N).

2-(4-(*tert*-Butyl)phenyl)-*N,N*-diisopropyl-*N'*-tosylacetimidamide[7] (11d)

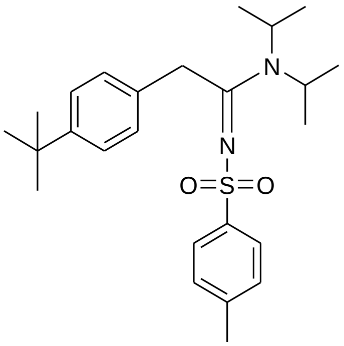

The general procedure yielded, after column chromatography (pentane/ethyl acetate: 8/2), the title compound as a colorless solid (308.3 mg, 72%).

^{1}H NMR (400 MHz, $CDCl_3$, 298K, TMS): δ (ppm) = 0.87 (d, 6H, $^3J_{HH}$ = 6.6 Hz, CH(CH_3)$_2$), 1.28 (s, 9H, C(CH_3)$_3$), 1.40 (d, 6H, $^3J_{HH}$ = 6.8 Hz, CH(CH_3)$_2$), 2.37 (s, 3H, Ar-CH_3), 3.45 (m, 1H, C*H*(CH$_3$)$_2$), 4.00 (septet, 1H, $^3J_{HH}$ = 6.6 Hz, C*H*(CH$_3$)$_2$), 4.35 (s, 2H, Ph-CH_2), 7.09 (d, 2H, $^3J_{HH}$ = 8.2 Hz, $C_{Ar}H$), 7.21 (d, 2H, $^3J_{HH}$ = 8.2 Hz, $C_{Ar}H$), 7.28 (d, 2H, $^3J_{HH}$ = 8.2 Hz, $C_{Ar}H$), 7.81 (d, 2H, $^3J_{HH}$ = 8.2 Hz, $C_{Ar}H$).

^{13}C-{^{1}H} NMR (100 MHz, $CDCl_3$, 298K, TMS): δ (ppm) = 19.8 (s, CH(*C*H$_3$)$_2$), 19.9 (s, CH(*C*H$_3$)$_2$), 21.4 (s, Ar-*C*H$_3$), 31.3 (s, C(*C*H)$_3$), 34.4 (s, *C*(CH)$_3$), 38.1 (s, Ph-*C*H$_2$), 48.0 (s, *C*H(CH$_3$)$_2$), 50.4 (s, *C*H(CH$_3$)$_2$), 125.6 (s, C_{Ar}H), 126.2 (s, C_{Ar}H), 127.6 (s, C_{Ar}H), 129.0 (s, C_{Ar}H), 131.7 (s, C^{IV}), 141.5 (s, C^{IV}), 141.6 (s, C^{IV}), 149.6 (s, C^{IV}), 163.8 (s, C^{IV}).

2-(3-Fluorophenyl)-*N,N*-diisopropyl-*N'*-tosylacetimidamide (11e)

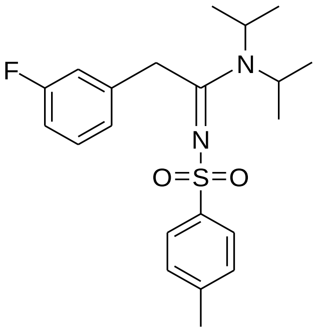

The general procedure yielded, after column chromatography (pentane/dichloromethane: 7/3), the title compound as a colorless solid (280.9 mg, 72%).

^{1}H NMR (400 MHz, $CDCl_3$, 298K, TMS): δ (ppm) = 0.92 (d, 6H, $^3J_{HH}$ = 6.6 Hz, CH(CH_3)$_2$), 1.39 (d, 6H, $^3J_{HH}$ = 6.8 Hz, CH(CH_3)$_2$), 2.39 (s, 3H, Ar-CH_3), 3.47 (m, 1H, CH(CH_3)$_2$), 3.95 (septet, 1H, $^3J_{HH}$ = 6.6 Hz, CH(CH_3)$_2$), 4.41 (s, 2H, Ph-CH_2), 6.89-6.94 (m, 2H, $C_{Ar}H$), 7.0 (d, 1H, $^3J_{HH}$ = 7.4 Hz, $C_{Ar}H$), 7.22-7.29 (m, 3H, $C_{Ar}H$), 7.82 (d, 2H, $^3J_{HH}$ = 8.2 Hz, $C_{Ar}H$).

^{13}C-{^{1}H} NMR (100 MHz, $CDCl_3$, 298K, TMS): δ (ppm) = 19.8 (s, CH(CH_3)$_2$), 19.9 (s, CH(CH_3)$_2$), 21.4 (s, Ar-CH_3), 38.31 (s, CH_2), 48.1 (s, CH(CH_3)$_2$), 50.5 (s, CH(CH_3)$_2$), 113.8 (d, C_{Ar}H), 115.0 (d, C_{Ar}H), 123.7 (s, C_{Ar}H), 126.2 (s, C^{IV}), 129.1 (s, C_{Ar}H), 130.3 (s, C^{IV}), 137.3 (s, C^{IV}-F), 141.5 (d, C^{IV}), 161.8 (s, C^{IV}), 162.6 (s, C^{IV}), 164.2 (s, C^{IV} C=N). **^{19}F NMR (377 MHz, $CDCl_3$):** δ (ppm) -112.4.

HRMS (EI) *m/z* for $C_{21}H_{27}FN_2O_2S$: theoretical value: 391.1850, observed data: 391.1847.

N,N-Diisopropyl-2-(*m*-tolyl)-*N'*-tosylacetimidamide (11f)

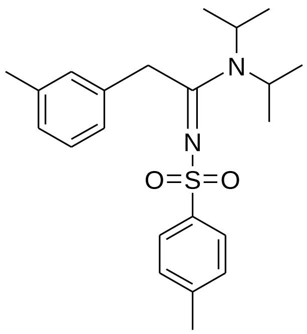

The general procedure yielded, after recrystallization (pentane/dichloromethane: 8/2), the title compound as a colorless solid (301.2 mg, 78%).

^{1}H NMR (400 MHz, $CDCl_3$, 298K, TMS): δ (ppm) = 0.88 (d, 6H, $^3J_{HH}$ = 6.6 Hz, CH(CH_3)$_2$), 1.41 (d, 6H, $^3J_{HH}$ = 6.8 Hz, CH(CH_3)$_2$), 2.28 (s, 3H, Ar-CH_3), 2.38 (s, 3H, Ar-CH_3), 3.45 (m, 1H, CH(CH_3)$_2$), 3.99 (septet, 1H, $^3J_{HH}$ = 6.6 Hz, CH(CH_3)$_2$), 4.36 (s, 2H, Ph-CH_2), 6.90 (s, 1H, $C_{Ar}H$), 6.96 (d, 1H, $^3J_{HH}$ = 7.7 Hz, $C_{Ar}H$), 7.00 (d, 1H, $^3J_{HH}$ = 7.7 Hz, $C_{Ar}H$), 7.15 (t, 1H, $^3J_{HH}$ = 7.7 Hz, $C_{Ar}H$), 7.21 (d, 2H, $^3J_{HH}$ = 8.3 Hz, $C_{Ar}H$), 7.81 (d, 2H, $^3J_{HH}$ = 8.3 Hz, $C_{Ar}H$).

^{13}C-{^{1}H} NMR (100 MHz, $CDCl_3$, 298K, TMS): δ (ppm) = 19.8 (s, CH(CH_3)$_2$), 19.9 (s, CH(CH_3)$_2$), 21.35 (Ar-CH_3), 21.4 (Ar-CH_3), 38.3 (s, Ph-CH_2), 48.0 (s, CH(CH_3)$_2$), 50.4 (s, CH(CH_3)$_2$), 124.9 (s, C_{Ar}H), 126.3 (s, C_{Ar}H), 127.4 (s, C_{Ar}H), 128.6 (s, C_{Ar}H), 128.7 (s, C_{Ar}H), 129.0 (s, C_{Ar}H), 134.7 (s, C^{IV}), 138.3 (s, C^{IV}), 141.5 (s, 2 C^{IV}), 163.6 (s, C^{IV} C=N).

HRMS (EI) *m/z* for $C_{22}H_{30}N_2O_2S$: theoretical value: 387.2101, observed data: 387.2100.

***N,N*-Diisopropyl-2-(2-methoxyphenyl)-*N'*-tosylacetimidamide (11g)**

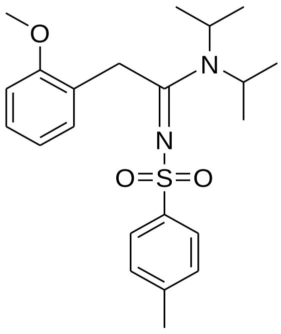

The general procedure yielded, after recrystallization (pentane/ethyl acetate: 8/2), the title compound as a colorless solid (329.8 mg, 82%).

^{1}H NMR (400 MHz, CDCl$_3$, 298K, TMS): δ (ppm) = 0.89 (d, 6H, $^3J_{HH}$ = 6.6 Hz, CH(C*H*$_3$)$_2$), 1.41 (d, 6H, $^3J_{HH}$ = 6.8 Hz, CH(C*H*$_3$)$_2$), 2.37 (s, 3H, Ar-C*H*$_3$), 3.46 (m, 1H, C*H*(CH$_3$)$_2$), 3.80-3.90 (m, 2H, C*H*(CH$_3$)$_2$) overlapped with 3.83 (s, 3H, OC*H*$_3$), 4.32 (s, 2H, Ph-C*H*$_2$), 6.83-6.86 (m, 2H, C$_{Ar}$*H*), 7.08 (d, 1H, $^3J_{HH}$ = 7.4 Hz, C$_{Ar}$*H*), 7.17-7.20 (m, 3H, C$_{Ar}$*H*), 7.81 (d, 2H, $^3J_{HH}$ = 8.2 Hz, C$_{Ar}$*H*).

^{13}C-{^{1}H} NMR (100 MHz, CDCl$_3$, 298K, TMS): δ (ppm) = 19.9 (s, 2 CH(*C*H$_3$)$_2$), 21.4 (s, Ar-*C*H$_3$), 31.9 (s, O*C*H$_3$), 47.9 (s, Ph-*C*H$_2$), 50.1 (s, *C*H(CH$_3$)$_2$), 55.5 (s, *C*H(CH$_3$)$_2$), 110.3 (s, *C*$_{Ar}$H), 120.9 (s, *C*$_{Ar}$H), 123.3 (s, C^{IV}), 126.2 (s, *C*$_{Ar}$H), 128.0 (s, *C*$_{Ar}$H), 128.5 (s, *C*$_{Ar}$H), 128.9 (s, *C*$_{Ar}$H), 141.4 (s, C^{IV}), 141.6 (s, C^{IV}), 156.0 (s, C^{IV}), 164.7 (s, C^{IV} *C*=N).

HRMS (EI) *m/z* for $C_{22}H_{30}N_2O_3S$: theoretical value: 403.2050, observed data: 403.2042.

2-(3,5-Bis(trifluoromethyl)phenyl)-*N,N*-diisopropyl-*N'*-tosylacetimidamide (11h)

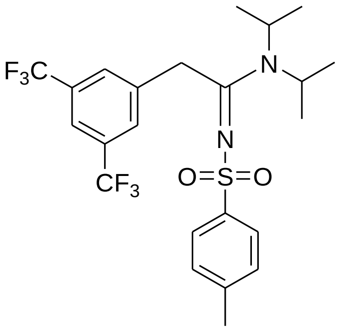

The general procedure yielded, after recrystallization (diethyl ether/ethyl acetate: 6/4), the title compound as a colorless solid (365.9 mg, 72%).

^{1}H NMR (400 MHz, CDCl$_3$, 298K, TMS): δ (ppm) = 0.96 (d, 6H, $^3J_{HH}$ = 6.6 Hz, CH(C*H*$_3$)$_2$), 1.43 (d, 6H, $^3J_{HH}$ = 6.8 Hz CH(C*H*$_3$)$_2$), 2.36 (s, 3H, Ar-C*H*$_3$), 3.53 (m, 1H, C*H*(CH$_3$)$_2$), 3.81 (septet, 1H, $^3J_{HH}$ = 6.6 Hz, C*H*(CH$_3$)$_2$), 4.57 (s, 2H, Ph-C*H*$_2$), 7.20 (d, 2H, $^3J_{HH}$ = 8.0 Hz, C$_{Ar}$*H*), 7.60 (s, 2H, C$_{Ar}$*H*), 7.75-7.77 (m, 3H, C$_{Ar}$*H*).

^{13}C-{^{1}H} NMR (100 MHz, CDCl$_3$, 298K, TMS): δ (ppm) = 19.7 (s, CH(*C*H$_3$)$_2$), 20.0 (s, CH(*C*H$_3$)$_2$), 21.4 (s, *C*H$_3$, Ts), 37.4 (s, *C*H$_2$), 48.5 (s, *C*H(CH$_3$)$_2$), 50.6 (s, *C*H(CH$_3$)$_2$), 120.9 (t, *C*$_{Ar}$H), 121.7 (s, *C*^{IV}-CF$_3$), 124.4 (s, *C*^{IV}-CF$_3$), 126.2 (s, *C*$_{Ar}$H), 128.1 (s, C^{IV}), 129.2 (s, *C*$_{Ar}$H), 132.1 (q, C^{IV}, *C*F$_3$), 137.4 (s, C^{IV}), 140.8 (s, C^{IV}), 142.1 (s, C^{IV}), 161.2 (s, C^{IV}, *C*=N). **^{19}F NMR (377 MHz, CDCl$_3$):** δ (ppm) -63.0.

HRMS (EI) *m/z* for $C_{23}H_{26}F_6N_2O_2S$: theoretical value: 509.1692, observed data: 509.1678.

***N,N*-Diisopropyl-2-(phenanthren-9-yl)-*N'*-tosylacetimidamide (11i)**

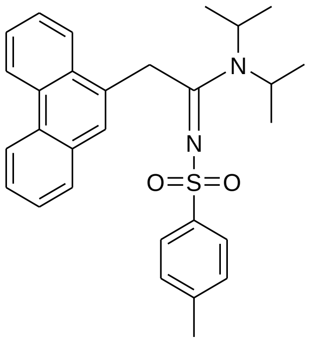

The general procedure yielded, after recrystallization (pentane/dichloromethane: 1/1), the title compound as a colorless solid (368.3 mg, 78%).

^{1}H NMR (400 MHz, CDCl$_3$, 298K, TMS): δ (ppm) = 1.02 (d, 6H, $^3J_{HH}$ = 6.6 Hz, CH(C*H*$_3$)$_2$), 1.66 (d, 6H, $^3J_{HH}$ = 6.8 Hz CH(C*H*$_3$)$_2$), 1.94 (s, 3H, Ar-C*H$_3$*), 3.68 (m, 1H, C*H*(CH$_3$)$_2$), 3.83 (septet, 1H, $^3J_{HH}$ = 6.6 Hz, C*H*(CH$_3$)$_2$), 4.75 (s, 2H, Ph-C*H$_2$*), 6.76 (d, 2H, $^3J_{HH}$ = 7.9 Hz, C$_{Ar}$*H*), 6.98 (s, 1H, C$_{Ar}$*H*), 7.53 (d, 1H, $^3J_{HH}$ = 4.3 Hz, C$_{Ar}$*H*), 7.58-7.63 (m, 3H, C$_{Ar}$*H*), 7.70 (quint, 2H, $^3J_{HH}$ = 8.0 Hz, C$_{Ar}$*H*), 7.96 (d, 2H, $^3J_{HH}$ = 8.0 Hz, C$_{Ar}$*H*), 8.63 (d, 1H, $^3J_{HH}$ = 8.2 Hz, C$_{Ar}$*H*), 8.73 (d, 1H, $^3J_{HH}$ = 8.2 Hz, C$_{Ar}$*H*).

^{13}C-{^{1}H} NMR (100 MHz, CDCl$_3$, 298K, TMS): δ (ppm) = 20.2 (s, CH(*C*H$_3$)$_2$), 20.3 (s, CH(*C*H$_3$)$_2$), 21.0 (Ar-CH$_3$), 34.3 (s, Ph-*C*H$_2$), 48.5 (s, *C*H(CH$_3$)$_2$), 50.8 (s, *C*H(CH$_3$)$_2$), 122.4 (s, *C*$_{Ar}$H), 123.3 (s, *C*$_{Ar}$H), 123.4 (s, *C*$_{Ar}$H), 125.0 (s, *C*$_{Ar}$H), 126.3 (s, *C*$_{Ar}$H), 126.6 (s, *C*$_{Ar}$H), 126.7 (s, *C*$_{Ar}$H), 126.8 (s, *C*$_{Ar}$H), 127.1 (s, *C*$_{Ar}$H), 128.0 (s, C^{IV}), 128.2 (s, *C*$_{Ar}$H), 128.7 (s, *C*$_{Ar}$H), 129.7 (s, C^{IV}), 130.1 (s, C^{IV}), 130.6 (s, C^{IV}), 131.1 (s, C^{IV}), 140.7 (s, C^{IV}), 141.6 (s, C^{IV}), 164.1 (s, C^{IV} *C*=N).

HRMS (EI) *m/z* for $C_{29}H_{32}N_2O_2S$: theoretical value: 473.2257, observed data: 473.2247.

2-(4-(-2-(Diisopropylamino)-2-(tosylimino)ethyl)phenyl)-*N,N*-diisopropyl-*N'*-tosylacetimidamide (11j)

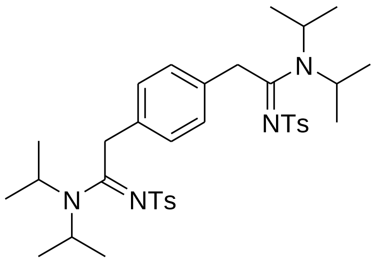

The general procedure yielded, after recrystallization (diethyl ether/acetone: 1/1), the title compound as an orange powder (306.5 mg, 92%).

^{1}H NMR (400 MHz, CDCl$_3$, 298K, TMS): δ (ppm) = 0.89 (d, 12H, $^3J_{HH}$ = 6.6 Hz, CH(C*H*$_3$)$_2$), 1.36 (d, 12H, $^3J_{HH}$ = 6.8 Hz, CH(C*H*$_3$)$_2$), 2.41 (s, 6H, Ar-C*H*$_3$), 3.44 (m, 2H, C*H*(CH$_3$)$_2$), 3.98 (septet, 2H, $^3J_{HH}$ = 6.6 Hz, C*H*(CH$_3$)$_2$), 4.38 (s, 4H, Ph-C*H$_2$*), 7.20 (s, 4H, C$_{Ar}$*H*), 7.25 (d, 4H, $^3J_{HH}$ = 8.3 Hz, C$_{Ar}$*H*), 7.84 (d, 4H, $^3J_{HH}$ = 8.3 Hz, C$_{Ar}$*H*).

^{13}C-{^{1}H} NMR (100 MHz, CDCl$_3$, 298K, TMS): δ (ppm) = 19.8 (s, CH(*C*H$_3$)$_2$), 19.9 (s, CH(*C*H$_3$)$_2$), 21.5 (s, Ar-*C*H$_3$), 38.6 (s, Ph-*C*H$_2$), 48.0 (s, *C*H(CH$_3$)$_2$), 50.4 (s, *C*H(CH$_3$)$_2$), 126.2 (s, *C*$_{Ar}$H), 128.6 (s, *C*$_{Ar}$H), 129.1 (s, *C*$_{Ar}$H), 133.7 (s, C^{IV}), 141.6 (s, C^{IV}),141.7 (s, C^{IV}), 163.1(s, C^{IV} *C*=N).

HRMS (EI) *m/z* for $C_{36}H_{50}N_4O_4S_2$: theoretical value: 667.3346, observed data: 667.3337.

2-(3-(2-(Diisopropylamino)-2-(tosylimino)ethyl)phenyl)-*N,N*-diisopropyl-*N'*-tosylacetimidamide (11k)

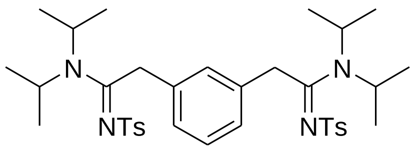

The general procedure yielded, after recrystallization (diethyl ether/acetone: 1/1), the title compound as a yellowish solid (233.2 mg, 85%).

^{1}H NMR (400 MHz, CDCl$_3$, 298K, TMS): δ (ppm) = 0.91 (d, 12H, $^3J_{HH}$ = 6.6 Hz, CH(C*H*$_3$)$_2$), 1.37 (d, 12H, $^3J_{HH}$ = 6.8 Hz, CH(C*H*$_3$)$_2$), 2.41 (s, 6H, Ar-C*H*$_3$), 3.45 (m, 2H, C*H*(CH$_3$)$_2$), 4.02

(septet, 2H, $^{3}J_{HH}$ = 6.6 Hz, C*H*(CH_3)$_2$), 4.40 (s, 4H, Ph-C*H*$_2$), 7.16-7.18 (m, 3H, C_{Ar}*H*), 7.24-7.29 (m, 5H, C_{Ar}*H*), 7.83 (d, 4H, $^{3}J_{HH}$ = 8.3 Hz, C_{Ar}*H*).

^{13}C-{^{1}H} NMR (100 MHz, $CDCl_3$, 298K, TMS): δ (ppm) = 19.8 (s, CH(*C*H_3)$_2$), 19.9 (s, CH(*C*H_3)$_2$), 21.5 (s, Ar-*C*H_3), 39.0 (s, Ph-*C*H_2), 48.0 (s, *C*H(CH_3)$_2$), 50.6 (s, *C*H(CH_3)$_2$), 126.1 (s, *C*$_{Ar}$H), 126.5 (s, *C*$_{Ar}$H), 128.3 (s, *C*$_{Ar}$H), 129.1 (s, *C*$_{Ar}$H), 129.4 (s, *C*$_{Ar}$H), 135.7 (s, C^{IV}), 141.6 (s, C^{IV}), 141.7 (s, C^{IV}), 163.0(s, C^{IV} *C*=N).

HRMS (EI) *m/z* for $C_{36}H_{50}N_4O_4S_2$: theoretical value: 667.3346, observed data: 667.3328.

N,N-Diisopropyl-*N'*-tosyloctanimidamide[8] (11l)

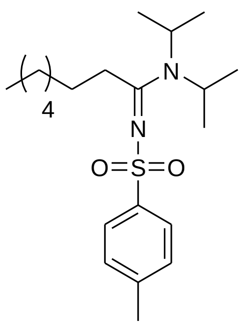

The general procedure yielded, after recrystallization (diethyl ether/acetone: 9/1), the title compound as a colorless solid (213.1 mg, 63%).

^{1}H NMR (400 MHz, $CDCl_3$, 298K, TMS): δ (ppm) = 0.89 (m, 2H, -(C*H*$_2$)-), 1.10 (m, 4H, -(C*H*$_2$)-) overlapped with 1.23 (d, 6H, $^{3}J_{HH}$ = 6.8 Hz, CH(C*H*$_3$)$_2$) and 1.32 (d, 6H, $^{3}J_{HH}$ = 6.8 Hz, CH(C*H*$_3$)$_2$), 1.50 (m, 2H, -(C*H*$_2$)-), 1.76 (m, 5H, -(C*H*$_2$)- and C*H*$_3$), 2.39 (s, 3H, ArC*H*$_3$), 2.87-2.91 (m, 2H, C*H*$_2$-C=N), 3.49 (m, 1H, C*H*(CH_3)$_2$), 4.02 (septet, 1H, $^{3}J_{HH}$ = 6.8 Hz, CH(CH_3)$_2$), 7.24 (d, 2H, $^{3}J_{HH}$ = 8.2 Hz, C_{Ar}*H*), 7.81 (d, 2H, $^{3}J_{HH}$ = 8.2 Hz, C_{Ar}*H*).

^{13}C-{^{1}H} NMR (100 MHz, $CDCl_3$, 298K, TMS): δ (ppm) = 20.1 (s, CH(*C*H_3)$_2$), 20.7 (s, CH(*C*H_3)$_2$), 21.4 (s, Ar-*C*H_3), 26.2 (s, -*C*H_2-), 26.5 (s, -*C*H_2-), 26.7 (s, *C*H_3), 30.6 (s, -*C*H_2-), 32.9 (s, -*C*H_2-), 34.5 (s, -*C*H_2-), 38.1 (s, *C*H_2-CN), 47.9 (s, *C*H(CH_3)$_2$), 49.8 (s, *C*H(CH_3)$_2$), 126.1 (s, *C*$_{Ar}$H), 129.0 (s, *C*$_{Ar}$H), 141.3 (s, C^{IV}), 141.9 (s, C^{IV}), 166.9 (s, C^{IV} *C*=N).

4-Bromo-*N,N*-diisopropyl-*N'*-tosylbutanimidamide (11m)

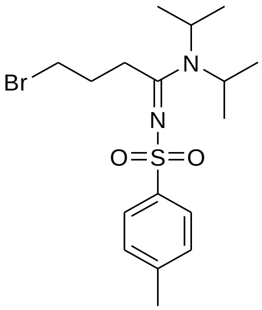

The general procedure yielded, after column chromatography (pentane/ethyl acetate: 7/3), the title compound as a colorless solid (281.5 mg, 70%).

^{1}H NMR (400 MHz, $CDCl_3$, 298K, TMS): δ (ppm) = 1.25 (d, 6H, $^3J_{HH}$ = 6.7 Hz, CH(CH_3)$_2$), 1.31 (d, 6H, $^3J_{HH}$ = 6.7 Hz, CH(CH_3)$_2$), 2.22-2.29 (m, 2H, -(CH_2)-), 2.40 (s, 3H, Ar-CH_3), 3.06-3.11 (m, 2H, -CH_2-C=N), 3.47-3.61 (m, 3H, -CH_2-Cl + CH(CH$_3$)$_2$), 4.14 (septet, 1H, $^3J_{HH}$ = 6.7 Hz, CH(CH$_3$)$_2$), 7.25 (d, 2H, $^3J_{HH}$ = 8.3 Hz, C$_{Ar}$$H$), 7.80 (d, 2H, $^3J_{HH}$ = 8.3 Hz, C$_{Ar}$$H$).

^{13}C-{^{1}H} NMR (100 MHz, $CDCl_3$, 298K, TMS): δ (ppm) = 20.0 (s, CH(CH$_3$)$_2$), 20.7 (s, CH(CH$_3$)$_2$), 21.5 (s, Ar-CH$_3$), 30.2 (s, -CH$_2$-), 31.6 (s, -CH$_2$-), 33.6 (s, -CH$_2$-CN), 48.1 (s, CH(CH$_3$)$_2$), 50.0 (s, CH(CH$_3$)$_2$), 126.0 (s, C_{Ar}H), 129.1 (s, C_{Ar}H), 141.5 (s, C^{IV}), 141.6 (s, C^{IV}), 164.8 (s, C^{IV} C=N).

HRMS (EI) *m/z* for $C_{18}H_{29}BrN_2O_2S$: theoretical value: 405.1030, observed data: 405.1034.

6-Chloro-*N,N*-diisopropyl-*N'*-tosylhexanimidamide (11n)

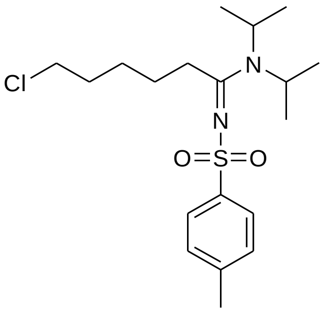

The general procedure yielded, after recrystallization (diethyl ether/acetone: 9/1), the title compound as a colorless solid (270.3 mg, 70%).

^{1}H NMR (400 MHz, $CDCl_3$, 298K, TMS): δ (ppm) = 1.23 (d, 6H, $^{3}J_{HH}$ = 6.7 Hz, $CH(CH_3)_2$), 1.31 (d, 6H, $^{3}J_{HH}$ = 6.7 Hz, $CH(CH_3)_2$), 1.54-1.62 (m, 2H, -(CH_2)-), 1.63-1.72 (m, 2H, -(CH_2)-), 1.79-1.86 (m, 2H, -(CH_2)-), 2.39 (s, 3H, Ar-CH_3), 2.88-2.92 (m, 2H, CH_2-C=N), 3.45-3.58 (m, 3H, $CH(CH_3)_2$ + CH_2-Cl), 4.02 (septet, 1H, $^{3}J_{HH}$ = 6.7 Hz, $CH(CH_3)_2$), 7.24 (d, 2H, $^{3}J_{HH}$ = 8.3 Hz, $C_{Ar}H$), 7.80 (d, 2H, $^{3}J_{HH}$ = 8.3 Hz, $C_{Ar}H$).

^{13}C-{^{1}H} NMR (100 MHz, $CDCl_3$, 298K, TMS): δ (ppm) = 20.0 (s, $CH(CH_3)_2$), 20.7 (s, $CH(CH_3)_2$), 21.4 (s, Ar-CH_3), 26.5 (s, -(CH_2)-), 27.0 (s, -(CH_2)-), 31.9 (s, -(CH_2)-), 32.6 (s, -(CH_2)-), 44.8 (s, -CH_2-CN), 48.0 (s, $CH(CH_3)_2$), 49.9 (s, $CH(CH_3)_2$), 126.0 (s, C_{Ar}H), 129.0 (s, C_{Ar}H), 141.5 (s, C^{IV}), 141.8 (s, C^{IV}), 165.9 (s, C^{IV} *C*=N).

HRMS (EI) *m/z* for $C_{19}H_{31}ClN_2O_2S$, theoretical value: 387.1868, observed data: 387.1866.

5-Chloro-*N,N*-diisopropyl-*N'*-tosylpentanimidamide[4] (11o)

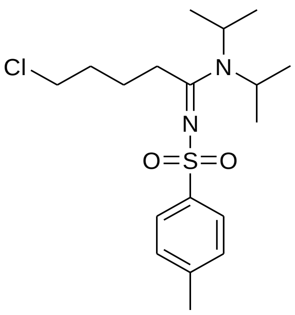

The general procedure yielded, after recrystallization (diethyl ether/acetone: 9/1), the title compound as a colorless solid (264.2 mg, 71%).

^{1}H NMR (400 MHz, $CDCl_3$, 298K, TMS): δ (ppm) = 1.25 (d, 6H, $^{3}J_{HH}$ = 6.7 Hz, $CH(CH_3)_2$), 1.32 (d, 6H, $^{3}J_{HH}$ = 6.7 Hz, $CH(CH_3)_2$), 1.79-1.87 (m, 2H, -(CH_2)-), 1.91-1.97 (m, 2H, -(CH_2)-), 2.40 (s, 3H, Ar-CH_3), 2.91-2.96 (m, 2H, CH_2-C=N), 3.35-3.55 (m, 1H, $CH(CH_3)_2$), 3.60 (t, 2H, $^{3}J_{HH}$ = 6.3 Hz, -CH_2-Cl), 4.07 (septet, 1H, $^{3}J_{HH}$ = 6.7 Hz, $CH(CH_3)_2$), 7.25 (d, 2H, $^{3}J_{HH}$ = 8.3 Hz, $C_{Ar}H$), 7.80 (d, 2H, $^{3}J_{HH}$ = 8.3 Hz, $C_{Ar}H$).

^{13}C-{^{1}H} NMR (100 MHz, $CDCl_3$, 298K, TMS): δ (ppm) = 20.0 (s, $CH(CH_3)_2$), 20.7 (s, $CH(CH_3)_2$), 21.4 (s, Ar-CH_3), 24.2 (s, -(CH_2)-), 31.8 (s, -(CH_2)-), 32.0 (s, -(CH_2)-), 44.4 (s, -CH_2-CN), 48.0 (s, $CH(CH_3)_2$), 50.0 (s, $CH(CH_3)_2$), 126.0 (s, C_{Ar}H), 129.0 (s, C_{Ar}H), 141.5 (s, C^{IV}), 141.7 (s, C^{IV}), 165.5 (s, C^{IV}, *C*=N).

4-Cyclohexyl-*N,N*-diisopropyl-*N'*-tosylbutanimidamide (11p)

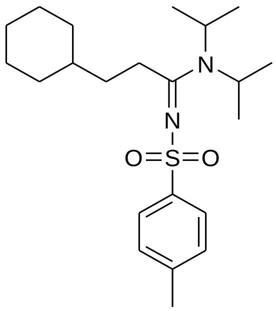

The general procedure yielded, after column chromatography (pentane/ethyl acetate: 8/2), the title compound as a colorless solid (243.2 mg, 62%).

^{1}H NMR (400 MHz, $CDCl_3$, 298K, TMS): δ (ppm) = 0.89-1.76 (m, 15H, CH_2 + cyclohexyl) overlapped with 1.23 (d, 6H, $^3J_{HH}$ = 6.7 Hz, $CH(CH_3)_2$) and 1.32 (d, 6H, $^3J_{HH}$ = 6.8 Hz, $CH(CH_3)_2$), 2.39 (s, 3H, Ar-CH_3), 2.87-2.91 (m, 2H, -CH_2-C=N), 3.49 (m, 1H, $CH(CH_3)_2$), 4.02 (septet, 1H, $^3J_{HH}$ = 6.7 Hz, $CH(CH_3)_2$), 7.24 (d, 2H, $^3J_{HH}$ = 8.3 Hz, $C_{Ar}H$), 7.81 (d, 2H, $^3J_{HH}$ = 8.3 Hz, $C_{Ar}H$).

^{13}C-{^{1}H} NMR (100 MHz, $CDCl_3$, 298K, TMS): δ (ppm) = 20.1 (s, $CH(CH_3)_2$), 20.7 (s, $CH(CH_3)_2$), 21.4 (s, Ar-CH_3), 26.2 (s, -(CH_2)-), 26.5 (s, -(CH_2)-), 30.6 (s, -(CH_2)-), 32.9 (s, -(CH_2)-), 34.5 (s, -CH-), 38.1 (s, CH_2-CN), 47.9 (s, $CH(CH_3)_2$), 49.8 (s, $CH(CH_3)_2$), 126.1 (s, $C_{Ar}H$), 129.0 (s, $C_{Ar}H$), 141.3 (s, C^{IV}), 141.9 (s, C^{IV}), 166.9 (s, C^{IV} C=N).

HRMS (EI) *m/z* for $C_{22}H_{36}N_2O_2S$: theoretical value: 393.2570, observed data: 393.2565.

***N,N*-Diisopropyl-4-phenyl-*N'*-tosylbutanimidamide (11q)**

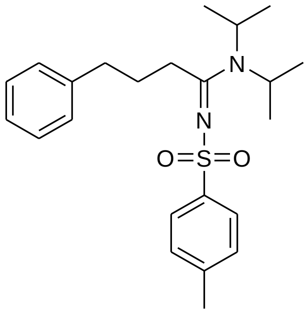

The general procedure yielded, after recrystallization (hexane/acetone: 9/1), the title compound as a colorless solid (312.2 mg, 78%).

^{1}H NMR (400 MHz, $CDCl_3$, 298K, TMS): δ (ppm) = 1.07 (d, 6H, $^3J_{HH}$ = 6.7 Hz, CH(CH_3)$_2$), 1.29 (d, 6H, $^3J_{HH}$ = 6.7 Hz, CH(CH_3)$_2$), 1.90-1.98 (m, 2H, -(CH_2)-), 2.39 (s, 3H, Ar-CH_3), 2.73 (t, 2H, $^3J_{HH}$ = 7.5 Hz, Ph-CH_2), 2.86-2.90 (m, 2H, -CH_2-C=N), 3.44 (m, 1H, C*H*(CH_3)$_2$), 3.63 (septet, 1H, $^3J_{HH}$ = 6.7 Hz, CH(CH_3)$_2$), 7.18-7.25 (m, 5H, $C_{Ar}H$), 7.27-7.31 (m, 2H, $C_{Ar}H$), 7.81 (d, 2H, $^3J_{HH}$ = 8.3 Hz, $C_{Ar}H$).

^{13}C-{^{1}H} NMR (100 MHz, $CDCl_3$, 298K, TMS): δ (ppm) = 20.0 (s, CH(*C*H_3)), 20.5 (s, CH(*C*H_3)), 21.4 (s, Ar-*C*H_3), 29.0 (s, -(*C*H_2)-), 31.9 (s, -(*C*H_2)-), 35.6 (s, -*C*H_2-CN), 47.9 (s, *C*H(CH_3)$_2$), 49.7 (s, *C*H(CH_3)$_2$), 126.1 (s, C_{Ar}H), 126.2 (s, C_{Ar}H), 128.4 (s, C_{Ar}H), 128.6 (s, C_{Ar}H), 129.0 (s, C_{Ar}H), 141.1 (s, C^{IV}), 141.4 (s, C^{IV}), 141.8 (s, C^{IV}), 166.1 (s, C^{IV} *C*=N).

HRMS (EI) *m/z* for $C_{23}H_{32}N_2O_2S$, theoretical value: 401.2257, observed data: 401.2253.

3-(Diisopropylamino)-1-phenyl-4-tosylbut-3-en-1-ol (11r)

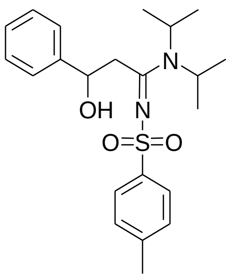

The general procedure yielded, after recrystallization (pentane/dichloromethane: 8/2), the title compound as a colorless solid (268 mg, 67%).

^{1}H NMR (400 MHz, $CDCl_3$, 298K, TMS): δ (ppm) = 1.23 (dd, 6H, $^3J_{HH}$ = 5.6 Hz, CH(CH_3)$_2$), 1.30 (d, 3H, $^3J_{HH}$ = 6.8 Hz, CH(CH_3)$_2$), 1.37 (d, 3H, $^3J_{HH}$ = 6.8 Hz, CH(CH_3)$_2$), 2.41 (s, 3H, Ar-CH_3), 3.22 (dd, 1H, $^3J_{HH}$ = 4.3 Hz, 1H from C*H*(CH_3)$_2$), 3.52-3.65 (m, 2H, 1H from C*H*-OH overlapped to C*H*(CH_3)$_2$), 4.25-4.32 (m, 2H, 1H from CH_2-C=N), 5.13-5.18 (m, 1H, O*H*), 7.25-7.31 (m, 3H, $C_{Ar}H$),7.38 (t, 2H, $^3J_{HH}$ = 7.8 Hz, $C_{Ar}H$), 7.51 (m, 2H, $^3J_{HH}$ = 7.8 Hz, $C_{Ar}H$), 7.84 (d, 2H, $^3J_{HH}$ = 8.3 Hz, $C_{Ar}H$).

^{13}C-{^{1}H} NMR (100 MHz, $CDCl_3$, 298K, TMS): δ (ppm) = 19.9(s, 3*C*, *CH*(*C*H_3)), 20.1 (s, 3*C*, CH(*C*H_3)), 20.5 (d, 6*C*, CH(*C*H_3)), 21.5 (s, *C*H_3 (Ts)), 42.2 (s, *C*H_2-CN), 48.4 (s, *C*H(CH_3)$_2$), 51.0 (s, *C*H(CH_3)$_2$), 72.1 (s, *C*H-OH), 125.5 (s, C_{Ar}H), 126.1 (s, C_{Ar}H), 127.8 (s, C_{Ar}H), 128.7 (s, C_{Ar}H), 129.1 (s, C_{Ar}H), 141.2 (s, C^{IV}), 141.8 (s, C^{IV}), 144.0 (s, C^{IV}), 162.8 (s, C^{IV}, *C*=N).

HRMS (EI) *m/z* for $C_{22}H_{30}N_2O_3S$, theoretical value: 403.2050, observed data: 403.2045.

***N,N*-Dicyclohexyl-2-phenyl-*N'*-tosylacetimidamide (12a)**

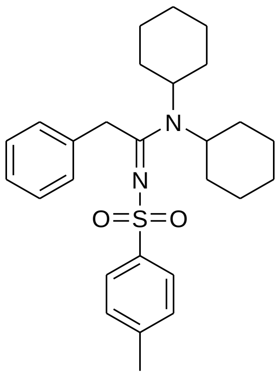

The general procedure yielded, after recrystallization (heptane/dichloromethane: 9/1), the title compound as a colorless solid (289.4 mg, 64%).

^{1}H NMR (400 MHz, $CDCl_3$, 298K, TMS): δ (ppm) = 0.86-1.70 (m, 20H, CH_2 cyclohexyl), 2.40 (s, 3H, Ar-CH_3), 2.58-2.60 (m, 1H, -C*H*-), 2.94 (m, 1H, -C*H*-), 4.40 (s, 2H, Ph-CH_2), 7.20-7.30 (m, 7H, $C_{Ar}H$), 7.84 (d, 2H, $^3J_{HH}$ = 8.3 Hz, $C_{Ar}H$).

^{13}C-{^{1}H} NMR (100 MHz, $CDCl_3$, 298K, TMS): δ (ppm) = 21.5 (s, Ar-CH_3), 25.0 (s, CH_2 cyclohexyl), 25.3 (s, CH_2 cyclohexyl), 25.6 (s, CH_2 cyclohexyl), 26.4 (s, CH_2 cyclohexyl), 28.6 (s, CH_2 cyclohexyl), 30.2 (s, CH_2 cyclohexyl), 39.4 (s, Ph-CH_2), 58.6 (s, *C*H cyclohexyl), 59.3 (s, *C*H cyclohexyl), 126.2 (s, C_{Ar}H), 126.8 (s, C_{Ar}H), 128.2 (s, C_{Ar}H), 128.8 (s, C_{Ar}H), 129.0 (s, C_{Ar}H), 135.4 (s, C^{IV}), 141.5 (s, C^{IV}), 141.6 (s, C^{IV}), 163.7 (s, C^{IV} *C*=N).

HRMS (EI) *m/z* for $C_{27}H_{36}N_2O_2S$, theoretical value: 453.2570, observed data: 453.2565.

***N*-Isopropyl-2-phenyl-*N'*-tosylacetimidamide[4] (12b)**

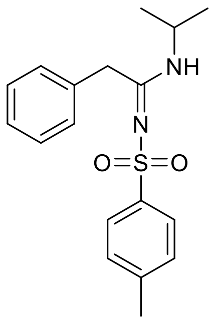

The general procedure yielded, after column chromatography (pentane/dichloromethane: 7/3), the title compound as colorless solid (237.7 mg, 72%).

^{1}H NMR (400 MHz, $CDCl_3$, 298K): δ (ppm) = 1.00 (d, 6H, $^3J_{HH}$ = 6.6 Hz, CH(CH_3)$_2$), 2.41 (s, 3H, Ar-CH_3), 4.09 (septet, 1H, $^3J_{HH}$ = 6.6 Hz, CH(CH_3)$_2$), 4.26 (s, 2H, Ph-CH_2), 5.00 (br. s, 1H, NH), 7.18 (d, $^3J_{HH}$ = 7.8 Hz, $C_{Ar}H$), 7.27 (d, $^3J_{HH}$ = 8.0 Hz, $C_{Ar}H$), 7.27-7.39 (m, 3H, $C_{Ar}H$), 7.86 (d, 2H, $^3J_{HH}$ = 7.8 Hz, $C_{Ar}H$).

^{13}C-{^{1}H} NMR (100 MHz, $CDCl_3$, 298K, TMS): δ (ppm) = 21.7 (s, CH(CH_3)$_2$), 23.4 (s, CH_3, Ts), 39.8 (s, CH_2), 43.9 (s, CH(CH_3)$_2$), 126.3 (s, C_{Ar}H), 126.3 (s, C_{Ar}H), 128.2 (s, C_{Ar}H), 129.2 (s, C_{Ar}H), 129.5 (s, C_{Ar}H), 130.1 (s, C_{Ar}H), 133.1 (s, C^{IV}), 140.1 (s, C^{IV}), 142.1 (s, C^{IV}), 165.4 (s, C^{IV}, C=N).

Diphenyl (1-(diisopropylamino)-2-phenylethylidene)phosphoramidate[6]

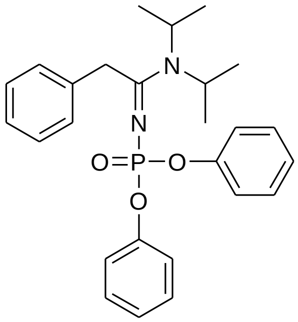

The general procedure yielded, after recrystallization (diethyl ether/acetone: 9/1), the title compound as a colorless solid (238.6 mg, 53%).

^{1}H NMR (400 MHz, $CDCl_3$, 298K, TMS): δ (ppm) = 0.88 (d, 6H, $^3J_{HH}$ = 6.6 Hz, CH(CH_3)$_2$), 1.22 (d, 6H, $^3J_{HH}$ = 6.8 Hz CH(CH_3)$_2$), 3.36 (m, 1H, CH(CH_3)$_2$), 3.97 (septet, 1H, $^3J_{HH}$ = 6.6 Hz CH(CH_3)$_2$ CH(CH_3)$_2$), 4.29 (s, 2H, Ph-CH_2), 7.08 (t, 2H, $^3J_{HH}$ = 6.9 Hz, $C_{Ar}H$), 7.19-7.29 (m, 13H,$C_{Ar}H$).

^{13}C-{^{1}H} NMR (100 MHz, $CDCl_3$, 298K, TMS): δ (ppm) = 19.5 (s, CH(CH_3)$_2$), 19.9 (s, CH(CH_3)$_2$), 41.7 (s, CH_2), 47.7 (s, CH(CH_3)$_2$), 50.8 (s, CH(CH_3)$_2$), 120.6 (s, C_{Ar}H), 124.0 (s, C_{Ar}H), 126.7 (s, C_{Ar}H), 127.9 (s, C^{IV}-CF_3), 128.8 (s, C_{Ar}H), 129.3 (s, C_{Ar}H), 135.4 (s, C^{IV}), 151.9 (s, C^{IV}), 166.1 (s, C^{IV}, C=N).

^{31}P-{^{1}H} NMR (162 MHz, $CDCl_3$, 298K): δ (ppm) = -5.46

4. Calculation of % V_{Bur}

4.1. [Cu(Pyr)(IPr)]OTf (4)

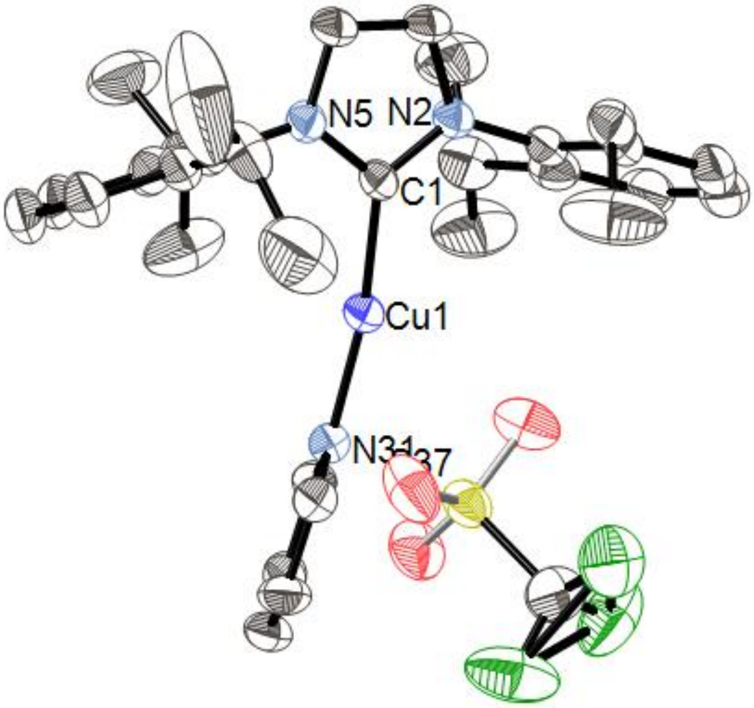

V Free	V Buried	V Total	V Exact
102.2	77.3	179.5	179.6

%V_Free	%V_Bur	% Tot/Ex
56.9	**43.1**	100.0

xy	V_f	V_b	V_t	%V_f	%V_b
--	24.8	20.0	44.9	55.4	**44.63**
-+	29.9	15.0	44.9	66.6	**33.42**
++	25.6	19.3	44.9	57	**43.00**
+-	21.9	23.0	44.9	48.8	**51.17**

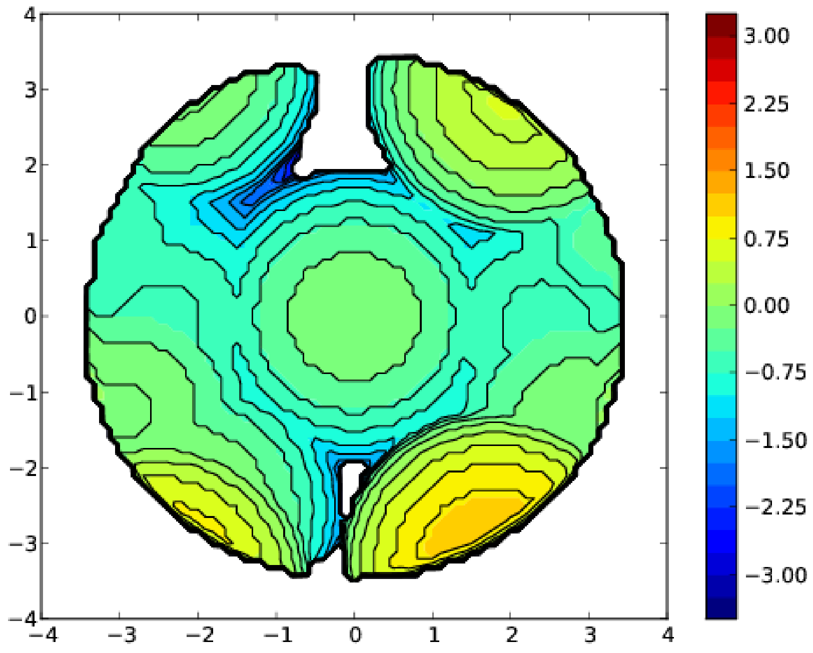

4.2. [Cu(IPr)(Triaz)]BF_4 (5)

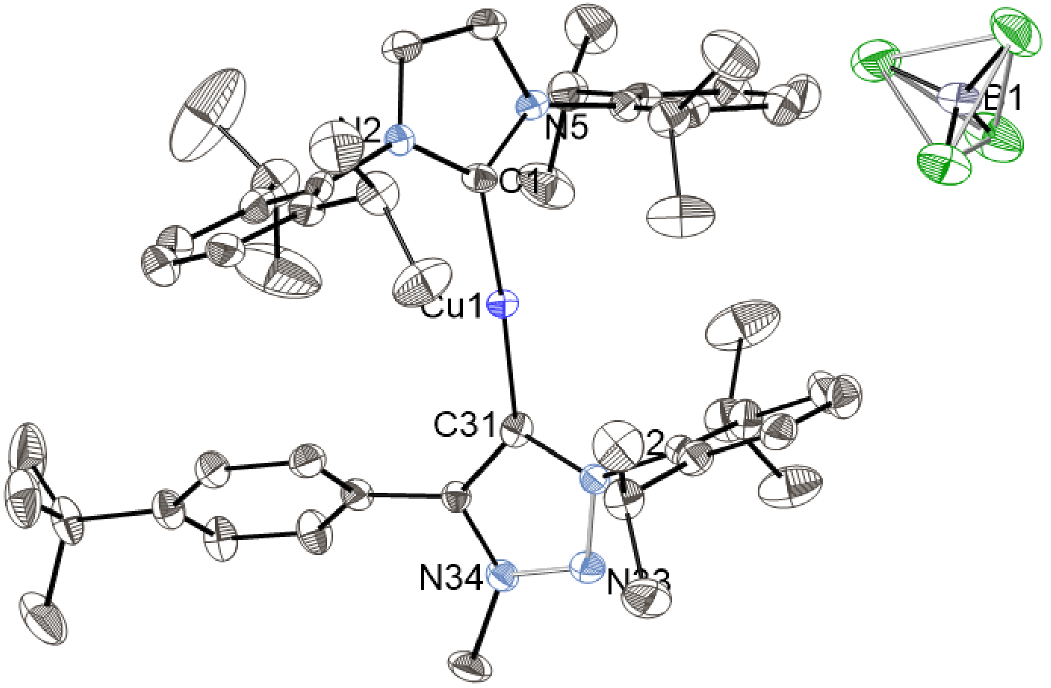

4.2.1. Ligand IPr

V Free	V Buried	V Total	V Exact
100.7	78.8	179.5	179.6

%V_Free	%V_Bur	% Tot/Ex
56.1	**43.9**	100

xy	V_f	V_b	V_t	%V_f	%V_b
−−	24.0	20.9	44.9	53.5	**46.48**
−+	27.7	17.2	44.9	61.7	**38.34**
++	26.2	18.6	44.9	58.5	**41.50**
+−	22.8	22.1	44.9	50.7	**49.26**

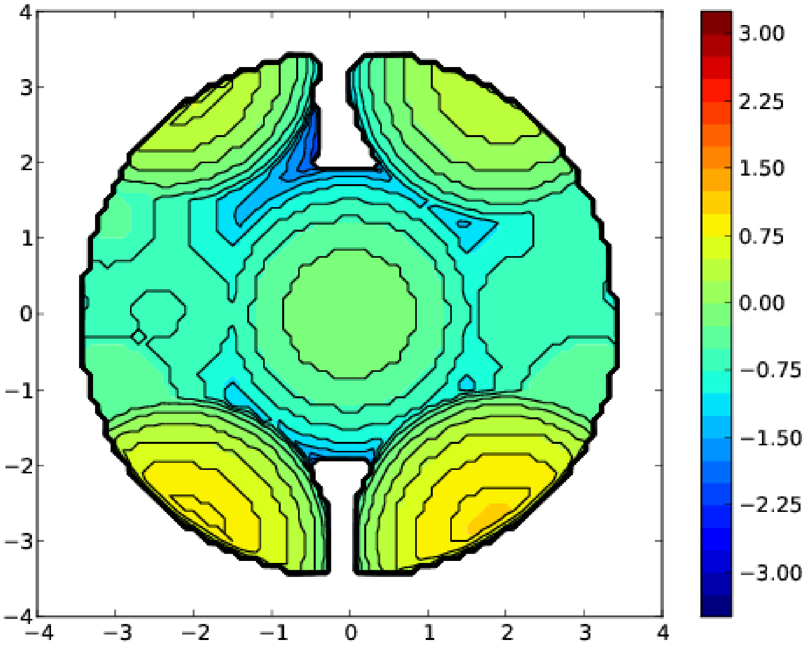

4.2.2. Ligand Triaz

V Free	V Buried	V Total	V Exact
123.5	56.0	179.5	179.6

%V_Free	%V_Bur	% Tot/Ex
68.8	31.2	100.0

xy	V_f	V_b	V_t	%V_f	%V_b
−−	34.0	10.9	44.9	75.8	24.23
−+	29.7	15.2	44.9	66.2	33.83
++	30.1	14.8	44.9	67.0	32.97
+−	29.7	15.2	44.9	66.2	33.78

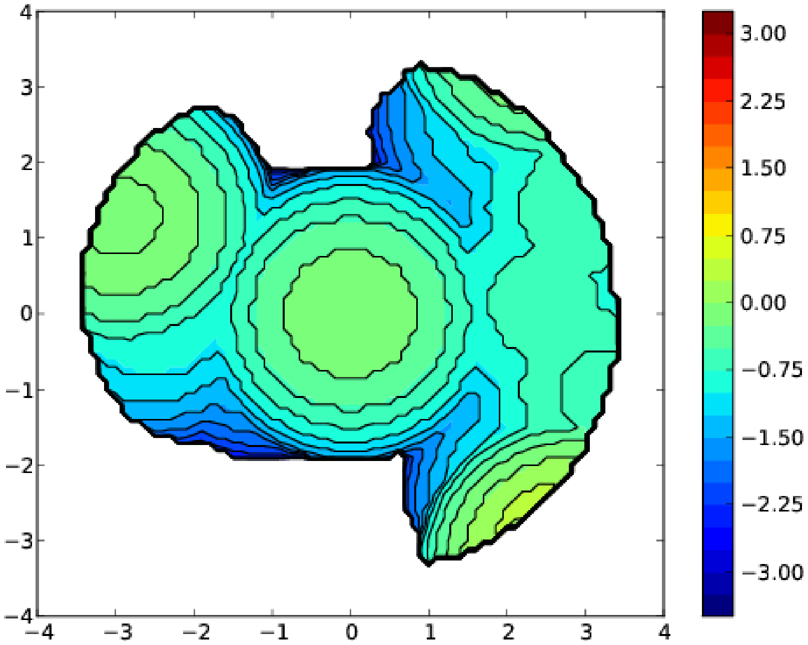

4.3 [Cu(Triaz)$_2$]BF$_4$ (6)

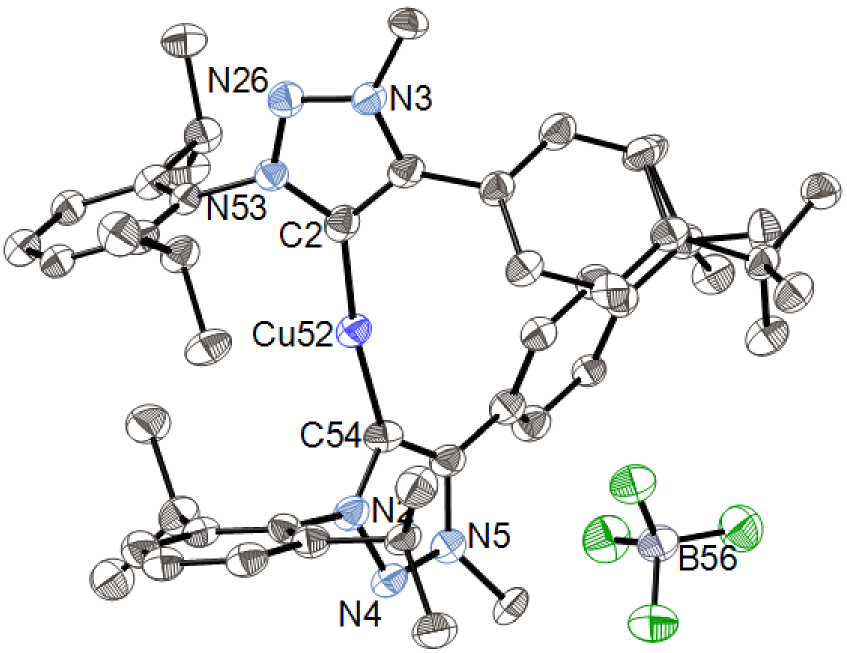

4.3.1. Ligand Triaz L1

V Free	V Buried	V Total	V Exact
114.7	64.8	179.5	179.6

%V_Free	%V_Bur	% Tot/Ex
63.9	**36.1**	100.0

xy	V_f	V_b	V_t	%V_f	%V_b
--	23.9	21.0	44.9	53.3	**46.73**
-+	29.5	15.4	44.9	65.7	**34.27**
++	28.3	16.5	44.9	63.2	**36.85**
+-	32.9	11.9	44.9	73.4	**26.61**

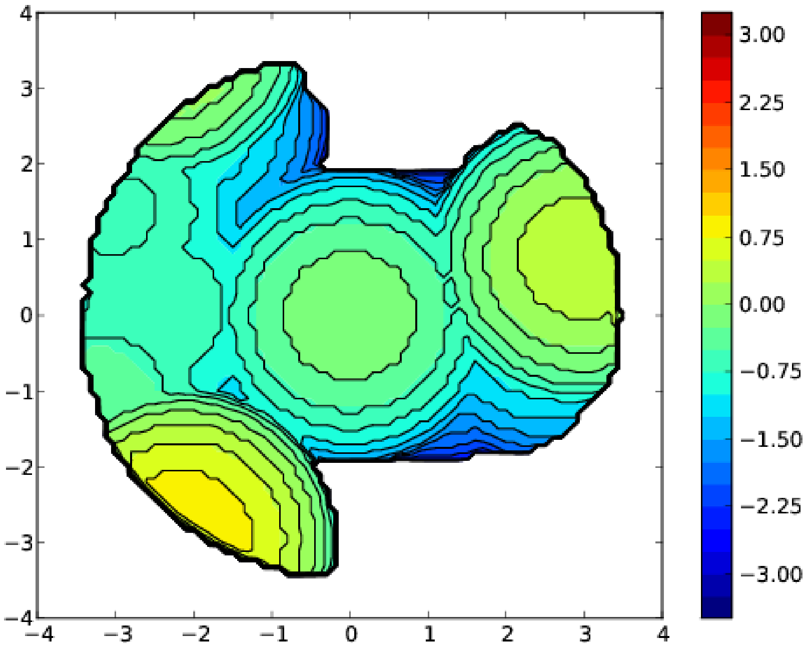

4.3.2 Ligand Triaz L2

V Free	V Buried	V Total	V Exact
115.7	63.8	179.5	179.6

%V_Free	%V_Bur	% Tot/Ex
64.5	35.5	100.0

xy	V_f	V_b	V_t	%V_f	%V_b
−−	32.8	12.1	44.9	73.0	27.00
−+	28.6	16.3	44.9	63.8	36.22
++	25.0	19.8	44.9	55.8	44.25
+−	29.3	15.6	44.9	65.3	34.69

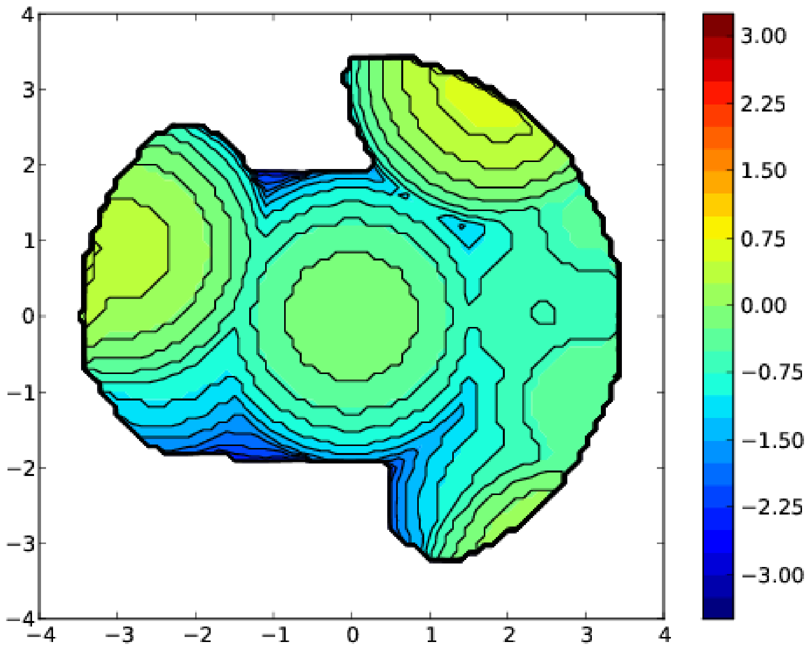

4
3
2
1
0
−1
−2
−3
−4
−4
−3
−2
−1
0
1
2
3
4
3.00
2.25
1.50
0.75
0.00
−0.75
−1.50
−2.25
−3.00

5. Mechanistic studies

5.1. Stoichiometric reaction 1: [Cu(Triaz)$_2$]BF$_4$ and phenylacetylene

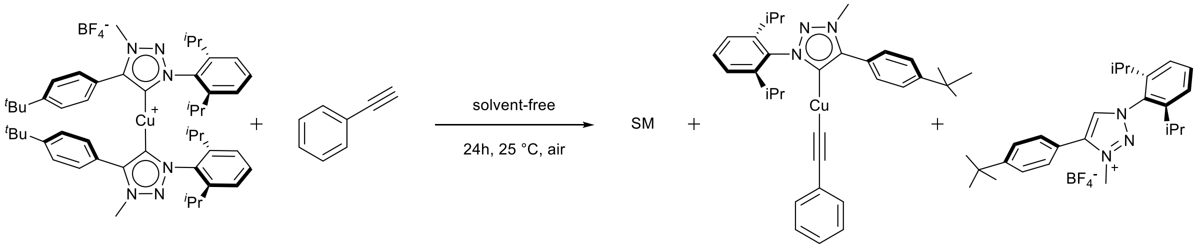

A vial was charged, in air, with [Cu(Triaz)$_2$]BF$_4$ (18.0 mg, 0.02 mmol) and phenylacetylene (2.2 μL, 0.02 mmol). The reaction mixture was stirred for 16 hours at room temperature under solvent-free conditions. $CDCl_3$ was added to the reaction mixture, which was analysed by ^{1}H NMR spectroscopy. The reaction led to the formation of the acetylide intermediate, [Cu(Triaz)(C≡CPh)] and the release of the triazolium salt Triaz·HBF$_4$. The presence of starting material was observed.

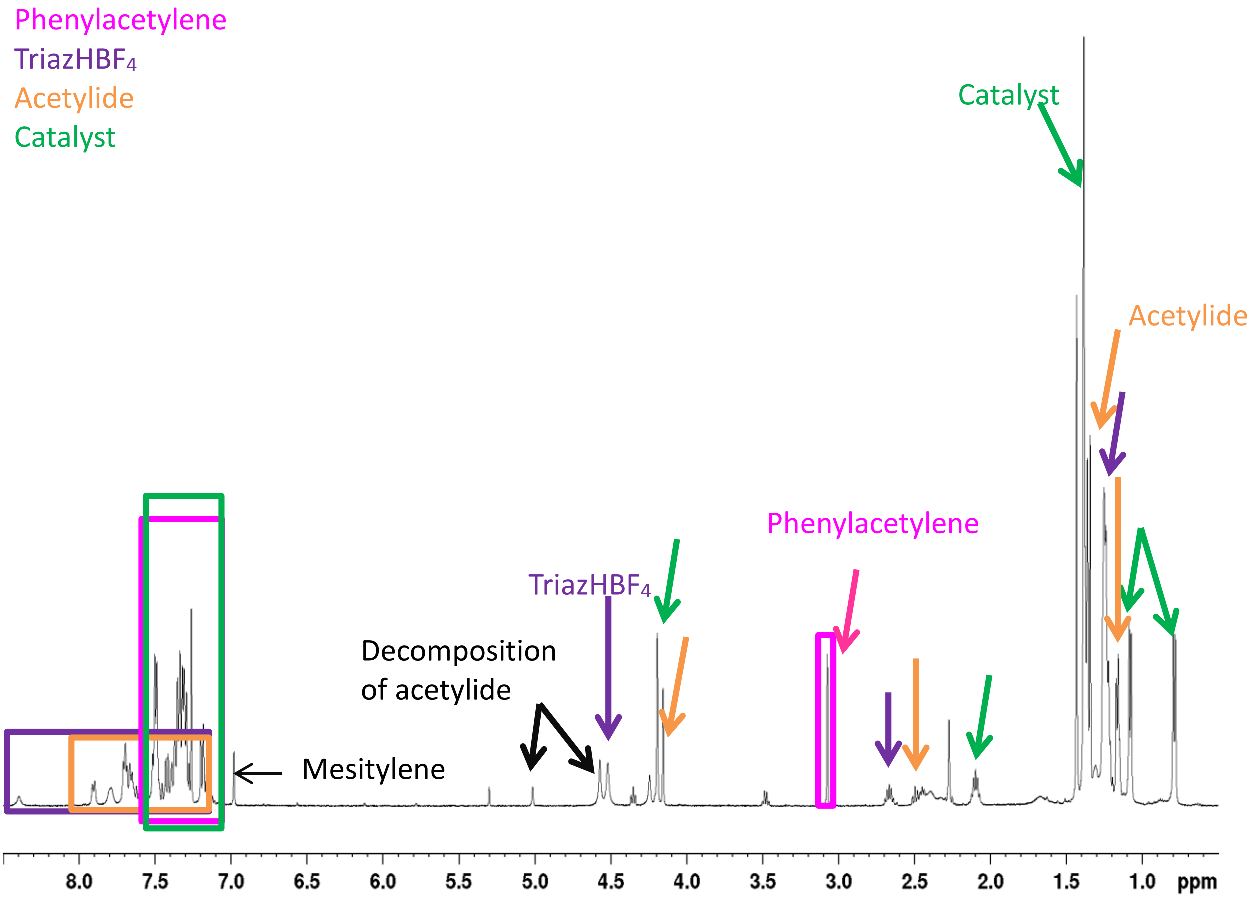

5.2. Synthesis of [Cu(Triaz)(C≡CPh)]

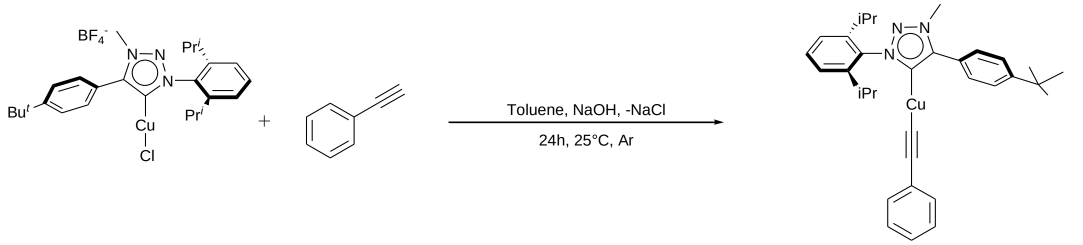

A vial was charged with [Cu(Cl)(Triaz)] (50.0 mg, 0.10 mmol), NaOH (16.0 mg, 0.40 mmol, 4 equivs.), phenylacetylene (110 µL, 1.00 mmol, 10 equivs.) and toluene (1.5 mL). The reaction mixture was stirred for 24 hours at room temperature under inert atmosphere. The solution was then filtered through a plug of celite, concentrated and pentane (10 mL) was added. The product was collected by filtration as a colourless solid (49 mg, 91%). The ^{13}C-{1H}-NMR was carried out in *D*-chloroform at −50 °C due to its high instability.

^{1}H NMR (500 MHz, $CDCl_3$, 298K, TMS): δ (ppm) = 1.15 (d, 6H, $^3J_{HH}$ = 6.8 Hz, CH(CH_3)$_2$), 1.34 (d, 6H, $^3J_{HH}$ = 6.8 Hz, CH(CH_3)$_2$), 1.37 (s, 9H, C(CH_3)$_3$), 2.35 (septet, 2H, $^3J_{HH}$ = 6.7 Hz, CH(CH_3)$_2$), 4.23 (s, 3H, N-CH_3), 7.07 (t, 1H, $^3J_{HH}$ = 8.0 Hz, $C_{Ar}H$), 7.13 (t, 2H, $^3J_{HH}$ = 7.6 Hz, $C_{Ar}H$), 7.30 (d, 2H, $^3J_{HH}$ = 7.8 Hz, $C_{Ar}H$), 7.37 (d, 2H, $^3J_{HH}$ = 7.6 Hz, $C_{Ar}H$), 7.51 (t, 1H, $^3J_{HH}$ = 7.8 Hz, $C_{Ar}H$), 7.56 (d, 2H, $^3J_{HH}$ = 7.8 Hz, $C_{Ar}H$), 7.72 (t, 2H, $^3J_{HH}$ = 7.8 Hz, $C_{Ar}H$). **^{13}C-{^1H} NMR (100 MHz, $CDCl_3$, 222.5K, TMS):** δ (ppm) = 21.6 (s, *C*H(CH_3)$_2$), 24.2 (s, CH(CH_3)$_2$), 24.6 (s, CH(CH_3)$_2$), 28.4 (s, *C*H(CH_3)$_2$), 31.1 (s, C(CH_3)$_3$), 34.8 (s, *C*(CH_3)$_3$), 37.6 (s, NCH_3), 106.1 (s, C^{IV}, C≡C), 120.1 (s, C^{IV}, C≡C), 152.8 (s, C^{IV}), 124.0 (s, C_{Ar}H), 126.1 (s, C_{Ar}H), 127.7 (s, C_{Ar}H), 128.2 (s, C_{Ar}H), 129.0 (s, C_{Ar}H), 129.2 (s, C_{Ar}H), 130.9 (s, C^{IV}), 131.8 (s, C_{Ar}H), 135.2 (s, C^{IV}), 137.9 (s, C^{IV}), 144.7 (s, C^{IV}), 148.7 (s, C^{IV}), 152.8 (s, C^{IV}), 167.5 (s, C^{IV}).

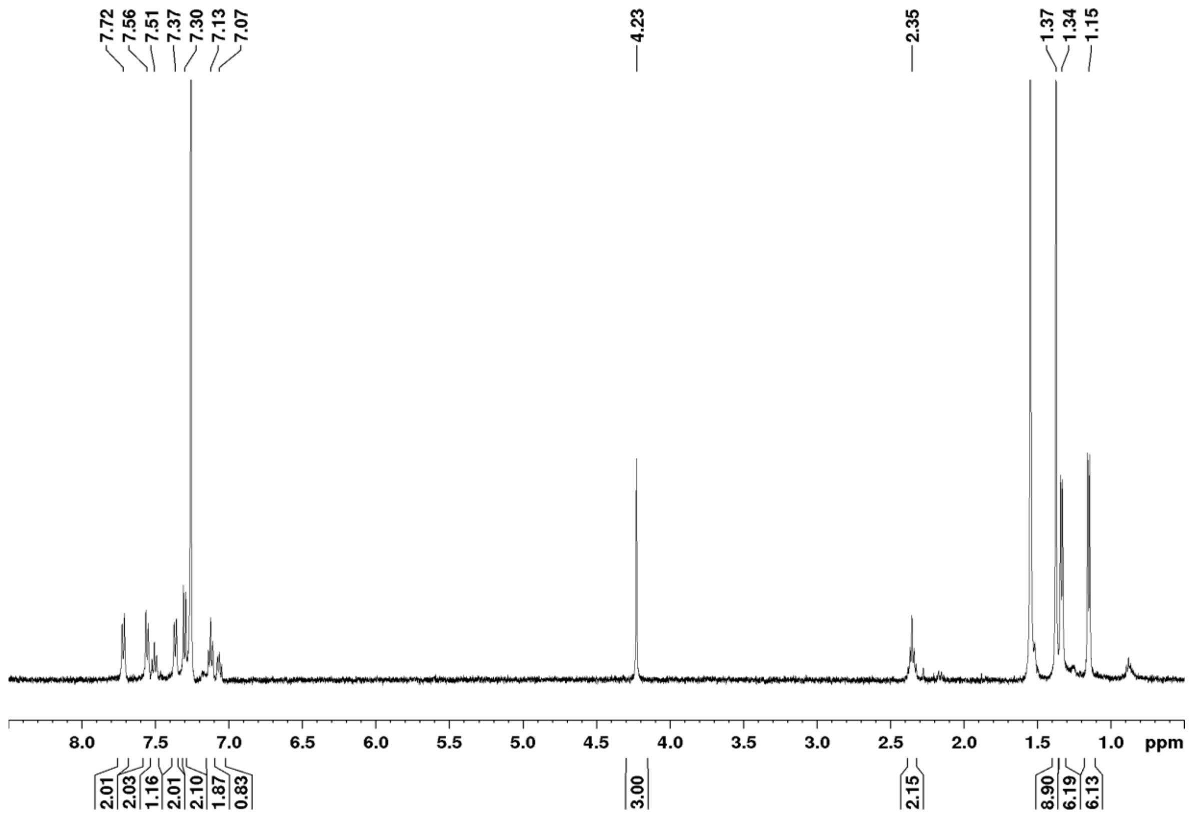

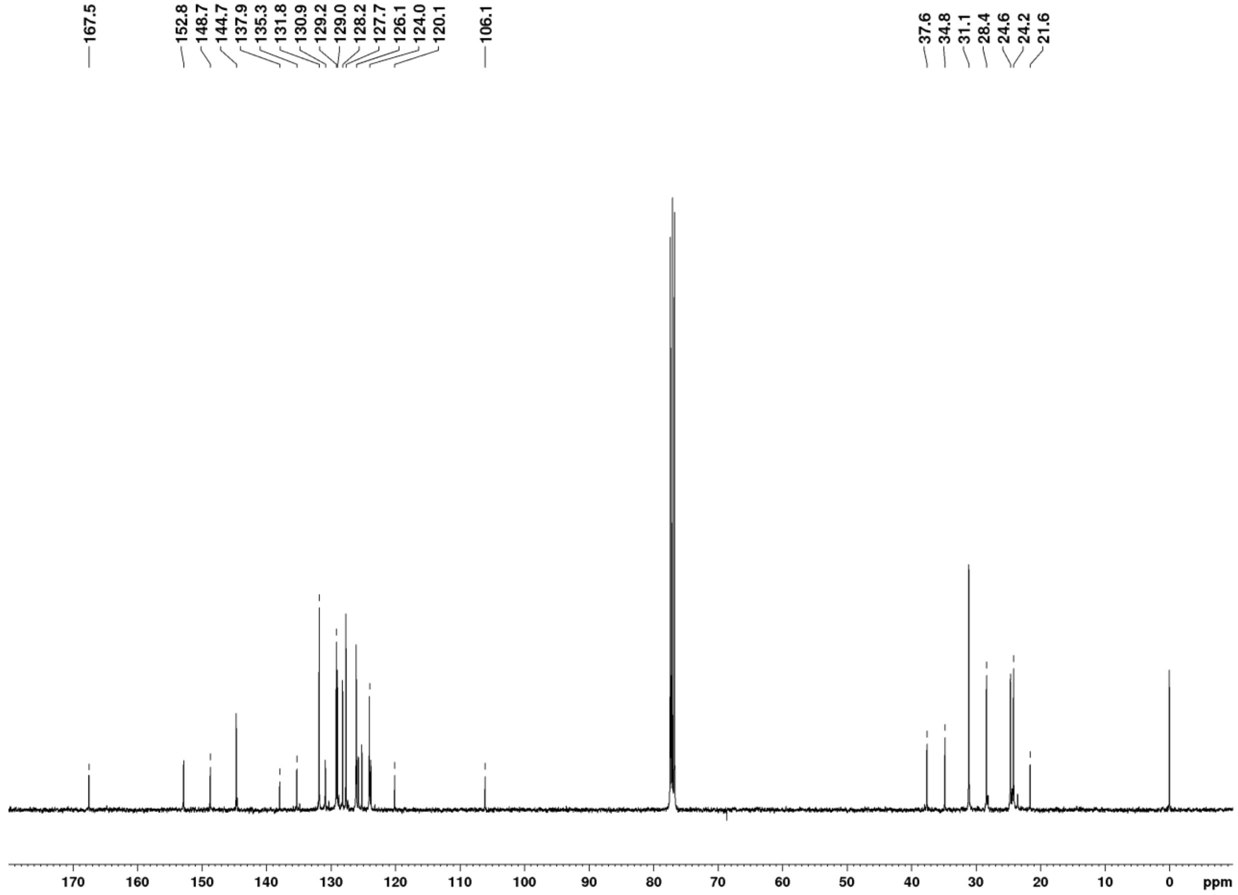

5.3. Stoichiometric reaction 2: tosyl azide and diisopropylamine

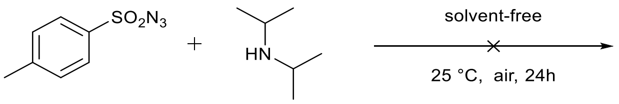

A vial was charged, in air, with tosyl azide (92 µL, 0.6 mmol) and diisopropylamine (85 µL, 0.6 mmol). The reaction mixture was stirred at room temperature for 24 hours under solvent-free conditions. Then, *D*-chloroform was added to the reaction mixture, which was analysed by ^{1}H-NMR spectroscopy. Only starting materials were observed.

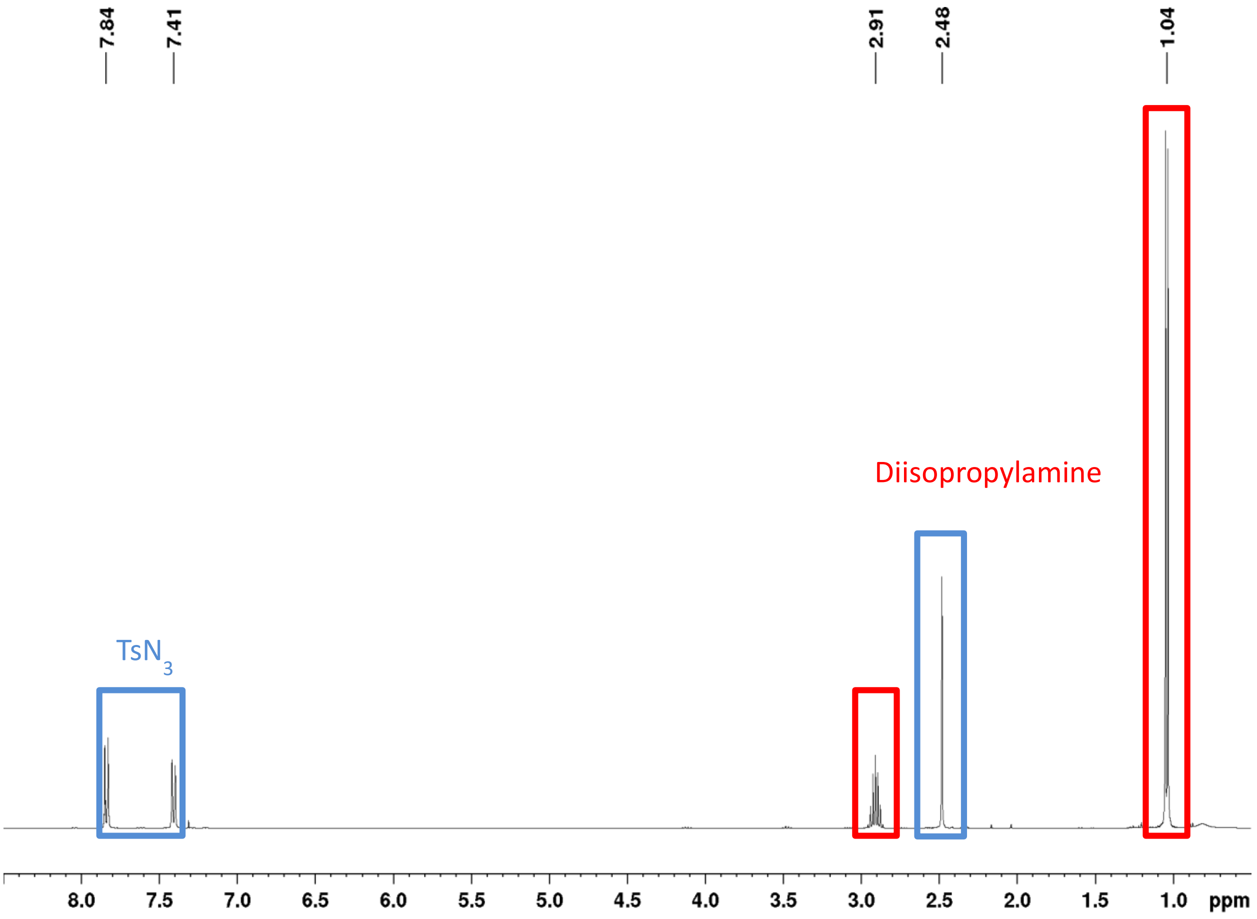

5.4. Stoichiometric reaction 3: $[Cu(Triaz)_2]BF_4$, tosyl azide and diisopropylamine

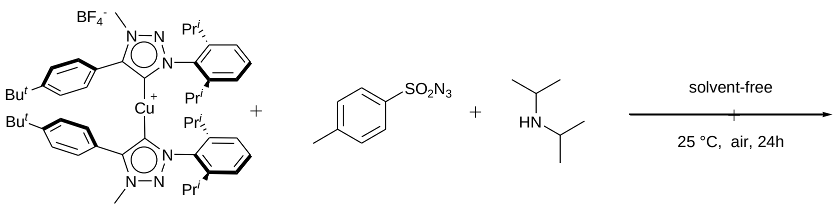

A vial was charged with $[Cu(Triaz)_2]BF_4$ (18 mg, 0.02 mmol), tosyl azide (3.1 µL, 0.02 mmol) and diisopropylamine (2.8 µL, 0.02 mmol). The reaction mixture was stirred at room temperature for 24 hours under solvent-free conditions. Then, *D*-chloroform was added to the reaction mixture, which was analysed by ^{1}H-NMR spectroscopy. Only starting materials were observed.

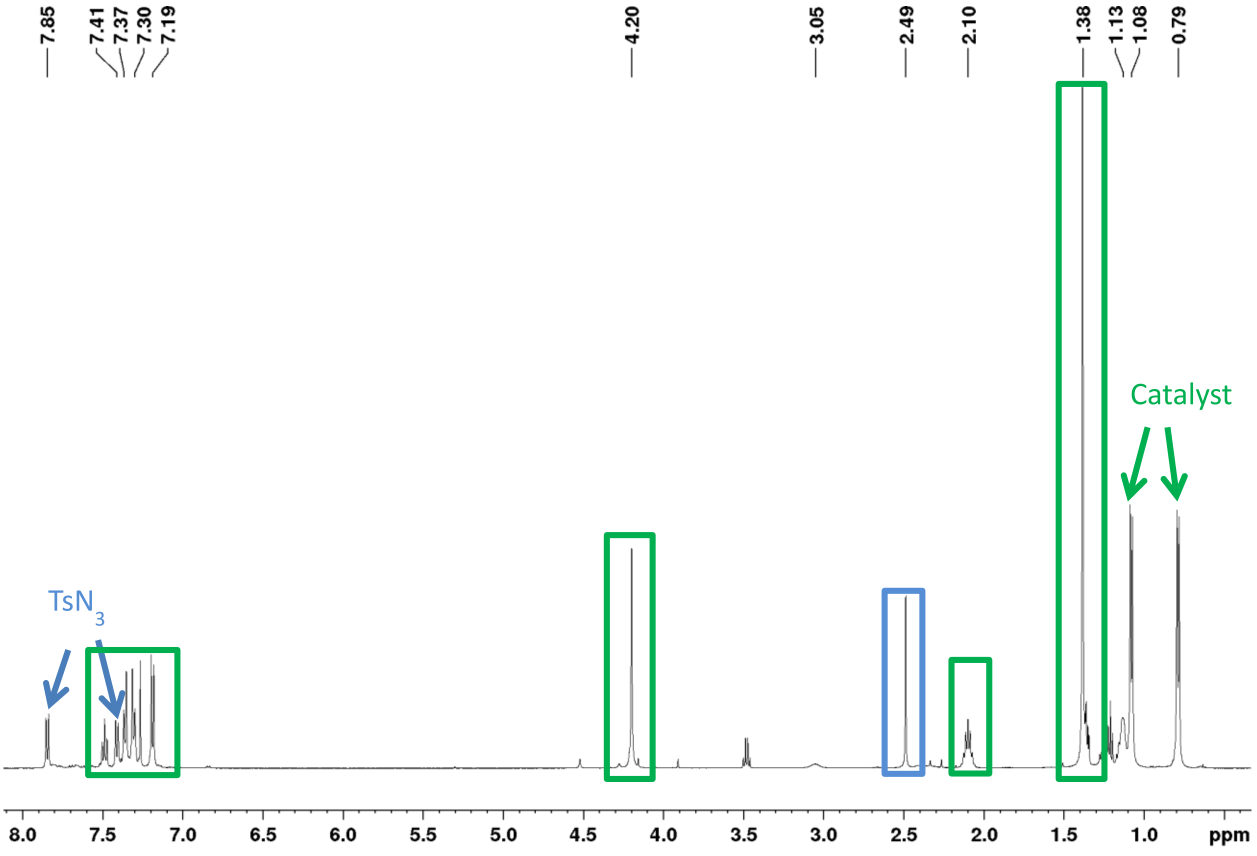

5.5. Stoichiometric reaction 4: Phenylacetylene and diisopropylamine

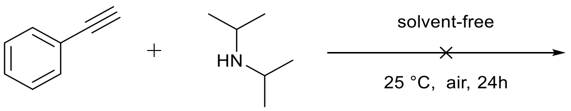

A vial was charged, in air, with phenylacetylene (54.8 µL, 0.6 mmol) and diisopropylamine (85 µL, 0.6 mmol). The reaction mixture was stirred at room temperature for 24 hours under solvent-free conditions. Then, *D*-chloroform was added to the mixture, which was analysed by ^{1}H-NMR spectroscopy. Only starting materials were observed.

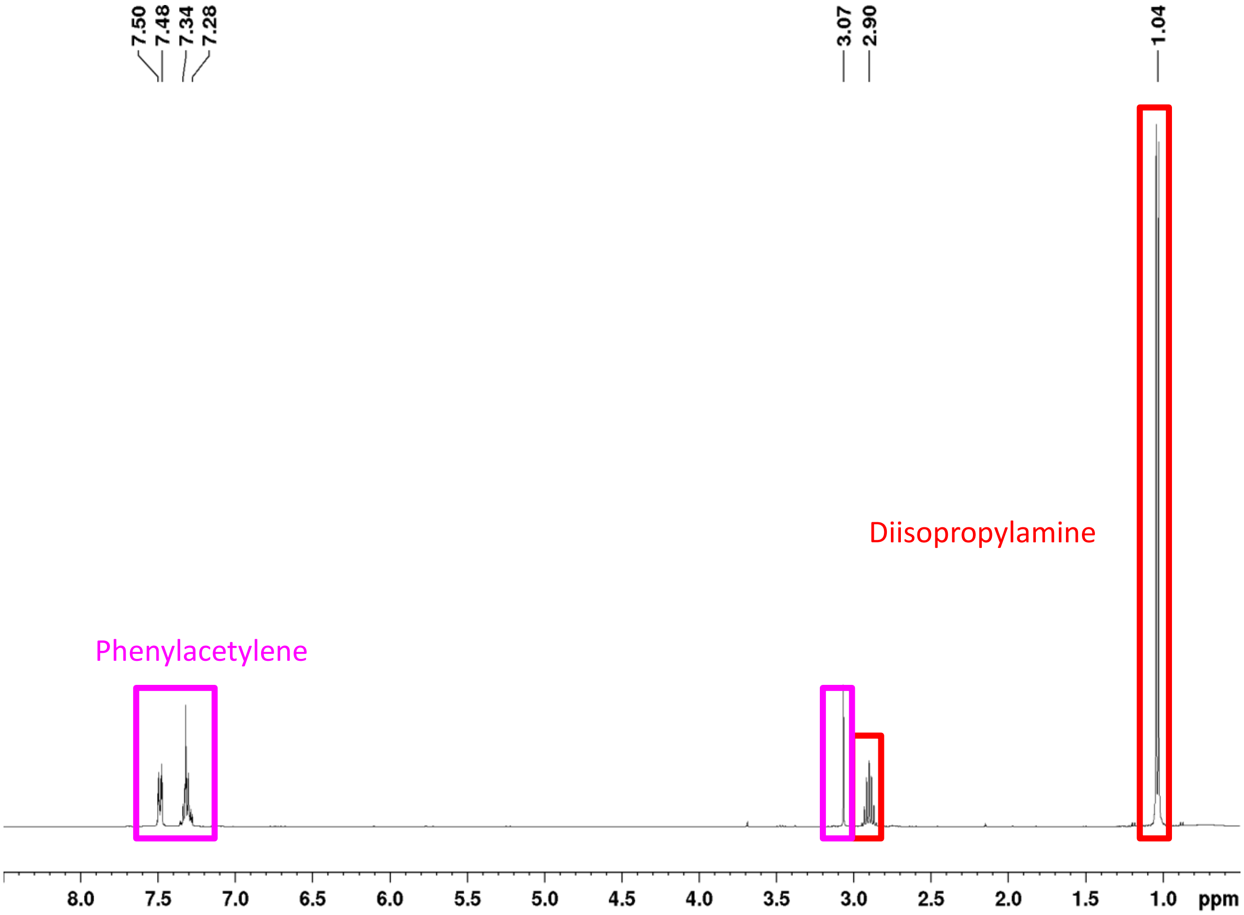

5.6. Stoichiometric reaction 5: $[Cu(Triaz)_2]BF_4$, phenylacetylene and diisopropylamine

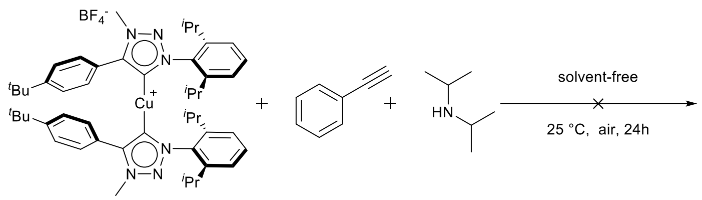

A vial was charged with $[Cu(Triaz)_2]BF_4$ (18 mg, 0.02 mmol), phenylacetylene (2.2 μL, 0.02 mmol) and diisopropylamine (2.8 μL, 0.02 mmol). The reaction mixture was stirred at room temperature for 24 hours neat. Then, *D*-chloroform was added to the reaction mixture, which was analysed by ^1H-NMR spectroscopy. The reaction led to the formation of the Cu-acetylide species and the $TriazHBF_4$ salt with a distinctive peak at 8.34 ppm. Starting materials were also observed.

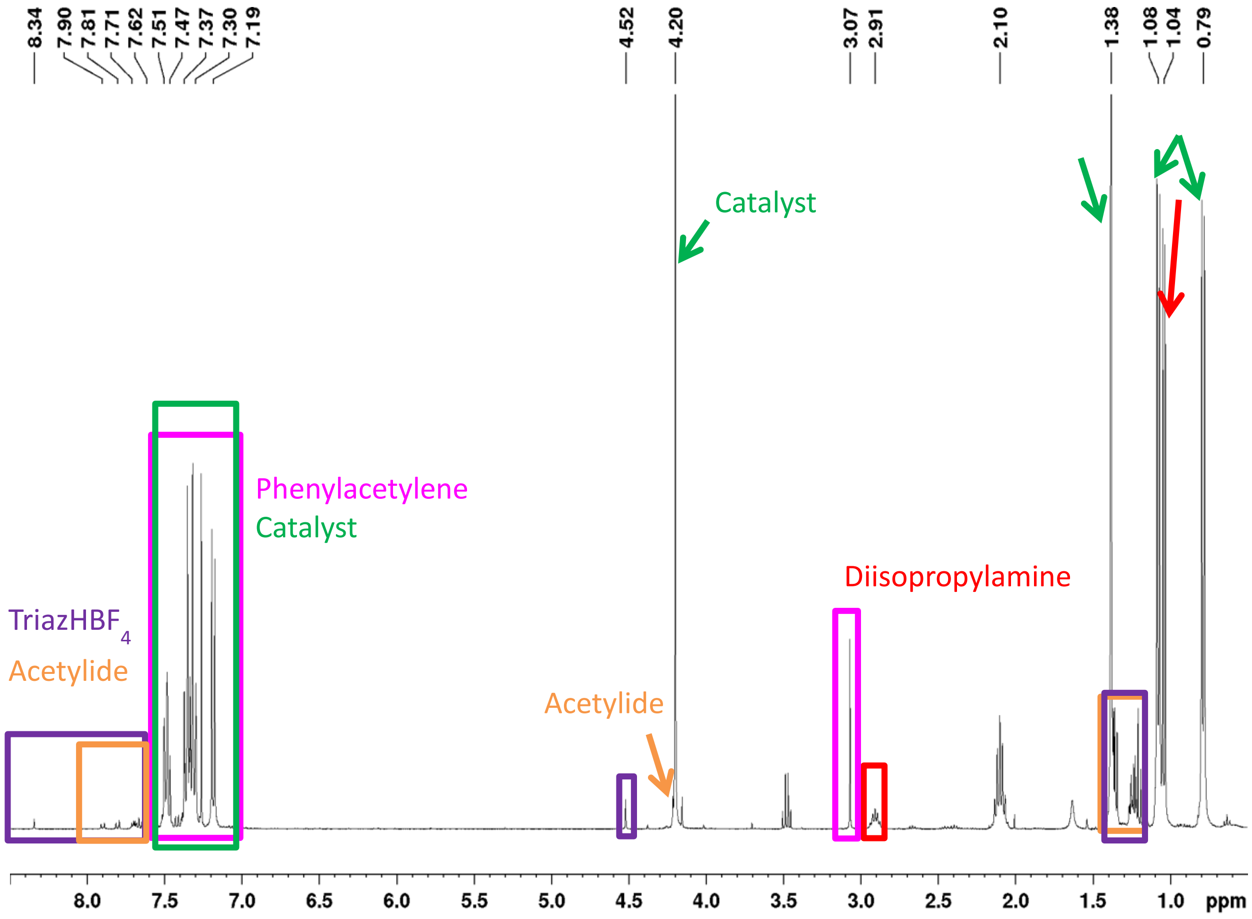

5.7. Stoichiometric reaction 6: [Cu(Triaz)(C≡CPh)] and tosyl azide

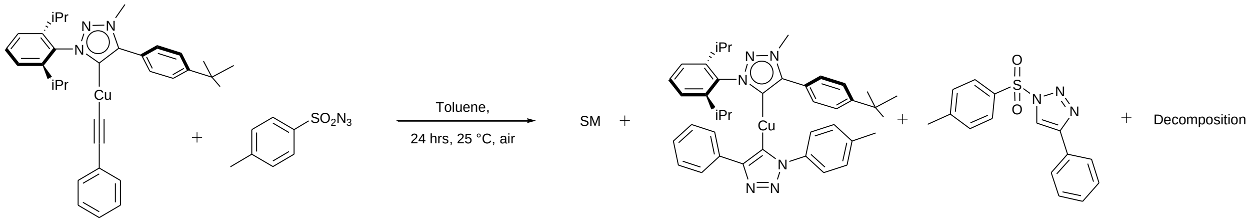

A vial was charged with [Cu(Triaz)(C≡CPh)] (12.5 mg, 0.02 mmol), tosyl azide (3.1 µL, 0.02 mmol) and toluene (1 mL). The mixture was stirred at room temperature for 24 hours in air. Then, toluene was removed under vacuum and *D*-chloroform was added to the reaction mixture, which was analysed by ^{1}H-NMR spectroscopy. The reaction led to the formation of a new species, which might be the highly unstable intermediate [Cu(Triaz)(SO_2Triaz)]. This was proved by the presence of the corresponding sulfonyl triazole released from the decomposition of the species.

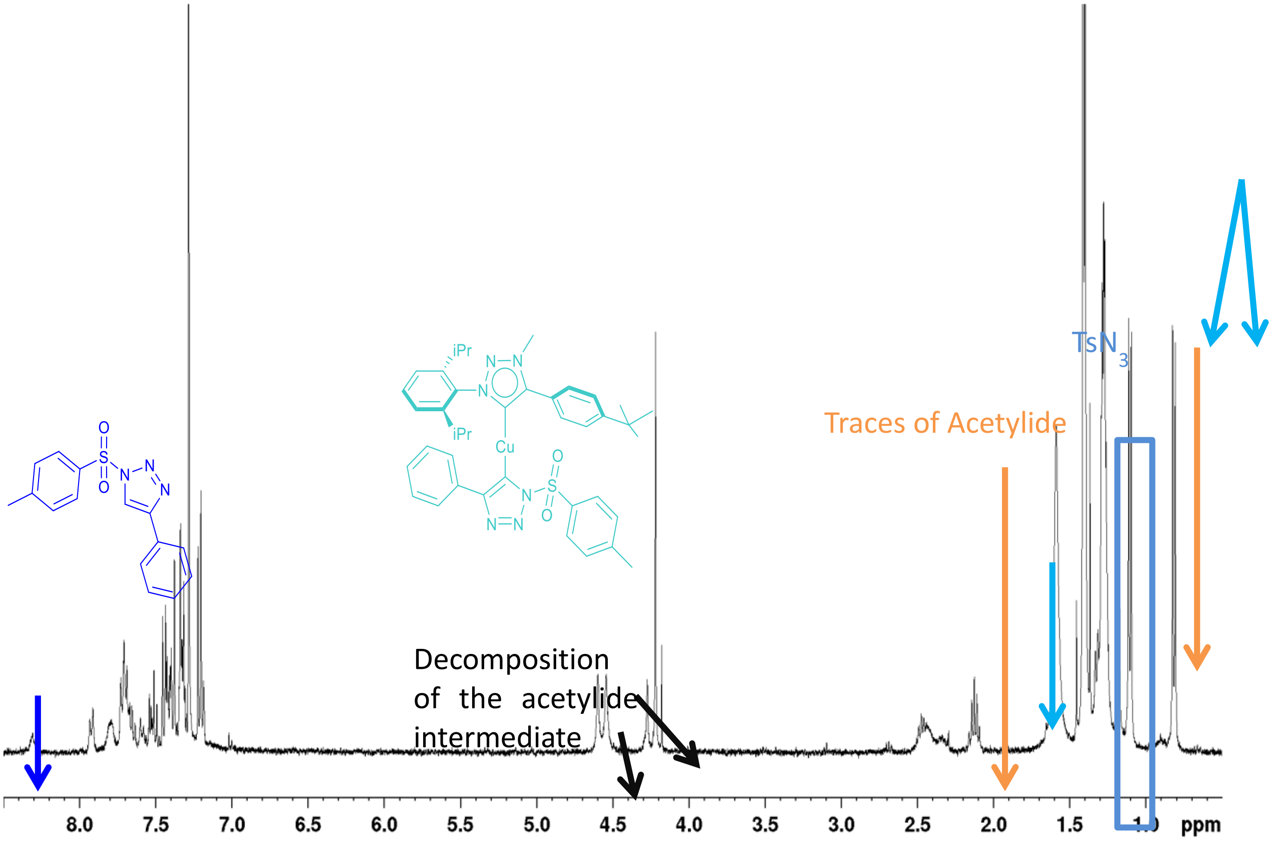

5.8. Stoichiometric reaction 7: [Cu(Triaz)(C≡CPh)], tosyl azide and diisopropylamine

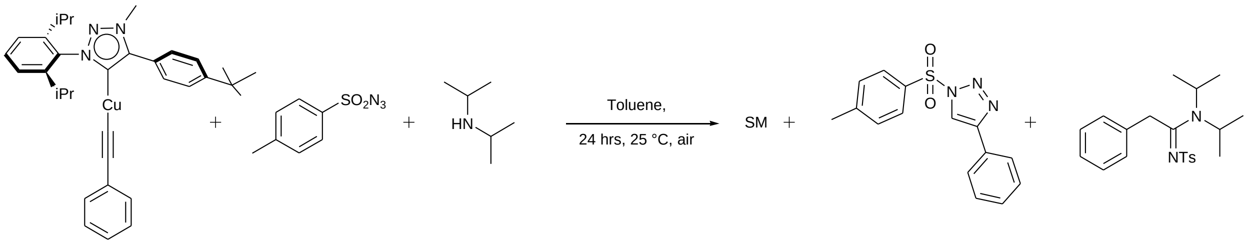

A vial was charged with [Cu(Triaz)(C≡CPh)] (12.5 mg, 0.02 mmol), tosyl azide (3.1 µL, 0.02 mmol), diisopropylamine (2.8 µL, 0.02 mmol) and toluene (1 mL). The mixture was stirred at room temperature for 24 hours in air. Toluene was then removed under vacuum and *D*-chloroform was added to the mixture, which was analysed by ^{1}H-NMR spectroscopy. The reaction led to the formation of the sulfonyl triazole and of the desired product, *N*-sulfonylamidine. Traces of starting materials were also observed.

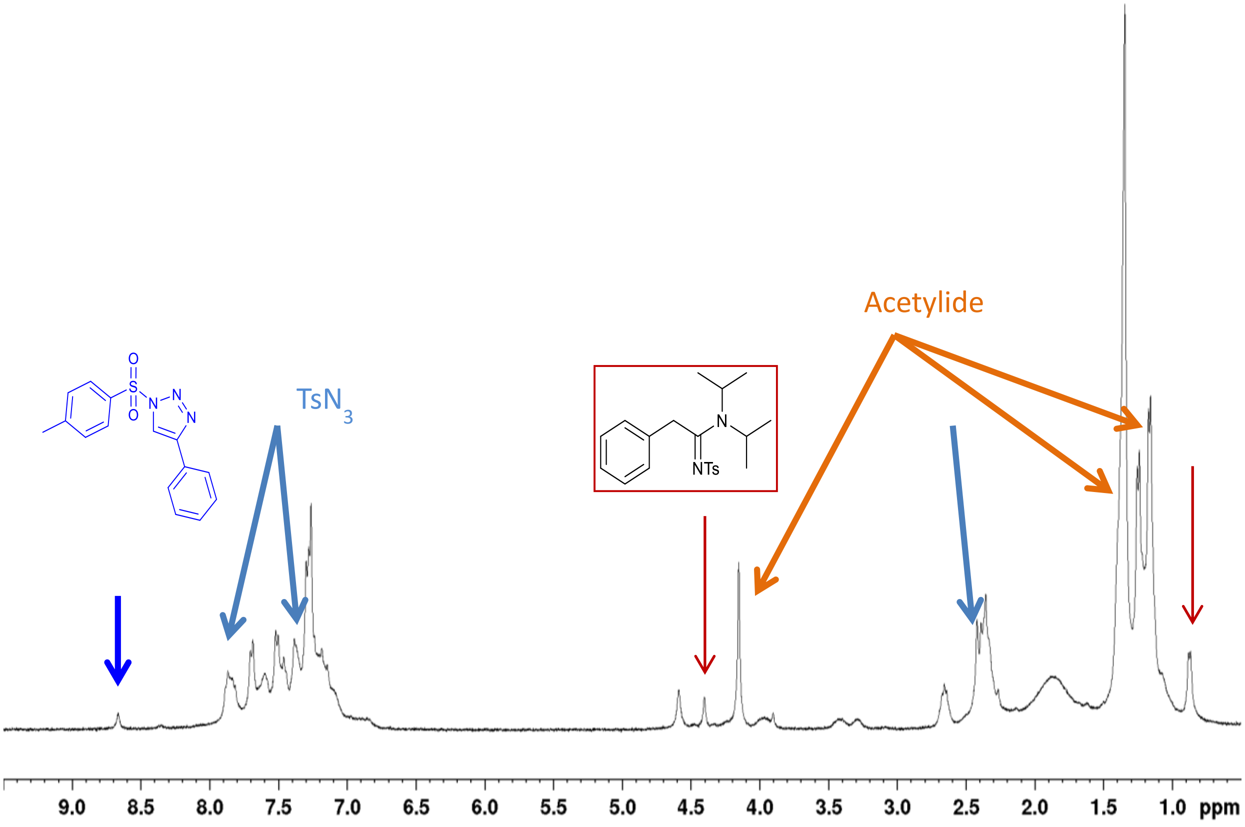

5.9. Stoichiometric reaction 8: [Cu(Triaz)(C≡CPh)], tosyl azide, diisopropylamine and $TriazHBF_4$

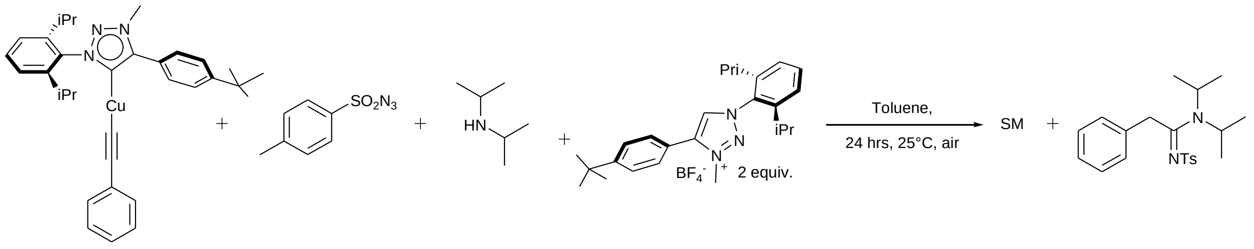

A vial was charged with [Cu(Triaz)(C≡CPh)] (12.5 mg, 0.02 mmol), tosyl azide (3.1 µL, 0.02 mmol), diisopropylamine (2.8 µL, 0.02 mmol), $TriazHBF_4$ (18.6 mg, 0.04 mmol) and toluene (1 mL). The mixture was stirred at room temperature for 24 hours in air. Toluene was then removed under vacuum and *D*-chloroform was added to the reaction mixture, which was analysed by ^{1}H-NMR spectroscopy. The reaction led to the formation of the desired final product, *N*-sulfonylamidine. No sulfonyl triazole was observed despite the presence of remaining starting materials.

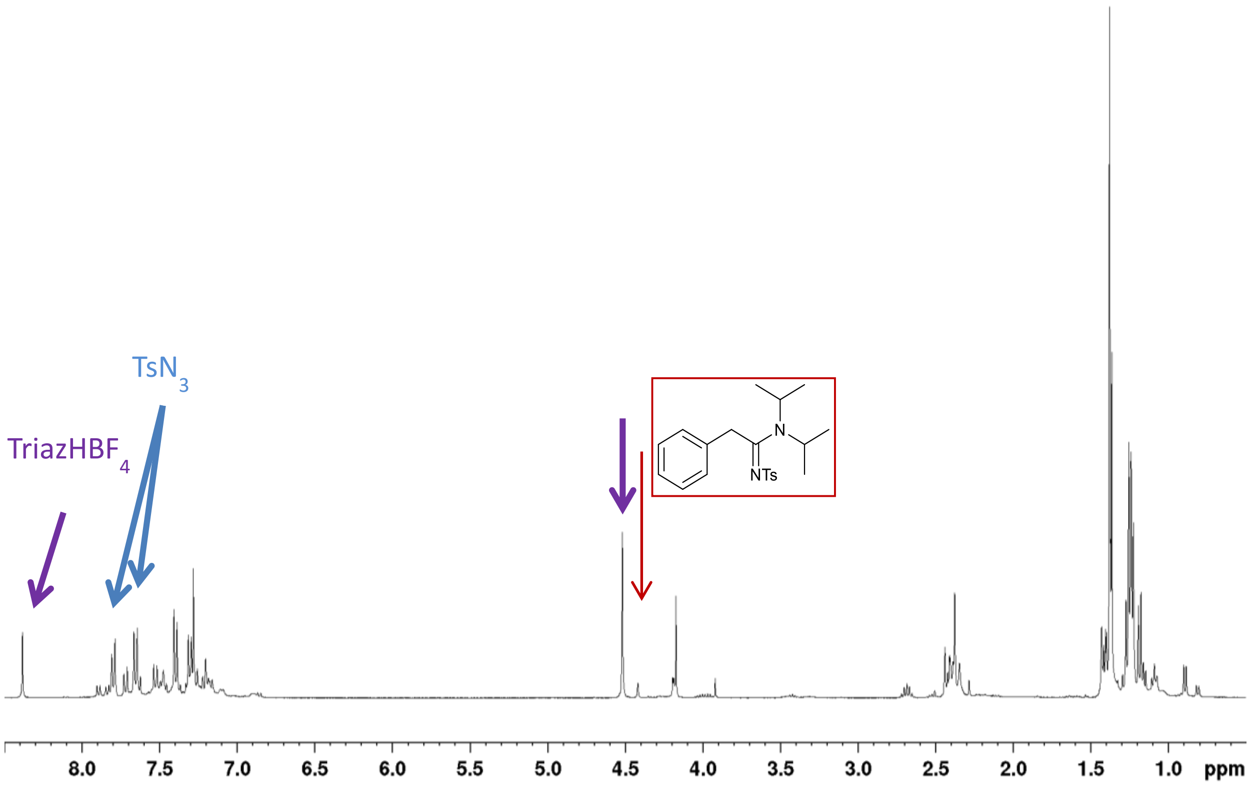

5.10. Catalytic transformation using [Cu(Triaz)(C≡CPh)]

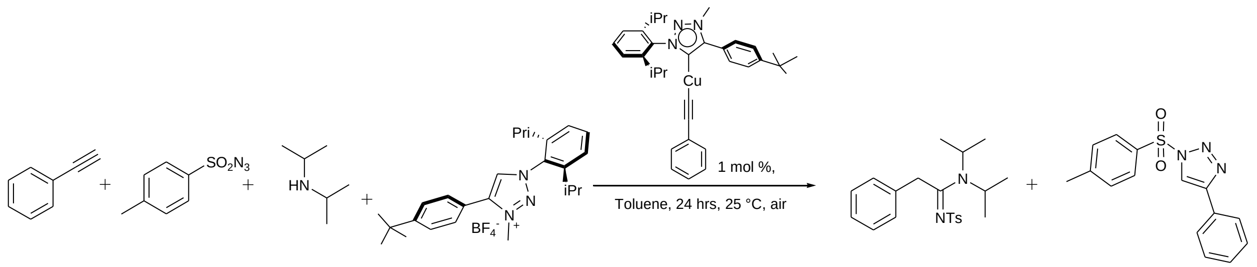

A vial was charged with [Cu(Triaz)(C≡CPh)] (3.13 mg, 0.005 mmol), phenylacetylene (54.8 µL, 0.5 mmol), tosyl azide (92 µL, 0.6 mmol) and diisopropylamine (84 µL, 0.6 mmol). The mixture was stirred at room temperature under solvent-free conditions in air. The reaction was complete after 45 min. Then *D*-chloroform was added to the reaction mixture, which was analysed by ^{1}H-NMR spectroscopy. The reaction led to the formation of the desired product, *N*-sulfonylamidine. A small amount of the sulfonyl triazole was observed as well as diisopropylamine and tosyl azide.

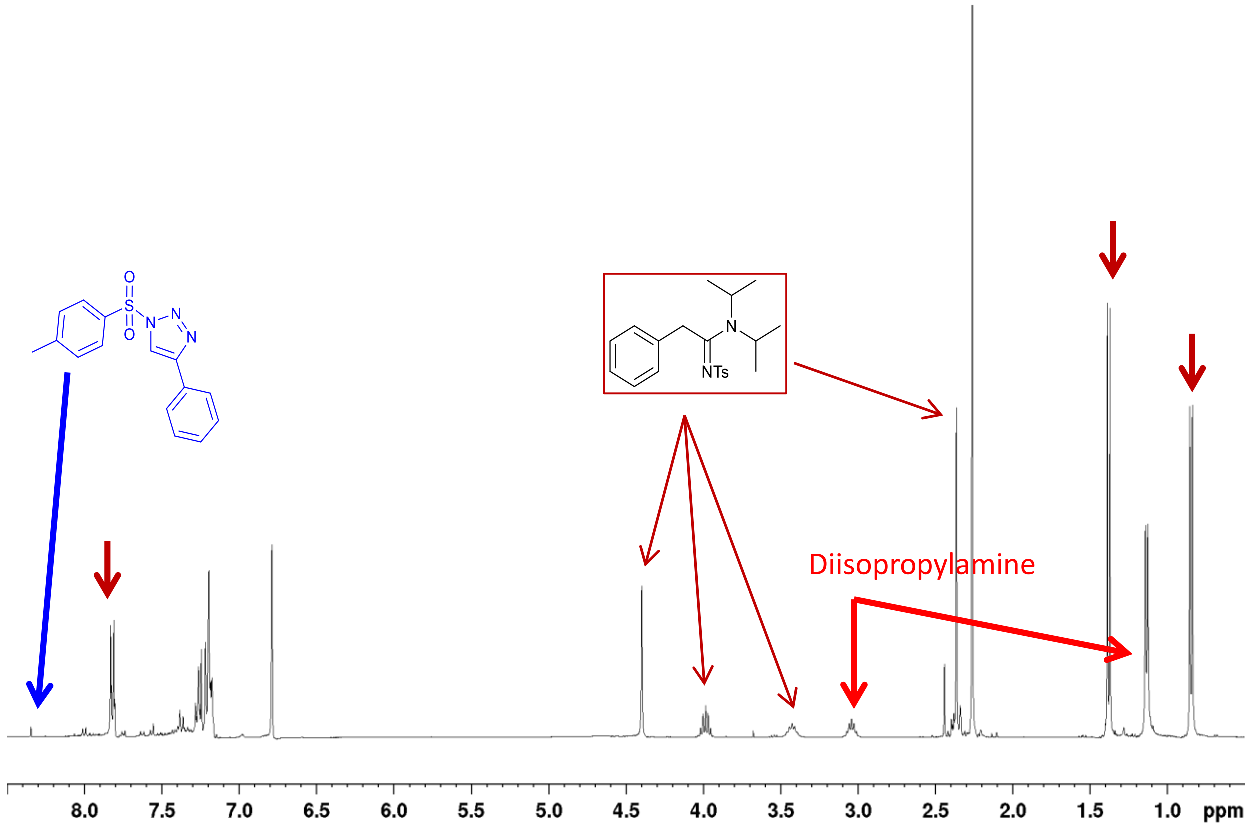

5.11. Stoichiometric benchmark reaction with TriazHBF$_4$ salt

A vial was charged with [Cu(Triaz)$_2$]BF$_4$ (18 mg, 0.005 mmol), phenylacetylene (2.2 µL, 0.02 mmol), tosyl azide (3.1 µL, 0.02 mmol), diisopropylamine (2.8 µL, 0.02 mmol), TriazHBF$_4$ (18.5 mg, 0.04 mmol) and toluene (1 mL). The mixture was stirred at room temperature for 24 hours under solvent-free conditions in air. Then, toluene was removed under vacuum and *D*-chloroform was added to the reaction mixture, which was analysed by ^{1}H-NMR spectroscopy. Low conversion towards the desired product, *N*-sulfonylamidine, was observed. The sulfonyl triazole was not observed, however remaining starting materials, such as TriazHBF$_4$, tosyl azide and phenylacetylene, were recorded. Diisopropylamine was removed during the evaporation step.

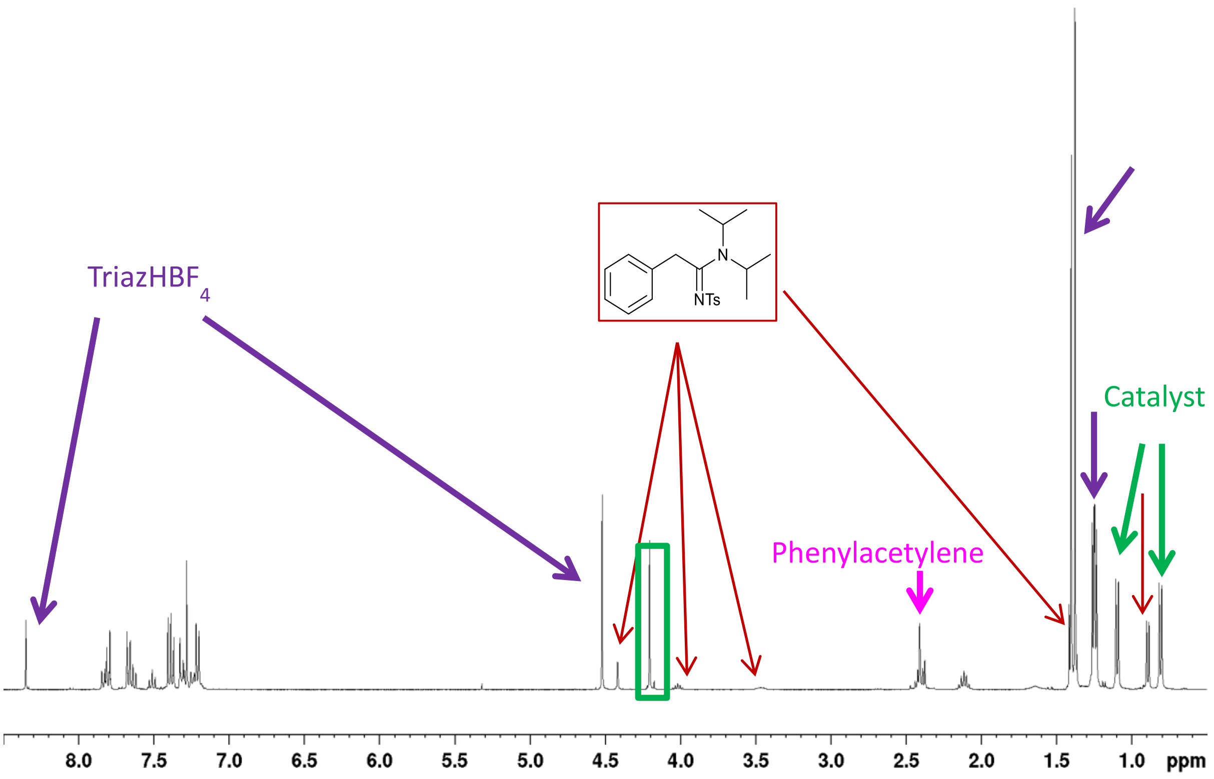

5.12. Synthesis of the sulfonyl triazole, 1-(4-methylphenylsulfonyl)-4-phenyl-1*H*-1,2,3-triazole

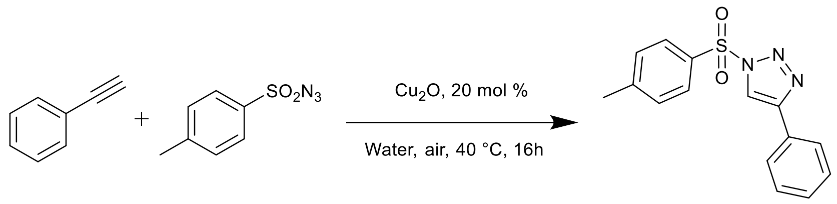

A vial was charged with copper (I) oxide (28 mg, 0.20 mmol), phenylacetylene (109.6 µL, 1 mmol), tosyl azide (184 µL, 1 mmol) and water (1 mL). The mixture was stirred for 16 hours at 40 °C. Dichloromethane was then added to the reaction mixture. The organic layer was filtered through a plug of celite and washed with dichloromethane. The solvent was removed under vacuum and acetone/pentane was added. The product was collected by filtration as a colourless solid (250 mg, 84 % isolated yield).

^{1}H NMR (400 MHz, $CDCl_3$, 298K, TMS): δ (ppm) = 2.45 (s, 3H, Ar-CH_3), 7.35-7.45 (m, 5H, $C_{Ar}H$), 7.81-7.84 (m, 2H, $C_{Ar}H$), 8.02-8.05 (m, 2H, $C_{Ar}H$), 8.31 (s, 1H, N-C*H*).

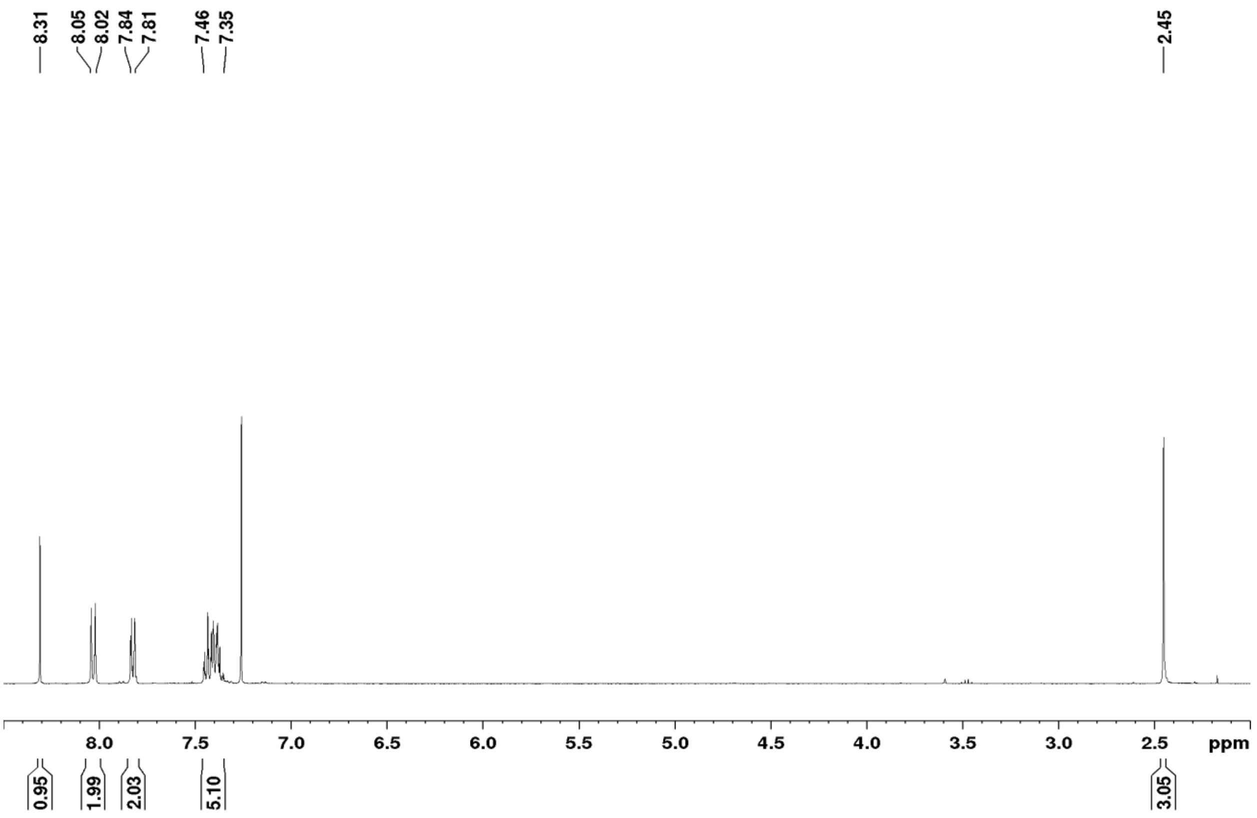

5.13. Stoichiometric reaction 9: $[Cu(Triaz)_2]BF_4$ and 1-(4-methylphenylsulfonyl)-4-phenyl-1*H*-1,2,3-triazole

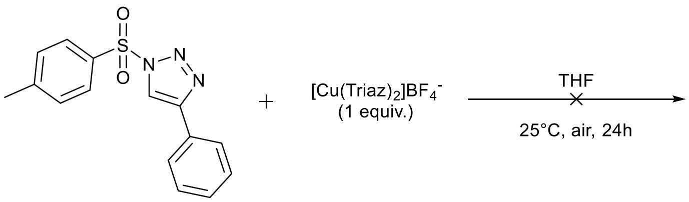

A vial was charged, in air, with the catalyst, $[Cu(Triaz)_2]BF_4$ (18.0 mg, 0.02 mmol), the sulfonyl triazole (6.0 mg, 0.02 mmol) and THF (1 mL). The mixture was stirred for 24 hours at room temperature. THF was then removed and *D*-chloroform was added to the reaction mixture, which was analysed by ^{1}H-NMR spectroscopy. No new products were observed.

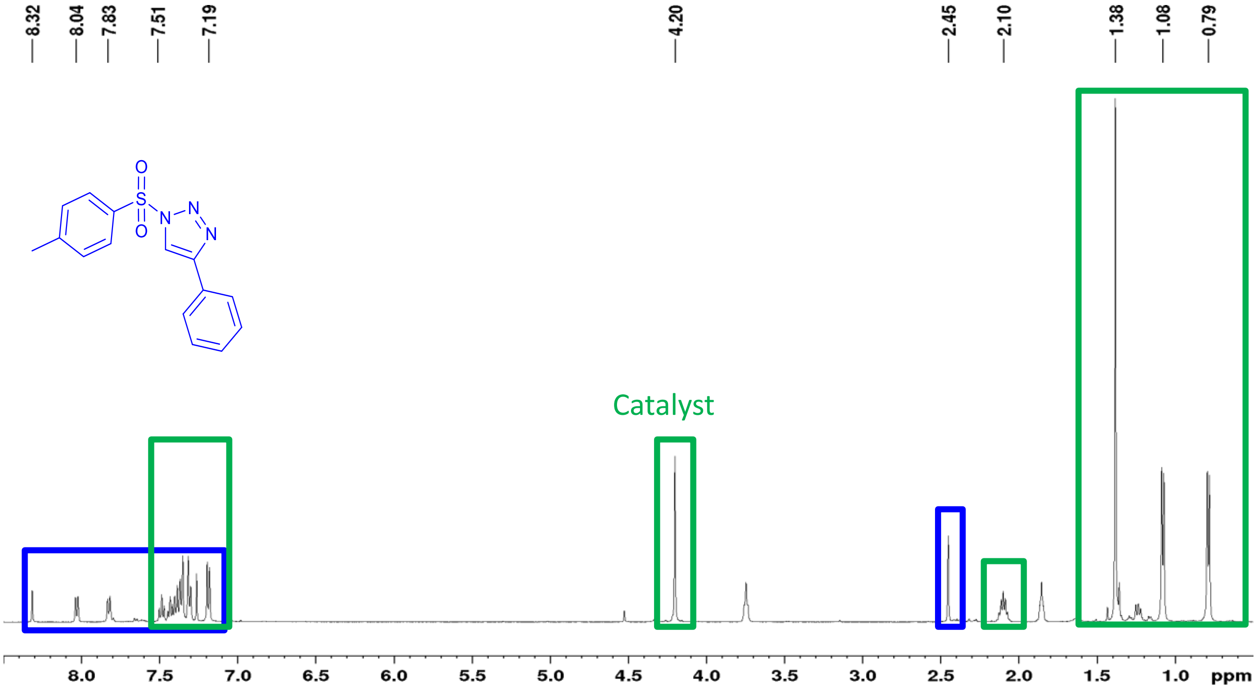

5.14. Stoichiometric reaction 10: 1-(4-methylphenylsulfonyl)-4-phenyl-1*H*-1,2,3-triazole and diisopropylamine

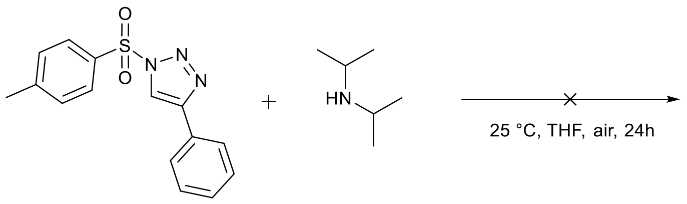

In air, a vial was charged with the sulfonyl triazole (12.0 mg, 0.04 mmol), diisopropylamine (5.6 μL, 0.04 mmol) and THF (1 mL). The mixture was stirred at room temperature for 24 hours. THF was then removed under vacuum and *D*-chloroform was added to the reaction mixture, which was analysed by ^{1}H-NMR spectroscopy. Only starting materials were observed.

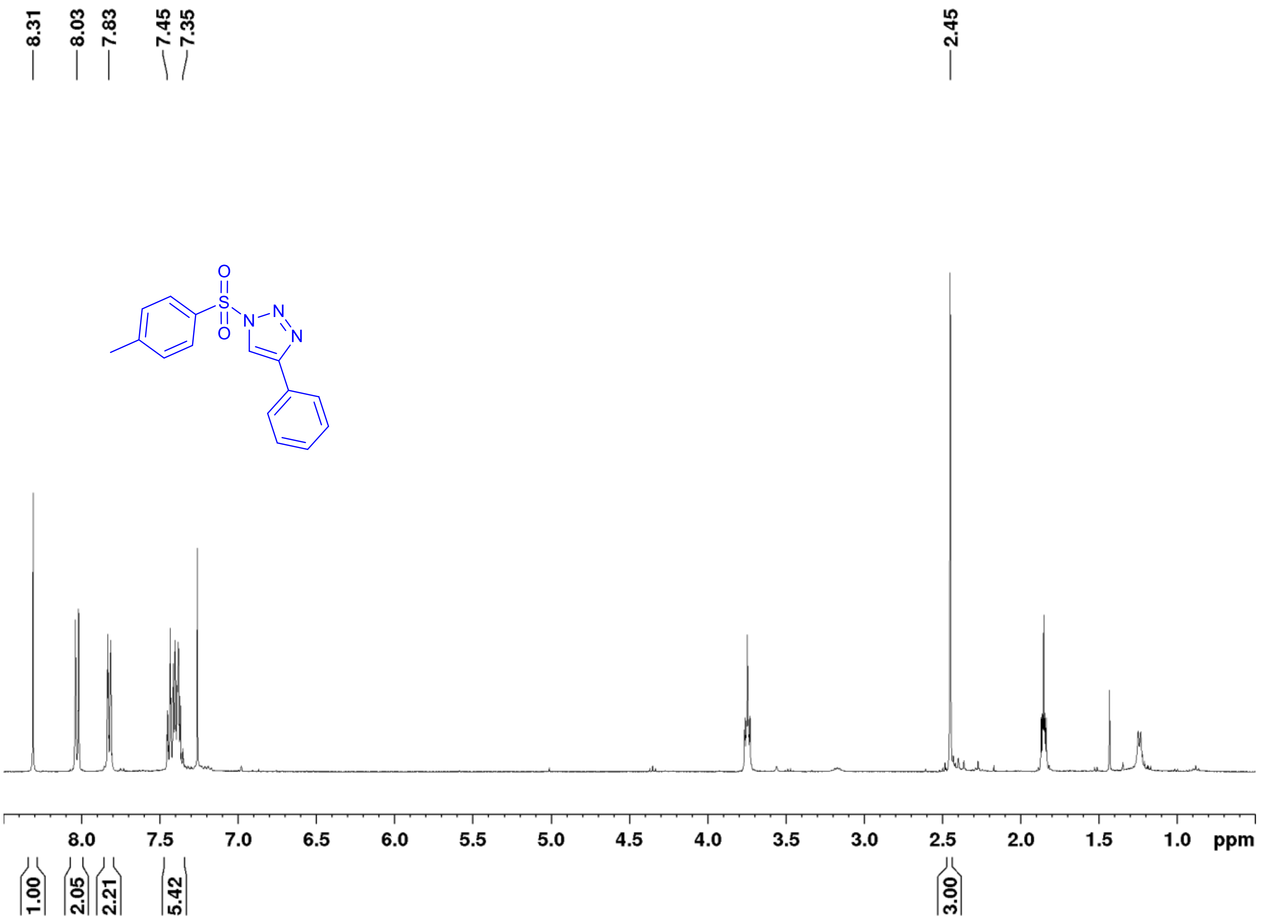

5.15. Stoichiometric reaction 11: $[Cu(Triaz)_2]BF_4$, 1-(4-methylphenylsulfonyl)-4-phenyl-1*H*-1,2,3-triazole and diisopropylamine

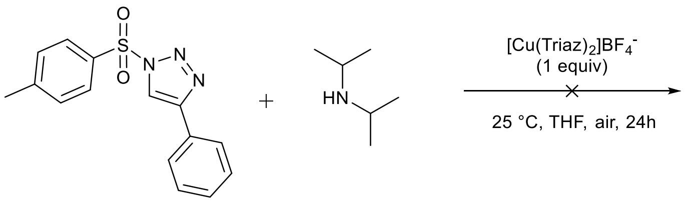

In air, a vial was charged with the catalyst $[Cu(Triaz)_2]BF_4$, the sulfonyl triazole (12.0 mg, 0.04 mmol), diisopropylamine (5.6 μL, 0.04 mmol) and THF (1 mL). The mixture was stirred at room temperature for 24 hours. THF was then removed under vacuum and *D*-chloroform was added to the mixture, which was analysed by ^{1}H-NMR spectroscopy. Only starting materials were observed.

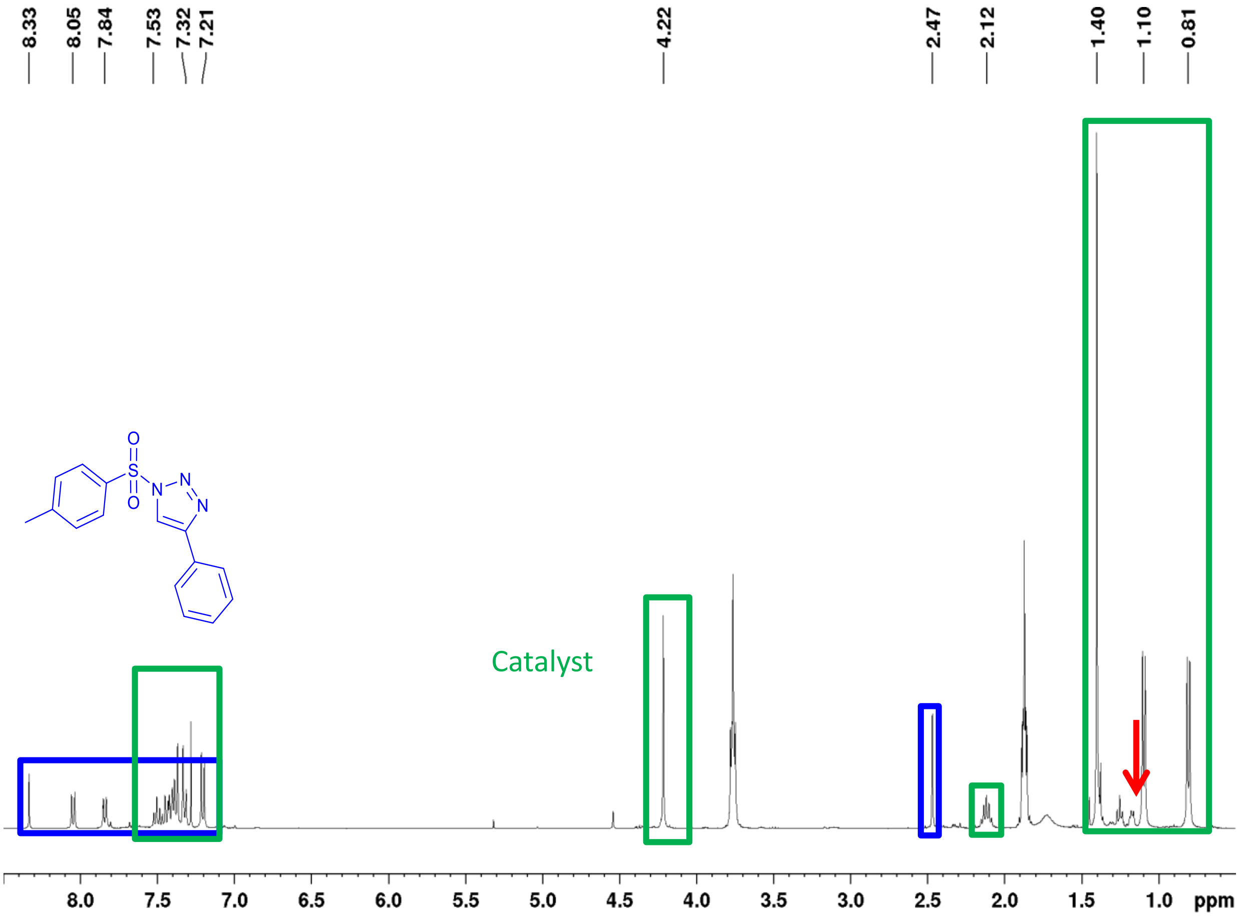

6. NMR Spectra

6.1. NMR Spectra of complexes

***N,N'*-Bis-{2,6-(diisopropyl)phenyl}imidazol-2-ylidene-imidazolidin-2-ylidene pyridine copper(I) triflate, [Cu(IPr)(Py)]OTf (4)**

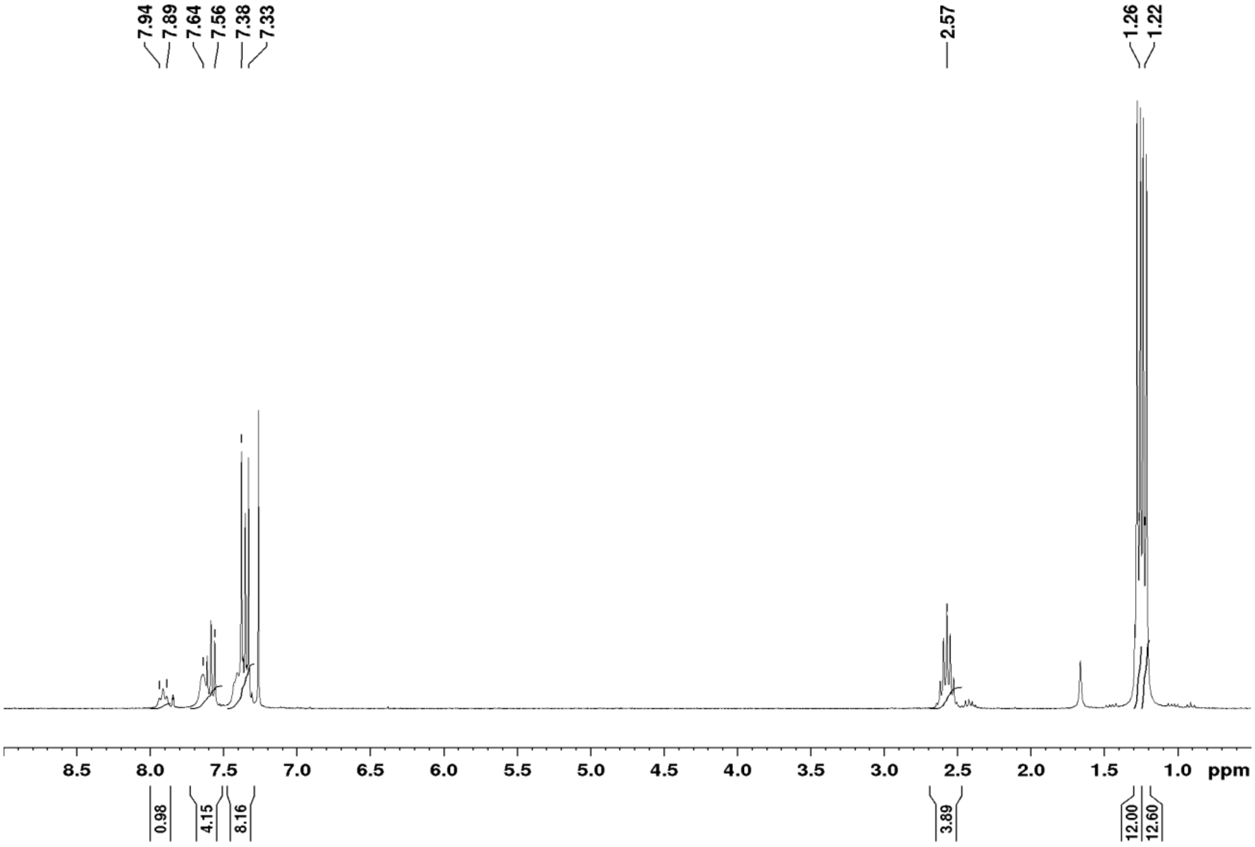

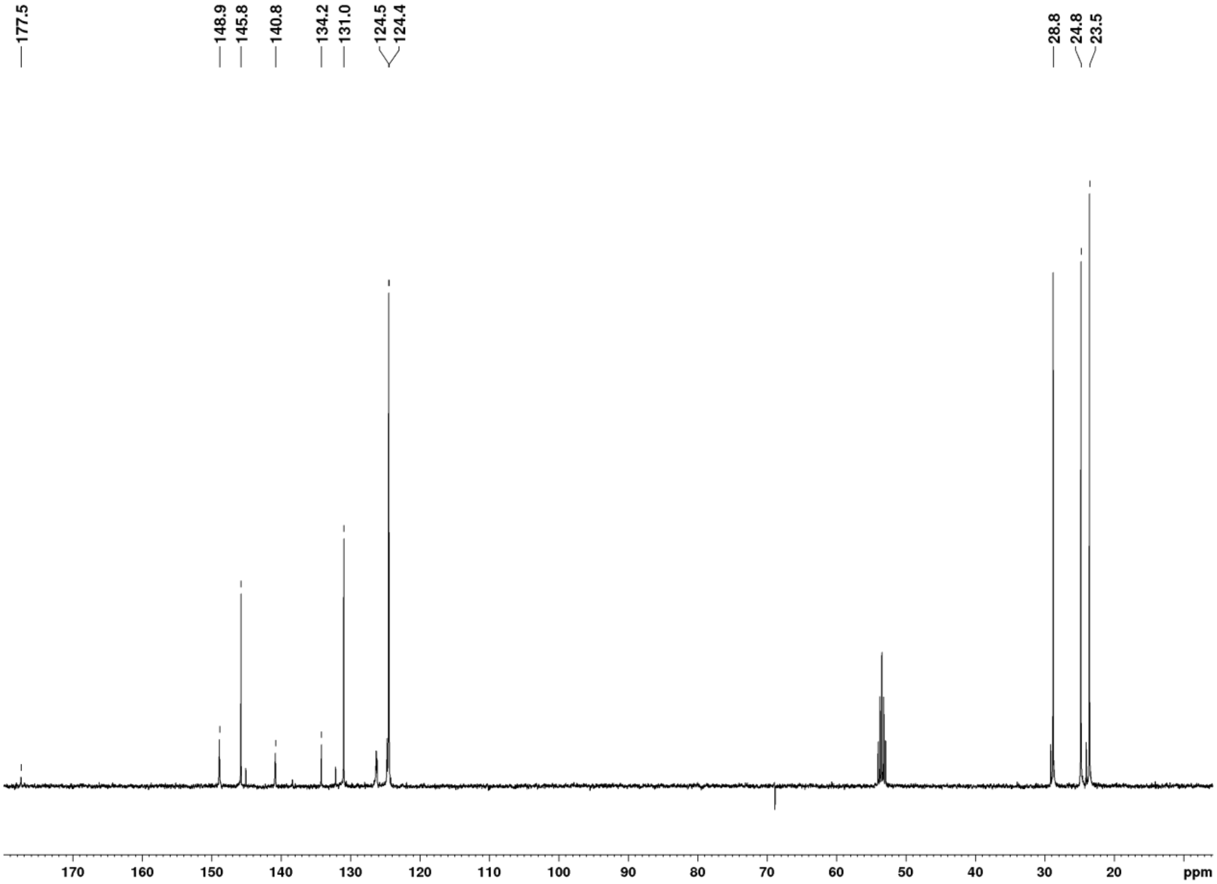
177.5
148.9
145.8
140.8
134.2
131.0
124.5
124.4
28.8
24.8
23.5
170 160 150 140 130 120 110 100 90 80 70 60 50 40 30 20 ppm

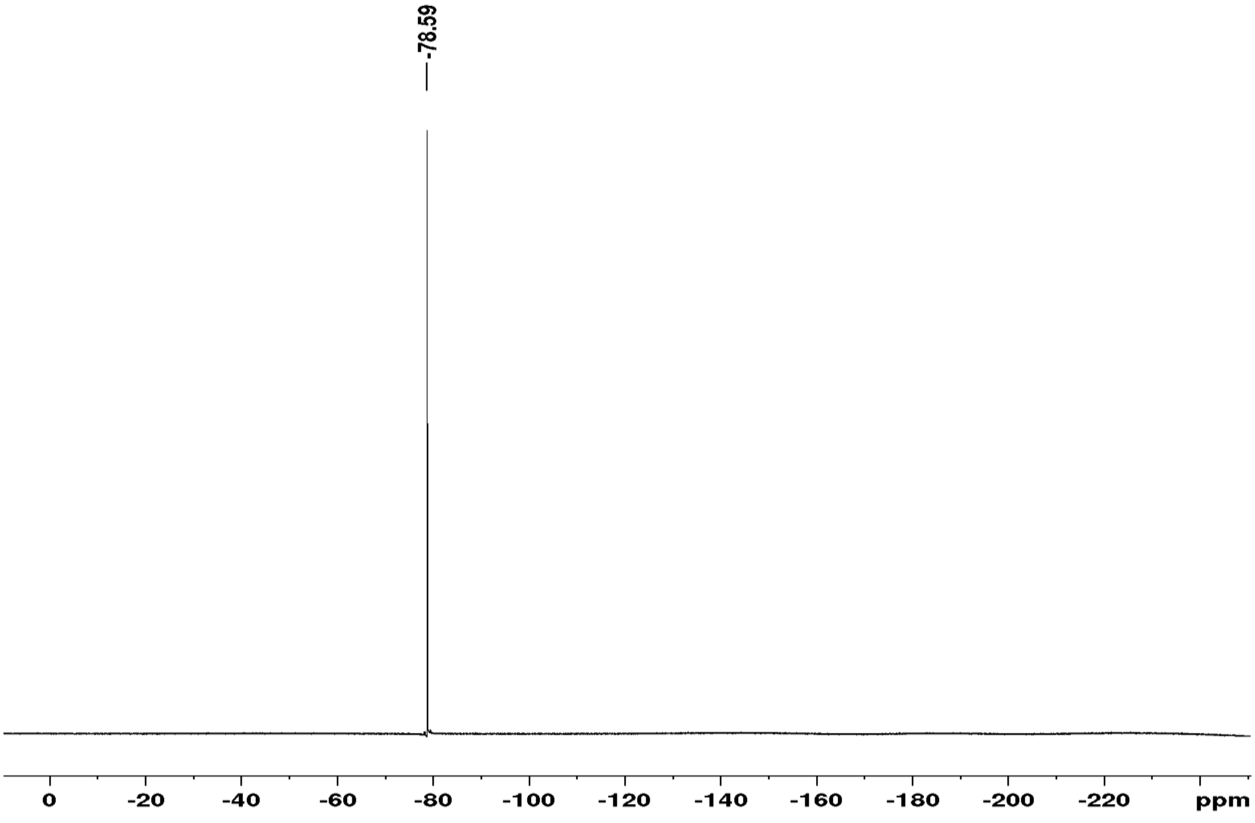
-78.59
0 -20 -40 -60 -80 -100 -120 -140 -160 -180 -200 -220 ppm

1-{2,6-(Diisopropyl)phenyl}-3-methyl-4-(4-*tert*-butylphenyl)-1,2,3-triazol-5-ylidene- *N,N'*-bis-{2,6-(diisopropyl)phenyl}imidazol-2-ylidene- copper(I) tetrafluoroborate, [Cu(IPr)(Triaz)]BF_4 (5)

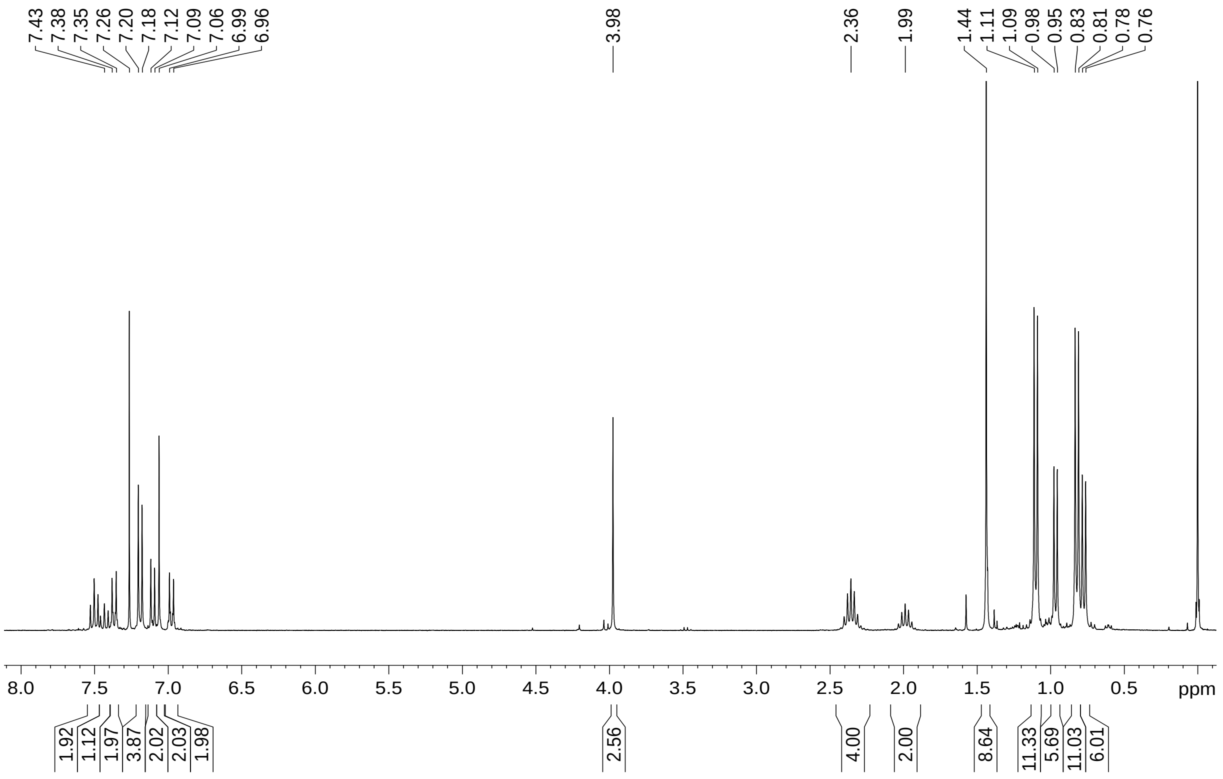

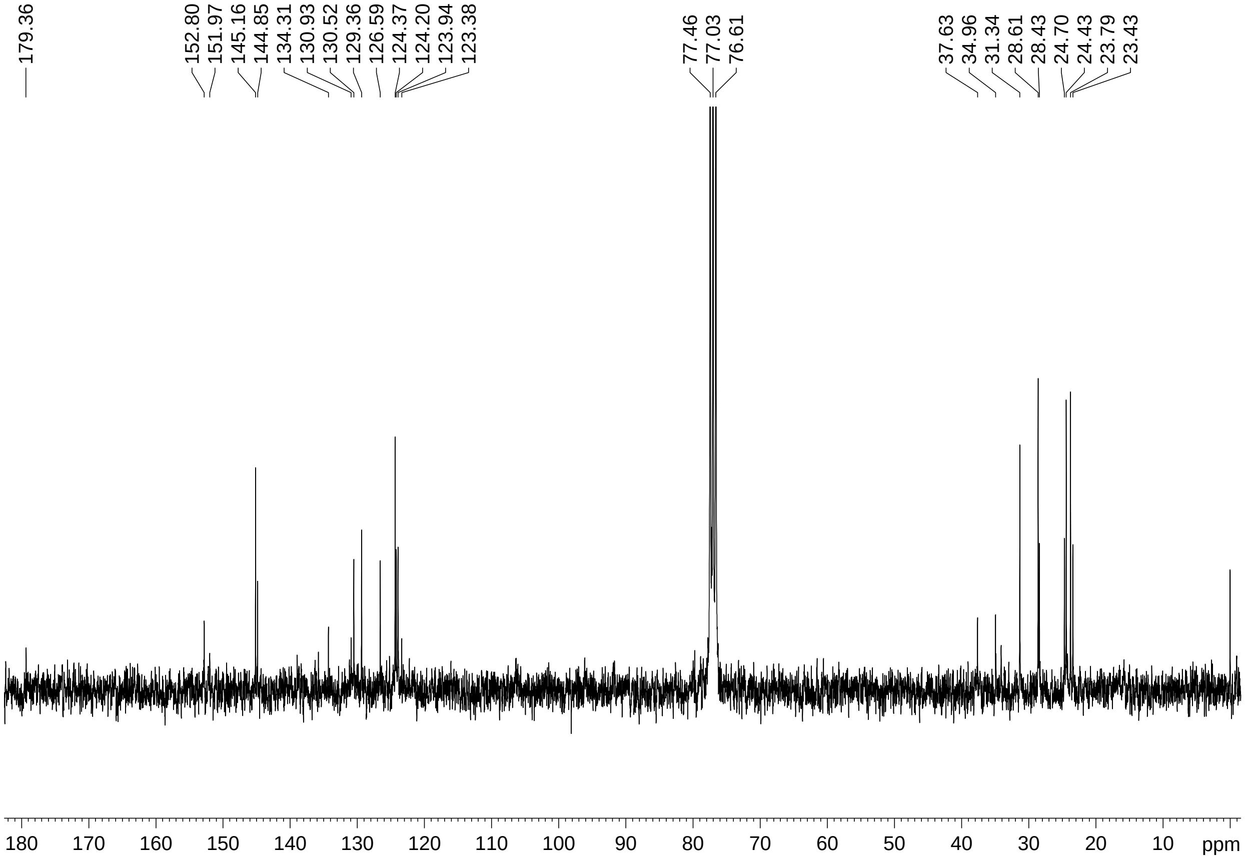
179.36
152.80
151.97
145.16
144.85
134.31
130.93
130.52
129.36
126.59
124.37
124.20
123.94
123.38
77.46
77.03
76.61
37.63
34.96
31.34
28.61
28.43
24.70
24.43
23.79
23.43
180 170 160 150 140 130 120 110 100 90 80 70 60 50 40 30 20 10 ppm

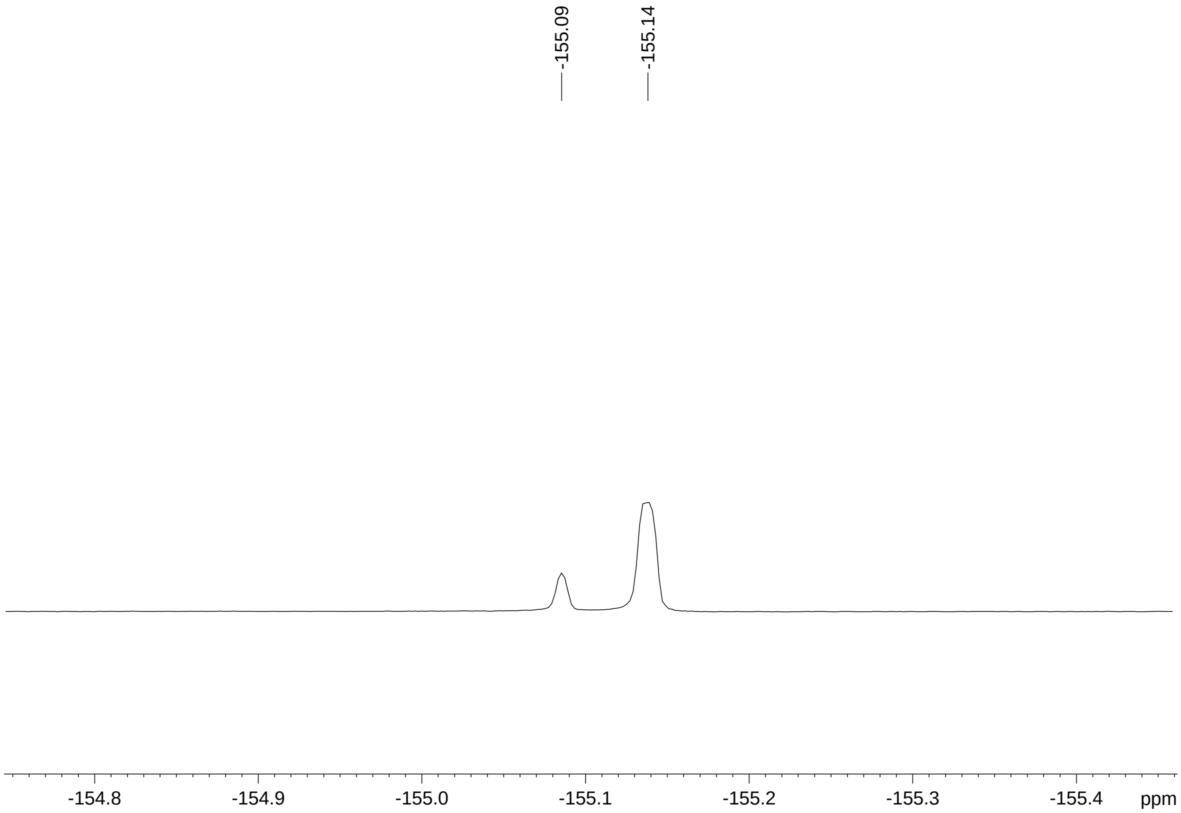
-155.09
-155.14
-154.8 -154.9 -155.0 -155.1 -155.2 -155.3 -155.4 ppm

Di-{1-{2,6-(diisopropyl)phenyl}-3-methyl-4-(4-*tert*-butylphenyl)-1,2,3-triazol-5-ylidene} copper(I) tetrafluoroborate, $[Cu(Triaz)_2]BF_4$ (6)

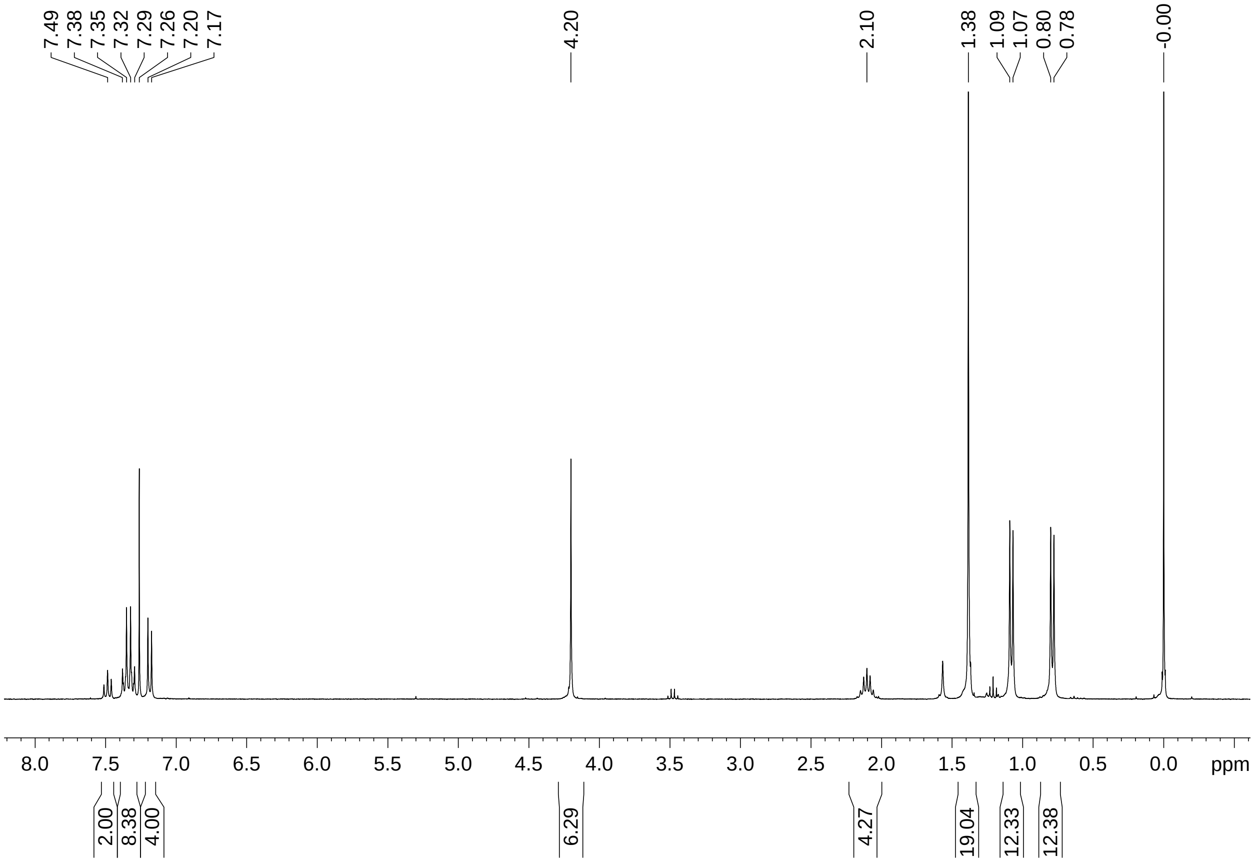

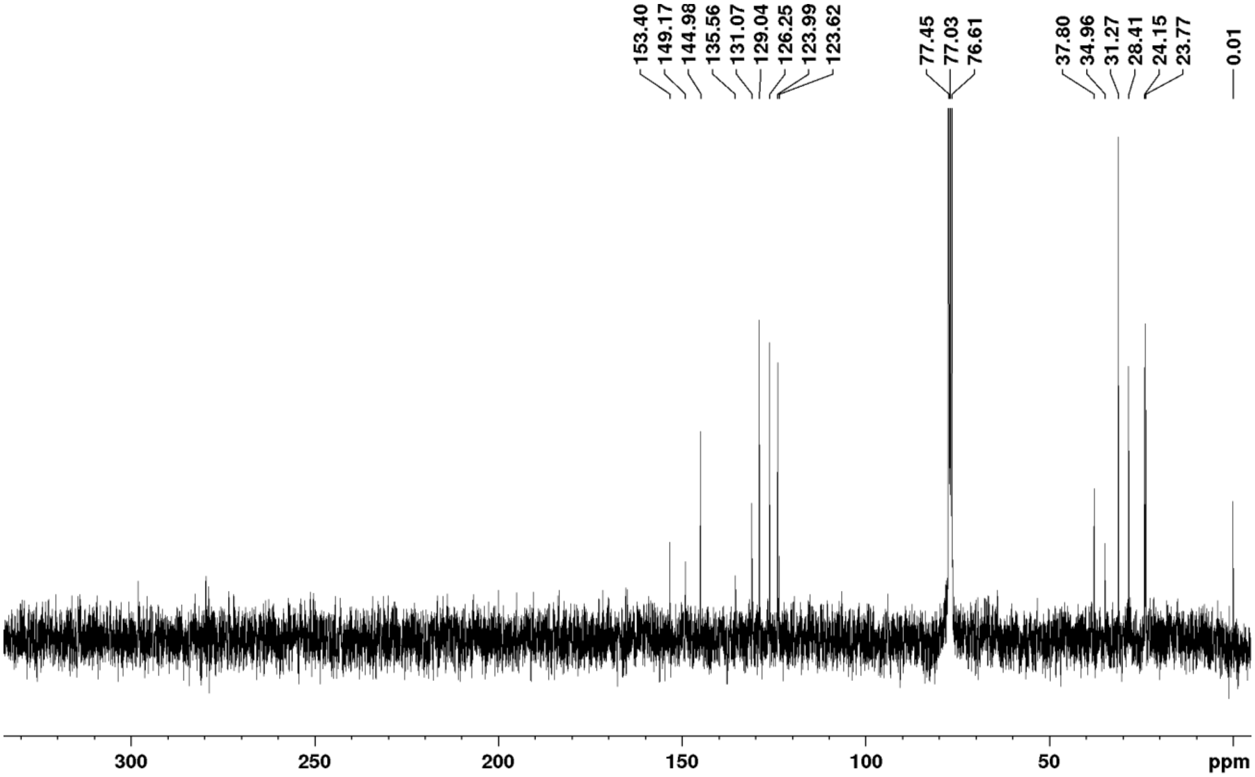
153.40
149.17
144.98
135.56
131.07
129.04
126.25
123.99
123.62
77.45
77.03
76.61
37.80
34.96
31.27
28.41
24.15
23.77
0.01
300
250
200
150
100
50
ppm

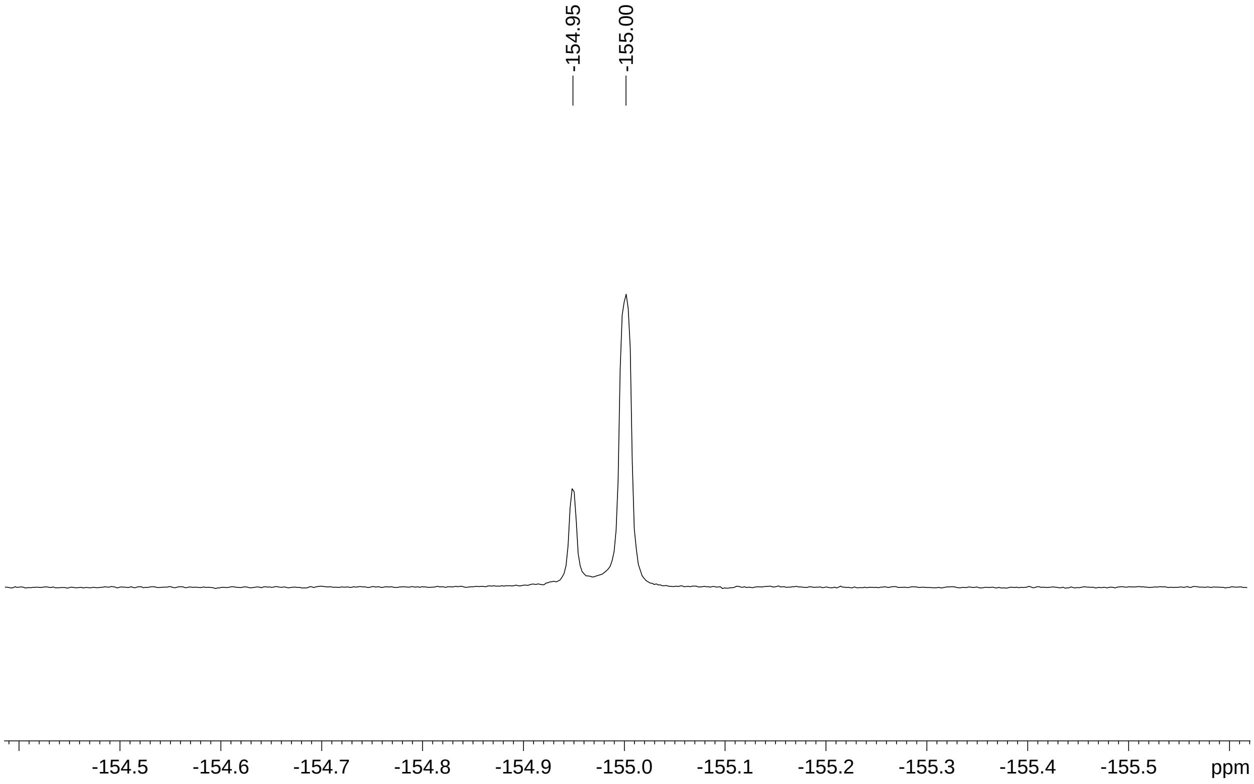
-154.95
-155.00
-154.5
-154.6
-154.7
-154.8
-154.9
-155.0
-155.1
-155.2
-155.3
-155.4
-155.5
ppm

6.2. NMR Spectra of catalysis products

N,N-Diisopropyl-2-phenyl-*N'*-tosylacetimidamide[4] (10a)

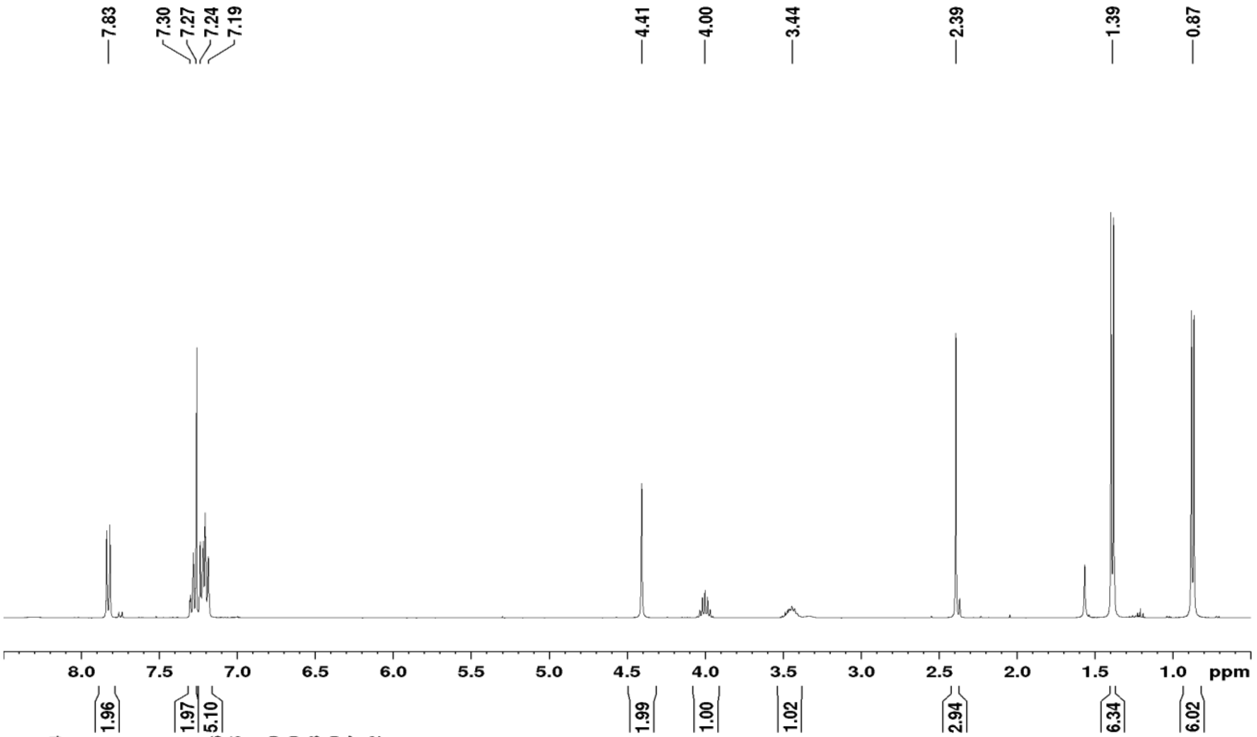

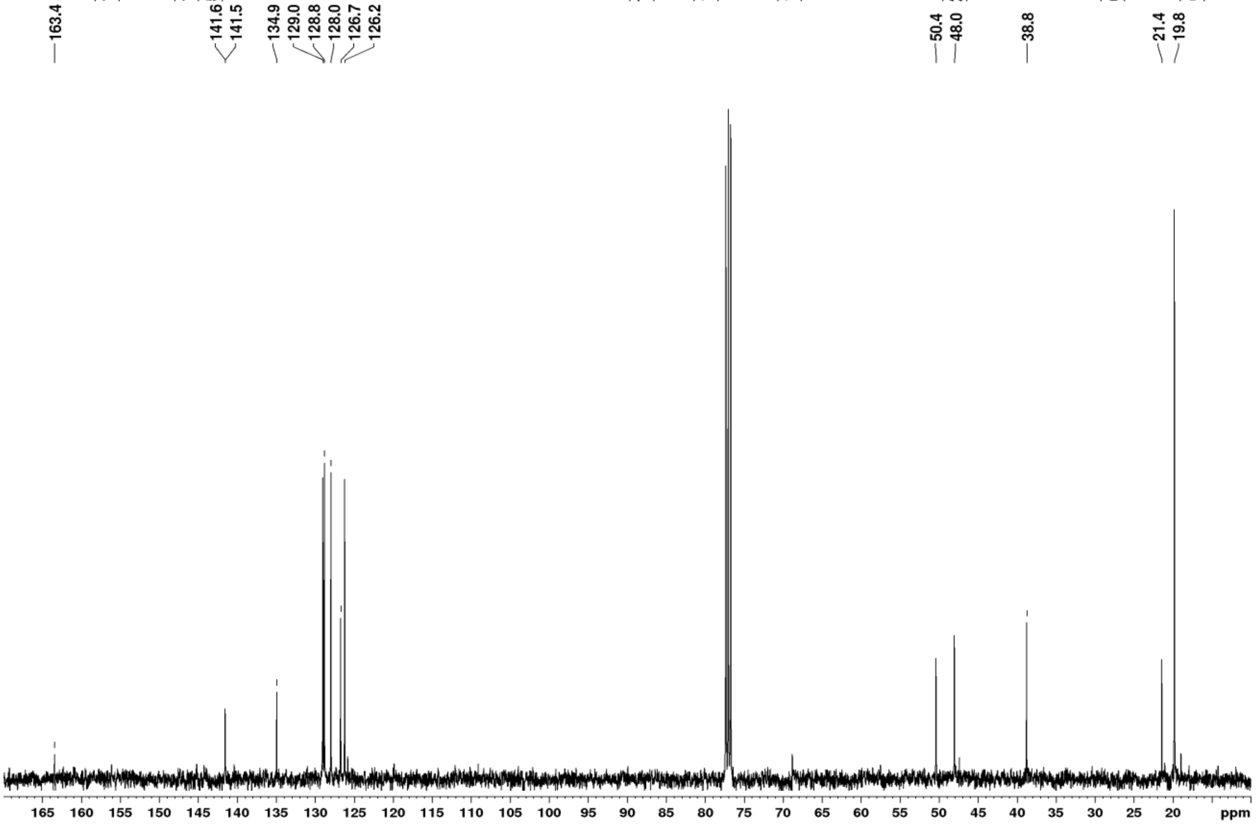

***N,N*-Diisopropyl-2-phenyl-*N*'-(phenylsulfonyl)acetimidamide**[5] **(10b)**

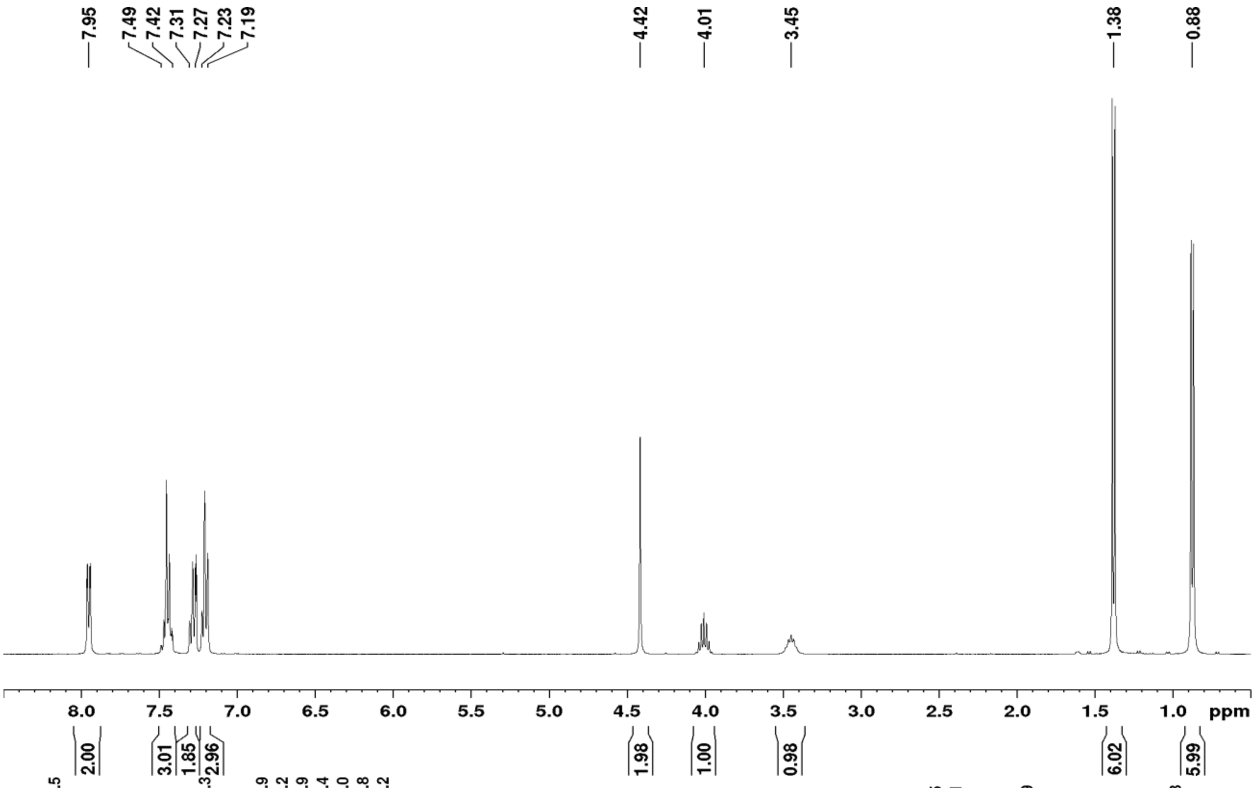

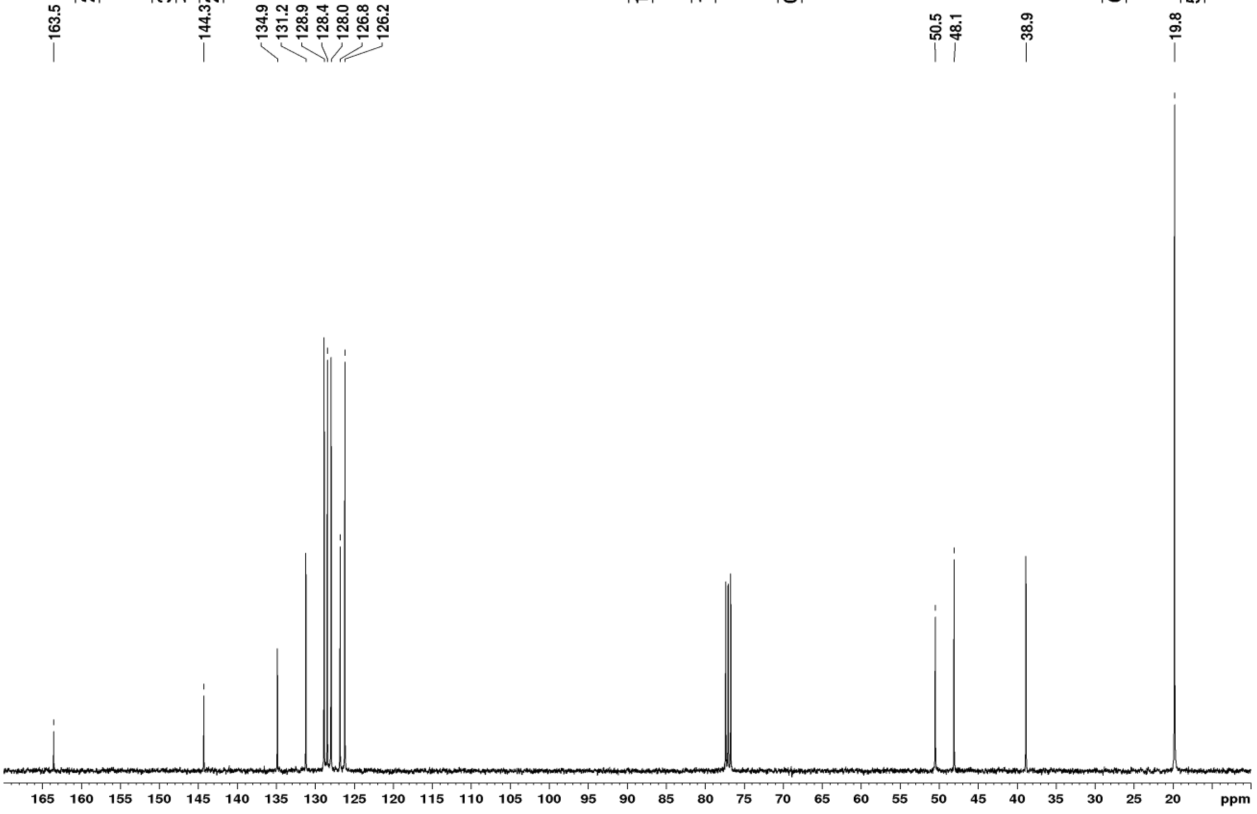

N,N-Diisopropyl-N'-((4-methoxyphenyl)sulfonyl)-2-phenylacetimidamide (10c)

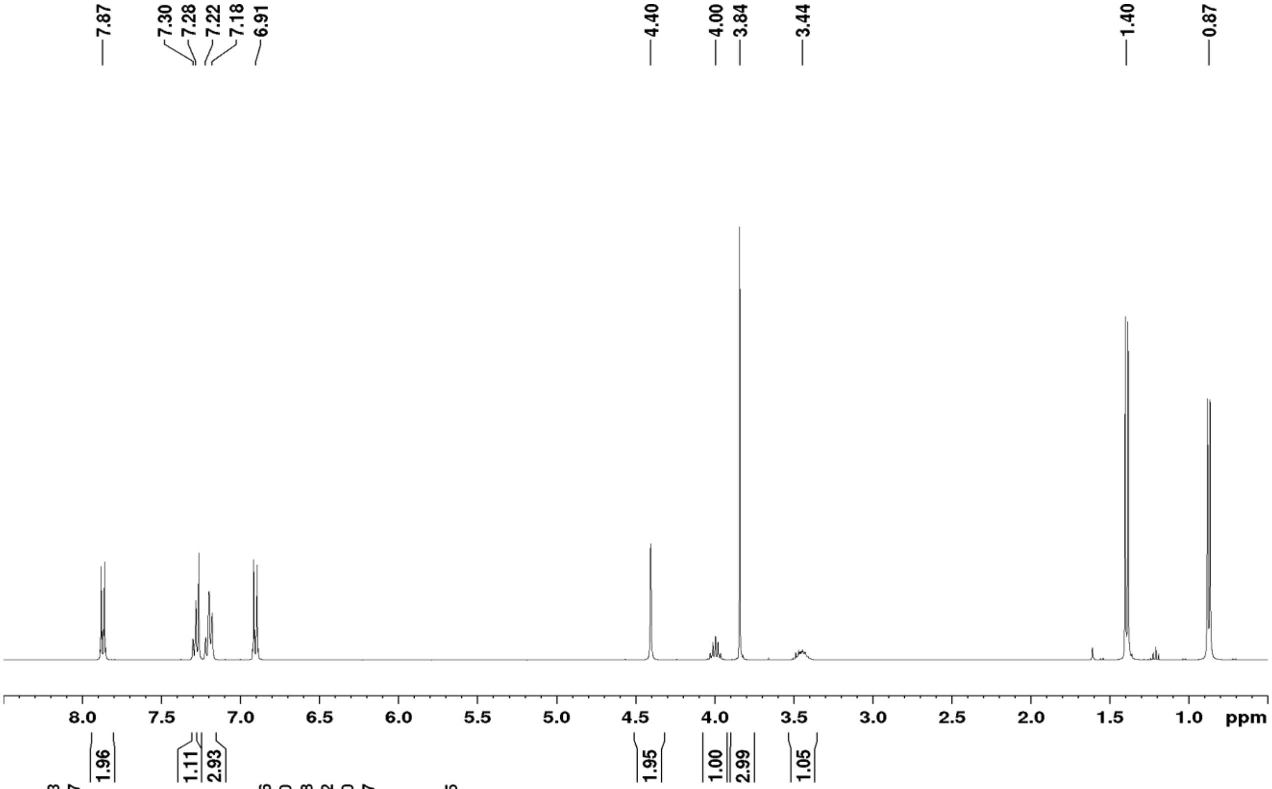

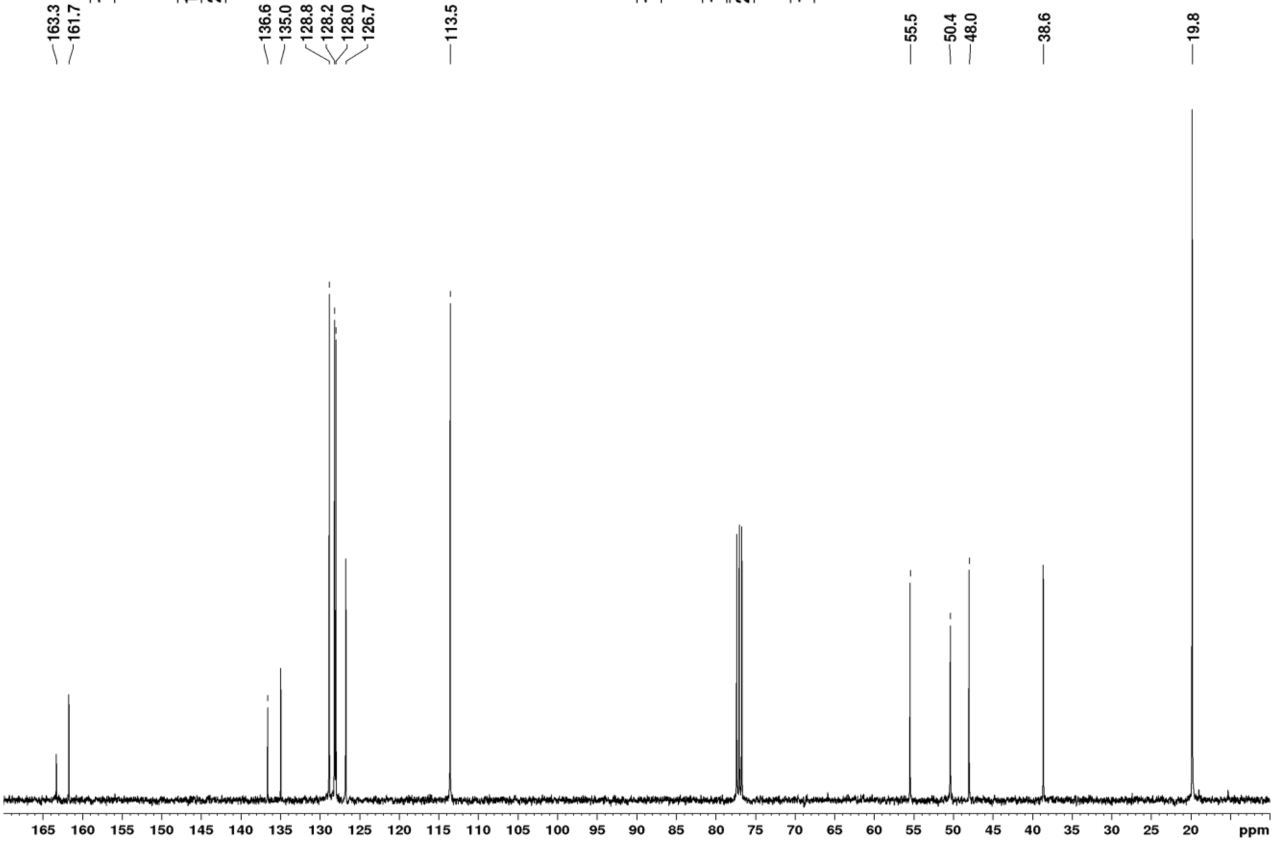

***N'*-((4-Bromophenyl)sulfonyl)-*N,N*-diisopropyl-2-phenylacetimidamide (10d)**

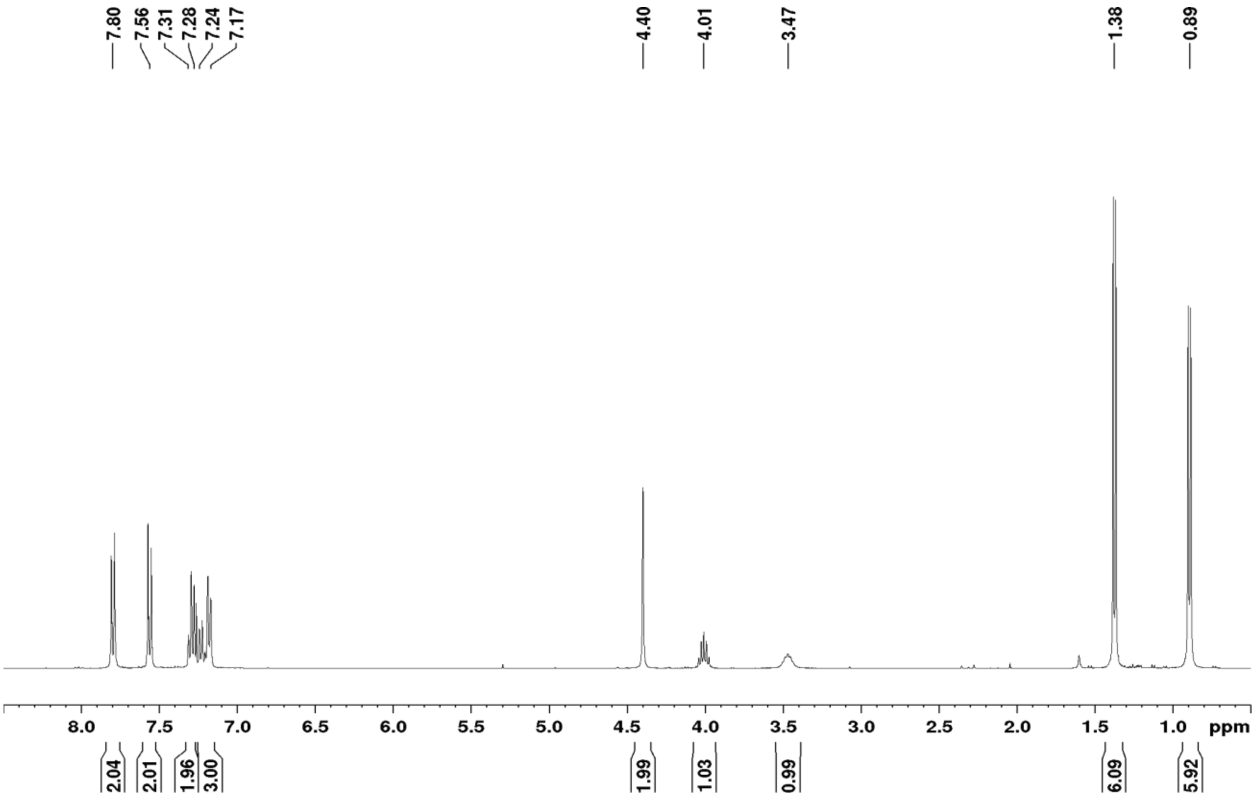

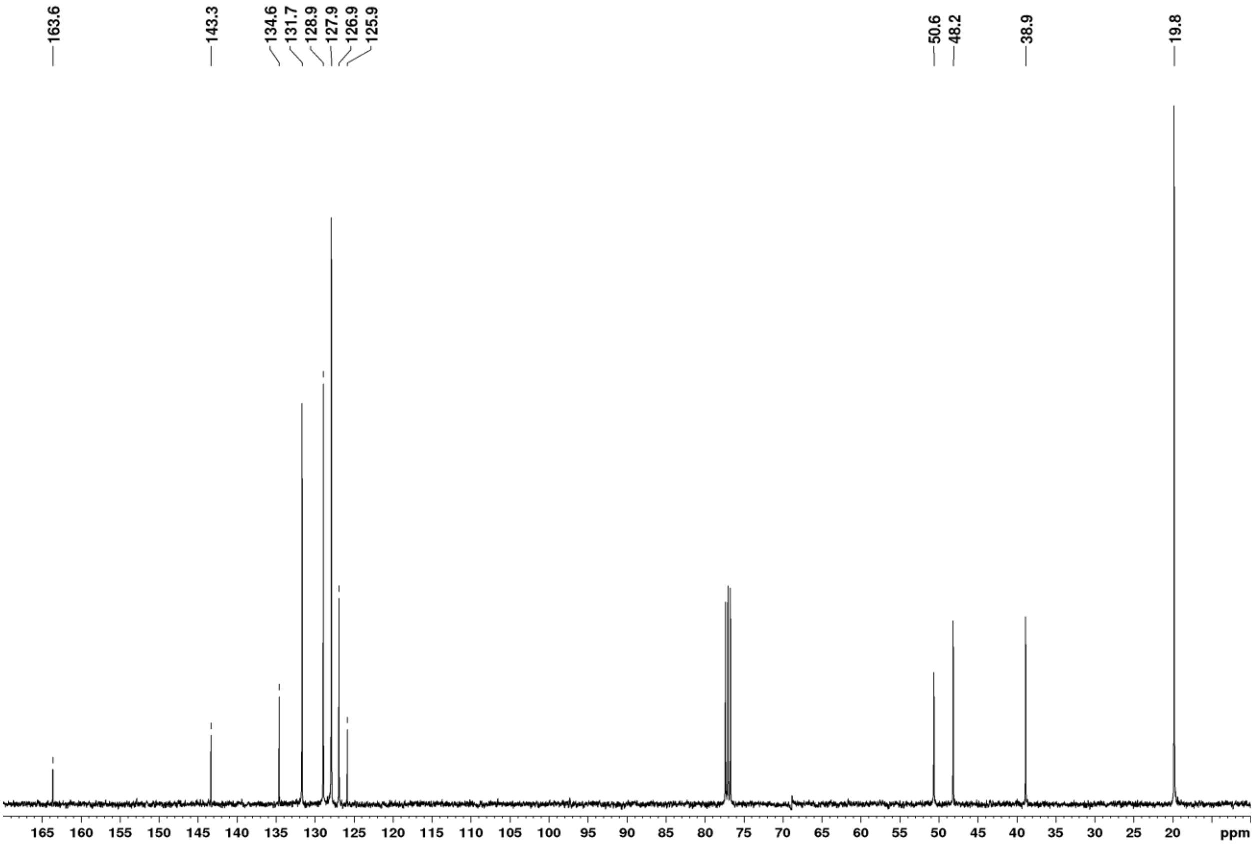

***N'*-((4-Cyanophenyl)sulfonyl)-*N,N*-diisopropyl-2-phenylacetimidamide (10e)**

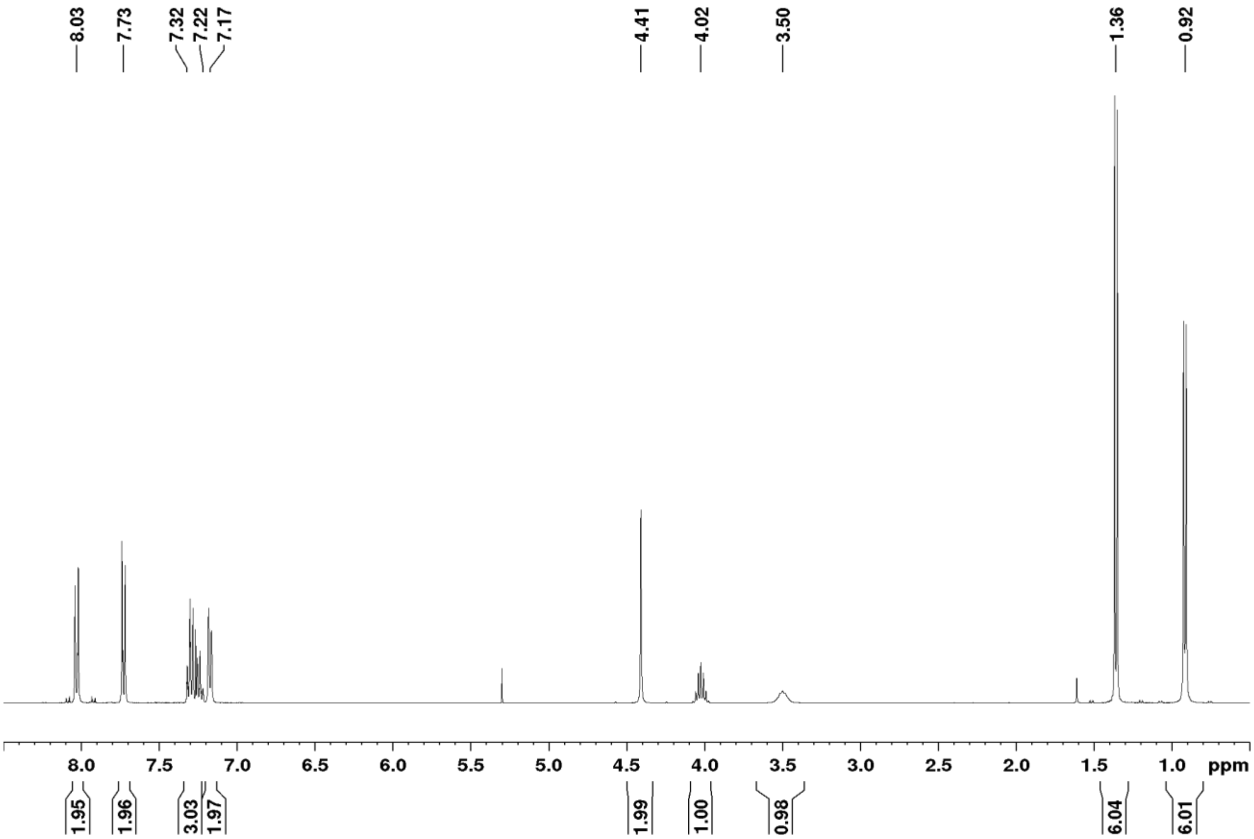

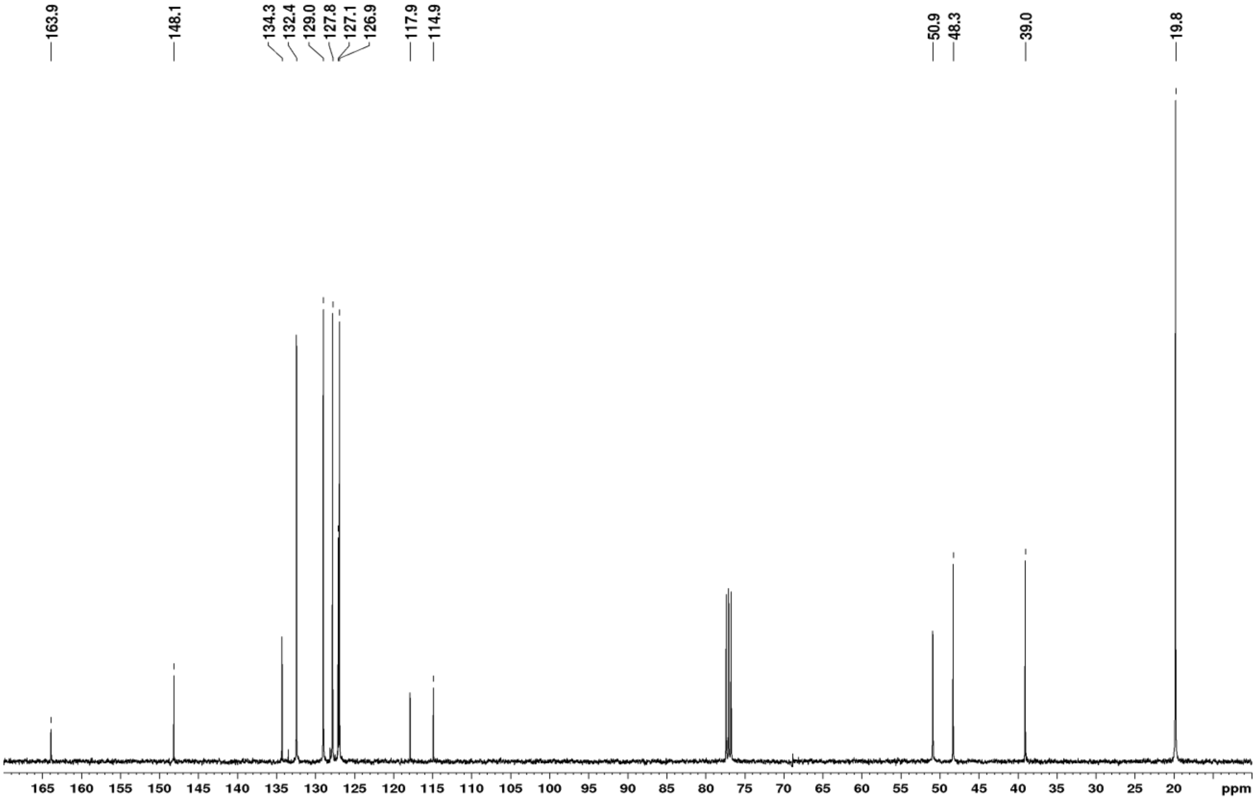

_N,N_-Diisopropyl-2-phenyl-_N'_-((2,4,6-triisopropylphenyl)sulfonyl)acetimidamide (10f)

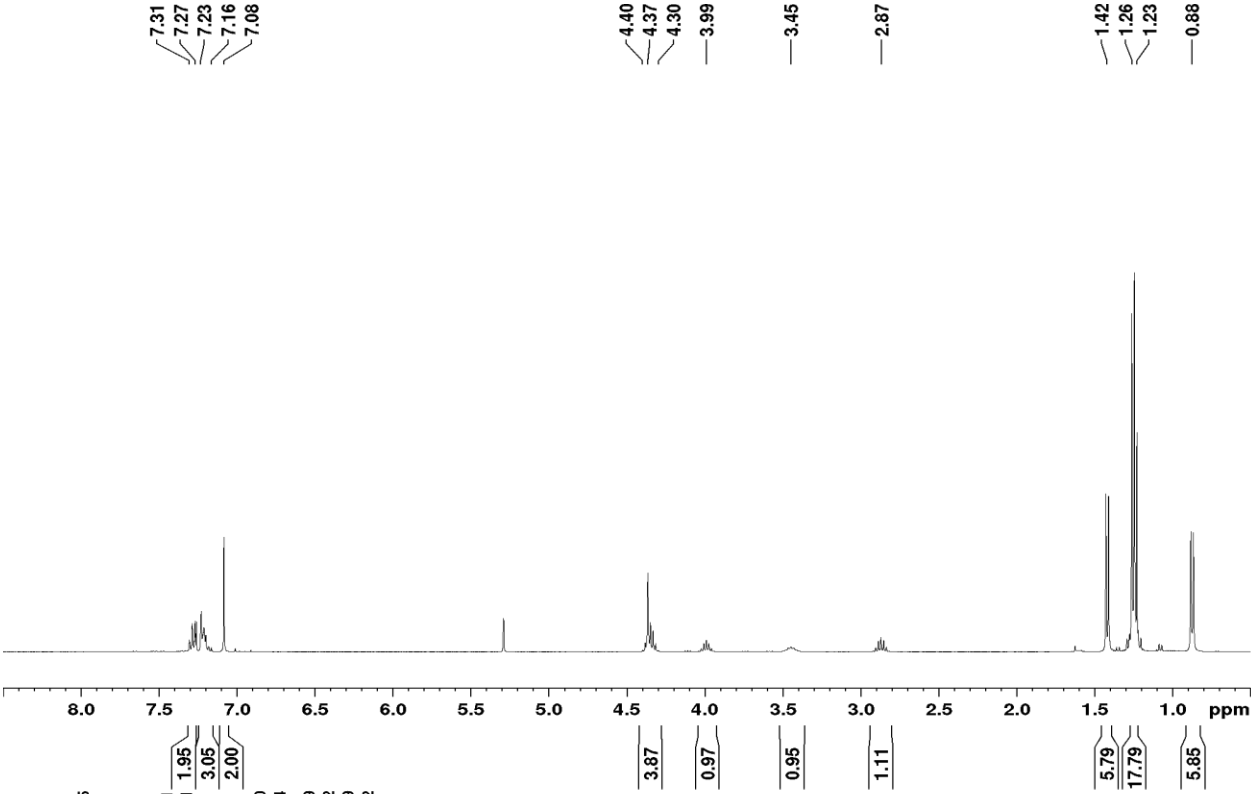

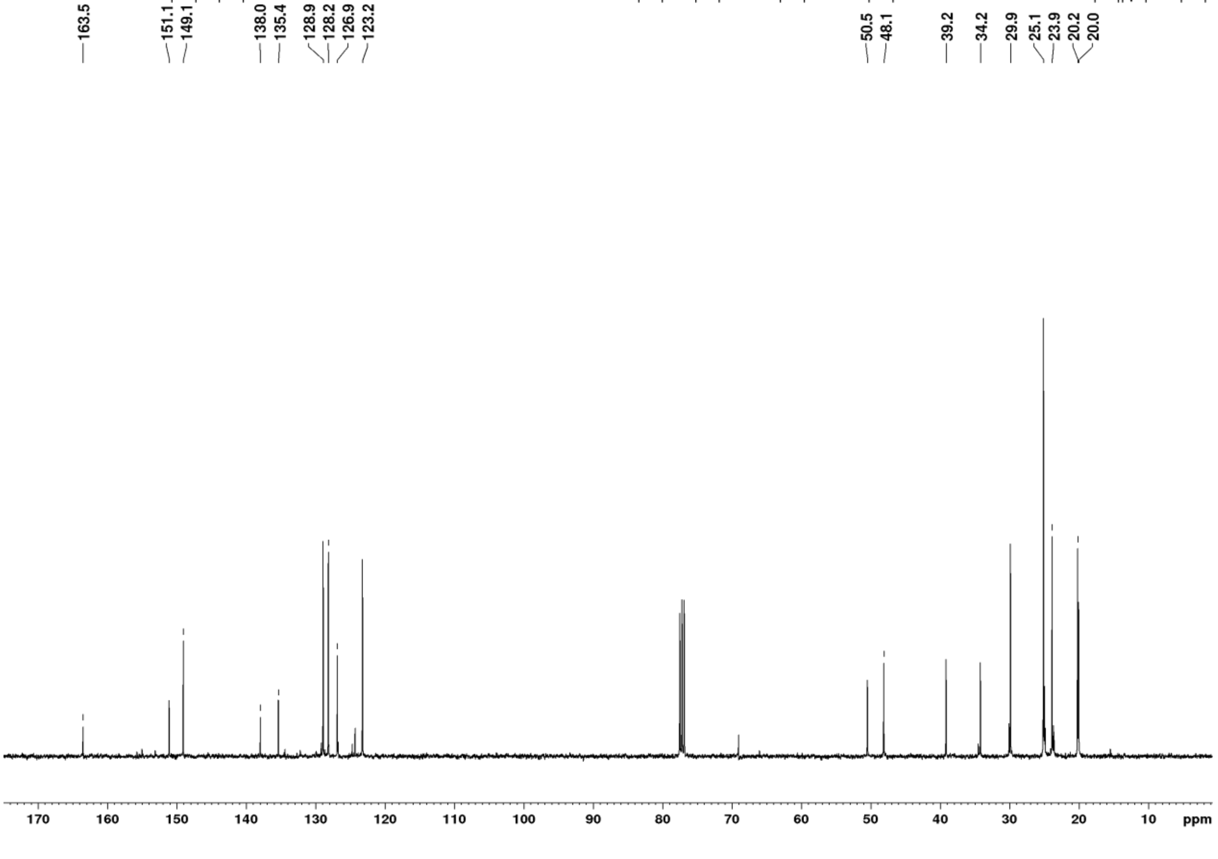

***N,N*-Diisopropyl-*N'*-(naphthalen-1-ylsulfonyl)-2-phenylacetimidamide (10g)**

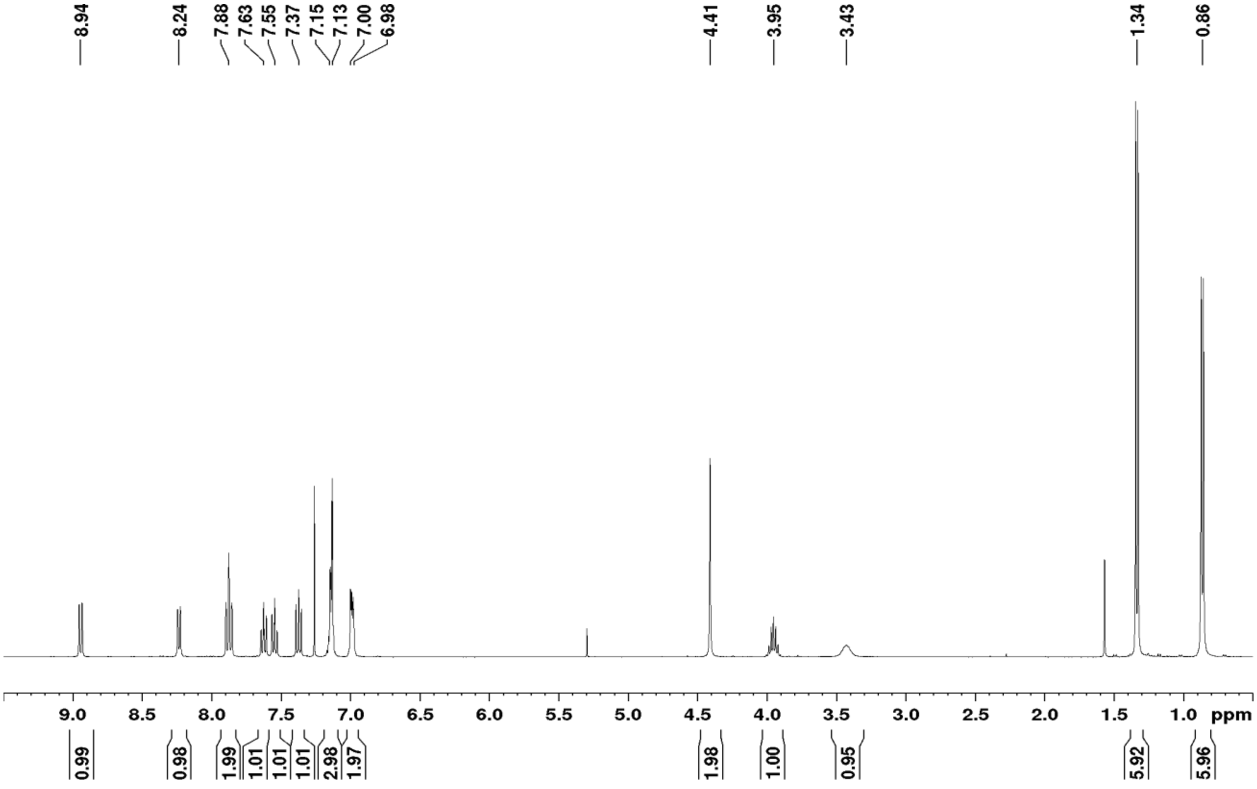

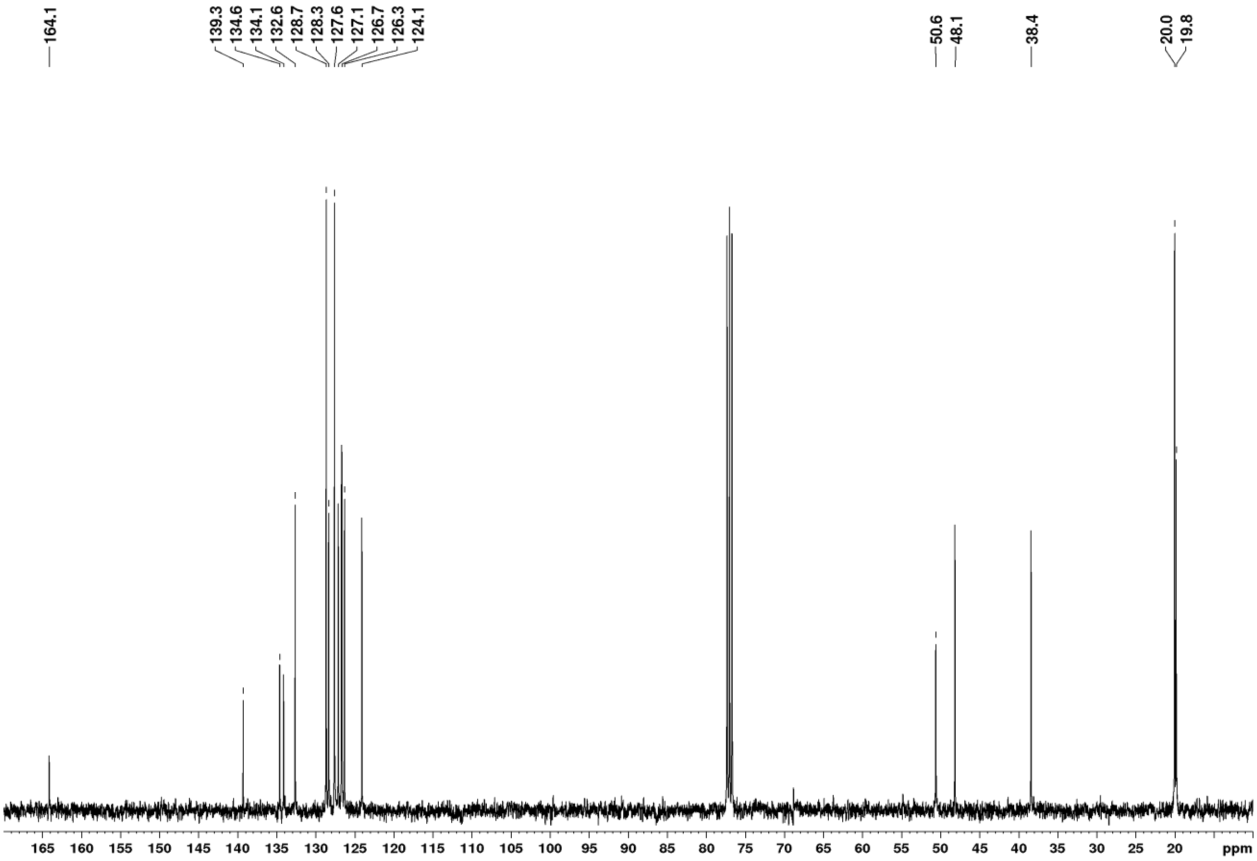

2-(4-Fluorophenyl)-*N,N*-diisopropyl-*N'*-tosylacetimidamide[7] (11a)

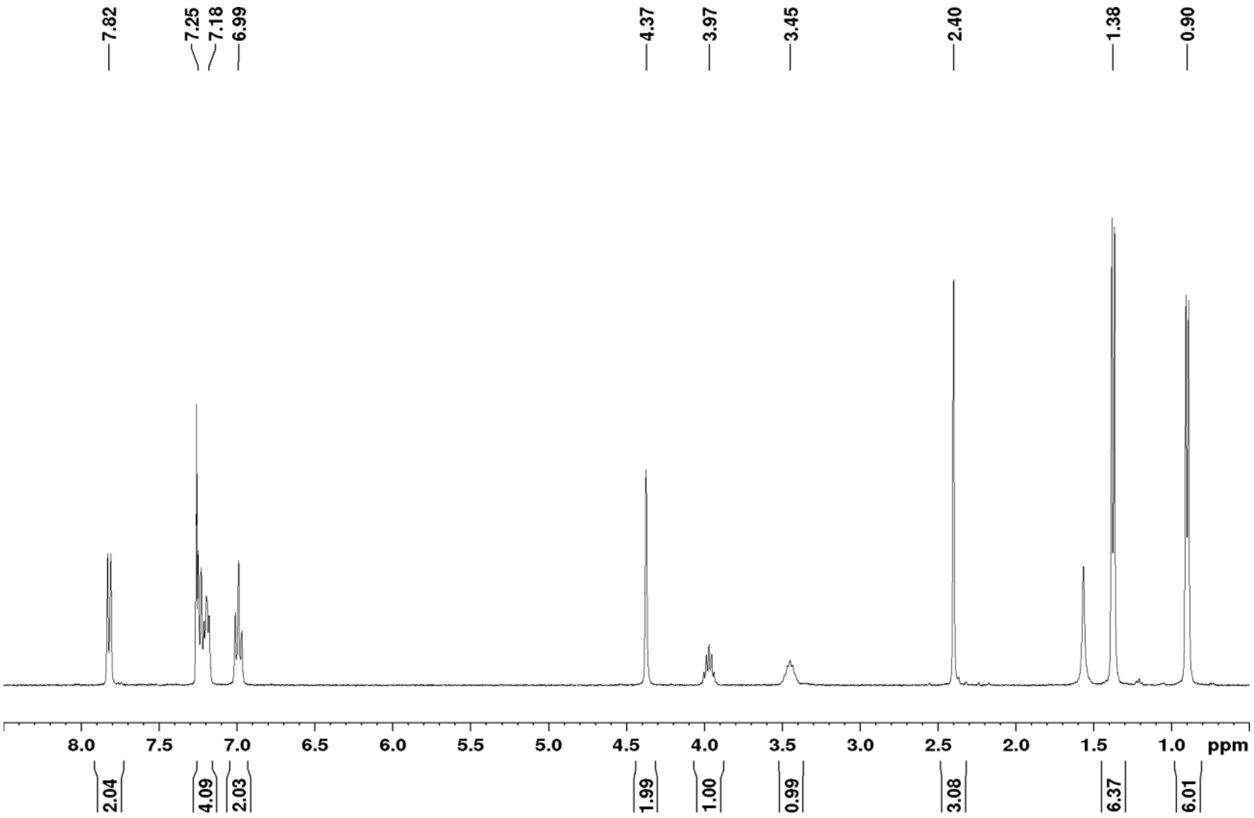

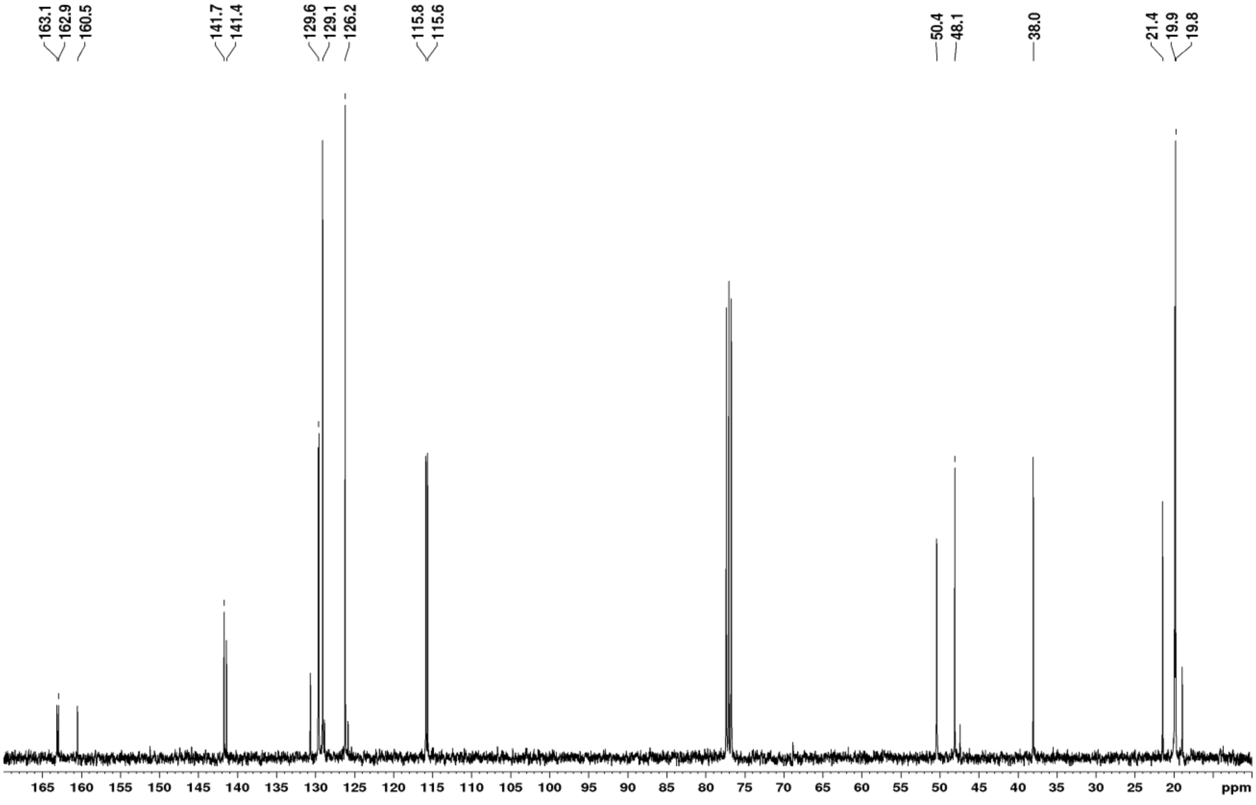

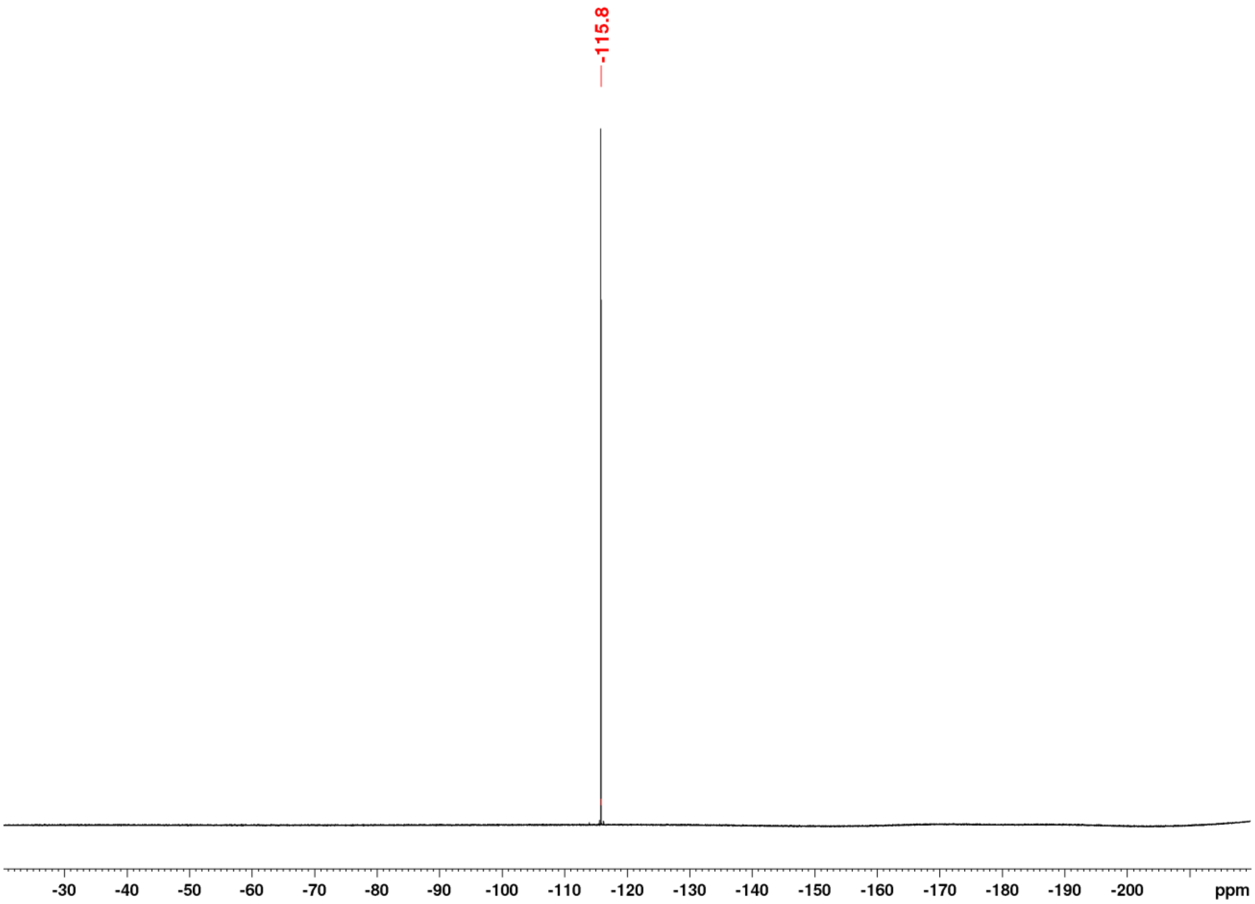
-115.8
-30
-40
-50
-60
-70
-80
-90
-100
-110
-120
-130
-140
-150
-160
-170
-180
-190
-200
ppm

***N,N*-Diisopropyl-2-(*p*-tolyl)-*N'*-tosylacetimidamide**[4] **(11b)**

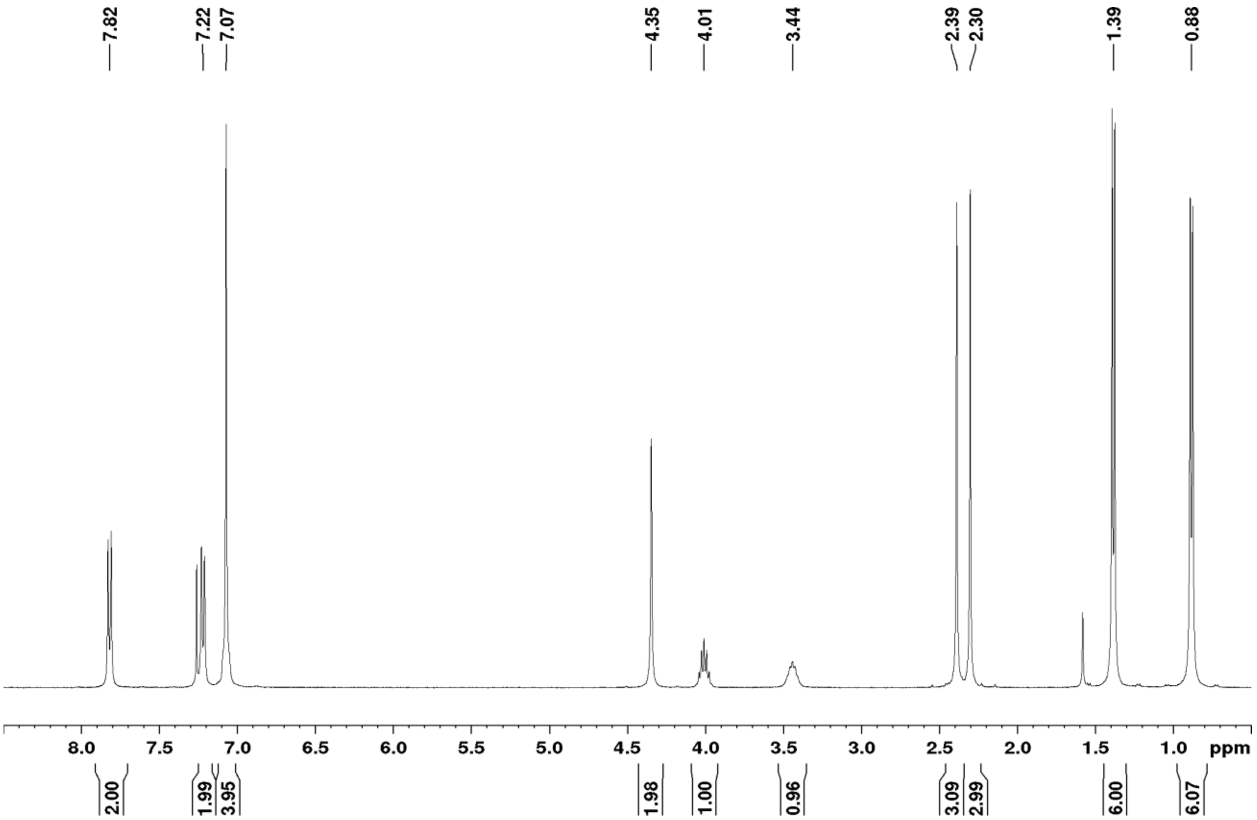

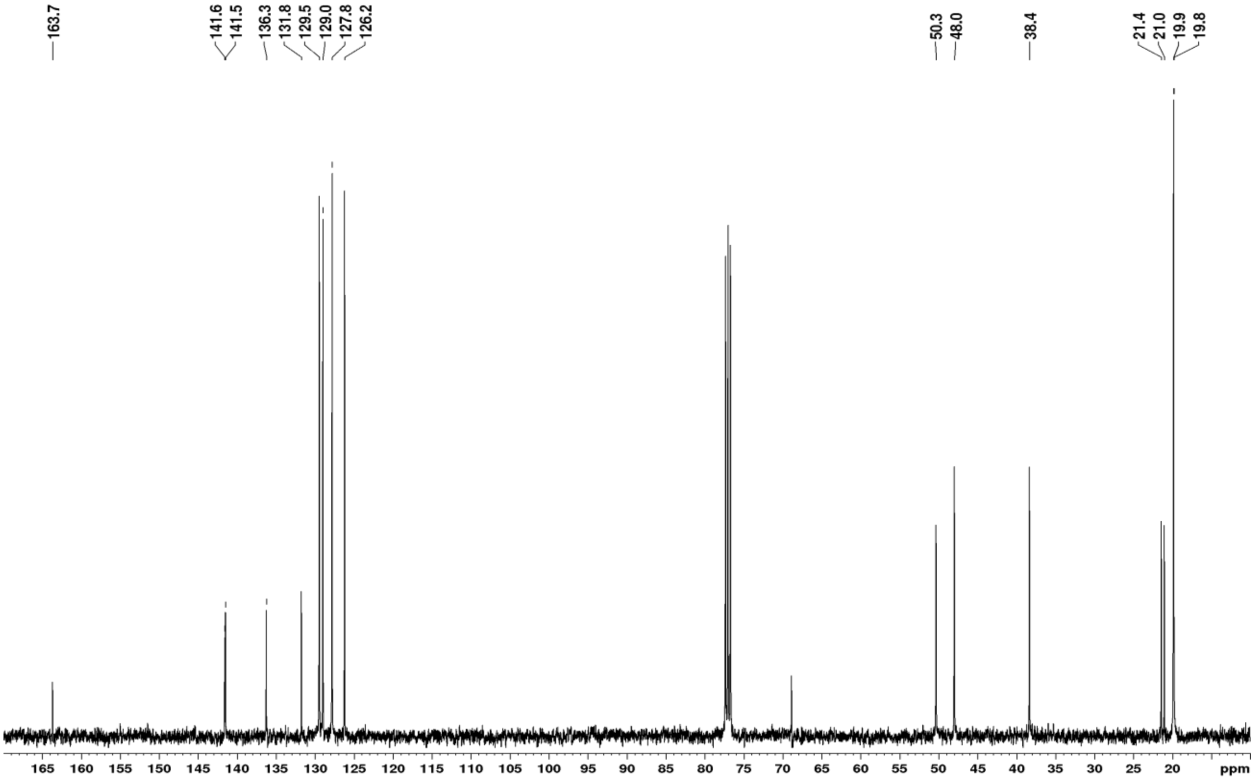

N,N-Diisopropyl-2-(4-methoxyphenyl)-N'-tosylacetimidamide[7] **(11c)**

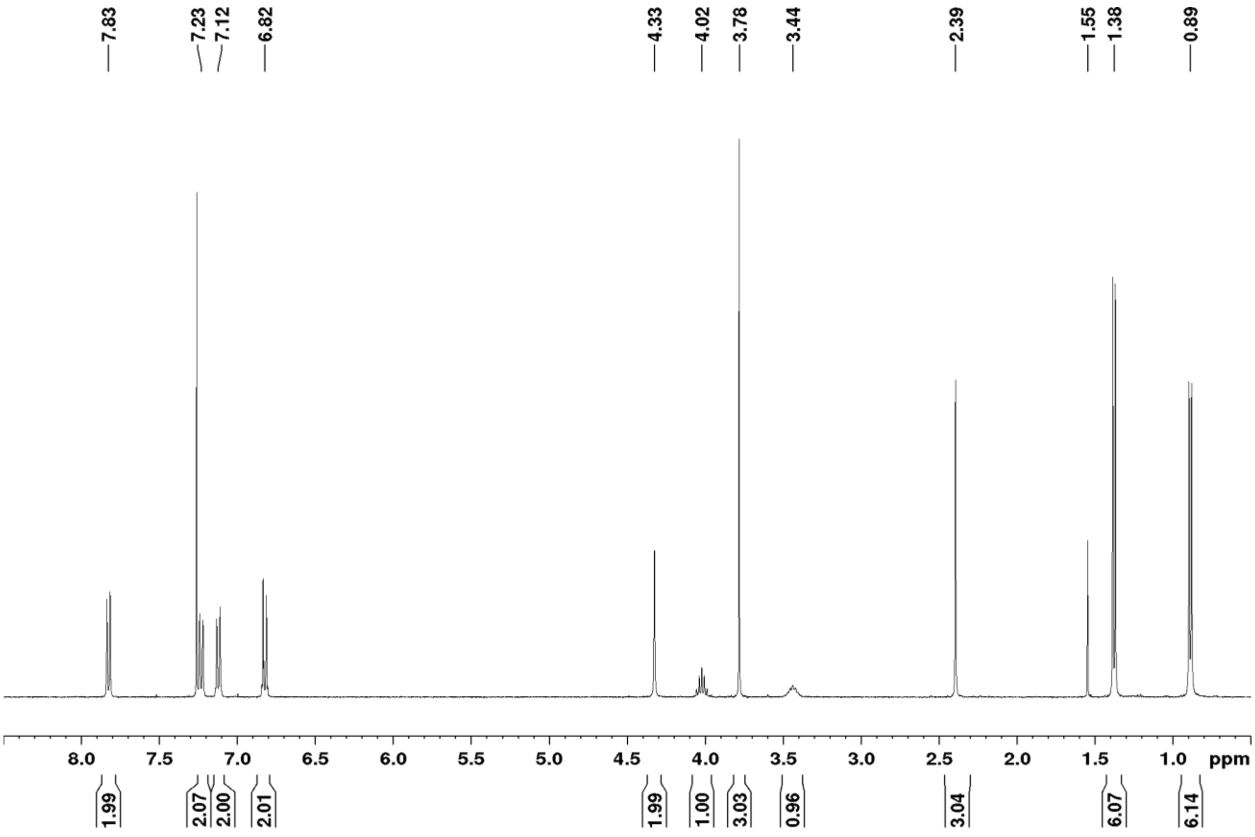

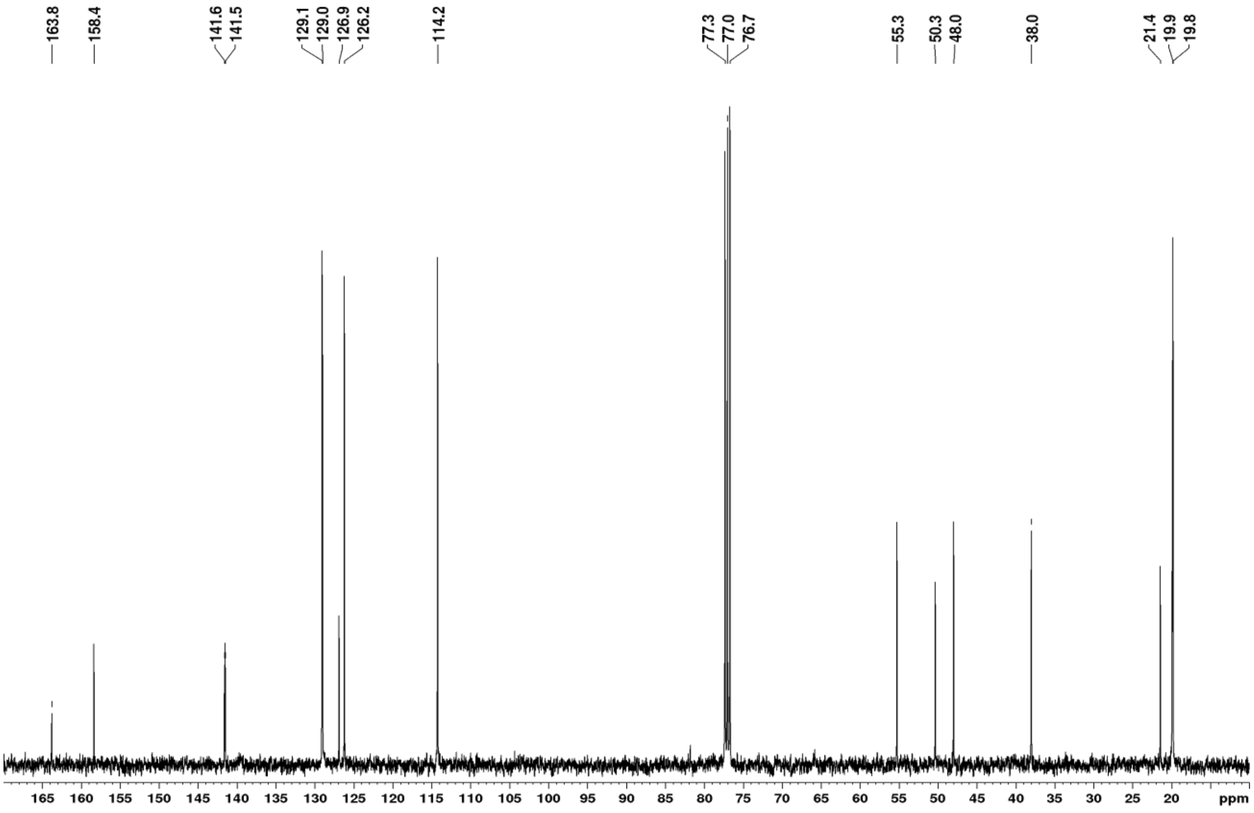

2-(4-(*tert*-Butyl)phenyl)-*N,N*-diisopropyl-*N'*-tosylacetimidamide[7] (11d)

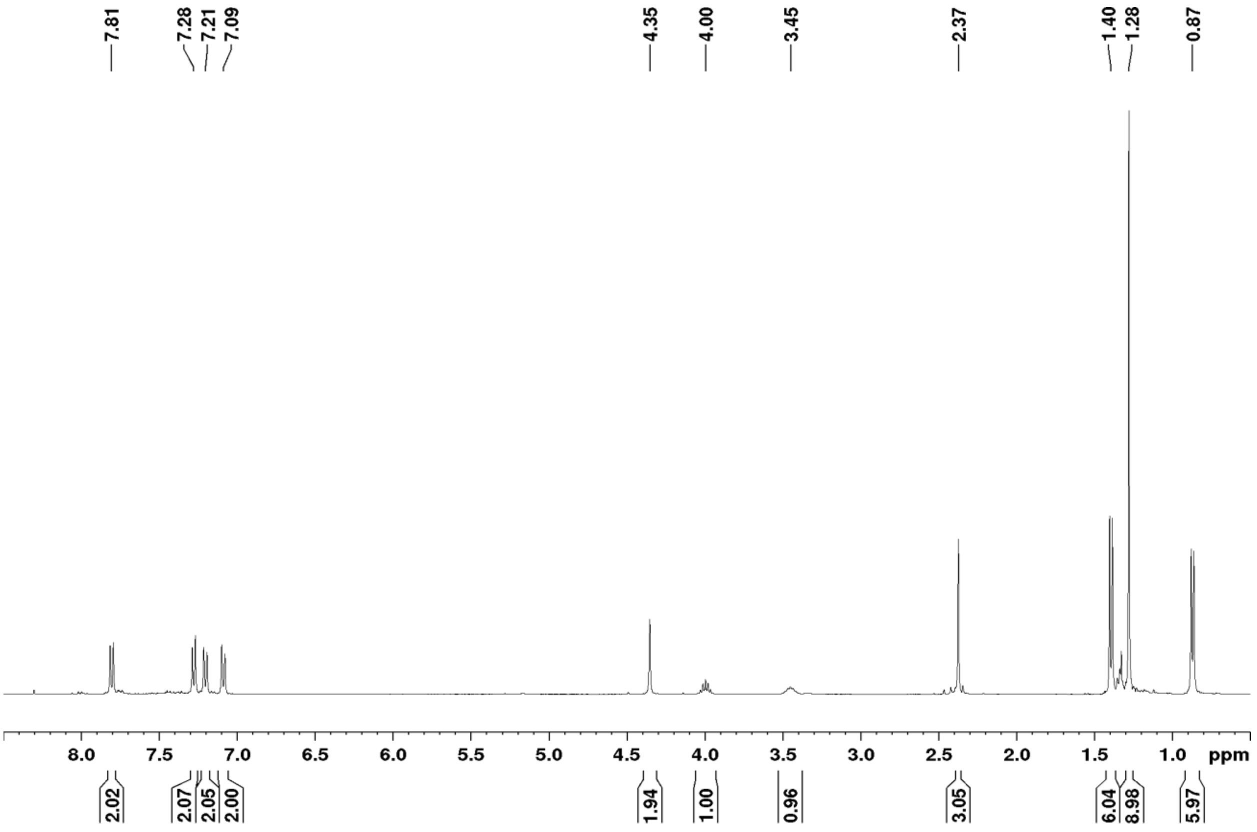

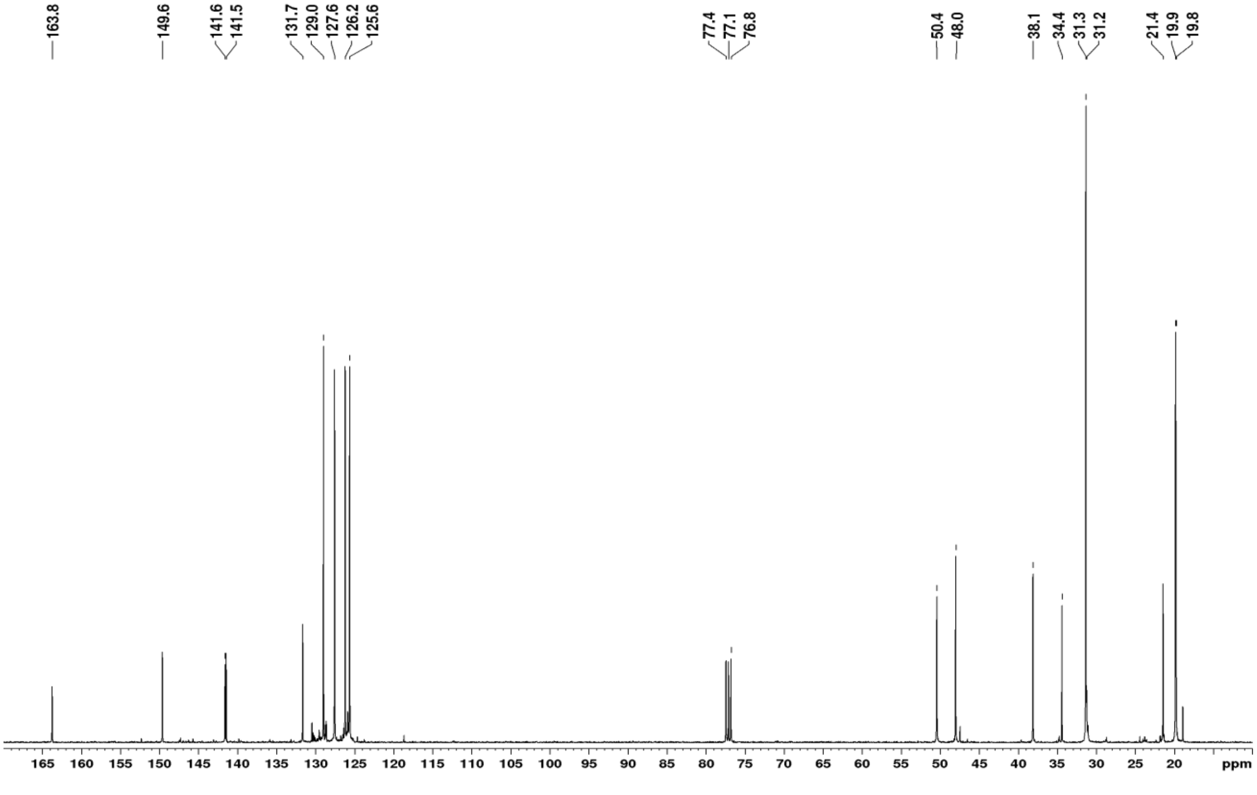

2-(3-fluorophenyl)-*N,N*-diisopropyl-*N'*-tosylacetimidamide (11e)

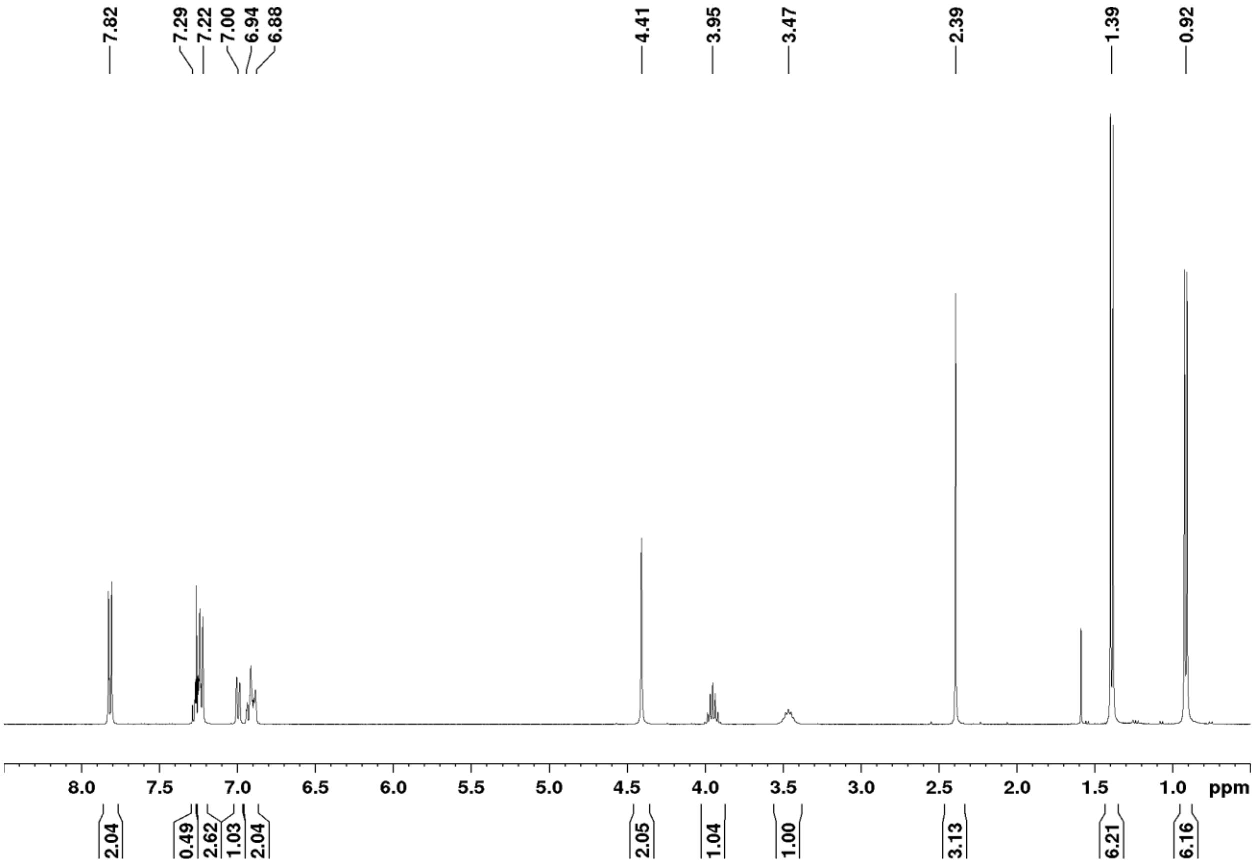

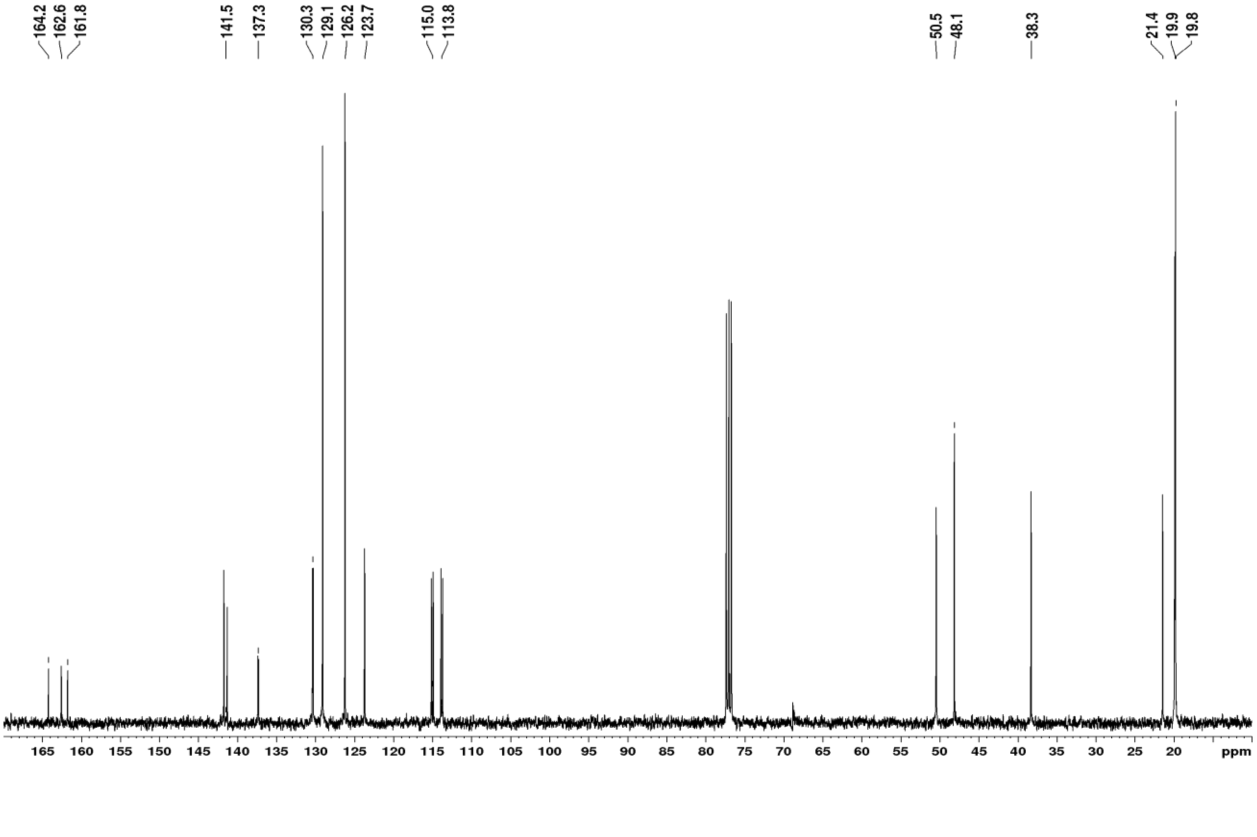

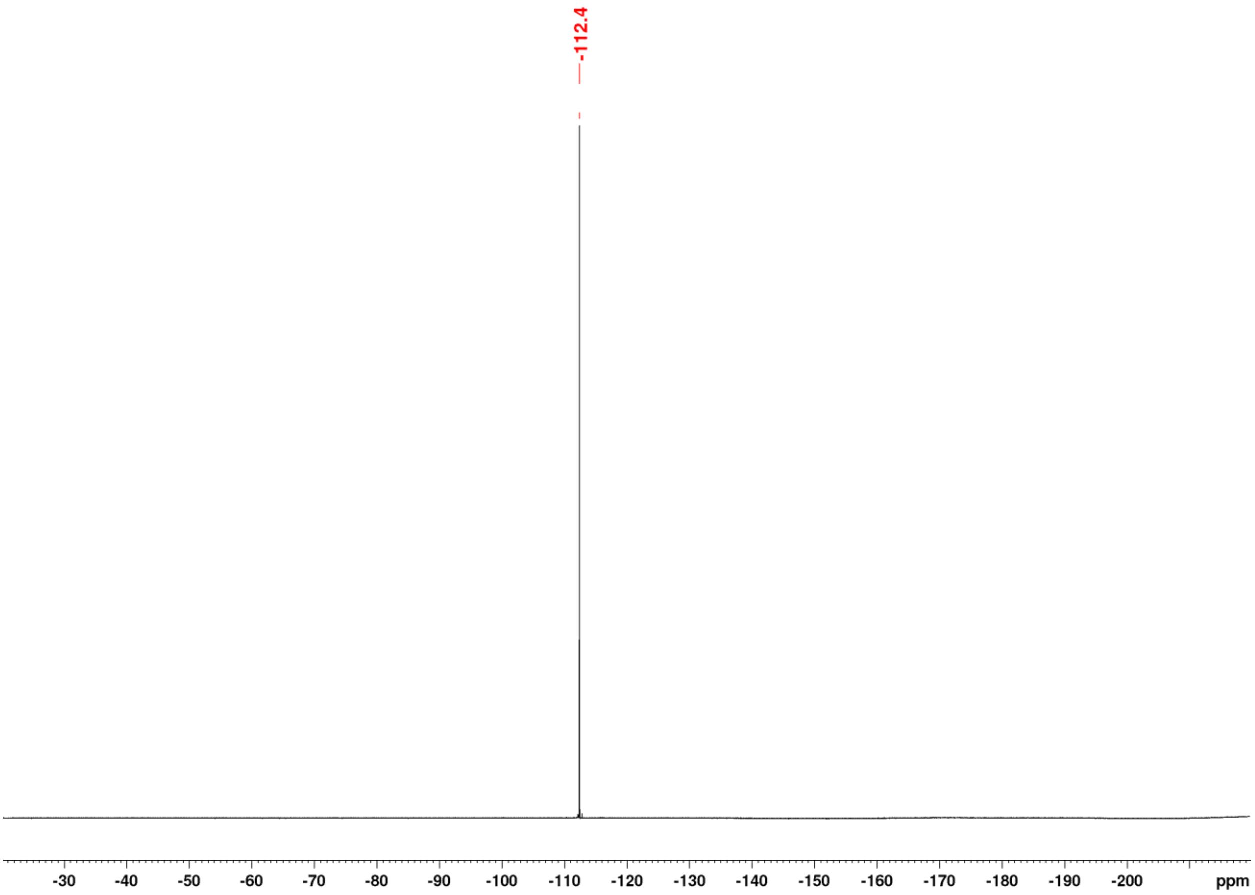
-112.4
-30
-40
-50
-60
-70
-80
-90
-100
-110
-120
-130
-140
-150
-160
-170
-180
-190
-200
ppm

_N,N_-Diisopropyl-2-(_m_-tolyl)-_N'_-tosylacetimidamide (11f)

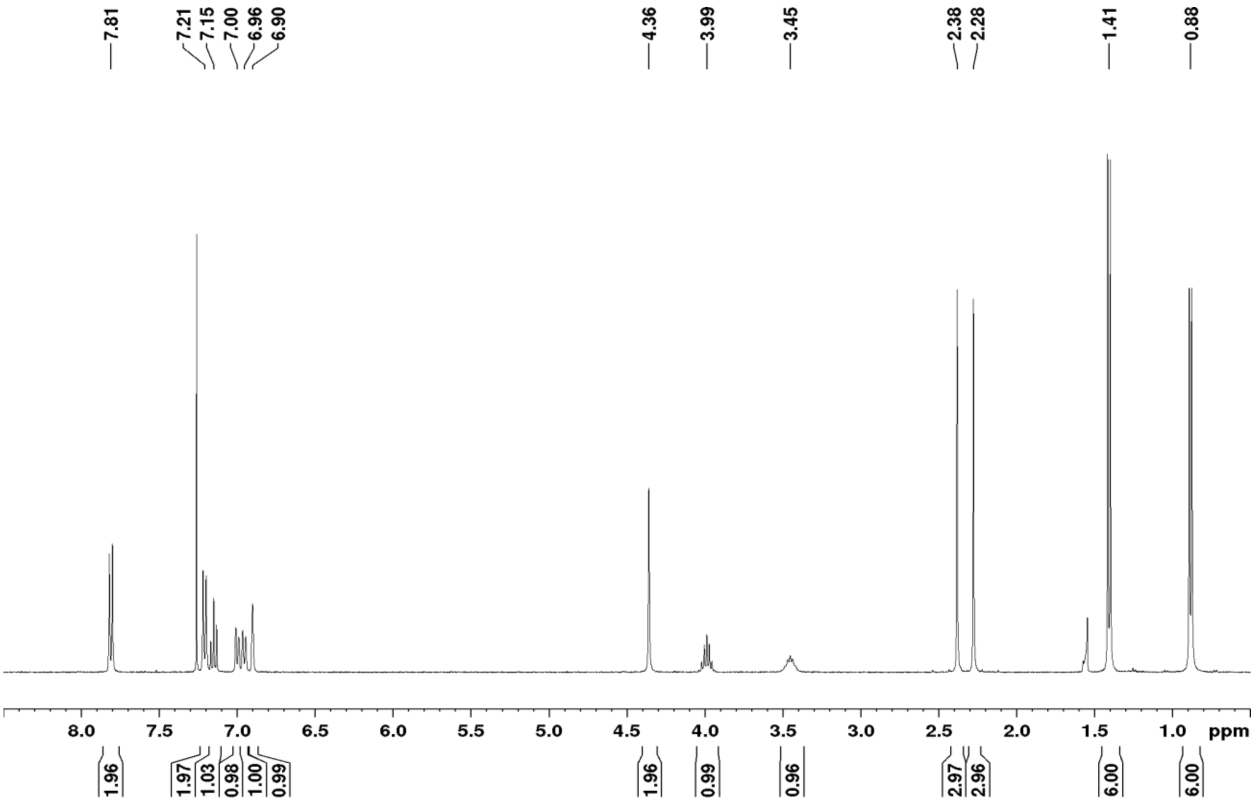

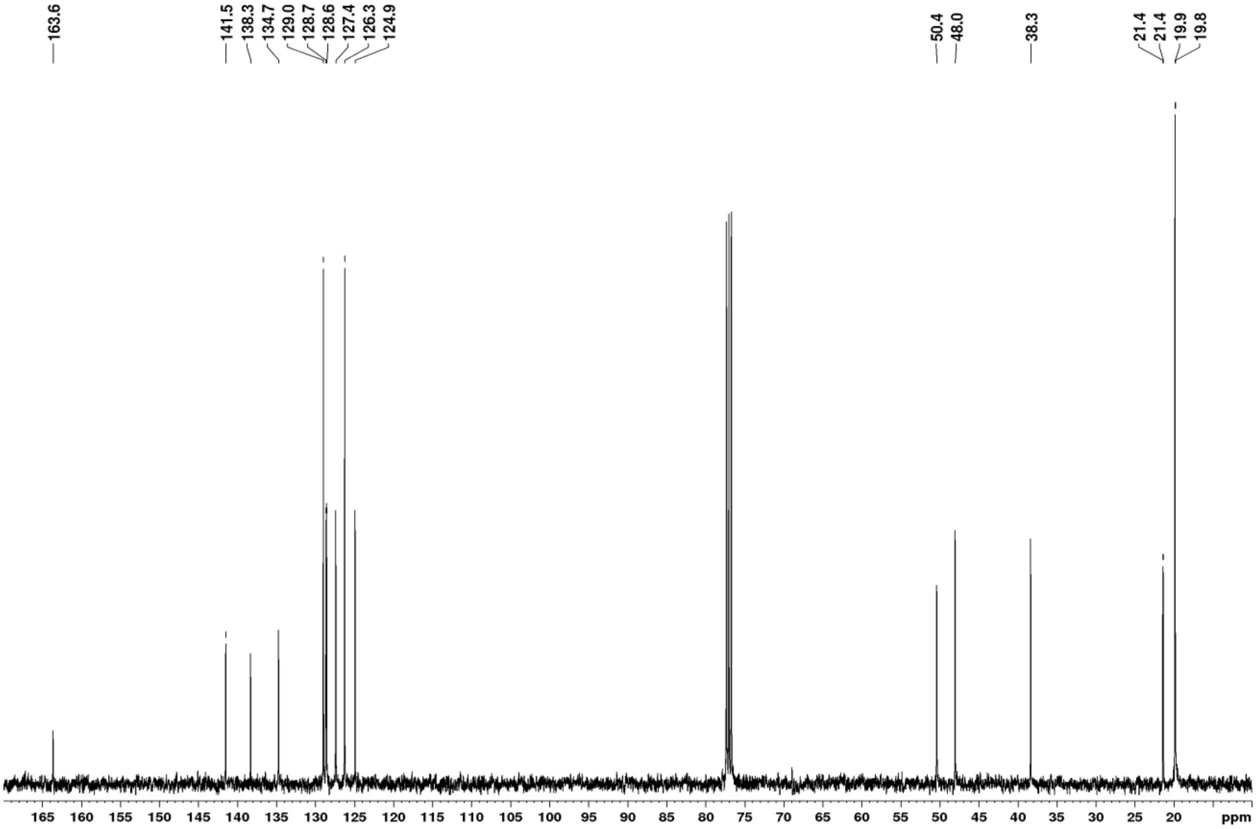

***N,N*-Diisopropyl-2-(2-methoxyphenyl)-*N'*-tosylacetimidamide (11g)**

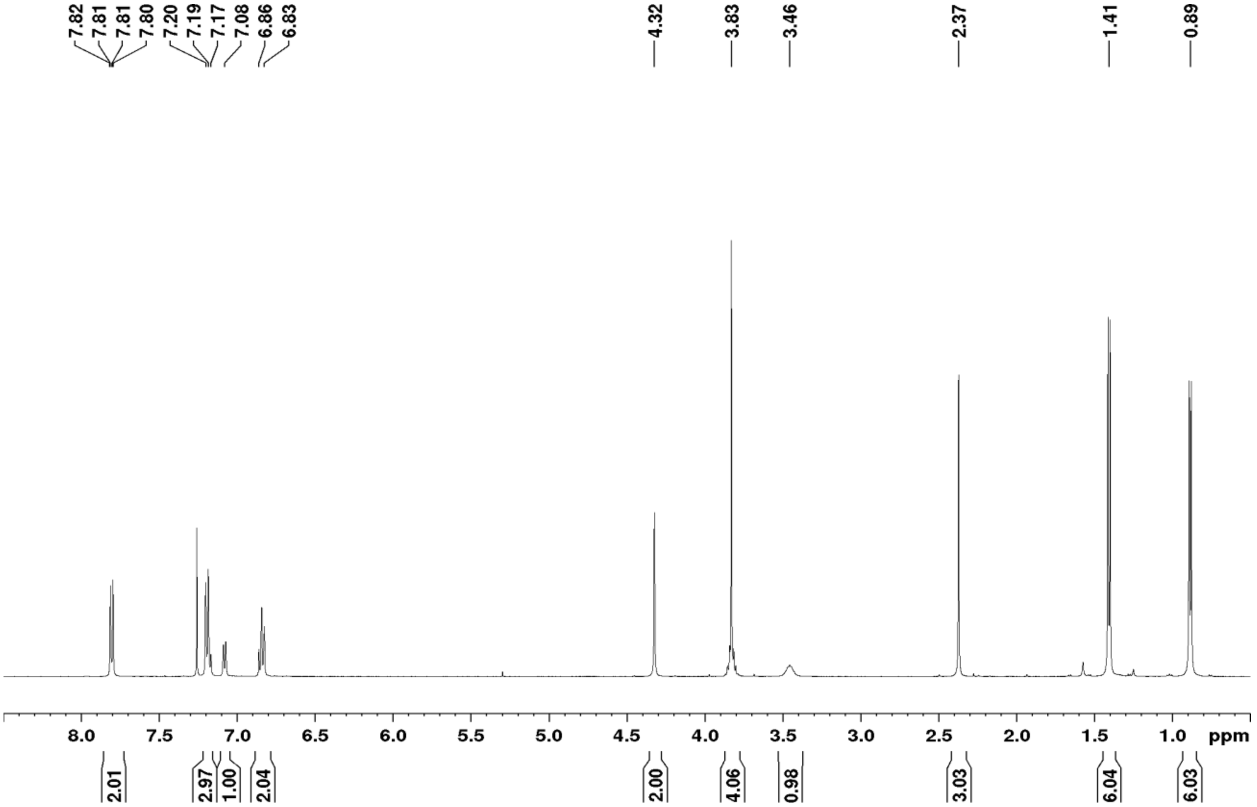

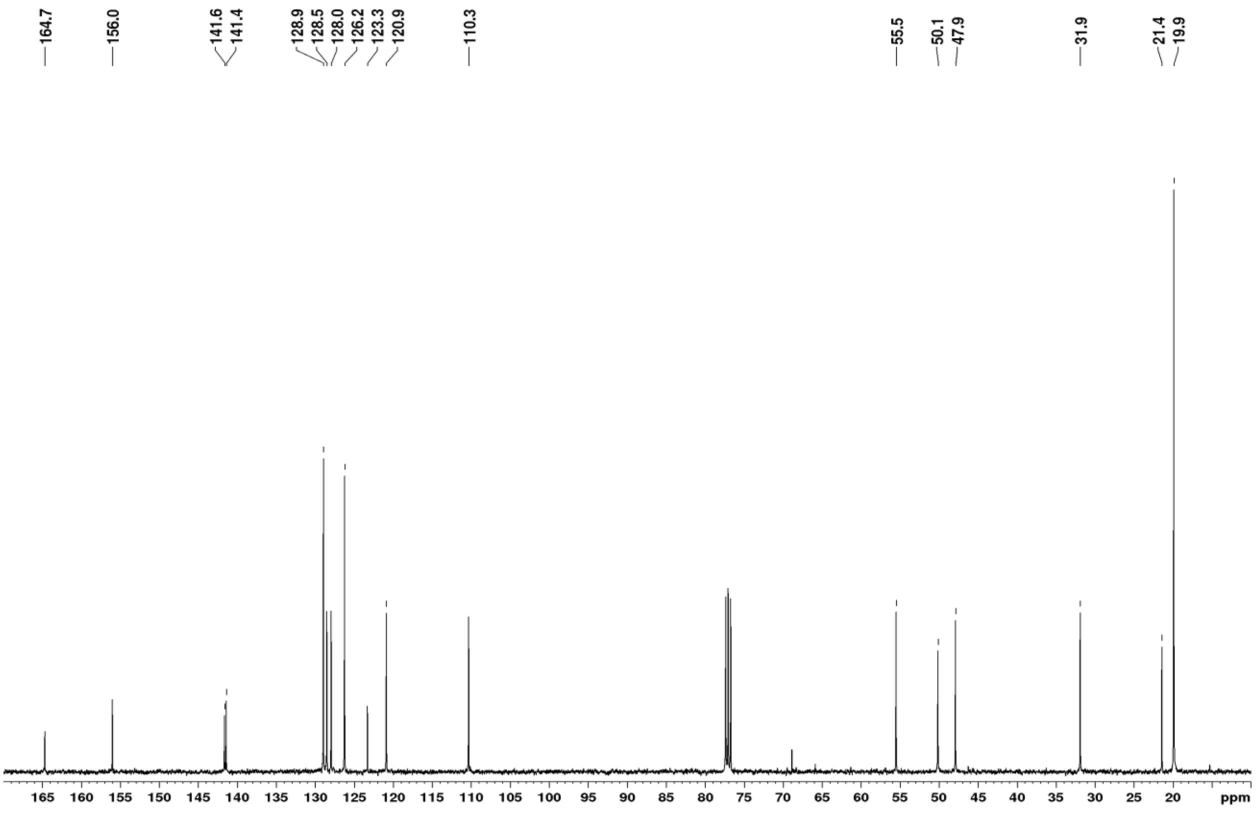

2-(3,5-Bis(trifluoromethyl)phenyl)-*N,N*-diisopropyl-*N'*-tosylacetimidamide (11h)

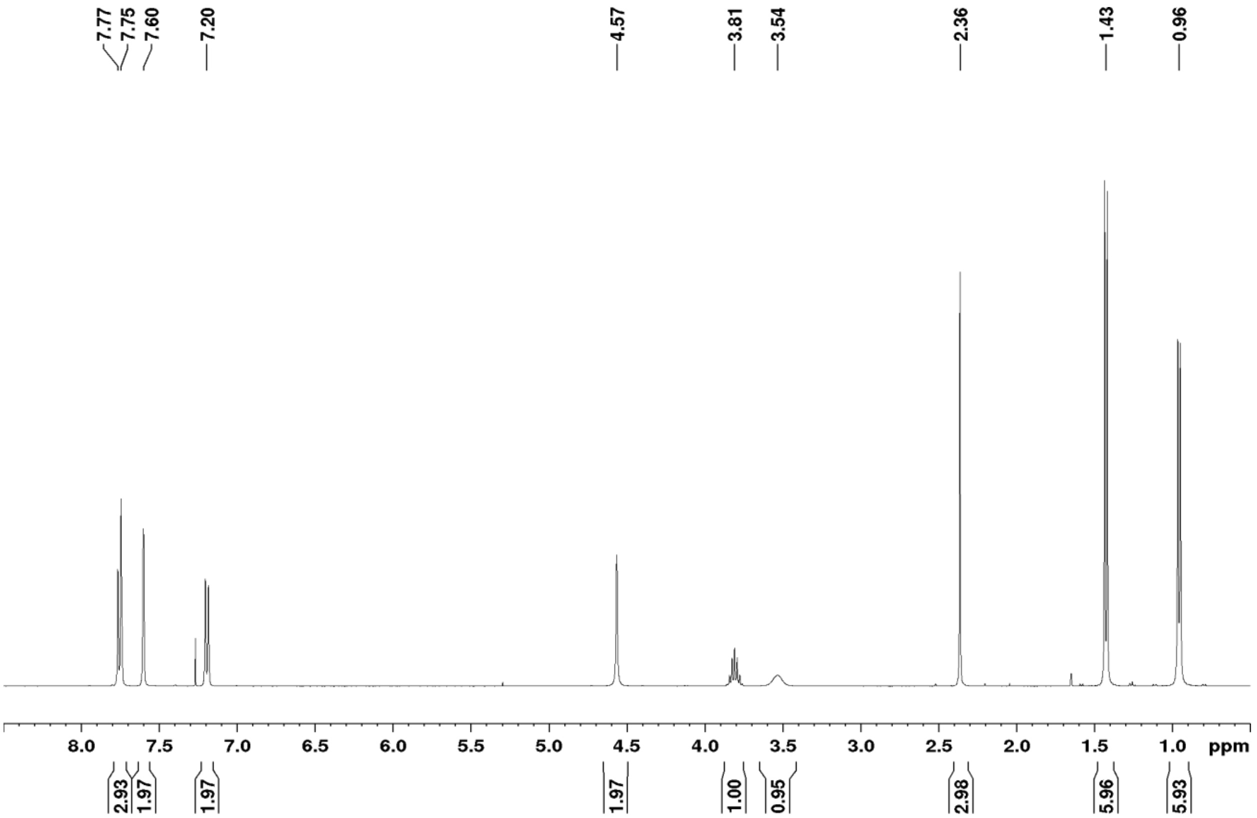

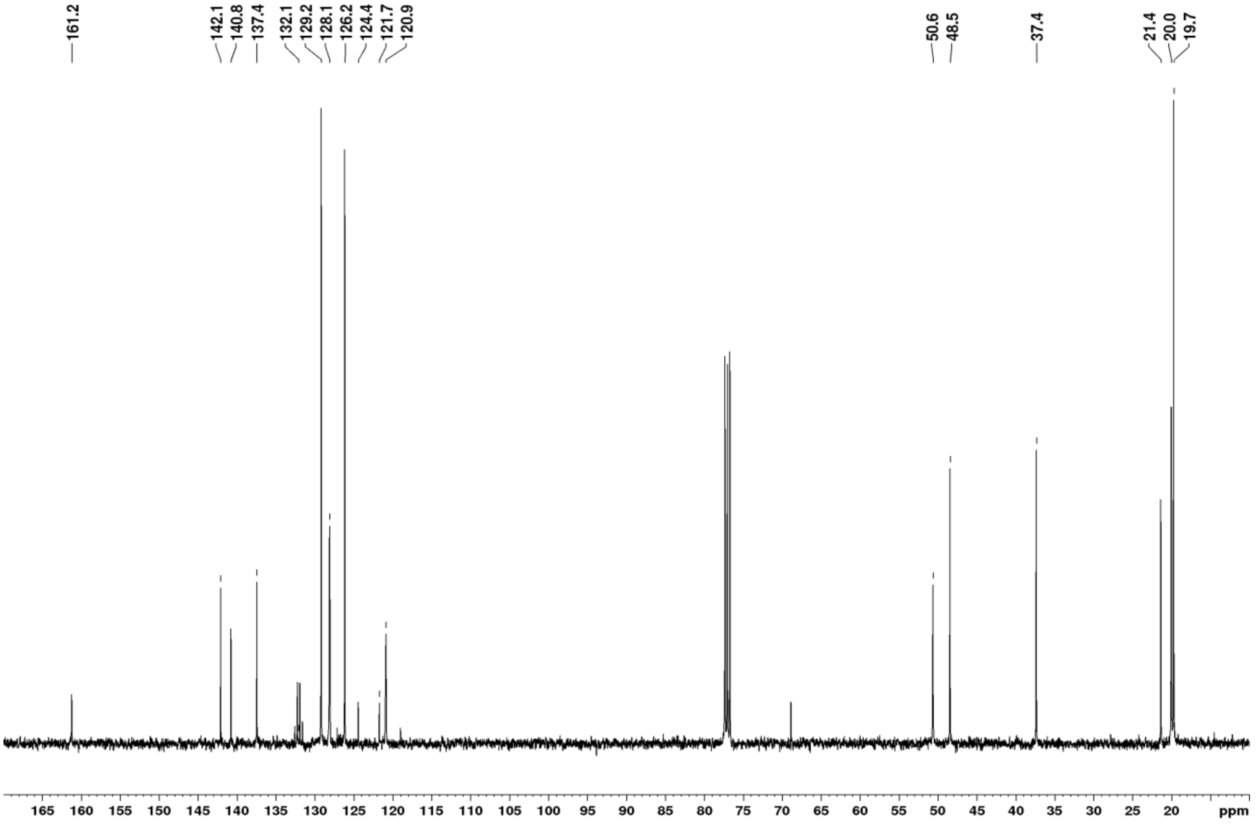

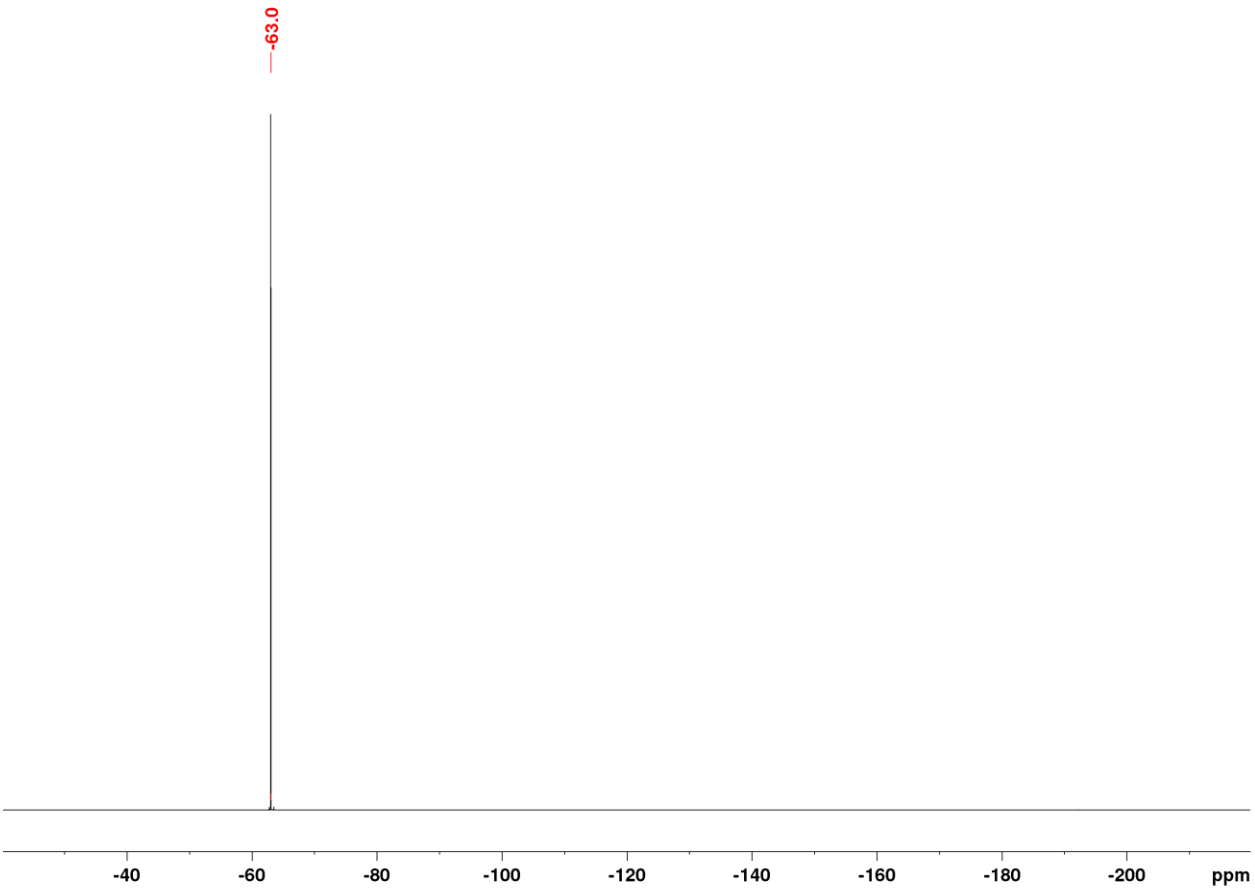

-63.0
-40
-60
-80
-100
-120
-140
-160
-180
-200
ppm

***N,N*-Diisopropyl-2-(phenanthren-9-yl)-*N'*-tosylacetimidamide (11i)**

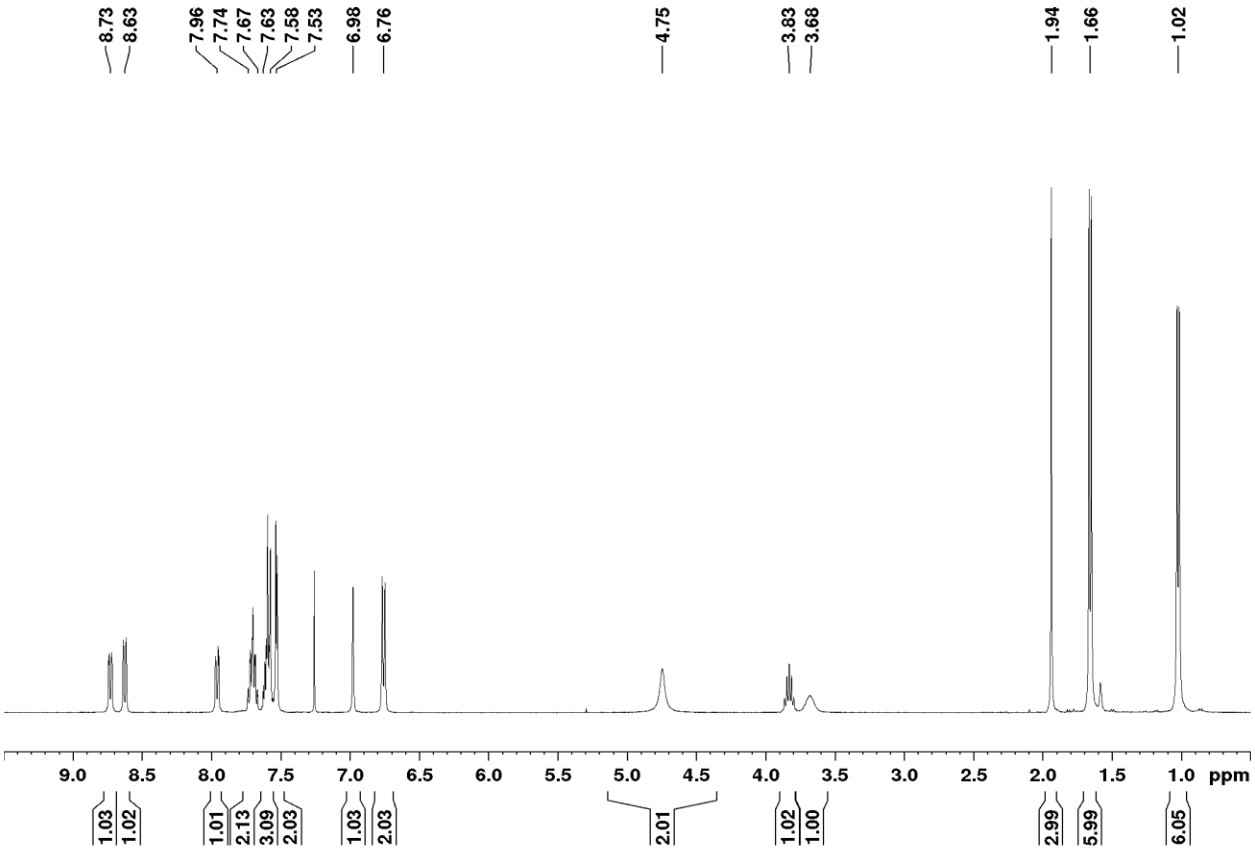

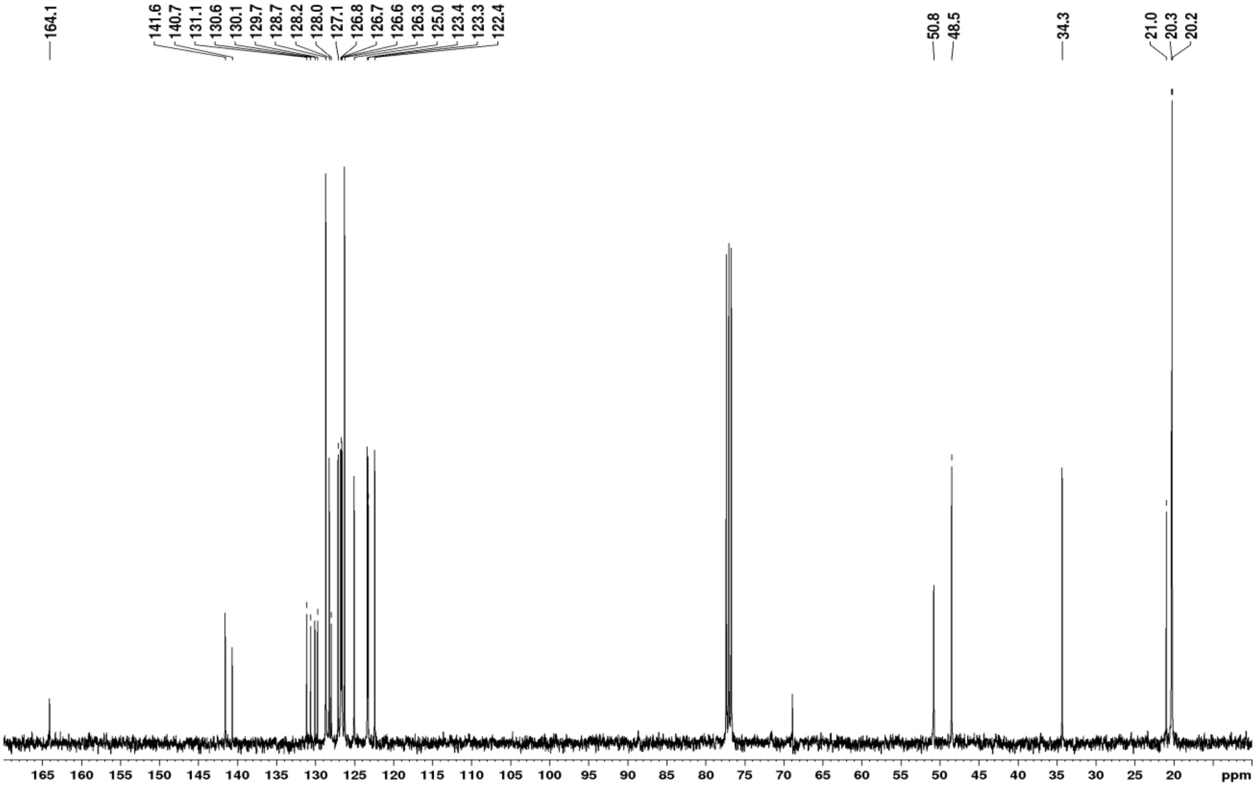

2-(4-(2-(Diisopropylamino)-2-(tosylimino)ethyl)phenyl)-*N*,*N*-diisopropyl-*N'*-tosylacetimidamide (11j)

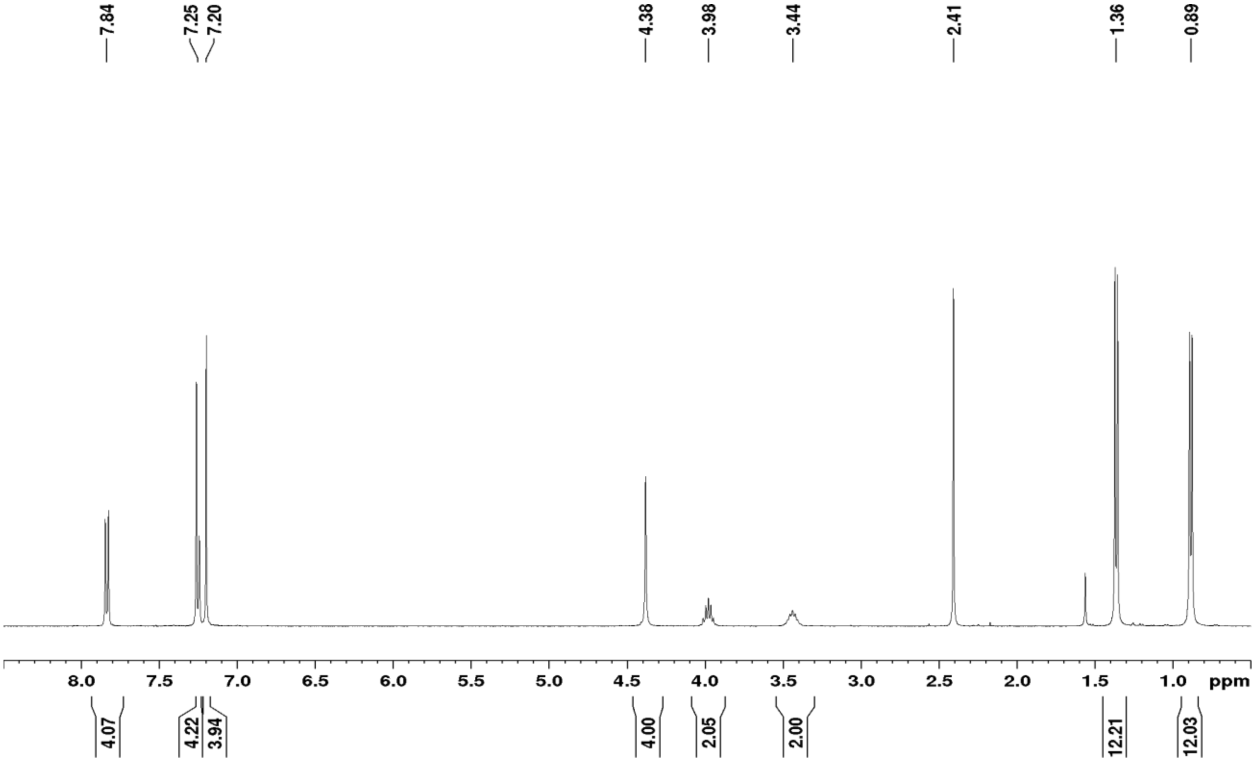

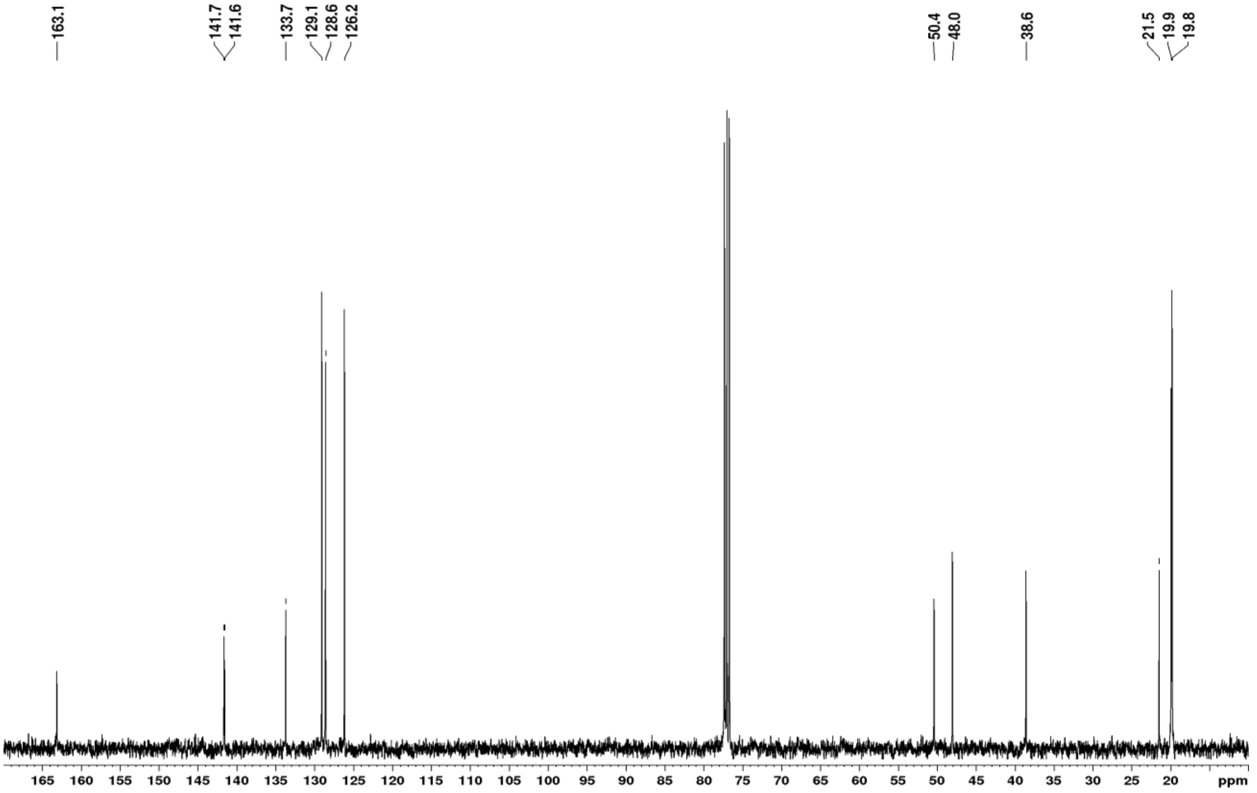

2-(3-(2-(Diisopropylamino)-2-(tosylimino)ethyl)phenyl)-*N,N*-diisopropyl-*N'*-tosylacetimidamide (11k)

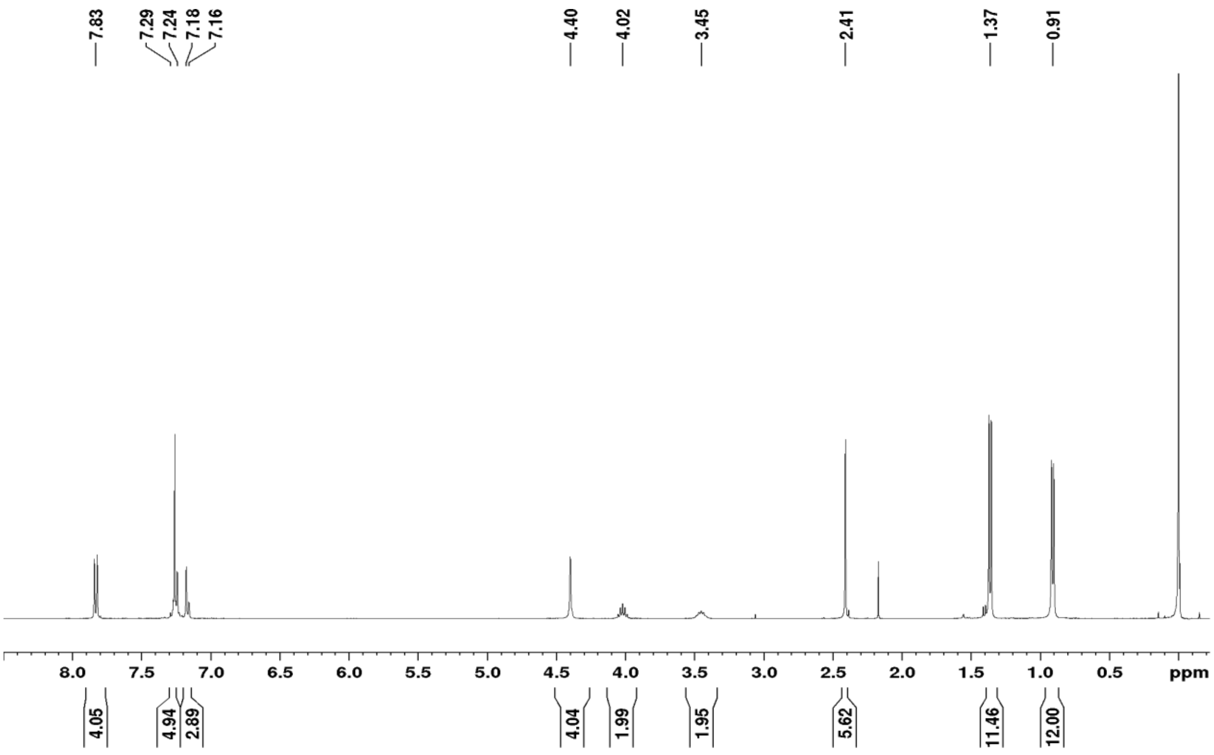

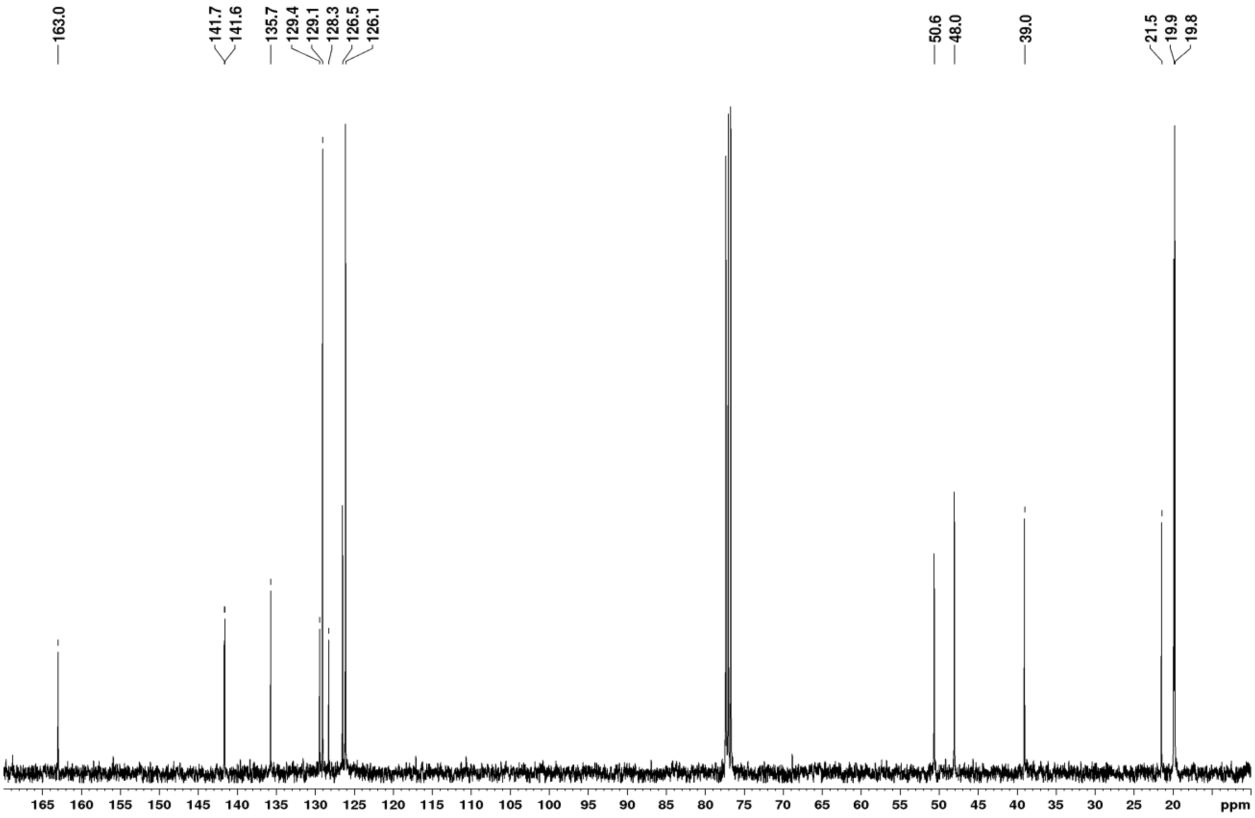

***N,N*-Diisopropyl-*N'*-tosyloctanimidamide**[8] **(11l)**

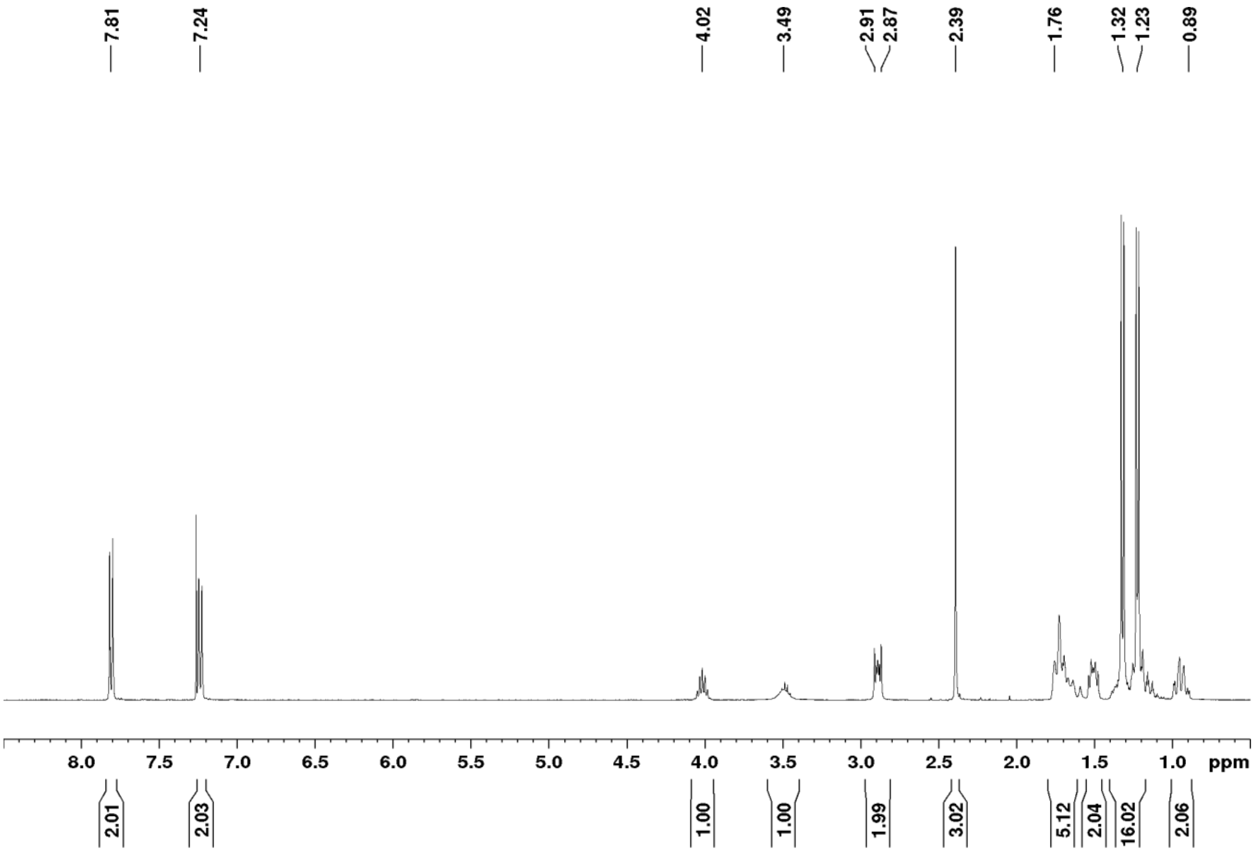

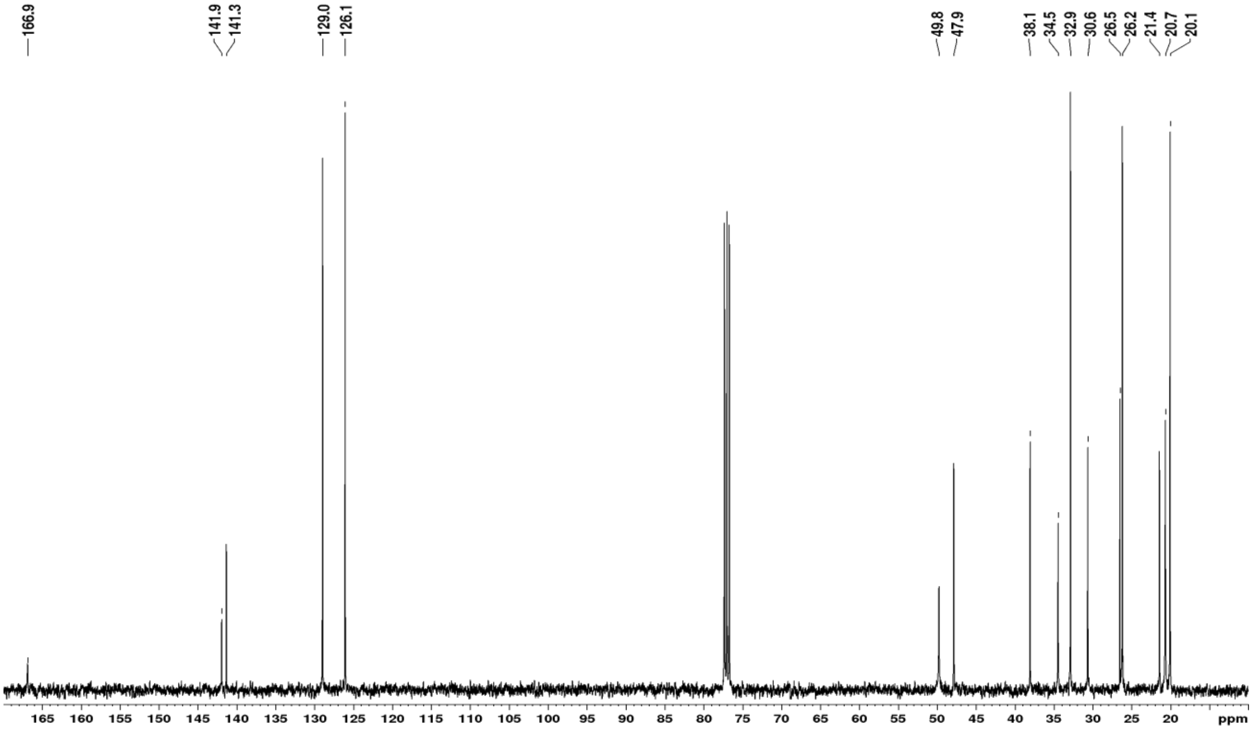

4-Bromo-*N,N*-diisopropyl-*N'*-tosylbutanimidamide (11m)

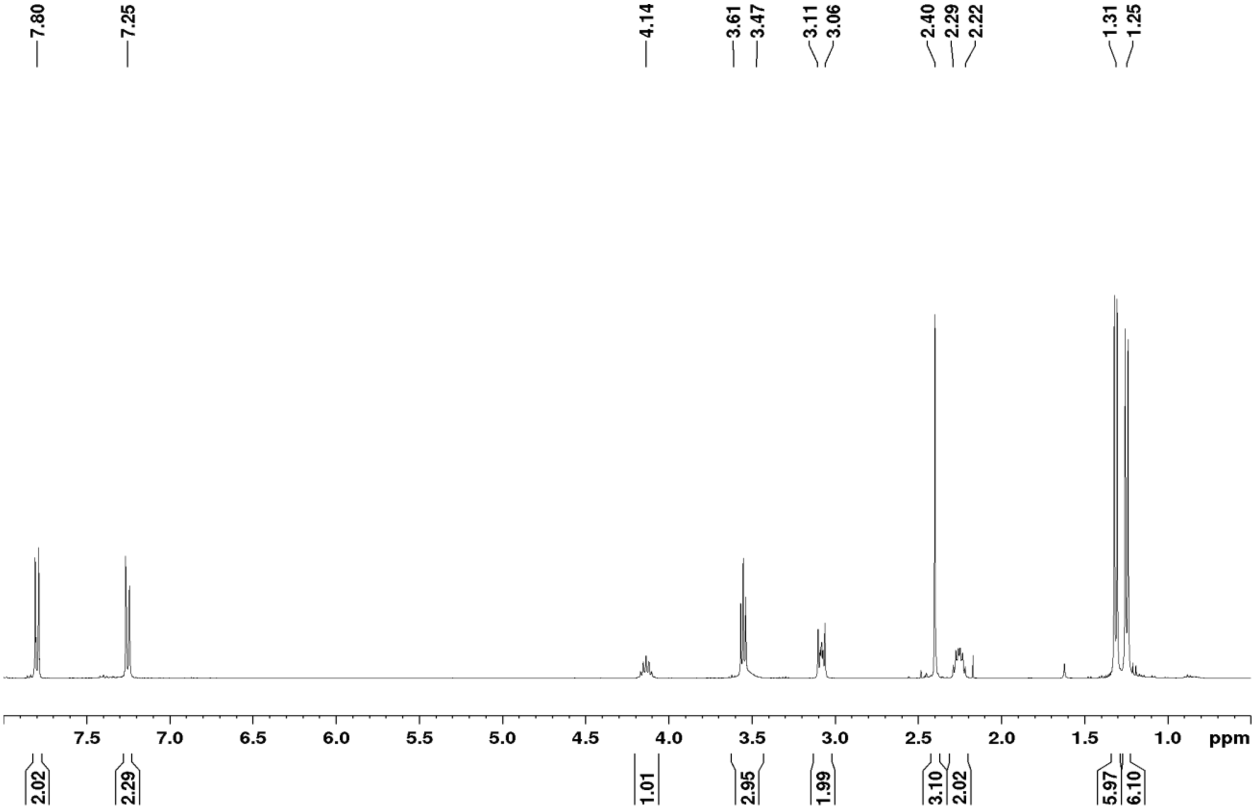

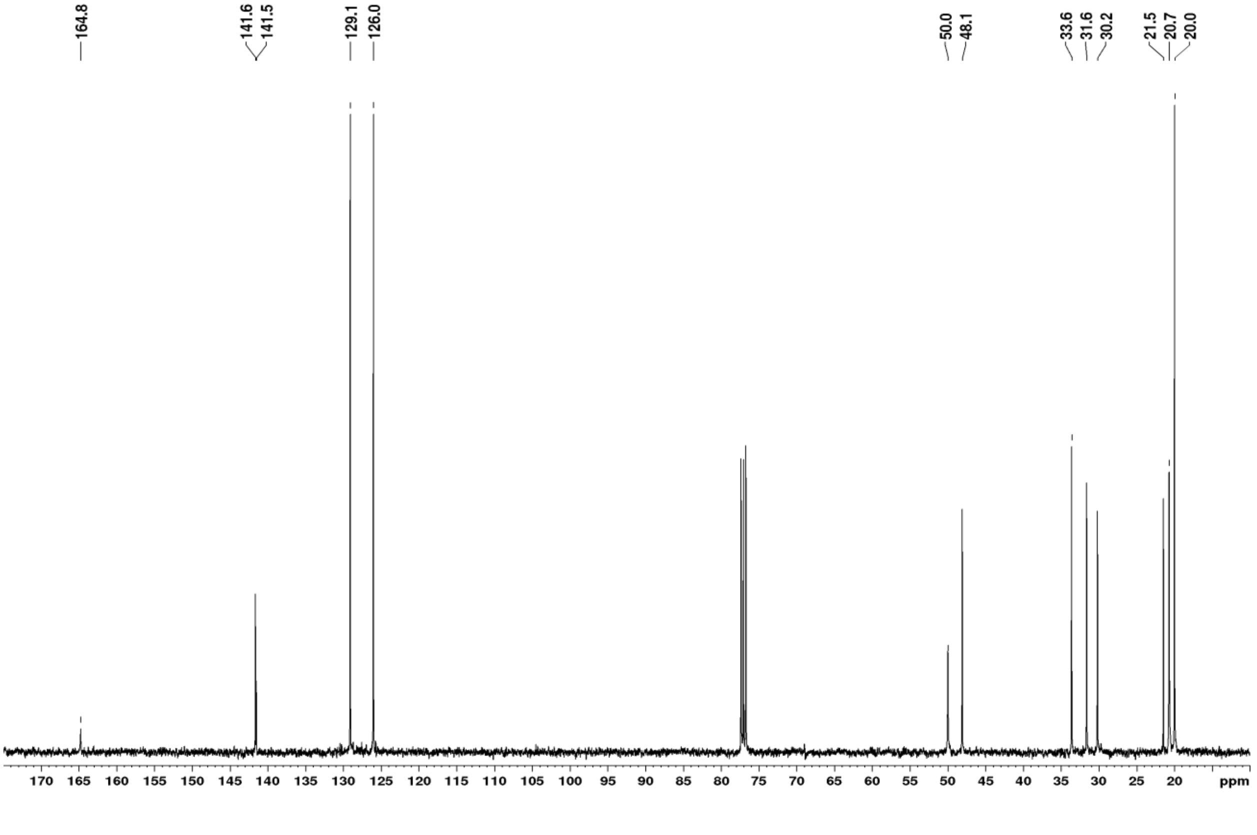

6-Chloro-*N,N*-diisopropyl-*N'*-tosylhexanimidamide (11n)

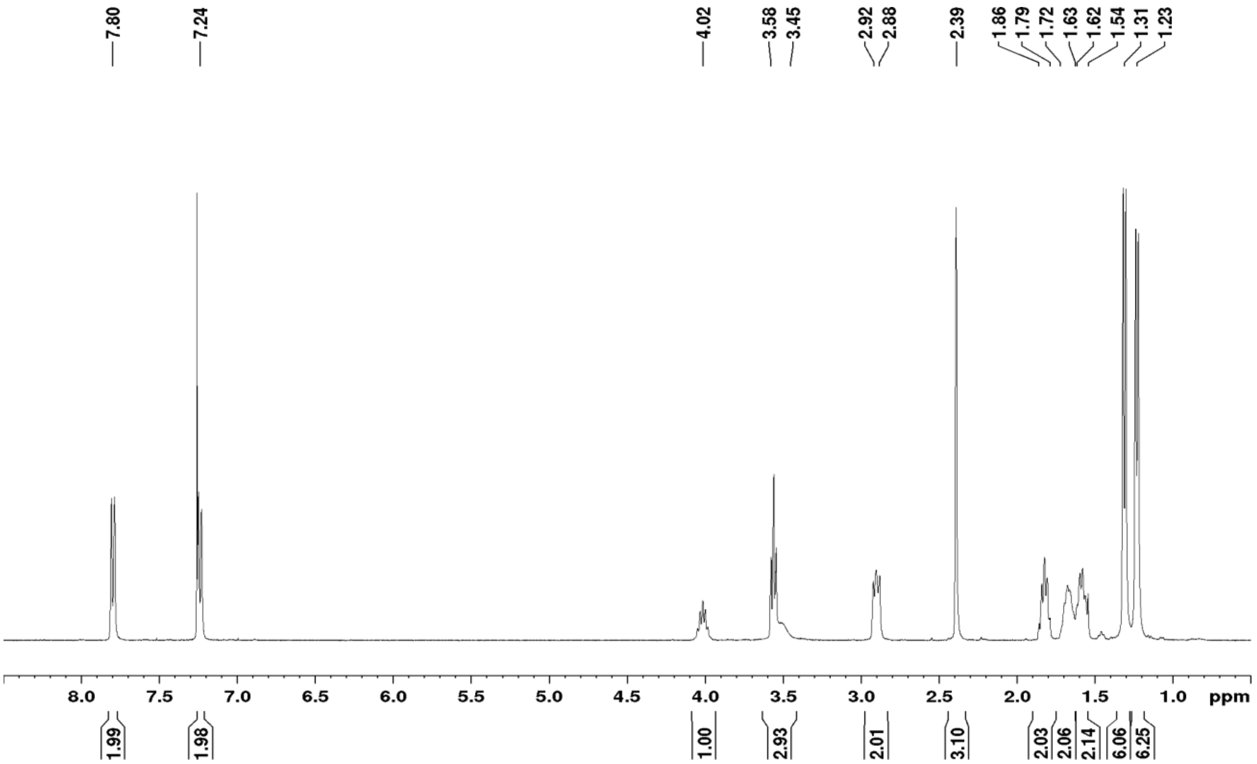

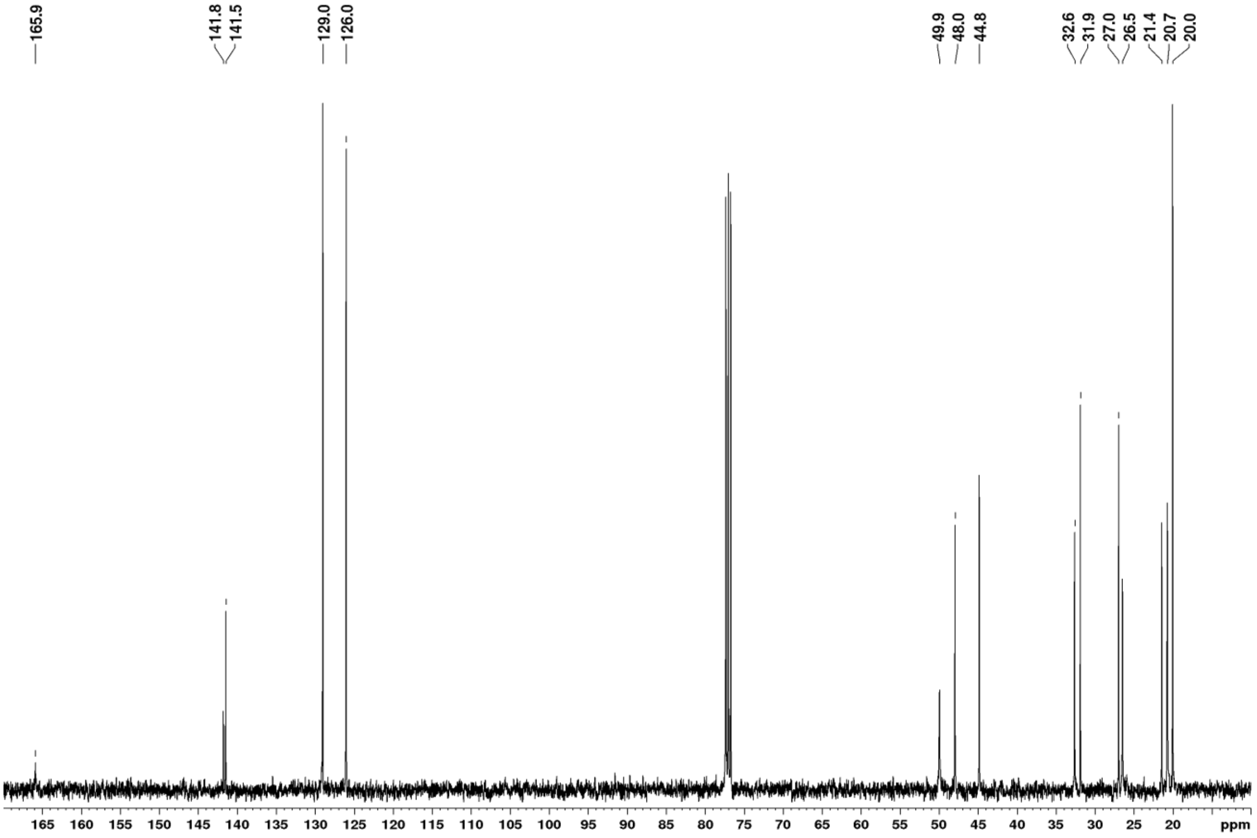

5-Chloro-*N,N*-diisopropyl-*N'*-tosylpentanimidamide[4] (11o)

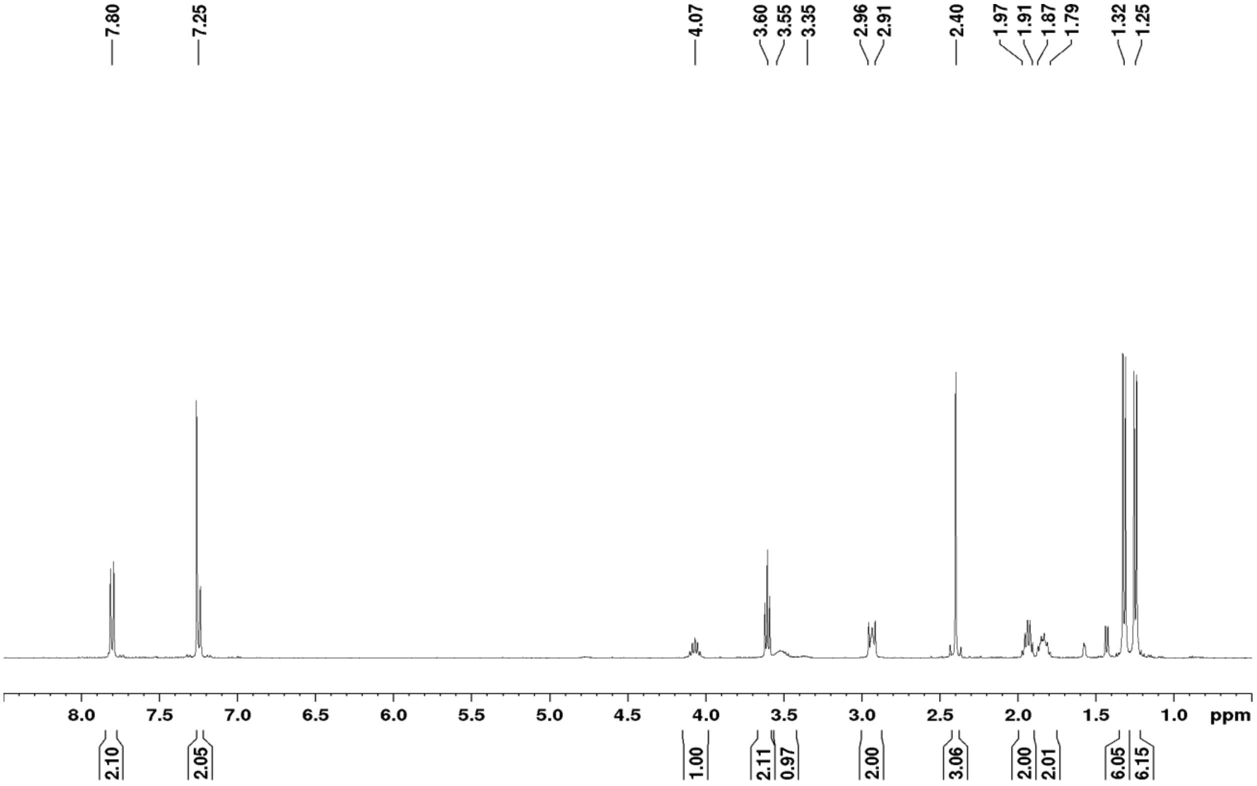

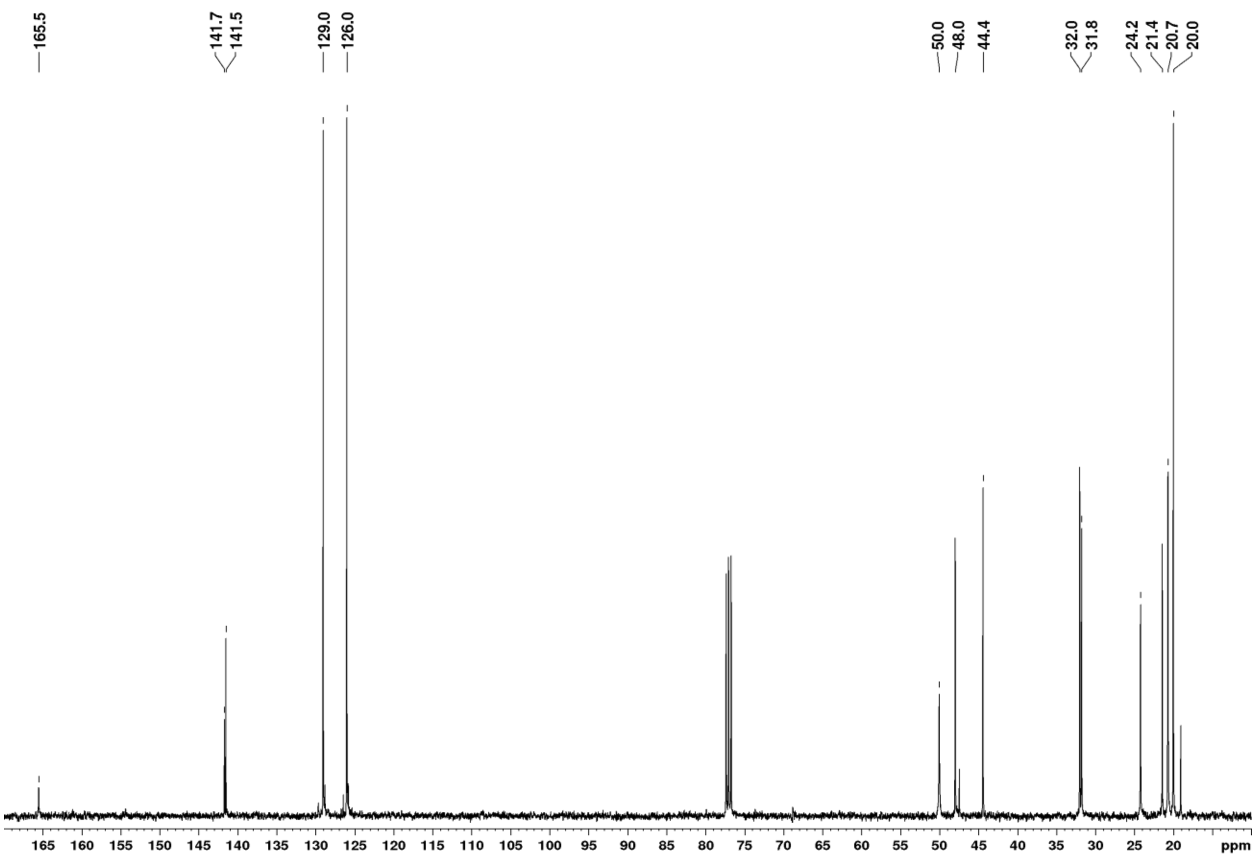

4-Cyclohexyl-*N,N*-diisopropyl-*N'*-tosylbutanimidamide (11p)

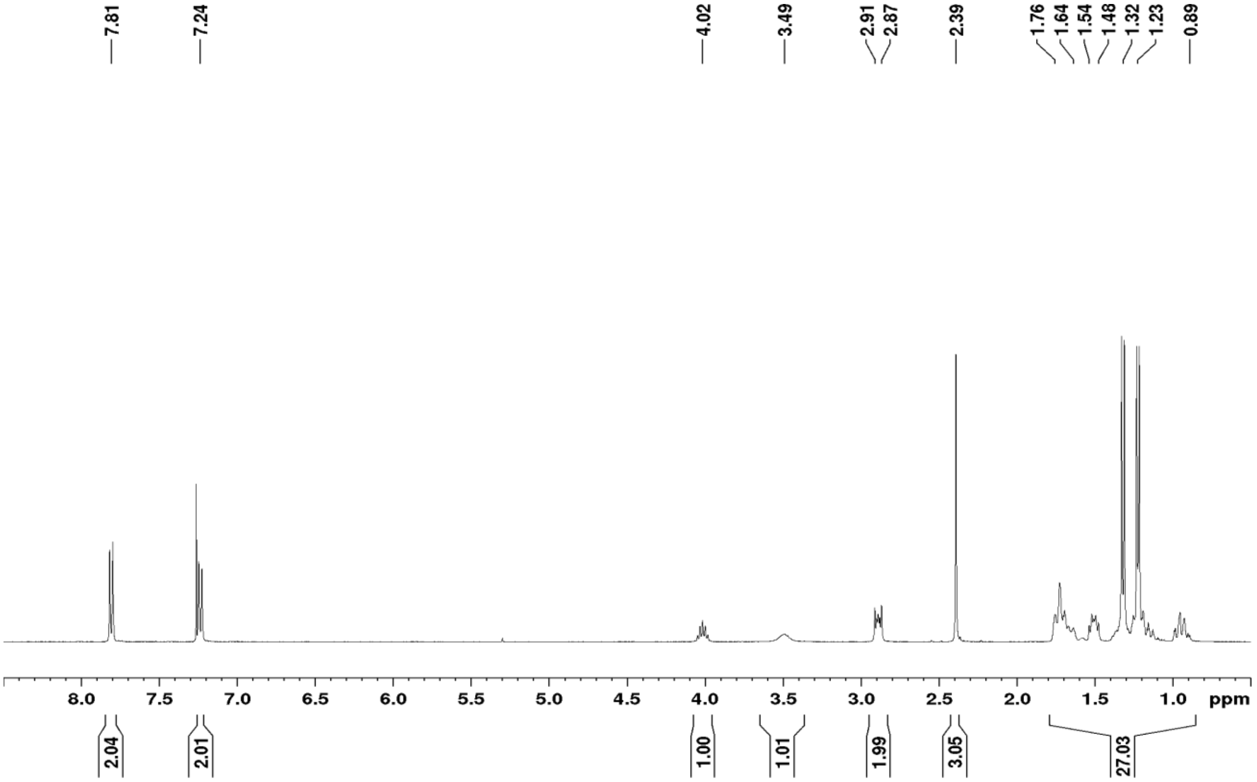

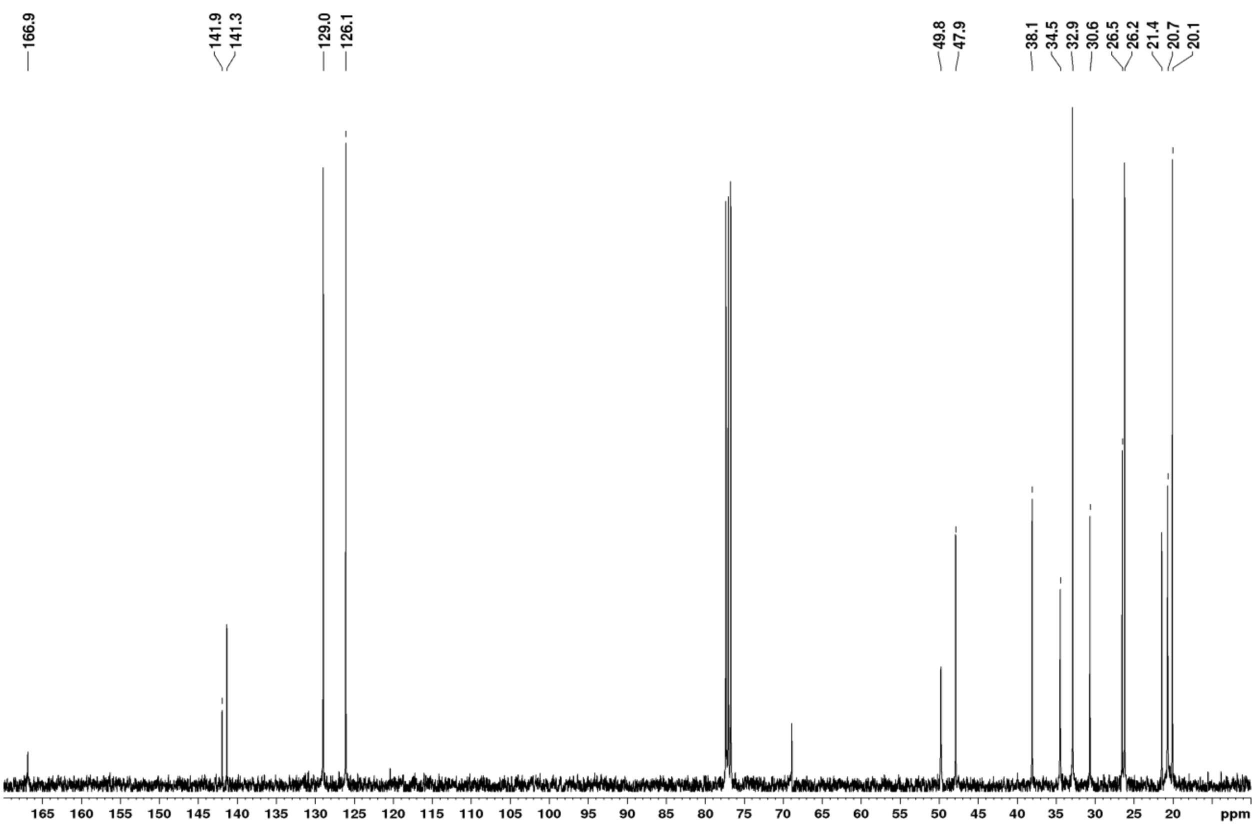

***N,N*-Diisopropyl-4-phenyl-*N'*-tosylbutanimidamide (11q)**

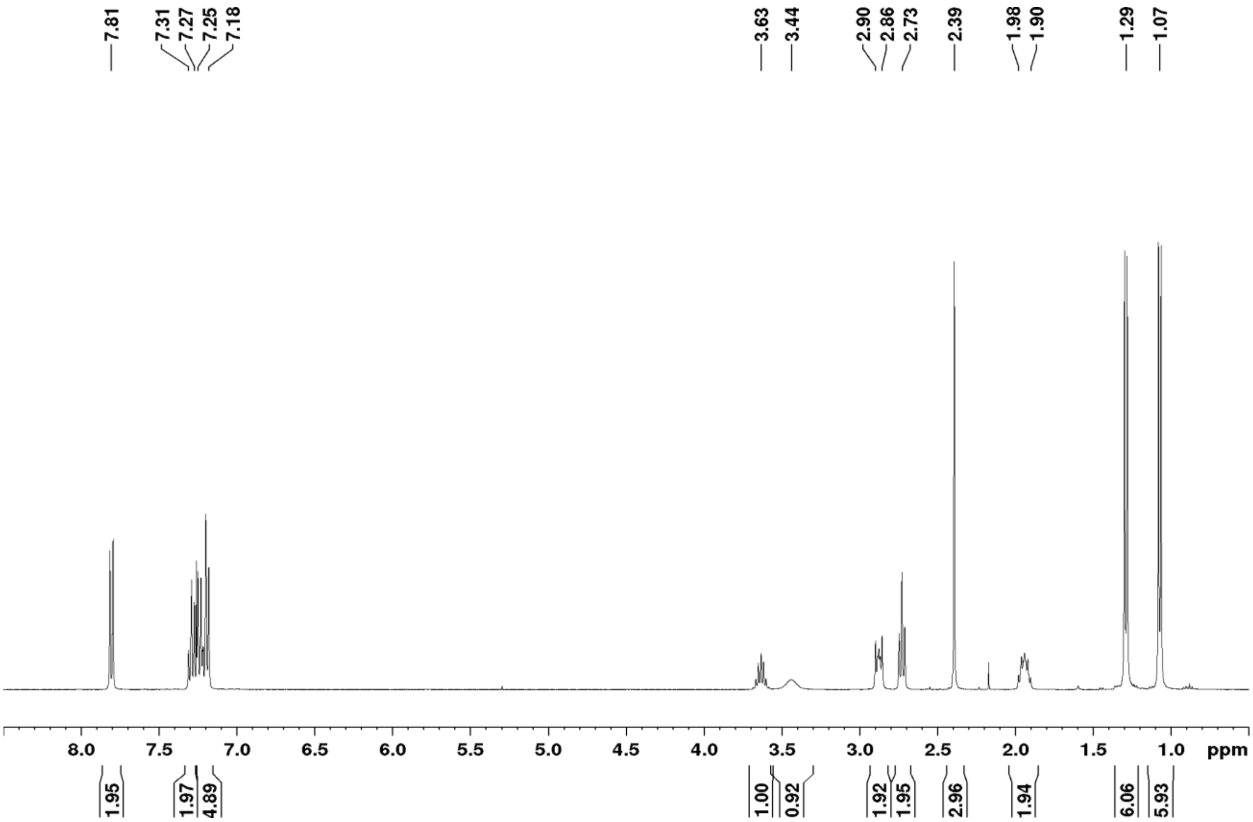

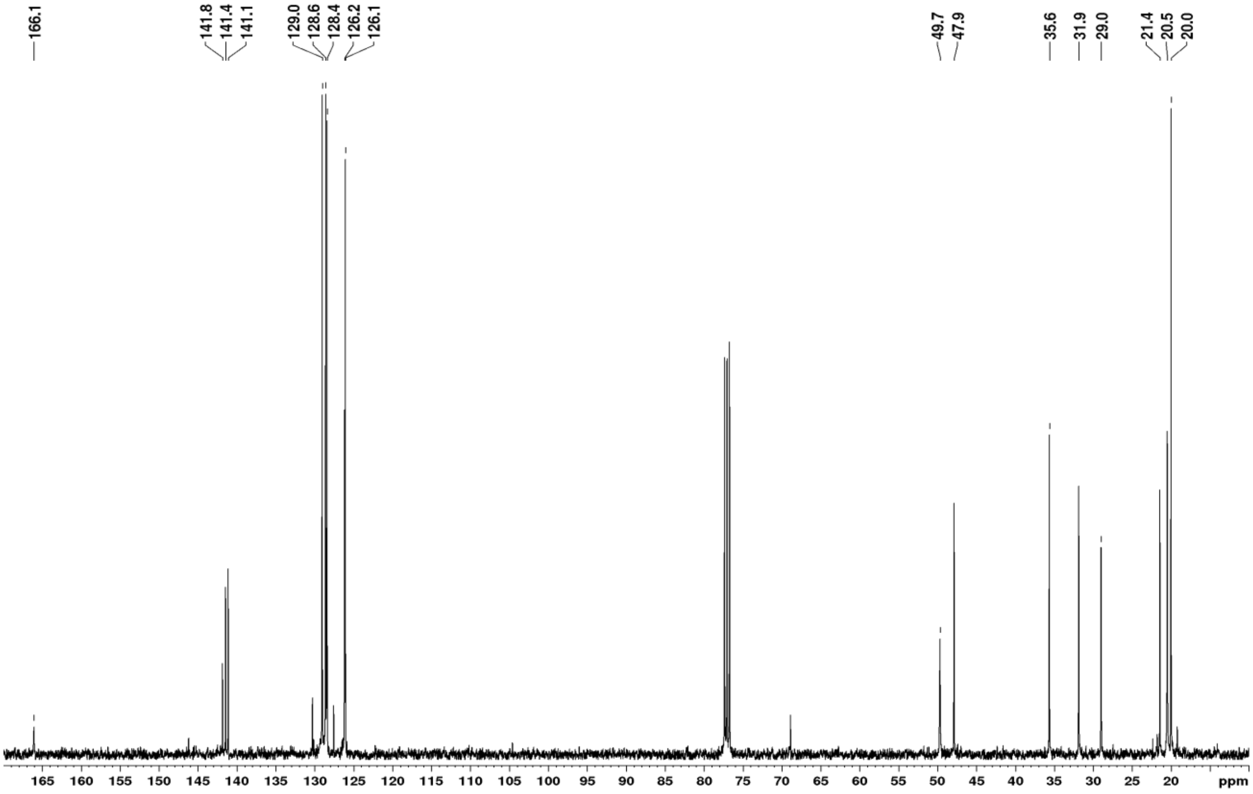

3-(Diisopropylamino)-1-phenyl-4-tosylbut-3-en-1-ol (11r)

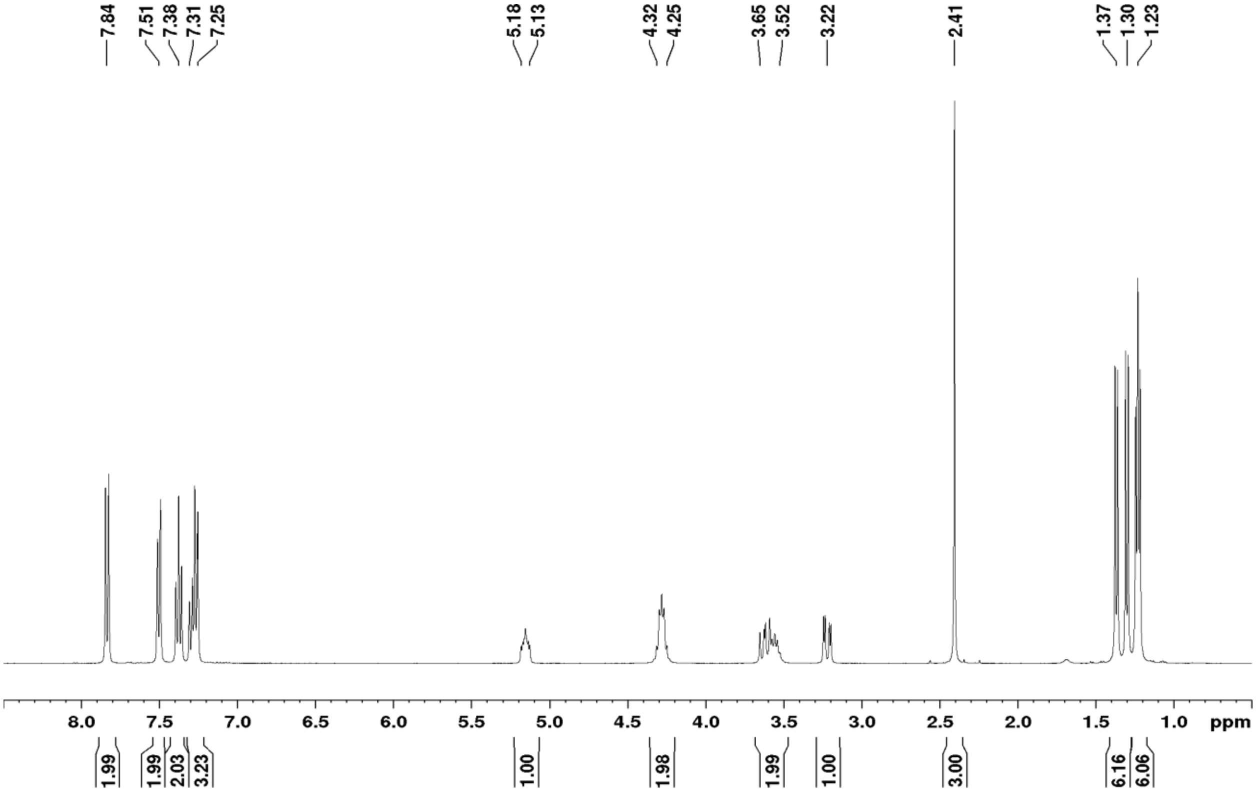

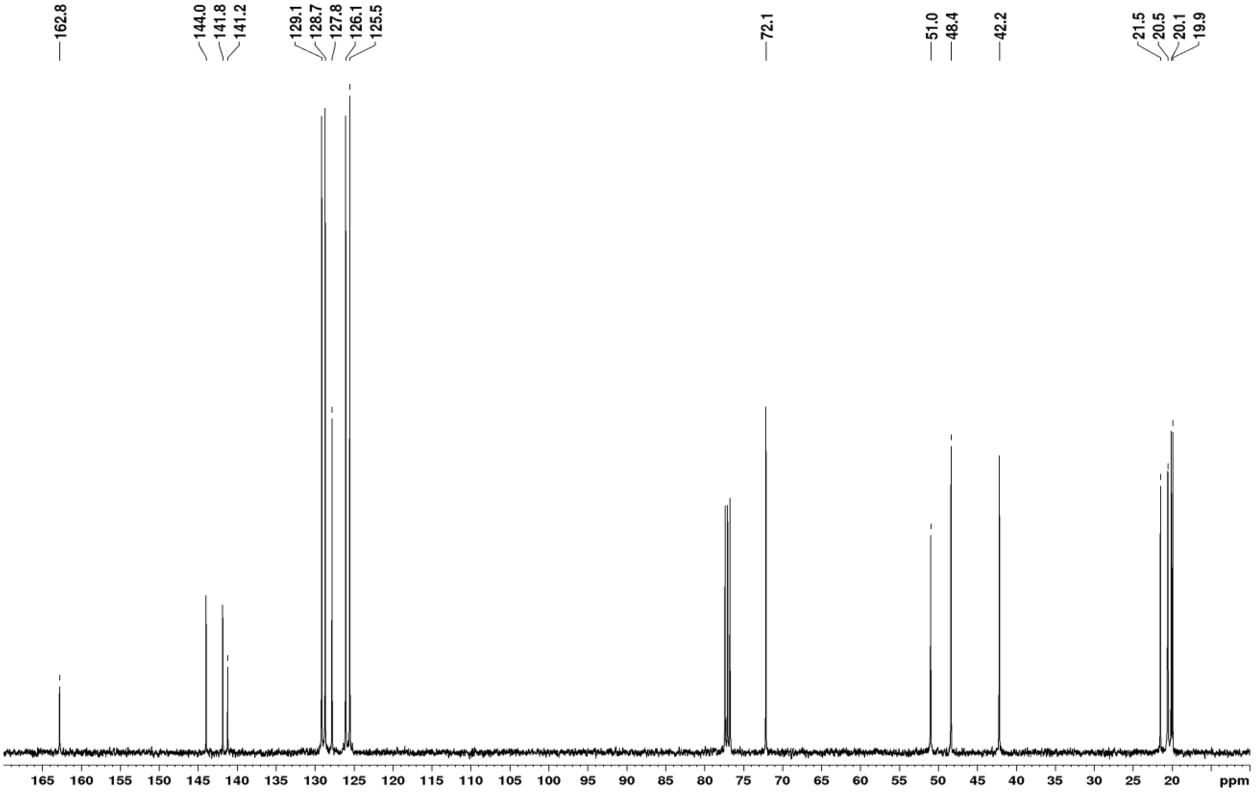

***N,N*-Dicyclohexyl-2-phenyl-*N'*-tosylacetimidamide (12a)**

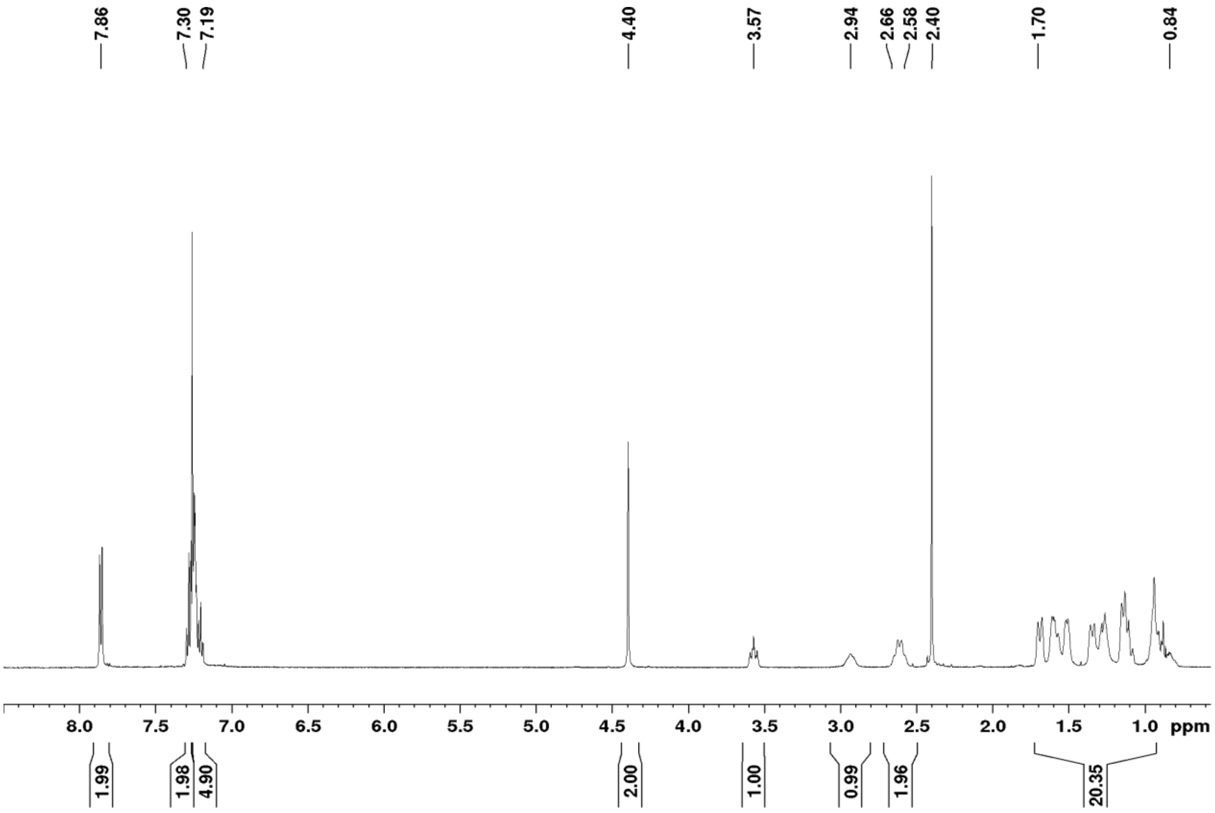

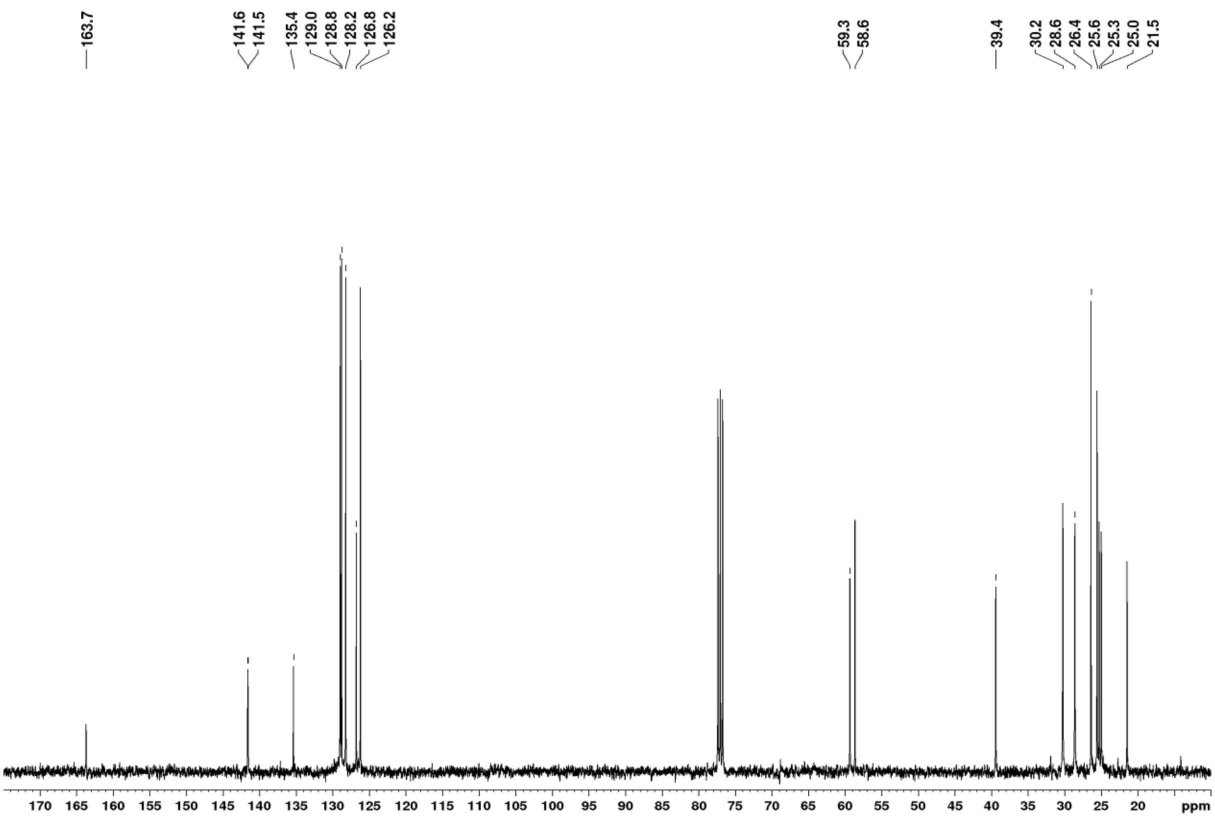

N-Isopropyl-2-phenyl-N'-tosylacetimidamide[4] (12b)

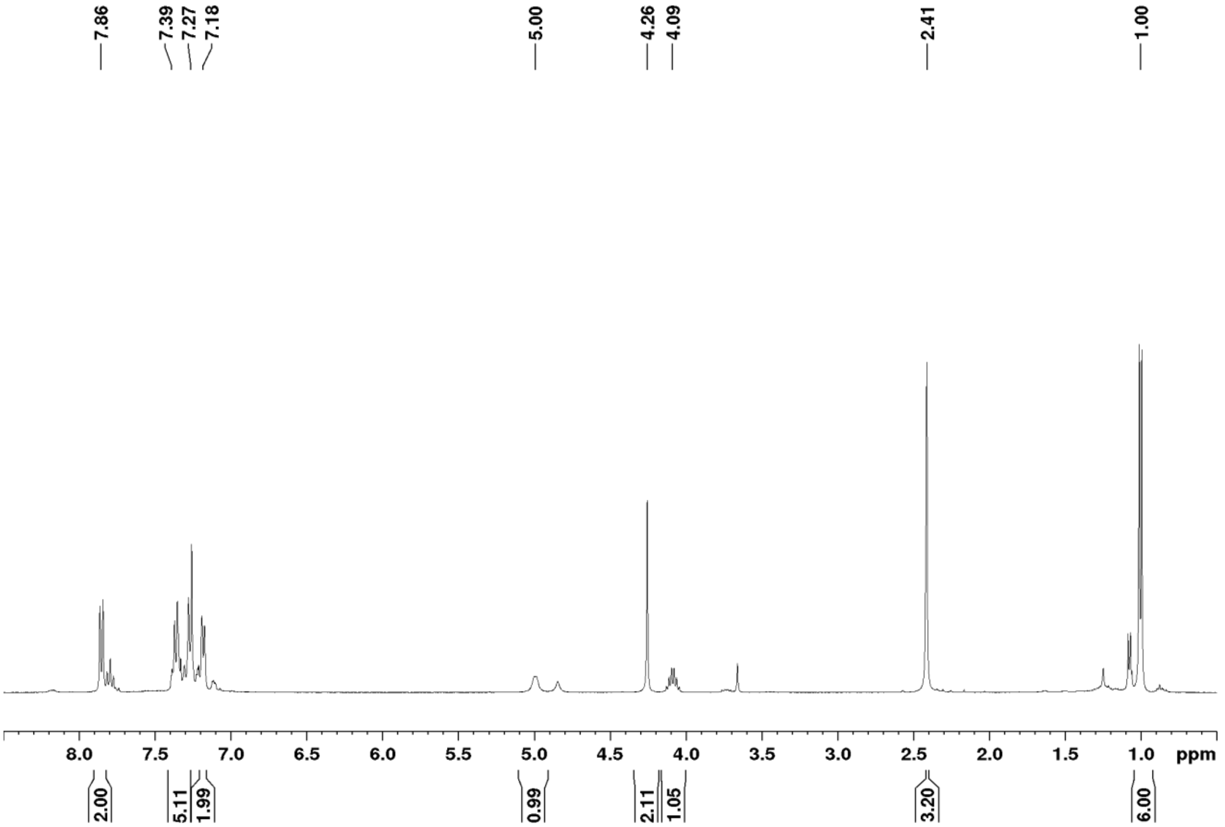

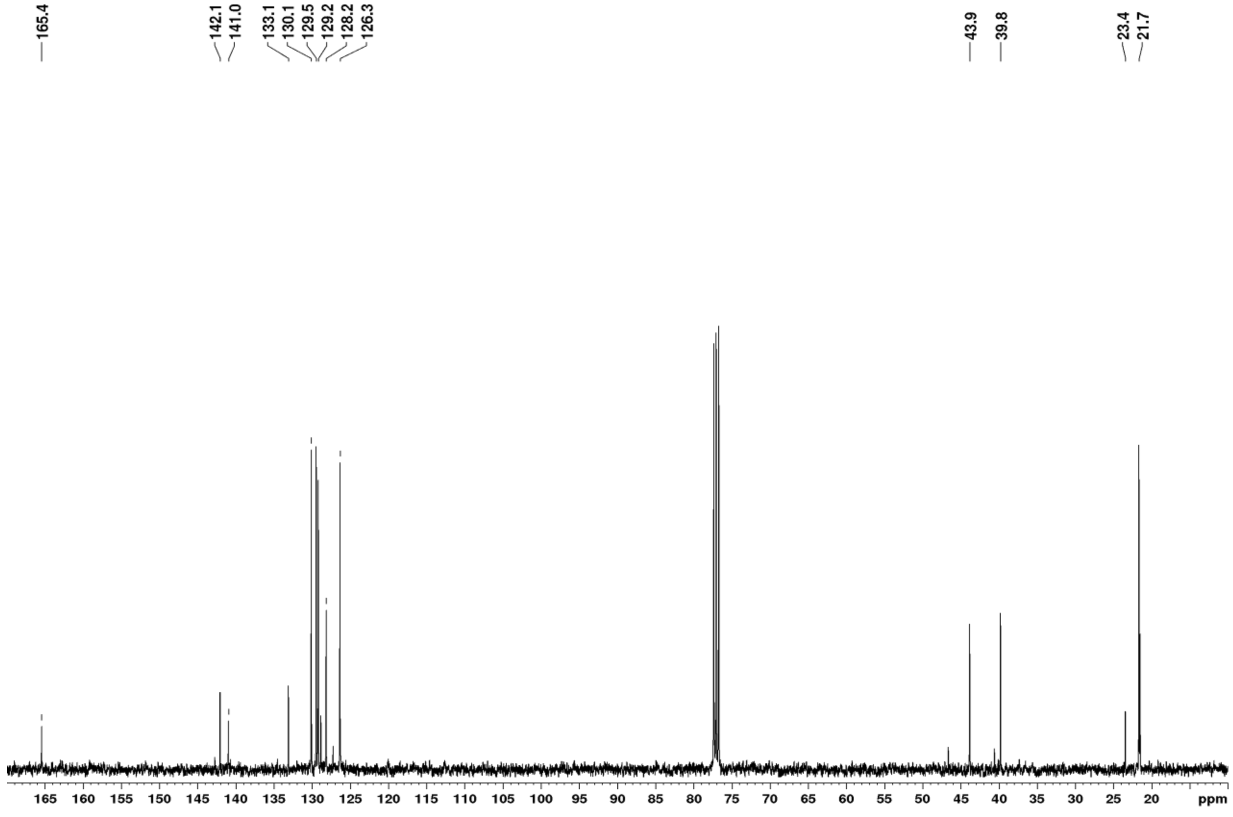

Diphenyl (1-(diisopropylamino)-2-phenylethylidene)phosphoramidate[6]

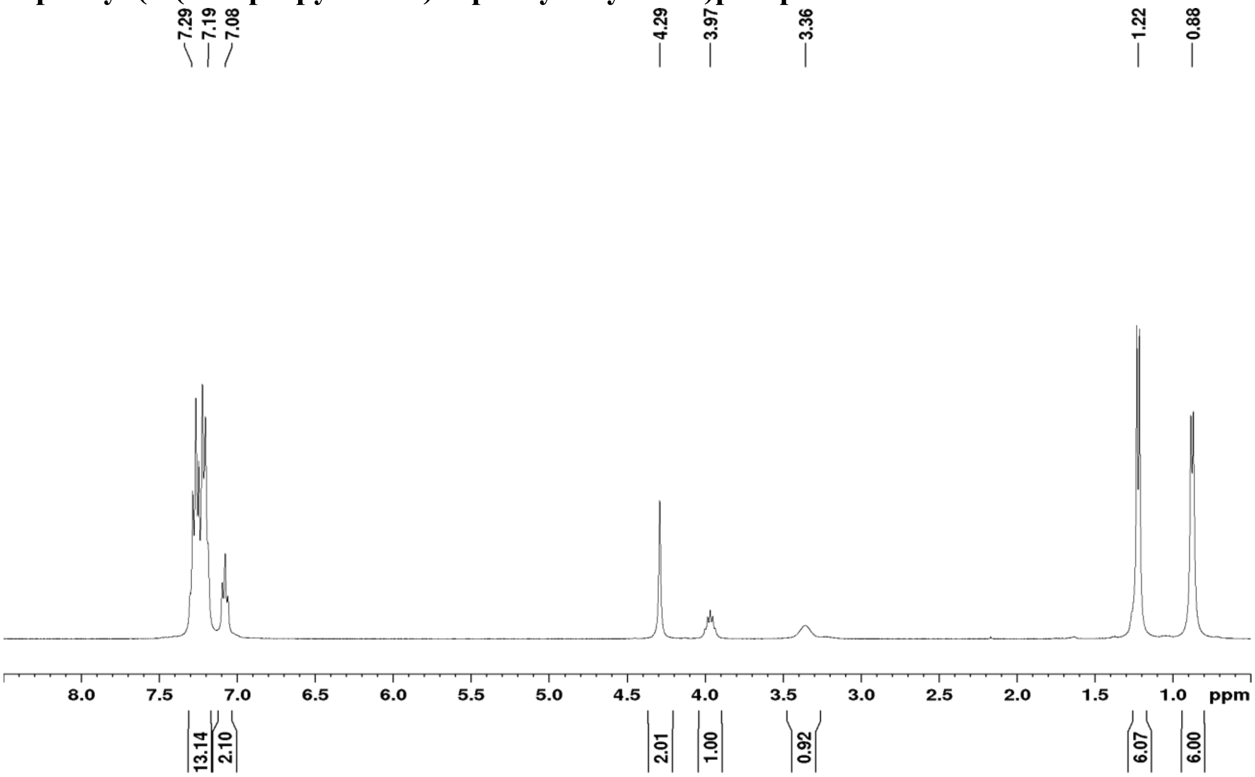

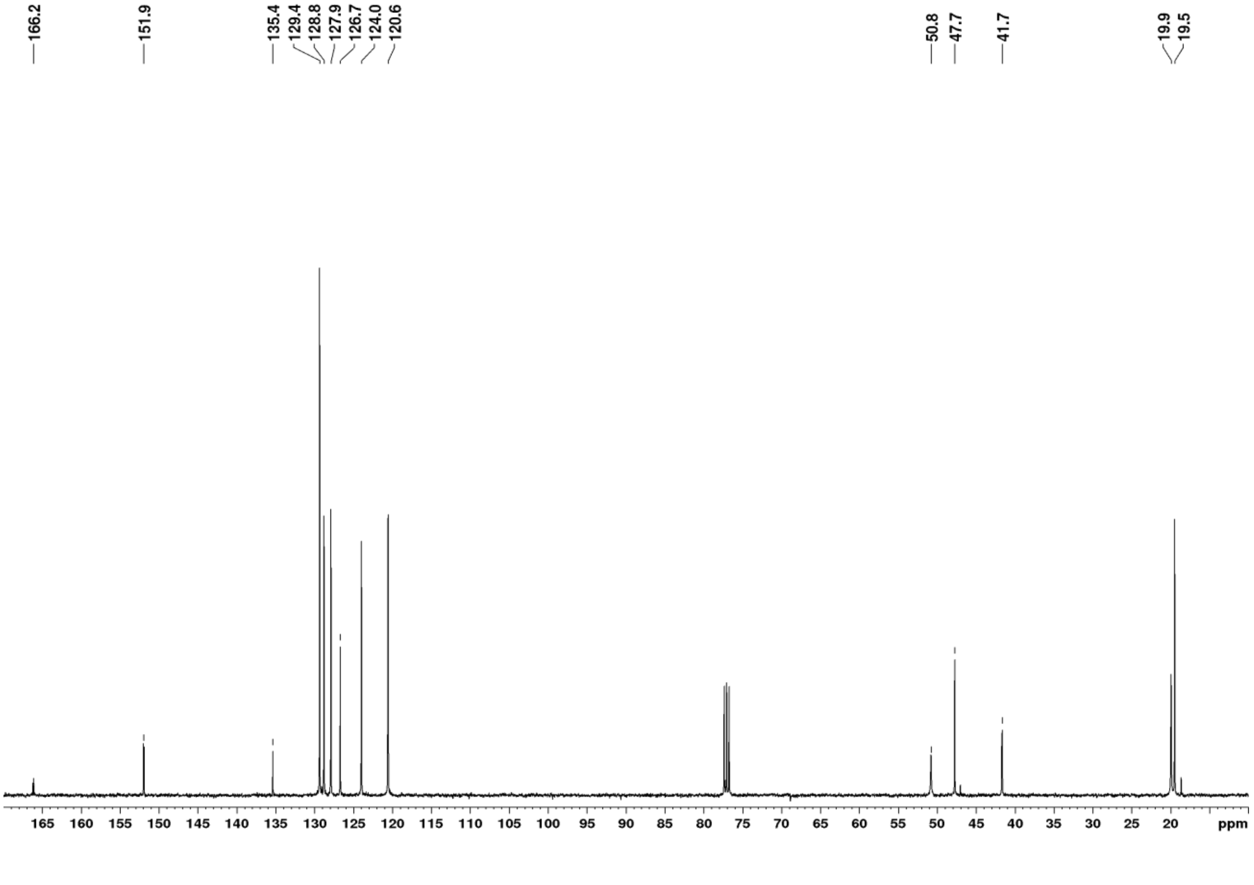

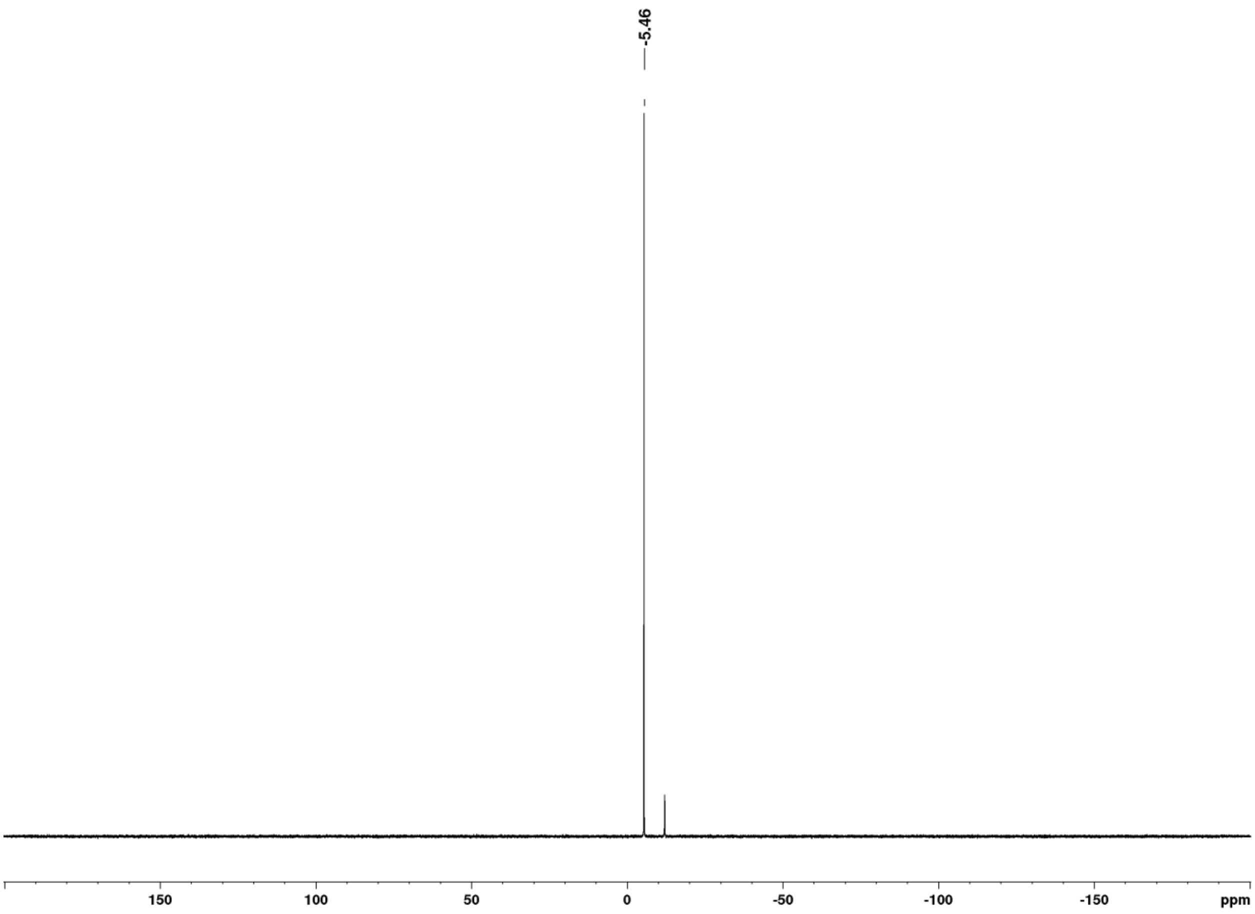

7. References

1 Ruppel, J. V. ; Jones, J. E.; Huff, C. A.; Kamble, R. M.; Chen, Y. and Zhang, X. P. *Org. Lett.*, 2008, **10**, 1995–1998.

2 Hogan, P. J. and Cox, B. G. *Org. Process Res. Dev.*, 2009, **13**, 875–879.

3 Lazreg, F.; Cordes, D. B.; Slawin, A. M. Z. and Cazin, C. S. J. *Organometallics*, 2015, 34, 419-425.

4 Bae, I.; Han, H. and Chang, S. *J. Am. Chem. Soc.*, 2005, **127**, 2038–2039.

5 Yoo, E. J.; Ahlquist, M.; Bae, I.; Sharpless, K. B.; Fokin V. V. and Chang, S. *J. Org. Chem.*, 2008, **73**, 5520–5528.

6 Kim, S. H.; Jung, D. Y. and Chang, S. *J. Org. Chem.*, 2007, **72**, 9769–9771.

7 Yang, T.; Cui, H.; Zhang, C.; Zhang L. and Su, C.-Y. *Inorg. Chem.*, 2013, **52**, 9053–9059.

8 DeKorver, K. A.; Johnson, W. L.; Zhang, Y.; Hsung, R. P.; Dai, H.; Deng, J.; Lohse, A. G.and Zhang, Y.-S. *J. Org. Chem.*, 2011, **76**, 5092–5103.
